# Identification of Novel lncRNAs Related to Colorectal Cancer Through Bioinformatics Analysis

**DOI:** 10.1155/bmri/5538575

**Published:** 2025-01-29

**Authors:** Razieh Heidari, Vahideh Assadollahi, Seyedeh Negar Marashi, Fatemeh Elahian, Seyed Abbas Mirzaei

**Affiliations:** ^1^Cancer Research Center, Shahrekord University of Medical Sciences, Shahrekord, Iran; ^2^Department of Medical Biotechnology, School of Advanced Technologies, Shahrekord University of Medical Sciences, Shahrekord, Iran; ^3^Department of Tissue Engineering and Applied Cell Sciences, School of Advanced Technologies, Shahrekord University of Medical Sciences, Shahrekord, Iran; ^4^Student Research Committee, Shahrekord University of Medical Sciences, Shahrekord, Iran; ^5^Advanced Technologies Core, Baylor College of Medicine, Houston, Texas, USA

**Keywords:** biomarker, colorectal cancer, differentially expressed genes (DEGs), lncRNA, weighted gene coexpression network analysis (WGCNA)

## Abstract

Long noncoding RNA (lncRNA) plays a critical role in cancer cell proliferation, invasion, metastasis, and chemoresistance. The current study introduces novel lncRNAs in colorectal cancer (CRC) through bioinformatics analysis. GSE134834 CRC-related microarray of Gene Expression Omnibus (GEO) was analyzed to identify differentially expressed genes (DEGs) in CRC samples against normal samples. Analysis revealed 6763 DEGs (*p* < 0.05 and |log fold change (FC)| ≥ 0.5) that include differentially expressed mRNA (DEmRNA) and differentially expressed long noncoding RNA (DElncRNA). Novel lncRNAs were identified, and to better understand the biological function of the identified lncRNAs, gene modules were constructed using weighted gene coexpression network analysis (WGCNA), and finally, two modules for lncRNAs were obtained. The coexpression modules with these lncRNAs were subjected to enrichment analysis in FunRich software to predict their functions through their coexpressed genes. Gene ontology results of modules related to novel lncRNA revealed they significantly enriched the cellular pathways regulation in cancer. The protein–protein interaction (PPI) network of novel lncRNAs-related modules was constructed using Search Tool for the Retrieval of Interacting Genes (STRING) and visualized using the Cytoscape software. Hub genes were screened from the PPI network by the CytoHubba plug-in of Cytoscape. The hub genes were *MRTO4*, *CDK1*, *CDC20*, *RPF2*, *NOP58*, *NIFK*, *GTPBP4*, *BUB1*, *BUB1B*, and *BOP1* for the lightpink4 module and *BYSL*, *RPS23* (ribosomal protein S23), *RSL1D1* (ribosomal L1 domain containing 1), *NAT10*, *NOP14*, *GNL2*, *MRPS12*, *NOL6* (nucleolar protein 6), *IMP4*, and *RRP12* (ribosomal RNA processing 12 homolog) for the pink module. The expression levels of the top DEmRNA and module hub genes in CRC were validated using the Gene Expression Profiling Interactive Analysis (GEPIA) database. Generally, our findings offer crucial insight into the hub genes and novel lncRNAs in the development of CRC by bioinformatics analysis, information that may prove useful in the identification of new biomarkers and treatment targets in CRC; however, more experimental investigation is required to validate the findings of the present study.

## 1. Introduction

Colorectal cancer (CRC) is the most common malignant neoplasm and is among the most life-threatening problems [1]. Identifying and studying genetic and epigenetic changes in cancer improve diagnostic ways and provide new therapeutic targets [2]. Around 70% of the human genome is transcribed into RNA, but only 2% is translated into protein. Nontranslated RNAs are called noncoding RNAs (ncRNAs). Some ncRNAs are abundant, like tRNAs and rRNAs, but some are rare and functionally important, including short RNAs (miRNA, small interfering RNA (siRNA), short hairpin RNA (shRNA), etc.) and long noncoding RNAs (lncRNAs). lncRNAs are a less-studied class of ncRNAs with lengths of 0.2–100 kb [3–5].

The number of lncRNAs transcribed from the genome competes with the protein-coding genes in humans. Public databases such as LNCipedia and LncRNADB have reported thousands of sets of lncRNAs with unknown functions [6, 7]. Scientists uncovered that lncRNA expressions are altered in tumor cells. They may exert oncogenic or tumor suppressor functions [8–10]. lncRNAs can be used as tumor biomarkers or therapeutic targets in cancer because they are tissue-specific, highly stable, and abundant in body fluids that can be measured in small blood, urine, or saliva samples using a simple real-time PCR (RT-PCR) technique. Measurement of the overexpressed lncRNA of prostate cancer gene-3 (PCA3) in urine has been approved by the FDA as a diagnostic tool for prostate cancer [11, 12].

Novel computational approaches to large databases have made complex genomic studies easier and faster [13]. The microarray and RNA-seq analysis methods are high-throughput approaches that can measure the total RNA concentrations in any specimens and make them ideal techniques for targeting unknown lncRNAs in pathological human diseases. This technology can simultaneously identify the differential expression patterns of several thousand specific sequences or coding regions. Microarrays have been widely applied in cancer research for the comparison of gene expression profiles between tumor and normal samples [14]. Traditional differential expression analyses have been performed to translate the microarray information into biological concepts. One in silico system biology tool used to perform microarray analysis is the weighted gene coexpression network analysis (WGCNA), which identifies gene modules. A module is a cluster of highly correlated genes with similar coexpression patterns [15]; thus, WGCNA analyzes the coexpression behavior of a set of genes instead of a single gene [16].

**Figure 1:**
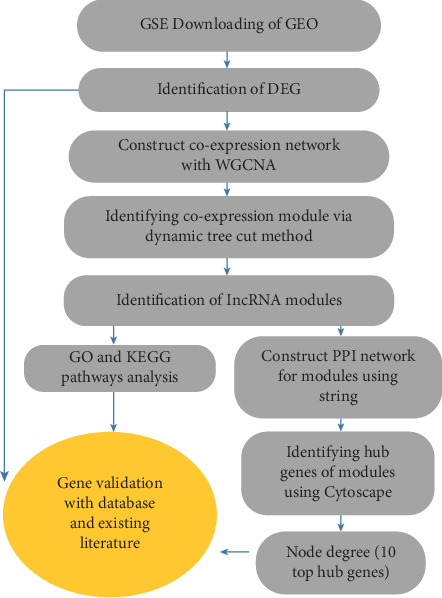
Flow diagram of data analysis.

In recent years, WGCNA has been successfully applied to uncover the potential biomarkers in various cancer types. By WGCNA, the RP11-164P12.5, RP11-33O4.1, PCGF5, and CTD-2396E7.11 lncRNAs have been reported to be involved in the development of colon adenocarcinoma [17]. FBXW4 has been demonstrated to be related to chemotherapy resistance and prognosis of CRC [18]. COL8A1 has been shown to be associated with prognosis and progression of CRC. WGCNA has also been performed to introduce key genes in CRC recurrence [19]. In one WGCNA study, 15 hub genes were identified as candidate biomarkers in breast cancer [20]. In the current study, Genomic Spatial Event (GSE) microarray data related to CRC patients was extracted from Gene Expression Omnibus (GEO) to examine CRC-related differentially expressed genes (DEGs) using the limma package in R software. Novel lncRNAs were found, and gene modules were constructed using WGCNA to investigate the function of these new lncRNAs. The gene ontology (GO) of the gene modules was identified using the FunRich tool (http://www.funrich.org). A protein–protein interaction (PPI) network of module genes was constructed using Search Tool for the Retrieval of Interacting Genes (STRING) and was visualized using Cytoscape software. Hub genes were screened from the PPI network by the CytoHubba plug-in of Cytoscape. The 10 genes with the highest degree were selected as hub genes (Figure 1).

## 2. Methodology

### 2.1. Data Collection

The GSE134834 microarray associated with CRC was downloaded from the GEO database (http://www.ncbi.nlm.nih.gov/geo) to identify the DEGs, including mRNA and novel lncRNAs. GSE134834 contains 10 samples (five of adjacent normal tissue and five of CRC tissue). The Agilent-067406 Human CBC lncRNA+mRNA microarray (V4.0) was the identified dataset platform.

### 2.2. Preprocessing and Analysis of Raw Dataset

The limma package was used from Bioconductor (http://www.bioconductor.org/) with R software (Version 4.3.3) for preprocessing of the gene expression profile data. The expression values were transformed into log fold change (FC) values, and the quantile normalization method was used to acquire a normalized expression matrix of the datasets. The cut-off criteria for screening DEGs were based on the *p* value and |log (FC)| by comparing expression values between tumor and normal samples (*p* > 0.05 and |log (FC)| > 0.5). The expression levels of the top genes were validated using the Gene Expression Profiling Interactive Analysis (GEPIA) database (http://gepia2.cancer-pku.cn). The top differentially expressed long noncoding (DElnc) was identified based on the defined criteria.

Some dysregulated lncRNAs with their names mentioned in Tables 1 and 2 were searched in the LNCipedia and GeneCard databases, and literature were reviewed in Google Scholar for these lncRNAs in CRC. lncRNAs without reports in CRC could be investigated as novel cases. Gene expression was examined using the GEPIA database, specifically designed for cancer research, to verify the expression patterns of DEGs identified in our analysis. The GEPIA database allows the exploration of data from the Cancer Genome Atlas (TCGA) and Genotype-Tissue Expression (GTEx) databases (http://gepia2.cancer-pku.cn/). Hub gene expression differences between normal tissues and CRC were evaluated using GEPIA's “expression DIY” tool. Statistical analysis was conducted using a significance threshold of *p* value < 0.01. This involved selecting the CRC dataset and inputting gene symbols into the provided text field to compare tumor and normal samples.

### 2.3. Construction of Gene Coexpression Network

The biological function of most lncRNAs is unknown; thus, their function mainly was predicted by examining the genes coexpressed with them. Network analysis was performed using WGCNA on the DEGs from the GSE dataset to determine the function of the lncRNAs by examining their comodule genes. The identification of outlier samples was performed using a hierarchical cluster. The similarity measurement denoted for each pair was Pearson's correlation coefficient.

The correlation matrix was converted into an adjacency matrix using the adjacency function and *β* power. The *β* (soft-threshold) power was calculated using the pickSoftThreshold function, and the scale-free topology curve was drawn. A topological overlap matrix (TOM) similarity function was then used to convert the adjacency matrix into TOM. After calculating the TOM matrix and obtaining the dissimilarity matrix, the gene modules are constructed using hierarchical clustering of at least 30 genes in each module (minModuleSize = 30) using the hclust criterion. The genes were grouped into modules using the dynamic tree-cut method. Each module is a cluster of genes having expression profiles that are highly significantly correlated. Finally, the modules with a high level of similarity were merged at the correlation level, having a threshold of 25%, and a dendrogram was drawn.

### 2.4. Enrichment Analysis of Modules

GO and the KEGG (Kyoto Encyclopedia of Genes and Genomes) pathways were applied to examine functional enrichment in a high-throughput genome or in transcriptome data. The GO concepts are the biological process (BP), molecular function (MF), and cellular component (CC). The FunRich tool (Version 3.1.3) was employed to determine the biological pathways of the modules [38]. A *p* value of < 0.05 was set for FunRich enrichment analysis.

### 2.5. PPI Network Analysis and Hub Gene Identification

The STRING database (http://string-db.org/) was used to evaluate the PPI network [39]. STRING finds possible interactions between the DEGs. To construct a PPI, the interaction score was set at a 0.07 confidence level with active interaction scores, experiments, and coexpression. The reconstructed coexpression PPI network containing the essential gene was observed using Cytoscape software (Version 3.7.0) and the CytoHubba plugin. The Top 10 genes with the highest degrees were selected as hub genes. The degree of a node indicates the number of its connections with other nodes [40].

## 3. Results

### 3.1. Identification of DEG

**Figure 2:**
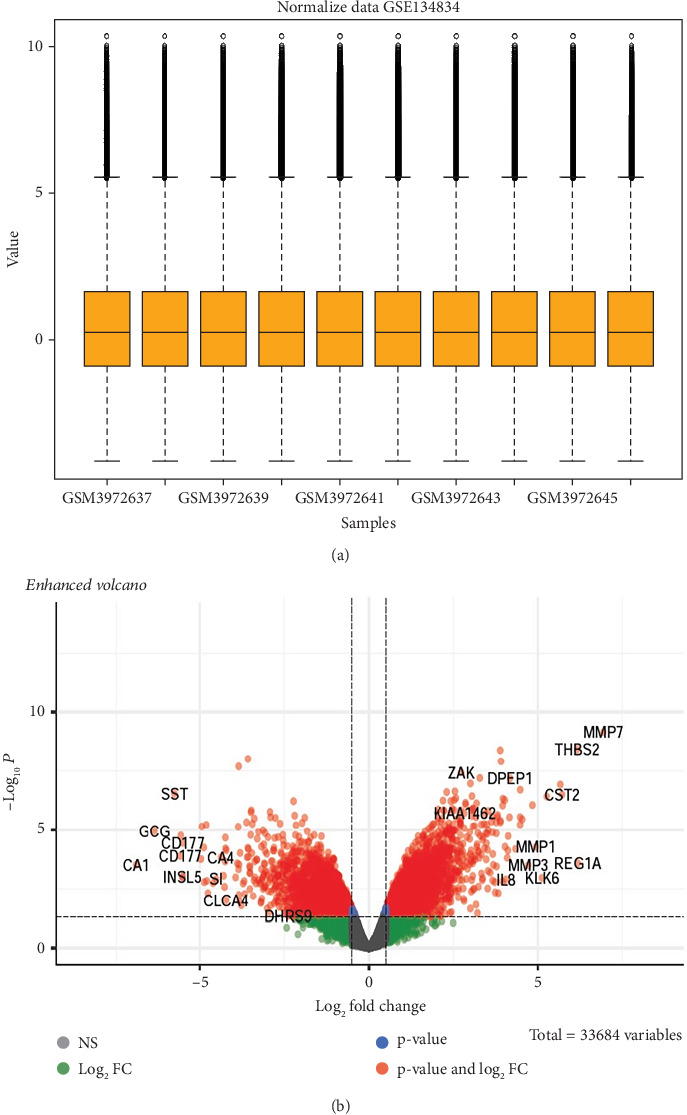
(a) Boxplot of datasets where the normal distribution of GSE134834 microarray datasets is evident; (b) DEG volcano plot of screening for DEGs at *p* < 0.05 and |log (FC)| ≥ 0.5.

GSE134834 was analyzed in R software using the limma package, and the normality of the samples was checked using the normalized quantiles function. The box plot shows that the samples have a normal distribution (Figure 2(a)). GSE analysis revealed 6763 DEGs in the CRC samples against the normal samples based on the defined criteria (*p* < 0.05 and |log (FC)| ≥ 0.5).  Figure 2(b) is a volcano plot of the DEGs. A total of 4725 differentially expressed mRNAs (DEmRNAs) (Supporting Information 1) were identified, and the top DEmRNAs (increased or decreased; *p* < 0.05 and |log (FC)| ≥ 0.5) are listed in Table 3.

**Figure 3:**
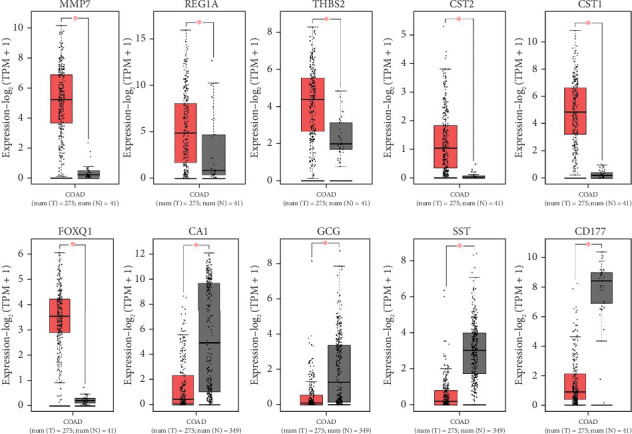
Validation of mRNA expression of top DEmRNA (MMP7, REG1A, THBS2, CST2, CST1, FOXQ1, CA1, GCG, SST, and CD177) in GSE134834 using GEPIA. The graphs are selected from match TCGA normal and GTEx data or match TCGA normal data (*p* < 0.01; red box: tumor samples; black box: normal samples; COAD: colon adenocarcinoma).

The expression of all top genes was significantly different between the normal and CRC tissues according to the GEPIA website, which was similar to our results (Figure 3). A total of 2039 DElncs were identified according to the defined criteria and are provided in detail in Supporting Information 2. Analysis revealed 867 upregulated lncRNAs and 1172 downregulated lncRNAs. The top DElncs (increased and decreased DElnc; *p* < 0.05 and |log (FC)| ≥ 0.5) are listed in Tables 1 and 2, respectively. These dysregulated lncRNAs (with the names mentioned in Tables 1 and 2) were investigated in the literature, and cases that had not been reported for CRC were identified. lncRNAs were searched in LNCipedia and GeneCard databases, and literature were reviewed in Google Scholar for these lncRNAs in CRC. Both increased and decreased lncRNAs without reports of CRC could be investigated as novel cases (Tables 1 and 2).

### 3.2. Construction of WGCNA and Identification of lncRNA Modules

**Figure 4:**
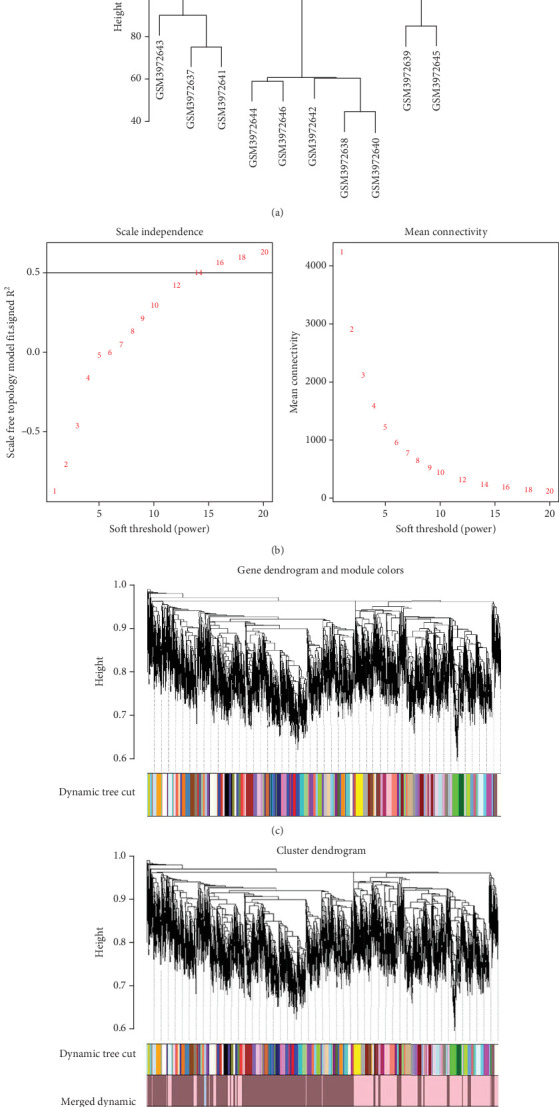
Construction of a weighted gene coexpression network of GSE134834: (a) dendrograms showing hierarchical clustering to find outlier data. No outlier samples in hierarchical clustering can be observed; (b) scale independence network topology analysis for soft-threshold powers and mean connectivity analysis for appropriate soft-threshold power of 14; (c, d) gene clustering dendrogram with dissimilarity based on topological overlap and module colors.

The coexpression network was built for the 6763 DEGs of GSE134834 using WGCNA. After outlier analysis and clustering (Figure 4(a)), the pickSoftThreshold function was performed to calculate the scale independence and mean connectivity of the modules at different power levels. The soft-thresholding power obtained was *β* = 14.  Figure 4(b) shows the graphs of scale independence and mean connectivity. Eighty-three modules were identified and are marked in different colors (Figure 4(c)). After integration, the number of modules was reduced to three according to the similarity criteria. The two color levels below the dendrogram (cut tree dynamic and merged dynamic) indicate the status of modules before and after integration, respectively (Figure 4(d)). Among the modules obtained, the lightpink4 and pink modules coexpressed with dysregulated lncRNAs.

### 3.3. Module Pathway Enrichment

**Figure 5:**
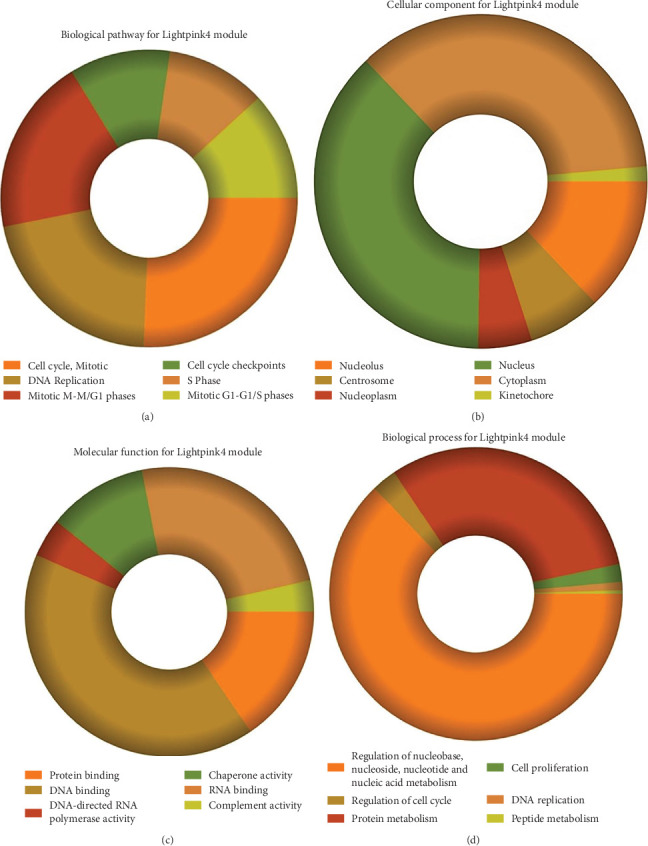
Functional enrichment of lightpink4 module: (a) KEGG pathway enrichment by FunRich of coexpressed lightpink4 module genes; (b–d) GO enrichment of coexpressed lightpink4 module genes; (b) biological process; (c) cellular components; (d) molecular function.

**Figure 6:**
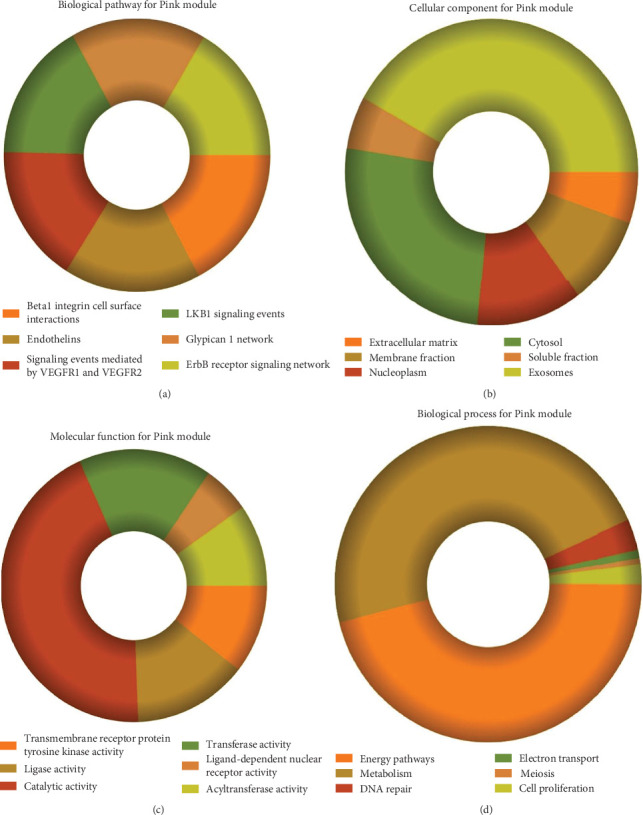
Functional enrichment of pink module: (a) KEGG pathway enrichment by FunRich of coexpressed lightpink4 module genes; (b–d) GO enrichment of coexpressed lightpink4module genes; (b) biological process; (c) cellular components; (d) molecular function.

The role of novel lncRNAs in CRC was predicted through the genes coexpressed in the modules. The MFs, CCs, BPs, and KEGG pathways of the coexpression modules with the identified DElncs were determined and are summarized in Table 4. The lightpink4 module was involved in the regulation of nucleic acid metabolism, DNA replication, cell proliferation, and different phases of the cell cycle (mitotic G1-G1/S and M-M/G1 phases, cell cycle checkpoints, and S phase). The role of the lightpink4 module in cellular pathways is consistent with its role in BPs involving regulation of nucleobase, nucleoside, nucleotide, and nucleic acid metabolism; regulation of cell cycle; protein metabolism; cell proliferation; DNA replication; and peptide metabolism (Figure 5). The pink module genes were enriched in transmembrane receptor protein tyrosine, kinase activity, ligase activity, and catalytic activity. In this module, the genes were mainly located in the extracellular matrix and membrane fraction in *β*1 integrin cell surface interactions, endothelin, VEGFR2 events, LKB1 signaling, the Glypican 1 network, and ErbB receptor pathways. BP for the pink module was enriched mainly in energy pathways and metabolism (Figure 6).

### 3.4. PPI Network and Hub Genes

**Figure 7:**
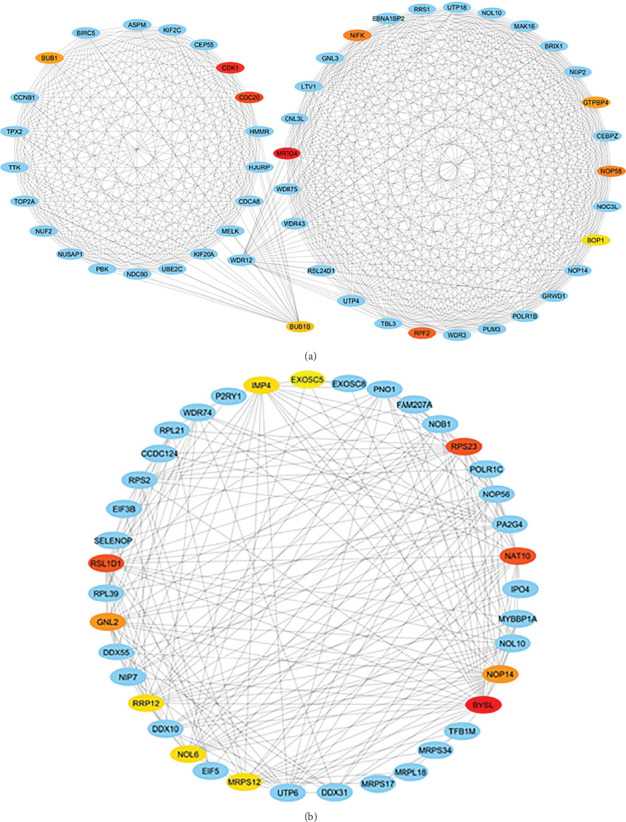
Establishment of PPI network of (a) lightpink4 module and (b) pink modules of GSE134834 analysis. The rectangles and lines represent genes and the interaction of proteins between genes, respectively. The hub gene network has been identified and ranked based on node degree. Between the hub genes, the red and yellow nodes have higher and lower node degrees in the network, respectively. The orange nodes have a medium node degree.

**Figure 8:**
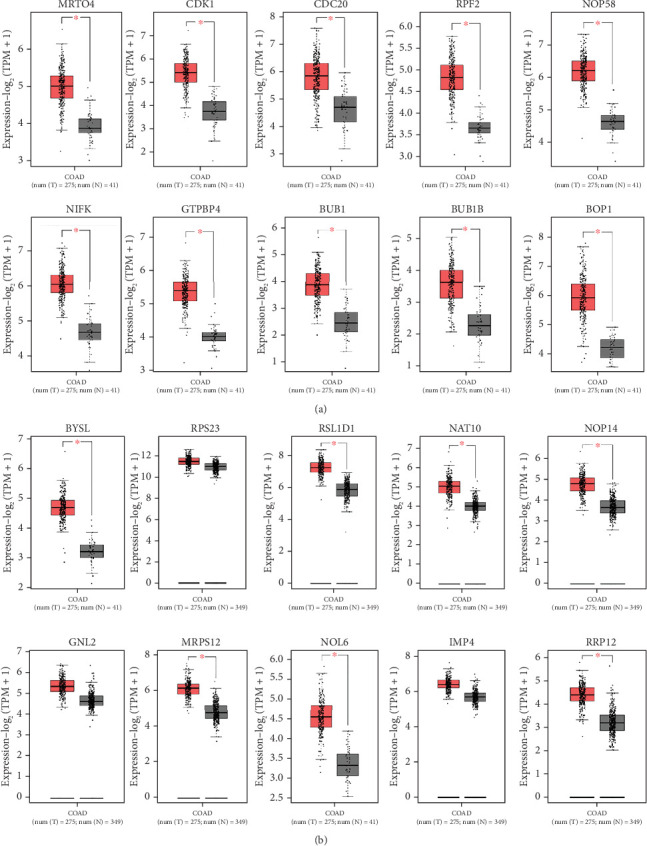
Validation of mRNA expression levels of hub gene in (a) lightpink4 module and (b) pink module for GSE134834 using GEPIA. The graphs are selected from match TCGA normal and GTEx data or match TCGA normal data (*p* < 0.01; red box: tumor samples; gray box: normal sample; COAD: colon adenocarcinoma).

The PPI network was created and visualized using the STRING database and Cytoscape software (Figure 7). The CytoHubba plugin of Cytoscape was used to screen the hub genes based on degree scores in the modules (Table 5). *MRTO4*, *CDK1*, *CDC20*, *RPF2*, *NOP58*, *NIFK*, *GTPBP4*, *BUB1*, *BUB1B*, and *BOP1* were hub genes in the lightpink4 module. Pink module hub genes were *BYSL*, *RPS23* (ribosomal protein S23), *RSL1D1* (ribosomal L1 domain containing 1), *NAT10*, *NOP14*, *GNL2*, *MRPS12*, *NOL6* (nucleolar protein 6), *IMP4*, and *RRP12* (ribosomal RNA processing 12 homolog). Based on our results, all hub genes in the lightpink4 and pink modules were upregulated in CRC. Overexpression of the hub genes was confirmed by the GEPIA website except for the *GNL2*, *PPS23*, and *IMP4* genes (Figure 8).

## 4. Discussion

CRC is the third most common malignancy with a 5-year survival rate. Approximately 90% of patients are diagnosed in the early stages, and around 14% of patients are diagnosed in more advanced stages [58, 59]. Cancer-associated genes are some keys to finding new cases in the early stages or also a way to treat patients to increase the overall survival rates [60]. Here, lncRNAs were introduced as useful biomarkers or accessible molecular therapeutic targets due to their abundance and regulatory functions [61–65]. More importantly, lncRNAs were associated with different stages of CRC disease [66, 67], and several lncRNAs have been identified to play roles in cancer resistance [68]. lncRNAs may activate or repress transcription, modulate transcription enhancers, interfere with RNA polymerase function, remodel chromatin, modify histones, influence DNA methylation, regulate translation, change mRNA stability, and rearrange splicing [69]. Dysregulated lncRNAs in CRC offer significant clinical utility in screening, diagnosis, prognosis, and treatment. Firstly, regions containing lncRNAs may harbor polymorphisms that heighten susceptibility to CRC. Second, lncRNAs are abundant in body fluids and identified as crucial biomarkers for early detection and prognostication of CRC. Third, lncRNAs are recognized as therapeutic targets linked to CRC patients [67, 69].

It was reported that lncRNAs trim many signaling paths associated with CRC, including Wnt/*β*-catenin, EGFR/IGF-IR, KRAS, PI-3-kinase, TGF-*β*, p53, and the epithelial—mesenchymal transition (EMT) program [67]. For example, PVT1, MALAT1, and PCAT-1 downregulations make susceptible CRC cells to fluorouracil by regulating MDR genes and promoting apoptosis. It is also reported that ENST00000547547 enhances fluorouracil sensitivity in CRC by competitively sequestering miR-31/ABCB9 axes [70]. In contrast, overexpression of the lncRNA of GIHCG promotes invasion and chemoresistance in CRC tissues [71]. Increasing interest can be seen among scientists to find competitive networks among lncRNAs/miRNAs/mRNAs involved in CRC pathogenesis during the last decade [72, 73].

**Figure 9:**
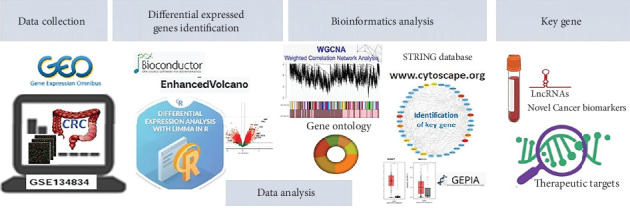
Schematic path represents new biomarkers in CRC using bioinformatics tools and the expression data analysis.

There are studies in various fields with the aim of achieving a correct understanding of cancer and finding the best treatment [74–77]. The use of computers, bioinformatics analysis, and different software in cancer studies with different goals is expanding [78, 79]. Gene expression profiles in high-throughput technologies are rapidly evolving. The CRC gene expression pattern has been documented in several studies with which to explore the expression profiles of miRNA, mRNA, and lncRNA in CRC [80]. The combination of high-throughput technologies such as microarrays with bioinformatic approaches has facilitated comprehensive analysis of changes in miRNA, mRNA, and lncRNA expression in cancer. The goal of the current study has been to identify top lncRNAs and novel lncRNAs as well as top mRNA in GSE134834-related CRC using a systems biology approach to better understand the molecular mechanism of cancer and to predict potential therapeutic target genes in CRC (Figure 9). Dai et al. demonstrated that 4307 lncRNAs were deregulated in CRC [81]. Another study suggested more than 1000 deregulated DElncs between CRC and normal tissues [82].

After GSE134834 analysis, we first identified 6762 DEGs that included DEmRNAs and DElnc in the CRC tissue compared with adjacent normal tissue. mRNA expression analysis was performed for the top identified genes of *MMP7*, *REG1A*, *THBS2*, *CST2*, *CST1*, *FOXQ1*, *CA1*, *GCG*, *SST*, and *CD177* using the GEPIA database to verify their expression patterns in CRC according to our findings. These mRNAs showed maximum expression change in our study and have previously been reported to participate in CRC pathogenesis (Table 3). Previously, *MMP7*, *THBS2*, *CST1*, *CA1*, *GCG*, *SST*, and *CD177* were identified as hub genes based on CRC microarray expression datasets [83]. For example, serum levels of *MMP7* in 112 patients with CRC and 115 healthy controls were significantly higher in CRC patients [84]. Knockdown of *REG Iα* was promoted by apoptosis in HCT116 and LOVO cells and also inhibited the migration and cell cycle of CRC [85]. The in vitro experimental data showed that *THBS2* overexpression promoted CRC cell growth, invasion, and migration [86]. Knockdown of *THBS2* has been shown to suppress Wnt/*β*-catenin signaling activity [86]. The *CST1* upregulation in colon cancer patients and CRC cell lines was confirmed by RT-PCR, immunohistochemistry, and western blot analyses [48]. *CST1* and *let-7d* were involved in the proliferation of cells in CRC. let-7d inhibits the progression of CRC through the CST1/p65 pathway [48]. The circ_0001821 binds to miR-339-3p to regulate *CST1* expression. Silencing circ_0001821 inhibited tumor development in vivo [87]. RT-PCR analysis revealed overexpression of *FOXQ1* in CRC. Overexpression of *FOXQ1* activates the FAK/PI3K/AKT signaling pathway and increases the aggressiveness of CRC [50]. Decreased expression of somatostatin in CRC was revealed by RT-PCR [88]. Downregulation of *SST* is partly attributed to higher methylation levels of *SST* in tumor tissues. Clinical research has indicated that elevated *SST* methylation levels in the serum of CRC patients can predict tumor recurrence and cancer-related mortality [55, 89].

The identified top DElnc role was investigated in the literature for association with the development and pathogenesis of CRC. The literature review represents some other reports with Tables 1 and 2, but the expression information or function was not highlighted. For example, Yang et al. reported upregulation of ENST00000419422.1, ENST00000429700.1, ENST00000602761.1, TCONS_00014191, ENST00000437781.1, ENST00000500112.1, and TCONS_00015168 and downregulation of ENST00000591283.1, uc.217+, ENST00000506514.1, and uc022axe.1 in CRC. They reported that ENST00000456532.1 or LINC02595 is upregulated in tumor tissues and blood samples of patients with CRC and CRC cell lines [24]. Zhang et al. reported ENST00000437781.1 knockdown in esophageal carcinoma cells that inhibited cell proliferation and facilitated cell apoptosis [90]. Knockdown of RP11-132A1.4 (EMS) in A549 and PC9 cells resulted in reduced cell proliferation. Conversely, overexpression of EMS shortened the G1 phase and prolonged the S phase in these cells [83]. Increased expression of ENST00000419422.1 (RP11-132A1.4 or EMSLR) has been documented in cancer in various studies, but its molecular mechanism remains largely unclear. RP11-132A1.4, also known as E2F1 mRNA-stabilizing lncRNA, has been identified as a direct transcriptional target of c-MYC. c-MYC, the major oncogene activated in over 50% of human cancers, plays a significant role in this context [24, 91]. In colony formation experiments, it was found that LINC02418 regulates the proliferation of CRC cells by regulating BCL2 [21]. Overexpression of LINC02257 was confirmed in both CRC tissue and cell lines by RT-PCR [92]. Overexpression of lncRNA colorectal neoplasia differentially expressed (CRNDE) regulates drug resistance in CRC by controlling the miR-181a-5p level and Wnt/*β*-catenin path [93]. LINC02418 was found to regulate BCL2 and thereby control the proliferation of CRC cells. Human colon cancer samples exhibited elevated levels of LINC02418, also referred to as lncRNA p3467, compared to adjacent tissue. LINC02418 interacted physically with miR-34b-5p and influenced the BCL2 signaling path. Silencing LINC02418 decreased CRC cell proliferation, whereas transfection of BCL2 or a miR-34b-5p inhibitor into LINC02418-silenced CRC cells enhanced cell growth significantly [21]. CRNDE targets miR-181a-5p as an inhibitor. CRNDE knockdown and miR-181a-5p overexpression resulted in the suppression of Wnt/*β*-catenin signaling. Here, miR-181a-5p targeted *β*-catenin and TCF4, the key components of the Wnt/*β*-catenin pathway [93].

BBOX1-AS1 is highly expressed in CRC and inhibits apoptosis, increased proliferation, invasion, resistance to radiotherapy, migration, and glycolysis [94]. Studies using quantitative real-time PCR (qRT-PCR) have revealed the overexpression of LINC01605 in CRC tissue specimens and CRC cells. This lncRNA has been found to target miR-3960/SOX11 to promote migration, proliferation, and invasion of CRC cells [29, 30]. These documents confirm the results of microarray analyses in our study. The DElncs that did not report in CRC can be proposed as novel lncRNAs and further investigated. To better understand the function of the novel DElncs, gene modules were constructed with WGCNA and GO to obtain the hub genes of their coexpression modules.

A module is a group of strongly coexpressed genes that appear to be functionally related and have a similar function or are involved in a common BP [64, 95]. The gene modules for top downregulated and upregulated DELnc are shown in Table 5. For example, lncRNA p3467, lncRNA p15938, and lncRNA p30155 with increased expression and lncRNA p8237 with decreased expression belong to the lightpink4 module, and upregulated lncRNA p15430 and downregulated DElnc (lncRNA p15430, lncRNA p4715, lncRNA p6343, and lncRNA p16717) belong to the pink module. GO and pathway analysis revealed that lightpink4 genes participate in essential BPs such as cell proliferation, cell cycle, and protein metabolism. Irregularity of the cell cycle is the basis of aberrant cell proliferation, which is a characteristic of cancer. Cancer genetics has shown that mutations in growth signaling networks are important factors in cancer [96, 97]. Pink module genes were mainly involved in confirmed paths for CRC development, including *β*1-integrin cell surface interaction [98], endothelin [98–100], VEGFR1 and VEGFR2 [101], LKB1 signaling events [102], Glypican 1 network [103], and ErbB receptor [104]. This suggests that gene expression changes in these pathways may play a critical function in CRC pathogenesis. The pink module was enriched mainly in energy pathways and metabolism. For unlimited proliferation and rapid growth, cancer cells increase their biosynthetic and metabolic activities and ribosome biogenesis. Ribosome biogenesis is an energy-consuming process that begins in the nucleus. Changes in the expression or mutations in the ribosomal protein gene play an important role in the development of cancer [105–107]. Using the information from the GO analysis of the modules, we predicted that these lncRNAs may play a functional role in CRC. Further experimental studies should be designed, however, to reveal the exact role of novel lncRNA.

Bioinformatics analysis on the hub genes of every module demonstrated that they participate in a similar BP. These hub genes interact with many other genes and play a critical function in developing initiation, proliferation, survival, migration, and invasion in CRC. The analysis showed that all hub genes within the lightpink4 module were upregulated in CRC. Verification of hub gene overexpression in CRC was conducted using the GEPIA website within the lightpink4 module (Figure 7). Hub genes such as *CDK1*, *CDC20*, *BUB1*, and *BUB1B* are primarily involved in cell cycle phases and cell proliferation. The risk of CRC is associated with defects in DNA replication. As with other cancers, they appear to involve the upregulation of DNA replication licensing proteins [20]. The upregulation of DNA replication licensing proteins appears to occur in the early stages of CRC. CDK1 is involved in cell cycle regulation (G2-M transition and regulator of G1 progression and G1-S transition), which is overexpressed in hepatocellular carcinoma (HCC) and CRC tissues [108]. Knockdown of CDC20 reduces CRC cell proliferation, suggesting its potential as a prognostic biomarker for patients. CDK1 promotes cell growth through JAK1 phosphorylation and the JAK/STAT3 path activation [109, 110].*BUB1*, a serine/threonine kinase, is a checkpoint involved in chromosome segregation in mitosis [111]. *BUB1B* is upregulated in prostate cancer and CRC tissue and increases cell proliferation [112, 113]. On the other hand, overexpression of *BOP1* increases the migration and invasion capabilities of the cells. *BOP1* is an important target for the Wnt/*β*-catenin path, Wnt/*β*-catenin-induced EMT in CRC cells [114].

Genes related to ribosome biogenesis and peptide metabolism, such as *MRTO4*, *RPF2*, *NOP58*, and *GTPBP4*, are other hub genes. Data enrichment analysis has shown that coexpressed genes in lightpink4 are involved in ribosome biosynthesis. Protein synthesis is necessary for the unlimited proliferation of cancer cells. Ribosome assembly factor *MRTO4* is a *trans*-acting factor involved in ribosome biogenesis. Previous studies based on system biology have shown the potential of *MRTO4* as a prognostic marker in CRC, even though *MRTO4* mechanisms remain unclear [115, 116]. *RPF2* is a factor involved in ribosome biosynthesis that is overexpressed in CRC tissues and cells. *RPF2* interacts directly with *CARM1*, activates the AKT/GSK-3*β* path, and enhances the migratory and invasive abilities of the cells both in vitro and in vivo [117, 118]. AKT/GSK-3*β* is an important mechanism to control tumor invasion and metastasis [119].

NOP58 is significantly overexpressed in CRC and hepatocellular carcinoma [120, 121]. In the intestinal epithelial cells, *ZFAS1*, *NOP58*, and snoRNAs are normally expressed, contributing to proper rRNA 2⁣′-O-methylation and translation. However, in CRC, *ZFAS1* and its binding partner *NOP58* are overexpressed. *ZFAS1* enhances the formation of small nucleolar ribonucleoprotein (snoRNP) by synergistically recruiting snoRNAs, particularly SNORD12C and SNORD78, together with *NOP58*. This leads to an elevation in rRNA 2⁣′-O-methylation levels, which ultimately alters translational activity and precision of downstream target genes, thereby promoting CRC proliferation [121]. NIFK promotes lung cancer, heHCC, and CRC [122, 123]. *GTPBP4* is a GTPase that plays a crucial role in 60S ribosome biogenesis and is responsible for tumor metastasis in CRC by disrupting the actin cytoskeleton, which is mediated by the reduced RhoA activity [124, 125]. *BYSL* is involved in ribosomal biogenesis that is overexpressed in HCC specimens [126].

Overexpression of the hub genes in the pink module in CRC was confirmed using the GEPIA website, with the exception of the *GNL2*, *PPS23*, and *IMP4* genes (Figure 7). The increased expression of hub genes *GNL2*, *RPS23*, and *IMP4* was not significant in GEPIA, but other studies have reported the increased expression of *GNL2*, *RPS23*, and *IMP4* in cancer. Expression of the *RPS23* gene increased in both early and advanced stages of CRC [127, 128]. There are few reports on the specific biological function of *IMP4* and *GNL2* (called NGP-1) in CRC. Dong et al. reported an increase in *GNL2* expression in liver hepatocellular carcinoma (LIHC), correlating with enhanced cell proliferation, invasion, and migration capabilities [129]. However, there is limited literature on the specific biological roles of *IMP4* and *GNL2* in CRC. Some demonstrated that *NGP-1* stimulates cell cycle progression, potentially by modulating the balance between p21 and the cyclin D1-CDK4 complex in a manner dependent on p53 [130]. Knockdown of *GNL2* reduced glioma cell migration and invasion. *GNL2* enhances the synthesis of ribosomal protein L11 (*RPL11*), thereby promoting glioma development [131]. The role of *IMP4*, known as *IMP U3*, remained underexplored in CRC. *IMP4*, *BRMS2*, and *MPP10* are implicated in stabilizing or facilitating base-pairing interactions between U3 small nucleolar RNA (snoRNA) and pre-rRNA, essential for U3 small nucleolar ribonucleoprotein complex (U3 snoRNP)–mediated rRNA maturation [132, 133]. *IMP4* plays a crucial role in enhancing cell proliferation and protein translation rates in CRC by promoting increased translation ability. Targeting *IMP4* could potentially inhibit CRC progression. Literature reported increased *IMP4* expression in human lung adenocarcinoma (LUAD) tissues, where it promoted disease progression through activation of the ERK pathway. *IMP4* is also involved in the maturation of 18S rRNA [134].

MRPS12 (mitochondrial ribosomal small subunit protein) was significantly upregulated in chemotherapy-treated tumors compared to untreated tumors [135]. Clinical samples and CRC cells often exhibited overexpression of *RRP12*. Knockdown of *RRP12* inhibits CRC cell invasion and migration since *RRP12* mediates the EMT process via *ZEB1*. In vivo studies have shown that *RRP12* knockdown reverses CRC cell metastasis [136]. *BYSL* is involved in ribosomal biogenesis and overexpresses in HCC specimens [126]. It is reported that *NAT10* through the NAT10/KIF23/GSK-3*β*/*β*-catenin path accelerates CRC progression. *NAT10* potentially facilitates CRC progression through ac4C modification. By binding to the 3⁣′-UTR (untranslated region) of KIF23 mRNA and enhancing mRNA ac4c modification. *NAT10* stabilizes KIF23 mRNA, and this stabilization increases KIF23 protein levels, subsequently activating the Wnt/*β*-catenin path and promoting CRC progression by enhancing *β*-catenin translocation into the nucleus. Depletion of *NAT10* has been associated with significant reductions in NUP210 and integrin *β*1 protein levels. Loss of *NAT10* decreases lung metastases in genetically modified and allograft mouse models of breast cancer [137–139].

*β*-catenin accumulation is a common factor in CRC, and its uncontrolled activation influences the Wnt/GSK-3*β*/*β*-catenin pathway and leads to tumorigenesis. Zhu et al. reported that *NOP14* increases the proliferation and metastasis of CRC cells through the NRIP1/GSK-3*β*/*β*-catenin path. *NOP14* overexpression has been linked to increased expression of *NRIP1* and phosphorylation of *GSK-3β*. This cascade results in the upregulation of *β*-catenin [140]. *RSL1D1* also suppresses autophagy and facilitates the growth and metastasis of the cells [141]. Liu et al. showed that the overexpression of *RSL1D1* in CRC patients regulates autophagy and tumor progression through the RSL1D1/RAN/STAT3 pathway. *RSL1D1* inhibits RAN deacetylation by competitive binding to Sirt7, reduces nuclear STAT3 accumulation, promotes cell proliferation and invasion, and suppresses autophagy [142]. *NOL6* is involved in ribosome biogenesis through interaction with pre-rRNA transcripts and accelerates the proliferation and migration in cancers [143, 144].

Finally, a lot of hub genes may be involved in ribosome biogenesis and assembly. Accurate ribosome biogenesis is crucial for protein biosynthesis, cell division, proliferation, and apoptosis. Increased protein synthesis is crucial for rapid cancer cell divisions [145]. Recent data suggest that cancer cells may possess a distinct category of ribosomes known as oncoribosomes. These oncoribosomes are believed to promote an oncogenic translation program, disrupt cellular processes, and induce metabolic reprogramming. Ribosomopathy arises from any mutations affecting the ribosomal proteins, rRNA processing, and factors involved in ribosome assembly [105]. Our study introduces some lncRNAs that have not been investigated in CRC. Examining the function of some known cases in CRC shows their relationship with cell proliferation and the Wnt/*β*-catenin path. Some previous studies proved the accuracy of some hub genes that we identified and suggested in CRC. Our study's limitation lies in the lack of experimental validation of the outcomes derived from bioinformatics techniques. Therefore, additional experimental research and clinical tissue verification are necessary to confirm our findings. It is hoped that the present study will contribute to the identification of biomarkers and therapeutic targets for CRC.

## 5. Conclusion

Considering the abundance of lncRNAs in the human genome and their role in various cells, it is likely that their expression disorder plays a key role in cancer pathogenesis. In the present study, GSE134834 datasets obtained from the GEO database and bioinformatics analysis led to the suggestion of a number of novel dysregulated lncRNAs in CRC that have not been reported. In order to identify as many of these lncRNAs as possible, WGCNA explored the coexpression module genes of these lncRNAs and subjected them to biological pathway analysis. We conducted an analysis of dysregulated lncRNA coexpression genes to identify paths that potentially correlated with CRC. Through this analysis, hub genes within modules of the PPI network were identified, which are positively associated with CRC. The next phase of research involved employing computational algorithms to predict substances that could target these hub genes. These hub genes predominantly participate in ribosome biogenesis and cell cycle regulation. Mutations affecting ribosomal proteins, rRNA processing, and ribosome assembly factors have been linked to an increased cancer risk. Recent studies have highlighted ribosome-targeted therapies as promising treatments for cancer patients. The dysregulation of the cell cycle underpins abnormal cell proliferation, a hallmark feature of cancer. Our study systematically and continuously examined the results obtained from the transcriptome analysis of CRC tissue compared to healthy tissue and provided information about lncRNAs and key genes that can facilitate the understanding of the molecular mechanisms of CRC. Experimental investigation is required to validate the findings of the present study. Our results suggest some new lncRNAs and key genes and provide useful information for researchers to better understand the molecular mechanisms and identify new diagnostic biomarkers and therapeutic targets for CRC.

## Figures and Tables

**Table 1:** Top upregulated lncRNAs in the analysis of GSE134834 microarray related to CRC. CRC: colorectal cancer, FC: fold change, lncRNA: long noncoding RNA.

**Gene symbol**	**lncRNA ID**	**Ensemble gene ID**	**Official name**	**Log (FC)**	**p** ** value**	**Adjusted ** **p** ** value**	**Function in CRC**
lncRNA p3467	ENST00000561864.1	ENSG00000261650.1	RP11-474D1.4 (LINC02418)	4.480166	2.85e − 06	0.001741	High expression of lncRNA p3467 promoted cancer progression in colon cancer cells [21, 22]
lncRNA p15938	ENST00000500112.1	ENSG00000247844.1	RP11-255B23.3	4.028627	5.46e − 07	0.00082	—
lncRNA p30155	XR_108886.3	LOC100507056	IDLOC100507056	3.962785	9.27e − 07	0.001041	—
lncRNA p15421	ENST00000419422.1	ENSG00000232445.1	RP11-132A1.4 (LNCPRESS1)	3.776749	4.71e − 06	0.002226	RP11-132A1.4 is upregulated in tissue from colon cancer [23]
lncRNA p39026_v4	ENST00000437781.1	ENSG00000206195.6	AP000525.9	3.665108	1.30e − 05	0.003626	High expression in CRC [24]
lncRNA p15430	ENST00000602761.1	ENSG00000228742.5	RP5-884M6.1	3.471953	1.41e − 05	0.003794	High expression in colon adenocarcinoma [24, 25]
lncRNA p34144_v4	ENST00000447298.1	ENSG00000130600.10	H19-014	3.388798	2.04e − 05	0.004494	—
LOC340340	Uncharacterized LOC340340	—	—	3.239423	8.20e − 05	0.010084	—
lncRNA p29027	ENST00000414790.1	ENSG00000130600.10	H19-001	3.230258	1.37e − 05	0.003769	—
lncRNA p34360_v4	ENST00000427798.1	ENSG00000225210.5	AL589743.1 (DUXAP9/LINC01296)	3.207409	4.19e − 05	0.007123	LINC01296 knockdown inhibited colon cancer cell proliferation, migration, and invasion in vivo [26]
lncRNA p17009	ENST00000456532.1	ENSG00000231566.1	RP5-1158E12.3	3.193662	7.45e − 07	0.000929	High expression of LINC02595 promoted tumor progression in CRC [24]
lncRNA p30261	uc004dgk.1	AK098783		3.089308	1.29e − 06	0.001291	LncRNA-AK098783 expression level was frequently higher in CRC and RT-PCR [27]
lncRNA p24045	TCONS_00015168	XLOC_007214	linc-FAM84B-8	3.084878	1.22e − 06	0.001291	—
lncRNA p23831	TCONS_00014191	XLOC_006200	linc-PIK3CG-1	3.08157	0.000903	0.029689	—
lncRNA p21811	TCONS_00029614	XLOC_014268	linc-PPARA-1	3.058199	3.71e − 07	0.00072	—
lncRNA p9376	ENST00000424322.1	ENSG00000227400.2	AC012501.2	3.050053	0.001343	0.035616	—
lncRNA p16370	ENST00000521586.1	ENSG00000253929.1	RP11382A18.2	2.990761	2.45e − 05	0.004912	Colon cancer risk SNPs [28]
lncRNA p21975	TCONS_00006729	XLOC_003364	linc-BCL6-5	2.926155	0.000445	0.0216	—
lncRNA p2786	ENST00000544553.1	ENSG00000256940.1	RP11783K16.5 (PPP1R14B-AS1)	2.904332	2.08e − 05	0.004494	High expression in CRC [24]
lncRNA p35380_v4	ENST00000521989.2	ENSG00000253414.2	RP11150O12.6	2.900774	0.000235	0.01617	Upregulated in CRC cells [29, 30]
lncRNA p956	ENST00000432653.1	ENSG00000234578.1	RP4-784A16.1	2.865494	7.09e − 07	0.000918	—
lncRNA p10603	ENST00000445003.1	ENSG00000224397.1	RP11-290F20.3	2.788621	0.000184	0.014888	High expression of LINC01272, promoted EMT, and metastasis by regulating miR-876/ITGB2 [31]

**Table 2:** Detail of top downregulated lncRNAs in the analysis of GSE134834 microarray related to CRC. CRC: colorectal cancer, FC: fold change, lncRNA: long noncoding RNA.

**Gene symbol**	**lncRNA ID**	**Ensemble gene ID**	**Official name**	**Log (FC)**	**p** ** value**	**Adjusted ** **p** ** value**	**Function in CRC**
lncRNA p33911	uc.217+	uc.217+	uc.217+	−4.93989	7.23e − 06	0.002648	Decreased in CRC tissue [24]
lncRNA p33840	uc022axe.1	FW340046	auc022axe.1	−3.893	0.000264	0.017247	Decreased in CRC tissue [24]
lncRNA p4715	ENST00000497872.2	ENSG00000253701.1	AL928768.3	−3.8548	0.000469	0.02198	Decreased in CRC tissue [32]
lncRNA p12653	ENST00000506514.1	ENSG00000249453.1	RP13-497K6.1	−3.58082	5.85e − 05	0.0087	Decreased in CRC tissue [24]
lncRNA p16717	ENST00000422420.1	ENSG00000240498.2	CDKN2B-AS1	−3.52951	0.000343	0.019	Decreased in CRC. CDKN2B-AS induced cell apoptosis and suppressed EMT. CDKN2B-AS1 regulates cell growth and survival by targeting DUSP1 [33]
lncRNA p28599	ASO1844	ASO1844	Accession=AK097806	−3.49506	1.67e − 05	0.004132	—
lncRNA p22463	TCONS_00008010	XLOC_003474	linc-SOD3-2	−3.45933	9.77e − 05	0.011	—
lncRNA p20354	TCONS_00025506	XLOC_012317	linc-TNRC6C-3	−3.37675	0.000218	0.015	—
lncRNA p20353	TCONS_00025200	XLOC_012317	linc-TNRC6C-3	−3.31869	0.00018	0.014	—
lncRNA p27381	uc.70-	uc.70-	uc.70-	−3.29996	2.42e − 05	0.0041	—
lncRNA p10205	ENST00000416200.1	ENSG00000225953.2	SATB2-AS1	−3.26443	1.23e − 05	0.003	Decreased in CRC tissue, suppressed CRC aggressiveness by inhibiting SATB2-dependent snail transcription and EMT [34]
lncRNA p25200	NR_026542.1	C21orf88	C21 orf88(B3GALT5-AS1)	−3.17449	0.000467	0.021	Decreased in CRC tissue, the suppressive role of B3GALT5-AS1/miR-203/EMT regulation axis was in colon cancer liver metastasis [35, 36]
lncRNA p16220	ENST00000526382.1	ENSG00000255130.1	RP11-396J6.1-001	−3.12744	1.03e − 05	0.003	—
lncRNA p20355	TCONS_00025507	XLOC_012317	linc-TNRC6C-3	−3.08187	0.000289	0.017	—
lncRNA p25346	NR_024418.1	LOC389332	LOC389332	−3.06741	8.12e − 05	0.01	Decreased in CRC tissue. LOC389332 exhibited longer overall survival time than patients with low expression levels of these genes [37]
lncRNA p11319	ENST00000425296.1	ENSG00000224822.1	THRB-IT1	−2.98159	2.08e − 05	0.0044	—
lncRNA p12499	ENST00000508106.1	ENSG00000234828.3	RP11-526A4.1	−2.81665	0.000444	0.02	—
lncRNA RNA143457|tRNA_374_71	RNA143457|tRNA_374_71			−2.8047	7.57e − 05	0.0097	—
lncRNA p21571	TCONS_00028228	XLOC_013571	linc-PFDN4-2	−2.65782	0.000291	0.0179	—
lncRNA p7182	ENST00000577761.1	ENSG00000266878.1	RP11-1090M7.2	−2.46434	0.000319	0.0183	—
lncRNA p42656_v4	NR_104064.1	None		−2.43954	0.00049	0.0224	—

**Table 3:** Top DEmRNA in CRC based on analyses of the GSE134834 dataset. FC: fold change, DEmRNA: differentially expressed mRNA, CRC: colorectal cancer.

**Gene symbol**	**Log FC**	**p** ** value**	**Adjusted ** **p** ** value**	**Expression and function in CRC in literature**	**References**
*MMP7*	6.941794	7.06e − 10	2.38e − 05	Upregulated: Potential prognostic marker in CRC	[41, 42]
*REG1A*	6.181124	0.000264	0.017247	Upregulated: Potential prognostic marker in CRC	[43, 44]
*THBS2*	6.164384	3.78e − 09	4.66e − 05	Upregulated: Potential prognostic marker in CRC	[45]
*CST2*	5.719364	3.17e − 07	0.000667	Upregulated: Potential prognostic marker in CRC	[46]
*CST1*	5.669326	1.20e − 07	0.000367	Upregulated in CRC tissues	[47–49]
*FOXQ1*	5.266674	3.85e − 07	0.00072	Upregulated: Potential prognostic biomarker	[50–52]
*CA1*	−6.90487	0.000302	0.018054	Downregulated: Patients with lower CA1 expression levels had a worse prognosis	[53]
*GCG*	−6.34476	1.13e − 05	0.003345	Downregulated: Had a major role in suppressing the development of CRC	[52, 54]
SST	−5.74713	2.93e − 07	0.000659	Downregulated in CRC: Promising prognostic biomarkers	[52, 55]
*CD177*	−5.57313	0.00013	0.012561	Downregulated in CRC tissue	[56, 57]

**Table 4:** Lightpink4 and pink modules enrichment results of analysis of GSE134834 microarray for CRC.

**Lightpink4 module gene ontology**		**Pink module gene ontology**	
Biological pathway	*p* value	Biological pathway	*p* value
Cell cycle, mitotic	1.51e − 33	*β*1 integrin cell surface interactions	7.32e − 05
DNA replication	5.9e − 28	Endothelin	0.000101
Mitotic M-M/G1 phases	6.92e − 25	Signaling events mediated by VEGFR1 and VEGFR2	0.000148
Cell cycle checkpoints	1.01e − 18	LKB1 signaling events	0.000157
S phase	2.63e − 17	Glypican 1 network	0.000166
Mitotic G1-G1/S phases	6.9e − 17	ErbB receptor signaling network	0.000176
Molecular function	*p* value	Molecular function	*p* value
Protein binding	1.68e − 05	Transmembrane receptor protein tyrosine kinase activity	0.000171
DNA binding	7.16e − 05	Ligase activity	0.003422
DNA-directed RNA polymerase activity	8.2e − 05	Catalytic activity	0.010486
Chaperone activity	0.000109	Transferase activity	0.010898
RNA binding	0.000333	Ligand-dependent nuclear receptor activity	0.01265
Complement activity	0.001271	Acyltransferase activity	0.013939
Cellular component	*p* value	Cellular component	*p* value
Nucleolus	6.77e − 36	Extracellular matrix	5.92e − 06
Centrosome	4e − 22	Membrane fraction	0.001116
Nucleoplasm	3.55e − 19	Nucleoplasm	0.00122
Nucleus	2.99e − 16	Cytosol	0.001908
Cytoplasm	1.03*e* − 11	Soluble fraction	0.001939
Kinetochore	3.88e − 10	Exosomes	0.002625
Biological process	*p* value	Biological process	*p* value
Regulation of nucleobase, nucleoside, nucleotide, and nucleic acid metabolism	6.24e − 05	Energy pathways	0.000194
Regulation of cell cycle	0.000108	Metabolism	0.000205
Protein metabolism	0.000325	DNA repair	0.003073
Cell proliferation	0.000533	Electron transport	0.006762
DNA replication	0.002767	Meiosis	0.015513
Peptide metabolism	0.0068	Cell proliferation	0.022418

**Table 5:** Top dysregulated lncRNA coexpression modules and module hub genes in the analysis of GSE134834 microarray related to CRC.

**Modules**	**DElnc**		**PPI networks**	**Hub genes**
	**Upregulated lncRNAs**	**Downregulated lncRNAs**		
Lightpink4	p3467, p15938, p30155, p15421, p39026_v4, p34144_v4, p29027, p34360_v4, p17009, p30261, p24045, p21811, p9376, p16370, p21975, p2786, p10603, p44527_v4, p28920, LOC100653147, p2227, p18281, p10499, p14903, p21076, p21710, and p30340	17436, p42655_v4, p26713, p40499_v4, p42344_v4, p4428, p4443, p33911, RNA143457|tRNA_374_71, p42656_v4, p42366_v4, and p2983	805 nodes and 4651 edges	MRTO4, CDK1, CDC20, RPF2, NOP58, NIFK, GTPBP4, BUB1, BUB1B, and BOP1
Pink	p15430, p35380_v4, p956, p16534, p23831, and LOC340340	p33840, p4715, p12653, p16717, p28599, p22463, p20354, p20353, p27381, p10205, p25200, p16220, p20355, p25346, p11319, p12499, p21571, p7182, p9438, p12621, and p12806	713 nodes and 1656 edges	BYSL, RPS23, RSL1D1, NAT10, NOP14, GNL2, MRPS12, NOL6, IMP4, and RRP12

## Data Availability

All databases (including NCBI GEO and Venn diagram maker) are freely available on the web. The raw data of this study are obtained from the GEO database (available at https://www.ncbi.nlm.nih.gov/geo/).

## Supporting Information

Supporting Information 1 GSE134834 was analyzed in R software using the limma package, and DEmRNAs were identified according to the defined criteria (*p* < 0.05 and |log (FC)| ≥ 0.5).

Supporting Information 2 GSE134834 was analyzed in R software using the limma package, and DElncs were identified according to the defined criteria (*p* < 0.05 and |log (FC)| ≥ 0.5).
