# Supplementary material for: Identification of Novel lncRNAs Related to Colorectal Cancer Through Bioinformatics Analysis
Source: Biomed Res Int. 2025 Jan 29;2025:5538575. doi: 10.1155/bmri/5538575 (PMC11824705; doi:10.1155/bmri/5538575)
Supplement: Supporting Information 1 — GSE134834 was analyzed in R software using the limma package, and DEmRNAs were identified according to the defined criteria (p < 0.05 and |log (FC)| ≥ 0.5). [file 5538575.f1.pdf]

Identification of novel lncRNAs related to colorectal cancer through bioinformatic analysis

GSE134834 was analyzed in R software using the Limma package

GeneSymb	logFC	P.Value	adj.P.Val
MMP7	6.941794	7.06E-10	2.38E-05
REG1A	6.181124	0.000264	0.017247
THBS2	6.164384	3.78E-09	4.66E-05
CST2	5.719364	3.17E-07	0.000667
CST1	5.669326	1.20E-07	0.000367
FOXQ1	5.266674	3.85E-07	0.00072
KLK6	5.131079	0.001089	0.03228
MMP1	4.944422	5.41E-05	0.008434
KRT23	4.84438	8.87E-07	0.00103
MMP3	4.720741	0.00032	0.018382
CDH3	4.542981	3.54E-06	0.001866
ADAMTS12	4.484138	1.99E-07	0.000558
COL12A1	4.480451	5.95E-06	0.002331
CXCL1	4.346956	6.50E-05	0.009164
DPEP1	4.181172	6.56E-08	0.000245
FAP	4.167064	2.91E-06	0.001741
CXCL11	4.128296	1.86E-05	0.00433
TUBB3	4.119203	7.60E-05	0.009702
CHI3L1	4.100977	0.000155	0.013802
IL8	4.05858	0.001276	0.034854
KRT23	4.035505	5.26E-05	0.008316
ACAN	3.970636	4.77E-07	0.000804
GAL	3.958769	0.001537	0.0383
NKD1	3.916005	1.24E-08	8.33E-05
KIAA1199	3.902157	4.08E-06	0.002094
FABP6	3.897242	4.74E-06	0.002226
COL8A1	3.893397	4.15E-09	4.66E-05
TACSTD2	3.836468	0.004826	0.064951
CLDN1	3.81432	2.31E-06	0.001623
COL12A1	3.814123	2.85E-06	0.001741
TNFRSF6B	3.782288	0.000359	0.019393
ALDOB	3.777913	1.43E-05	0.003823
TRIM29	3.743993	0.004518	0.063098
S100A8	3.737005	0.001642	0.039533
S100A9	3.689208	0.000308	0.01814
TNFRSF12A	3.672233	0.000107	0.011394
CXCL3	3.668829	0.001403	0.036519
SLC6A6	3.655359	2.67E-06	0.001741
LRP8	3.635503	4.50E-06	0.002197
SPP1	3.598229	0.000652	0.025813
PPBP	3.539599	1.57E-06	0.001299
THBS2	3.536767	1.01E-05	0.003269

S100A2	3.53266	0.000551	0.024022
SAA1	3.521415	0.003059	0.052731
TESC	3.516808	7.07E-05	0.009523
FNDC1	3.510025	1.48E-05	0.003935
ALDOB	3.496041	5.86E-06	0.002331
CXCL1	3.485878	3.81E-05	0.006678
COL1A1	3.479438	0.00018	0.014718
CGREF1	3.436338	1.96E-06	0.001403
SLCO4A1	3.425849	6.03E-05	0.008825
CTHRC1	3.413499	5.55E-06	0.002331
SP5	3.372985	0.001118	0.032657
PCSK9	3.369661	2.69E-05	0.005366
IL11	3.361297	0.001352	0.03575
APOLD1	3.338952	0.000112	0.011505
IL1B	3.337006	0.000714	0.026741
VSNL1	3.331868	5.95E-06	0.002331
SLC7A5	3.303012	1.81E-05	0.004262
EPHX4	3.28959	6.35E-08	0.000245
ENST0000C	3.274489	0.000156	0.013802
PDX1	3.254988	8.49E-05	0.010317
CXCL2	3.247028	0.002579	0.048754
MUC5AC	3.219627	0.034821	0.186065
na	3.198985	5.36E-05	0.008434
SULF1	3.178569	2.87E-06	0.001741
CA9	3.176626	0.015136	0.118128
FCGR3A	3.152607	0.00018	0.014718
AREG	3.143501	0.000127	0.01239
FLJ25917	3.13744	6.89E-06	0.002577
SERPINB5	3.122622	0.003109	0.053118
C10orf81	3.091636	0.000203	0.015344
CXCL10	3.089318	0.00336	0.055387
PAQR4	3.087003	7.07E-06	0.002616
AREG	3.076297	0.000145	0.013426
NKD2	3.075018	0.000263	0.017247
ARNTL2	3.060628	1.42E-06	0.001291
FLJ25917	3.055198	2.50E-06	0.001684
KIAA1199	3.033674	7.57E-05	0.009702
CYR61	3.030699	0.000219	0.015622
TRIB3	3.01044	1.08E-07	0.000363
NMU	3.010366	0.022274	0.145825
CXCL2	3.002167	0.000108	0.011456
PDPN	2.993353	1.23E-05	0.003513
CKMT2	2.985687	0.000293	0.017972
TNFAIP6	2.928525	0.00014	0.013188
STC2	2.888429	0.001207	0.034085
PHLDA1	2.873423	8.41E-07	0.001011
KIAA1462	2.829058	1.94E-06	0.001403

EGFL6	2.828075	8.55E-06	0.00291
NPSR1	2.823005	0.010621	0.098122
UBD	2.821648	0.000353	0.01927
CXCL10	2.811608	0.008608	0.087918
PITX2	2.798591	0.003033	0.052573
TRIP13	2.773564	0.000114	0.011696
C2CD4A	2.765539	0.001368	0.035932
C10orf81	2.76075	0.000354	0.01927
ZAK	2.740799	3.71E-08	0.000179
MTHFD1L	2.735064	2.98E-06	0.001741
CLDN2	2.733828	5.73E-05	0.008692
SFRP4	2.724614	1.48E-06	0.001291
TGFB2	2.721035	5.34E-06	0.002331
SNORA71C	2.718934	0.000449	0.021681
APOLD1	2.716264	9.11E-05	0.010683
ADAMTS5	2.716003	1.74E-05	0.0042
HILPDA	2.712695	5.95E-06	0.002331
ADAMTS2	2.711552	4.13E-06	0.002094
TNS4	2.709255	4.57E-05	0.00762
PRRX1	2.701107	0.000233	0.01617
COL5A2	2.693795	0.00081	0.028585
SLC6A6	2.68102	8.11E-06	0.002818
ATP11A	2.662707	0.000565	0.024243
APOBEC1	2.658672	0.000215	0.015576
COL10A1	2.655457	0.002895	0.051643
IL1RN	2.653292	0.007433	0.081187
SLC7A11	2.651096	0.003773	0.058594
GRIP2	2.637134	0.00018	0.014718
ANLN	2.636819	0.00011	0.011458
na	2.627958	3.04E-05	0.005816
NFE2L3	2.620864	8.10E-05	0.010024
ASB9	2.620302	9.30E-05	0.010731
KIF4A	2.616755	3.37E-06	0.001802
UBE2C	2.609777	0.000146	0.013433
PSAT1	2.599291	3.01E-05	0.005787
ENST00000	2.584087	4.53E-07	0.000803
ALDH4A1	2.581063	0.000556	0.024133
GZMB	2.570103	0.003931	0.059487
CDH6	2.56444	1.07E-05	0.003269
BRCA2	2.533204	0.000272	0.017383
C9orf116	2.53122	7.14E-05	0.009538
PLAU	2.53048	5.43E-07	0.00082
SQLE	2.526127	0.001158	0.033343
LARP6	2.524224	0.000963	0.030495
KCNJ15	2.523074	0.000455	0.021751
TREM1	2.520351	0.001333	0.035505
FKBP10	2.517367	0.000605	0.025109

CENPN	2.517048	6.30E-05	0.008981
GPT2	2.51077	1.07E-05	0.003269
SAA2	2.509064	0.003038	0.052587
C9orf140	2.503062	0.000169	0.014304
FNDC1	2.502904	5.76E-06	0.002331
THY1	2.4872	1.09E-05	0.003269
CTPS	2.485588	2.12E-05	0.004546
MYEOV	2.483652	3.78E-05	0.006668
IGFBP5	2.471631	1.24E-06	0.001291
CFI	2.459199	0.00132	0.035376
AJUBA	2.4511	0.001135	0.032951
MYC	2.450607	1.63E-06	0.001308
TGFBI	2.448321	1.83E-05	0.004289
CTPS	2.447355	0.000499	0.022681
CEP55	2.443376	0.00019	0.015016
TMEM211	2.437072	0.011352	0.10188
PRUNE2	2.431917	0.001111	0.032541
FAM89A	2.430491	6.24E-05	0.008951
SPON2	2.418456	9.83E-06	0.003215
na	2.416102	1.65E-05	0.004132
ATAD2	2.414372	0.000173	0.014387
MMP12	2.403637	0.034143	0.183803
TTLL10	2.397291	0.00025	0.016779
SLC29A1	2.396004	0.002448	0.047424
IL17RD	2.395888	0.005331	0.068007
HKDC1	2.394402	0.000984	0.03071
TRIP13	2.389098	8.49E-06	0.00291
FADS2	2.38738	0.000535	0.023636
DGAT2	2.387167	9.01E-05	0.010648
CPZ	2.387023	0.000122	0.012224
na	2.385006	9.73E-06	0.003215
TG	2.384788	5.90E-05	0.008758
ABLIM2	2.382242	7.37E-05	0.00962
F2RL2	2.382226	0.000132	0.012679
UBTD1	2.379874	7.84E-06	0.002751
CDC25A	2.37865	0.00011	0.011458
FSCN1	2.375137	0.006601	0.076708
SULT2B1	2.372549	5.18E-06	0.002325
RAD54B	2.367344	7.31E-05	0.009581
CKAP2	2.36442	0.000824	0.028953
PDCD2L	2.363314	0.000127	0.01239
S100P	2.354233	0.00703	0.079167
KRT17	2.351593	0.001838	0.041501
NNMT	2.345289	0.000301	0.018054
SALL4	2.344805	0.000324	0.018527
CHEK1	2.341805	0.005332	0.068007
TPD52L1	2.335615	0.007976	0.084566

MCAM	2.3343	5.79E-05	0.00872
AZGP1	2.334001	0.002146	0.045033
ASCL2	2.333599	6.21E-05	0.008951
SELE	2.332757	0.00934	0.091895
DACH1	2.328941	0.001567	0.038615
TGIF2	2.326575	2.79E-05	0.005488
GREM1	2.324825	0.002075	0.044137
C10orf81	2.316229	0.003377	0.055466
C3orf26	2.313512	1.53E-05	0.004032
PMAIP1	2.311204	0.000108	0.011444
SMOX	2.309886	2.50E-07	0.000601
SLC35D3	2.299686	0.002797	0.050569
na	2.294977	1.30E-05	0.003626
SCD	2.292789	0.000913	0.029777
BUB1	2.279173	0.000992	0.030776
FAM40B	2.277561	1.38E-05	0.003785
G0S2	2.273773	0.005766	0.071357
IQGAP3	2.27248	6.59E-05	0.009173
ACSL6	2.272473	0.036997	0.192385
PLA2G16	2.270215	0.000101	0.01119
MYC	2.266606	0.000107	0.011394
GINS4	2.262617	0.00334	0.055282
C4BPB	2.259644	0.008592	0.087914
CDCA5	2.255423	0.000225	0.015796
C3orf14	2.254799	0.000525	0.023388
TNFSF15	2.24894	6.26E-06	0.002391
IL1A	2.237537	8.98E-05	0.010648
C1orf135	2.2358	0.000214	0.015563
FOXS1	2.235341	1.58E-06	0.001299
RGS16	2.234802	0.000143	0.013308
IER3	2.224643	0.0007	0.026452
HS6ST2	2.224079	0.009321	0.091808
EDNRA	2.217699	0.000154	0.013799
PFDN4	2.217125	0.000653	0.025813
CHAC1	2.215031	0.000745	0.027304
ANGPT2	2.214517	1.09E-05	0.003269
GUCA1B	2.213338	0.000309	0.01814
DCUN1D5	2.213247	0.000733	0.02718
FANCB	2.213166	4.32E-05	0.007239
IFI44L	2.210272	0.002004	0.043223
PHGDH	2.205914	0.000235	0.01617
PYCR1	2.203182	0.000107	0.011421
TIMP1	2.199601	5.93E-05	0.008758
GINS1	2.196751	0.001614	0.039191
KIF2C	2.190105	0.000182	0.014786
NR4A2	2.185725	0.000696	0.026359
ORC6	2.178663	0.000171	0.014319

AZGP1	2.177227	0.006703	0.077154
PF4	2.176497	0.000381	0.019875
KLF7	2.175904	1.60E-05	0.004132
LY6E	2.174783	3.28E-06	0.001782
BMP7	2.173079	0.02037	0.138594
LOXL2	2.170989	0.000448	0.021666
C11orf53	2.168706	0.009538	0.093043
S100A12	2.168066	0.009382	0.092106
KAL1	2.166349	0.00012	0.012128
CDH11	2.160696	0.000677	0.026073
DDX10	2.160473	0.001053	0.031583
IER5L	2.159038	0.001477	0.03769
HTRA3	2.158403	6.57E-05	0.009173
FAM72D	2.153272	0.000103	0.011247
LMTK3	2.152688	0.002563	0.048607
ULBP3	2.152442	8.29E-05	0.01015
CKS2	2.150646	0.000165	0.014211
UBE2T	2.143701	0.000669	0.026049
TBC1D16	2.139302	0.000635	0.025626
PRSS22	2.136355	0.002029	0.043618
HAPLN3	2.132143	3.55E-05	0.006435
CITED4	2.13158	0.000122	0.012224
C8orf84	2.13146	0.026256	0.159267
CENPF	2.128766	4.74E-05	0.007746
GNG4	2.126506	0.000111	0.011458
FANCD2	2.125125	4.16E-06	0.002094
MELK	2.123255	0.000282	0.017824
FAM84B	2.122778	0.000828	0.028953
CENPW	2.122084	0.001569	0.038615
MAD2L1	2.121424	0.000846	0.029272
TTK	2.120838	0.000262	0.017247
ELOVL5	2.113597	5.63E-05	0.00863
TRNP1	2.110051	0.045061	0.214929
ZAK	2.109429	1.67E-05	0.004132
OTX1	2.104922	0.00213	0.044809
IFITM1	2.100722	3.54E-05	0.006435
EREG	2.100005	0.001745	0.040395
HJURP	2.096033	0.000498	0.022678
CTSL2	2.089486	7.28E-05	0.009575
MET	2.08544	0.001491	0.037812
PLXNA1	2.084428	0.001309	0.035307
SKA3	2.083979	0.000265	0.017247
BRCA1	2.081545	0.000302	0.018054
GPX8	2.081145	1.94E-05	0.004386
CDCA3	2.077594	0.000271	0.017383
DBNDD1	2.0767	0.000251	0.016791
CIT	2.070122	0.003799	0.058693

PHLDA2	2.06543	0.00093	0.030067
RACGAP1	2.063138	0.000176	0.014575
IFITM1	2.062448	7.05E-05	0.009523
MFAP2	2.060352	2.28E-05	0.004727
BAG2	2.058444	0.003087	0.053029
MZT1	2.053311	0.000133	0.012708
KIF15	2.05038	0.002349	0.046631
FAM54A	2.047852	1.34E-06	0.001291
MAP7D2	2.04054	0.000161	0.014104
na	2.039793	0.001718	0.040279
LEF1	2.039037	0.000101	0.01119
HTRA3	2.036615	1.17E-05	0.003406
CCDC150	2.036289	3.10E-06	0.001741
RAD54L	2.035736	0.000666	0.026049
NOTCH4	2.034208	4.37E-06	0.002167
ERRFI1	2.030545	0.001564	0.038613
CHEK2	2.02885	0.000103	0.01121
GPR116	2.028119	0.011321	0.101796
RGS4	2.024019	0.003992	0.059925
C8orf4	2.022359	0.00178	0.040876
TPX2	2.019705	0.000191	0.015016
CTGF	2.019212	2.19E-05	0.004614
GTF2IRD1	2.017196	0.000147	0.013458
CCNF	2.015431	0.001218	0.034193
PLS3	2.014668	6.59E-05	0.009173
ENST0000C	2.013328	1.87E-06	0.001403
C12orf48	2.012645	0.004797	0.064885
ENST0000C	2.01225	0.000121	0.012216
AGT	2.009886	0.00046	0.021838
PLEKHB1	2.006978	0.00029	0.017929
CEP55	2.000167	0.000426	0.021173
CENPN	1.999234	3.64E-05	0.006501
STC1	1.997819	0.004002	0.060037
IFI6	1.996289	0.000109	0.011456
SNTB1	1.979938	0.003244	0.054426
HSPH1	1.979782	3.97E-05	0.006861
TTC23L	1.975888	0.000273	0.017383
PDGFRB	1.97557	0.004165	0.060999
OR2T6	1.97429	0.000257	0.017125
HTRA1	1.974052	0.000114	0.011696
CHAF1B	1.973168	0.000904	0.029689
PTGES	1.972881	0.011132	0.100985
CCNB1	1.966715	0.0012	0.034037
VWA2	1.962836	1.65E-05	0.004132
MEX3A	1.96063	0.02568	0.157446
CFI	1.955321	0.003867	0.05913
ITGA2	1.951922	0.000378	0.019839

DDX21	1.951649	0.004591	0.063673
LY6G6D	1.949357	0.030126	0.171469
CASC5	1.949346	8.39E-05	0.01024
DNAH14	1.946629	0.002676	0.049316
ZNRF3	1.943955	0.007786	0.083473
CENPI	1.94374	0.000352	0.01927
KIF23	1.942389	7.23E-05	0.00955
MGP	1.942285	0.000402	0.020525
ATRIP	1.939684	6.32E-05	0.008981
CSE1L	1.938044	0.000155	0.013802
CD93	1.934503	0.003199	0.053859
COL1A2	1.933718	0.000622	0.025453
CACNG1	1.932787	7.16E-05	0.009538
CENPI	1.929743	0.000123	0.012224
TM4SF1	1.929499	0.001519	0.038036
PMEPA1	1.929173	0.00017	0.014304
PSRC1	1.928418	0.001219	0.034193
GALNT6	1.926755	0.000235	0.01617
NMB	1.92046	0.000238	0.016263
PVT1	1.91914	0.000583	0.024719
TOMM34	1.918838	0.000334	0.018849
EDNRA	1.916636	0.001281	0.03496
PTRH1	1.916501	0.000379	0.019839
GTF2IRD1	1.915983	0.00122	0.034193
MMP11	1.915136	0.000992	0.030776
BIRC5	1.913756	7.02E-05	0.009523
HMMR	1.913102	0.005703	0.070827
DSN1	1.91151	0.000336	0.018936
ETV4	1.909913	5.60E-06	0.002331
DCDC2	1.907677	0.007659	0.082625
OR12D3	1.907663	0.000512	0.023103
NR4A1	1.904038	0.017256	0.127442
KRT80	1.900557	0.013574	0.111595
CBX3	1.896062	0.000304	0.018061
SIK1	1.895072	0.005126	0.066816
URB2	1.894494	0.005064	0.066451
CENPI	1.893829	0.000965	0.030507
ZAK	1.891434	0.000125	0.012342
NOTCH3	1.888437	0.000775	0.027933
DHCR7	1.888109	0.004714	0.064638
RGS16	1.887631	0.000346	0.019206
LOX	1.884538	0.008871	0.089178
WISP1	1.883074	0.00097	0.030507
PXDN	1.881949	0.007687	0.082782
LAPTM4B	1.881928	0.000471	0.022013
na	1.88075	0.001868	0.04172
IER3	1.877263	0.000123	0.012224

COL6A3	1.874791	0.000264	0.017247
ESCO2	1.873739	0.007055	0.079245
CDKN3	1.873034	0.007358	0.080814
HCAR3	1.872354	0.00033	0.018737
SNAI1	1.872301	0.000279	0.017684
LMBR1	1.871432	0.005778	0.07142
CDK1	1.870101	0.002859	0.051253
PARD6A	1.868269	5.80E-06	0.002331
KIAA1257	1.86822	0.004408	0.06241
FMO3	1.867346	0.000746	0.027304
CSE1L	1.867339	0.000288	0.017929
GTPBP4	1.867026	0.001086	0.032224
CDC45	1.866715	0.002783	0.050428
GRHL1	1.864369	0.002986	0.052281
NEBL	1.857664	0.000464	0.021881
GPR4	1.856625	0.000549	0.023973
PUS7	1.851809	0.010843	0.099069
CREM	1.850927	0.000185	0.014888
DUSP14	1.850698	0.000191	0.015016
HAUS7	1.850664	0.000375	0.019784
FADS1	1.849298	0.000236	0.0162
CEBPB	1.847823	0.000619	0.025381
KIFC1	1.846351	0.000419	0.021069
KDELC2	1.846313	0.004321	0.061771
TDGF1	1.845892	0.00068	0.02615
BCL2A1	1.843354	0.004512	0.063084
C12orf48	1.841973	0.000855	0.0294
RNF183	1.840568	0.004516	0.063098
na	1.839383	0.000559	0.024133
C11orf84	1.83925	9.70E-05	0.010965
OTUD6B	1.839151	0.000589	0.024788
CRABP2	1.838475	0.016155	0.122589
KCNJ14	1.829937	0.003308	0.054973
HYAL3	1.829697	7.36E-06	0.002653
OIP5	1.829659	0.003907	0.059394
C13orf27	1.829581	0.000165	0.014211
KDELC1	1.828422	1.08E-05	0.003269
CEP164	1.827913	2.05E-05	0.004494
DIAPH3	1.825926	3.32E-05	0.006241
TFPI2	1.822883	0.047012	0.219095
TGM2	1.822643	0.003704	0.05804
TCFL5	1.822633	0.000372	0.019782
WNT5A	1.821951	0.000164	0.014202
IGF2	1.816997	0.000946	0.030238
FAM72A	1.815598	0.000332	0.018773
TIPIN	1.815277	0.001233	0.034335
RDH10	1.813063	0.01153	0.102643

CCNB1	1.812187	0.001815	0.041279
MLXIPL	1.811819	0.001716	0.040279
KIF14	1.809428	0.003363	0.055387
CCDC3	1.808062	0.000387	0.020052
HAUS7	1.806803	0.000237	0.0162
HS6ST2	1.805089	0.049484	0.225065
IRS2	1.804238	0.023888	0.15176
CHN1	1.803377	0.000203	0.015344
LRRC8E	1.801041	0.000602	0.025049
JPH1	1.797704	0.022531	0.146889
EGR1	1.797208	0.018294	0.131239
CREB5	1.796718	0.001044	0.031472
RIPK2	1.795494	6.37E-05	0.009017
IL33	1.788513	0.013918	0.113046
RHOB	1.786088	0.002739	0.049909
GPX8	1.785246	0.000211	0.015409
TROAP	1.782117	0.00033	0.018727
PSMG1	1.779346	0.000128	0.01239
FOSL1	1.778315	0.001184	0.033824
NUSAP1	1.778268	0.000102	0.01119
KIF24	1.776088	0.000227	0.015885
GMPR	1.775737	0.002968	0.052177
CTSK	1.771154	7.13E-05	0.009538
CLCN4	1.769531	0.017389	0.127972
FBXO41	1.769232	0.007788	0.083473
PSG5	1.767935	0.01083	0.099047
RPL36AP33	1.767003	0.000162	0.014159
MBTPS2	1.766637	3.78E-05	0.006668
CCNE2	1.765529	0.000267	0.017271
SMC2	1.762688	0.000277	0.017628
INSIG1	1.760553	0.006169	0.073819
PRUNE2	1.76015	0.004146	0.060956
HAUS6	1.758291	0.000347	0.019206
IPPK	1.756741	0.000143	0.013308
PMEPA1	1.754986	0.000772	0.02784
LIN7A	1.754624	0.000638	0.025626
DKC1	1.75132	0.001399	0.036458
CKAP2	1.751216	0.003078	0.052953
SLC27A5	1.74967	0.001658	0.039662
RGS5	1.749285	0.00047	0.022013
IFRD2	1.74907	0.001047	0.031499
SPC25	1.747661	0.002128	0.044805
DMBT1	1.746255	0.042432	0.207069
RECQL4	1.745669	0.000164	0.014202
MST152	1.744282	0.000932	0.030098
RRP15	1.74414	0.005277	0.067876
RFC3	1.743159	0.002912	0.051814

OLFML2B	1.742961	0.00017	0.014304
LRRC34	1.742676	0.004433	0.062578
TOP2A	1.739957	0.001662	0.039681
KIF9	1.739936	0.000674	0.026049
CCL4	1.736407	4.99E-05	0.008079
XPO5	1.733611	0.000728	0.027114
CCND1	1.731889	0.008986	0.089822
TNC	1.730836	0.001108	0.032541
TMEM97	1.729791	0.001246	0.034391
PTTG1	1.729672	0.001942	0.042526
DDX10	1.724147	0.005322	0.068007
GINS3	1.723599	0.001867	0.04172
MKI67	1.722594	0.000761	0.027699
ASPM	1.722175	0.010321	0.096624
ASPHD1	1.722063	0.000291	0.017929
REG3A	1.721691	0.027812	0.164271
SORD	1.721486	0.000894	0.029588
C12orf73	1.717175	0.000328	0.018674
SLC25A32	1.716728	0.000223	0.015789
CHTF18	1.712063	0.000235	0.01617
GART	1.709422	0.000126	0.012344
ROBO2	1.708695	0.000851	0.029331
CDKN3	1.706148	0.0008	0.028465
DBF4	1.705243	0.000951	0.030348
SGOL1	1.704959	0.002645	0.049127
LYAR	1.699317	4.06E-05	0.006977
AKR1E2	1.697483	0.017828	0.129604
SLC39A10	1.69683	0.000977	0.030643
C15orf23	1.695664	0.000588	0.024775
SPC24	1.694021	5.41E-05	0.008434
PFKFB3	1.693537	0.002344	0.046631
NT5DC2	1.693501	0.00395	0.05954
P4HA1	1.692668	0.003423	0.055831
TM4SF1	1.692195	0.001677	0.039785
ENST0000C	1.691739	0.001503	0.037931
MTHFD1L	1.687263	7.66E-05	0.009704
SHC3	1.682871	0.026461	0.15986
GREM1	1.681518	0.002974	0.052177
SERPINH1	1.679723	0.000735	0.027192
PPM1H	1.679547	0.005299	0.067974
GBP1	1.679078	0.006026	0.072898
KIF4A	1.678977	0.000604	0.025086
EZH2	1.67718	0.006861	0.078174
C11orf82	1.676426	0.000696	0.026359
CBX3	1.675649	0.00058	0.024654
KIAA0101	1.67396	0.009901	0.094652
PSMD14	1.673649	0.000996	0.030777

SLC5A6	1.671073	0.001613	0.039191
SACS	1.670843	2.44E-05	0.004912
XPO4	1.669898	0.003316	0.055075
BLM	1.66957	0.002158	0.045072
CCL2	1.669211	0.000441	0.021545
PLK1	1.66831	0.000486	0.022369
FAM188B	1.667735	0.000413	0.020865
SERPINE2	1.666489	0.010834	0.099055
ZWILCH	1.665999	0.004559	0.063356
BGN	1.665701	0.001813	0.041279
MTHFD1L	1.664787	0.00094	0.030182
MACC1	1.664753	0.001665	0.039681
LAMP3	1.663767	0.015181	0.118293
NCAPG2	1.658673	0.002163	0.045072
NUPL1	1.65776	0.00321	0.053954
MYBL2	1.656748	0.000497	0.022676
TOMM34	1.654898	0.000541	0.023709
IFITM3	1.654844	0.000188	0.015016
HSPH1	1.654632	0.000302	0.018054
GSG2	1.65409	0.003	0.052334
TCFL5	1.652178	0.000132	0.012706
IMMP2L	1.651293	0.006205	0.074113
PLK1	1.648454	0.000666	0.026049
LUM	1.648117	0.000386	0.020042
SMC2	1.647637	0.001851	0.041537
EXOSC2	1.647459	0.002221	0.045511
PTGER3	1.646829	0.001245	0.034391
TMEM132A	1.646383	0.003065	0.052777
P2RX3	1.645714	0.009584	0.093306
TIMELESS	1.645308	0.005127	0.066816
TCTEX1D2	1.643098	0.000584	0.024719
NAMPT	1.643068	0.001963	0.042748
VASN	1.641025	0.002297	0.0463
DIAPH3	1.638858	0.000506	0.022878
GUCY1A3	1.637071	0.000142	0.013274
WDR77	1.637007	0.000373	0.019782
RDM1	1.636911	0.005359	0.068193
HAUS2	1.633132	0.001103	0.032479
CDT1	1.632518	0.000596	0.024991
DUSP10	1.632142	0.004745	0.064703
TRIP6	1.631597	0.004224	0.061247
FZD6	1.626182	0.000502	0.022766
SLC2A1	1.625816	0.006337	0.074977
PPM1N	1.625071	0.000493	0.022555
DKK3	1.623667	0.000264	0.017247
MCMBP	1.62325	0.000632	0.02561
ME1	1.623161	0.000714	0.026741

PRKDC	1.622747	0.001857	0.041598
RAD23B	1.622307	0.015375	0.11919
PPIL1	1.622241	0.007993	0.084644
CKS1B	1.622232	0.000589	0.024788
ETV7	1.620142	0.000119	0.012082
TPM2	1.619022	0.00113	0.032847
ARHGEF19	1.618826	0.01783	0.129604
DSC3	1.618668	0.000972	0.030552
COL3A1	1.61362	0.000857	0.0294
TCF19	1.613064	0.002188	0.045346
SUV39H1	1.611818	0.000225	0.015796
KLHL21	1.611255	0.006139	0.073611
IRAK1	1.610696	0.000543	0.023765
LGR5	1.610321	0.01211	0.105808
PSAT1	1.609825	0.001988	0.043006
CCL3	1.609561	0.000203	0.015344
RFC3	1.609103	0.000897	0.029634
C8orf55	1.606355	0.000524	0.023388
C16orf87	1.606302	0.004817	0.064885
PRC1	1.605465	0.002014	0.043372
LAPTM4B	1.604634	0.003004	0.052334
UBE2H	1.604256	0.003815	0.058802
ANP32E	1.60409	0.002692	0.049328
ULBP2	1.60336	0.000695	0.026359
NAMPT	1.602528	0.014909	0.117211
DEPDC1	1.601197	0.001723	0.040322
CDCA7	1.600276	0.001416	0.036692
GRB10	1.598208	0.008596	0.087914
BNIP3	1.598049	0.002048	0.043794
SYN1	1.597562	0.04636	0.21788
TPM2	1.596443	0.000741	0.0273
NCOA7	1.592046	0.000574	0.024489
SPTBN2	1.59019	0.000862	0.029439
SLC2A8	1.589905	0.000509	0.022997
IGF2	1.588483	0.002614	0.048991
FCGR1B	1.588014	0.009195	0.091072
SORD	1.587809	0.003513	0.056475
ATF3	1.587216	0.043431	0.209889
ProSAPiP1	1.586293	0.005399	0.068503
CDK2	1.586272	0.000635	0.025626
ANXA3	1.586218	0.00036	0.019406
CENPH	1.5857	0.000828	0.028953
MORC4	1.584259	0.000732	0.02718
NEBL	1.580596	0.001119	0.032657
KIAA1430	1.580108	0.017865	0.129743
ABCB6	1.579123	0.003616	0.057184
CENPP	1.578978	0.000337	0.018991

ELTD1	1.577855	0.002987	0.052281
NDC80	1.57455	0.007869	0.084149
ENST0000C	1.573	0.002665	0.049207
PLAUR	1.571201	0.004115	0.060737
EARS2	1.570051	0.008497	0.087298
PLAU	1.568606	0.000529	0.023455
PSMG4	1.568514	0.000498	0.022678
SGOL2	1.566815	0.002892	0.051643
GUCY1B3	1.5656	3.83E-05	0.006678
RNFT2	1.564549	0.0004	0.020525
NAMPT	1.563804	0.00084	0.029214
CHCHD6	1.563492	0.000359	0.019393
KIRREL	1.563354	0.000404	0.020554
ACCN2	1.561976	0.000839	0.029214
PTPLA	1.560971	0.000377	0.019839
AURKA	1.560675	0.001595	0.038953
ABCC1	1.559941	0.001276	0.034854
MGC11082	1.557625	0.024061	0.152178
LYPD3	1.557121	0.037027	0.192412
IL6	1.555128	0.002541	0.048357
AMOTL2	1.554413	0.00197	0.042764
CTLA4	1.552034	0.004155	0.060975
C5orf28	1.550338	0.006485	0.075956
TTC26	1.550129	0.022074	0.145235
SERPINA12	1.549138	0.003961	0.059613
F12	1.547781	0.001288	0.035027
CARD6	1.546073	0.004015	0.060074
OAS3	1.545963	0.000476	0.022188
Q6IHG2	1.544778	0.003811	0.058802
ROBO4	1.542006	0.000412	0.020865
DEK	1.541888	0.003833	0.058887
CCDC103	1.539823	0.000711	0.026702
CCNE1	1.536965	0.007901	0.084171
ZNF593	1.536592	0.000288	0.017929
UHRF1	1.536203	0.007389	0.080929
GEM	1.53529	0.000166	0.014211
PRDX4	1.535214	0.001312	0.035342
DTYMK	1.534478	0.002633	0.049127
AFAP1L1	1.534203	0.000139	0.013131
na	1.533297	0.000392	0.020183
METTL11A	1.531397	0.000597	0.024991
TMEM123	1.530705	0.005956	0.072511
MGP	1.529385	0.003354	0.055379
ISG15	1.526574	0.000864	0.029443
PSMD14	1.526144	0.002636	0.049127
GYLTL1B	1.525008	0.01932	0.135103
LPCAT1	1.521485	0.009754	0.093955

URB1	1.521454	0.006101	0.07329
C2	1.520688	0.000524	0.023388
ASPM	1.519806	0.004728	0.06467
GLS	1.517456	0.000351	0.01927
RAD51AP1	1.51525	0.000586	0.024765
LOXL1	1.515129	0.000284	0.017861
CFB	1.514829	0.002488	0.047855
C11orf96	1.513018	0.002163	0.045072
GPN3	1.512159	0.000908	0.029721
RUVBL1	1.50967	0.000192	0.015016
ACOT9	1.509428	0.000881	0.029547
PYCRL	1.508577	0.000639	0.025626
S100A11	1.505116	0.000313	0.018271
FGGY	1.505065	0.018697	0.133065
ENST00000	1.504592	0.001734	0.040395
GOLT1A	1.503888	0.011524	0.102643
PROCR	1.503086	0.014318	0.114795
GMPS	1.502671	0.000654	0.025825
SRM	1.5017	0.000966	0.030507
RBL1	1.501053	0.000739	0.027291
LIMK1	1.49892	0.000848	0.029272
C2orf70	1.498581	0.011502	0.102643
SPC24	1.497659	0.001831	0.041455
WDR62	1.497644	0.0074	0.080929
GRPEL2	1.495401	0.000848	0.029272
BOP1	1.49452	0.000283	0.017845
FANCI	1.494062	0.000169	0.014304
S100A11	1.492285	0.000982	0.030689
TMEM52	1.492079	0.003394	0.055607
CTNNAL1	1.491733	0.000886	0.029559
EIF1AX	1.490222	0.000353	0.01927
LAMC2	1.489959	0.012058	0.105554
GALK1	1.487773	0.00021	0.015409
CENPF	1.487755	0.001488	0.037812
PRPF4	1.485882	0.000388	0.020096
TBX18	1.484925	0.026308	0.159437
ECE2	1.483336	0.000313	0.018271
LRRC6	1.480091	0.01096	0.099845
FAM188B	1.48009	0.000321	0.018409
ODF2	1.478006	0.002687	0.049328
SFRP2	1.477273	0.003015	0.0524
KREMEN2	1.474919	0.006905	0.078446
MRTO4	1.474649	0.001505	0.037931
FCAR	1.474542	0.003117	0.05319
RAD1	1.473914	0.001244	0.034391
E2F6	1.472264	8.89E-05	0.010613
RNF8	1.469593	0.003676	0.057802

MTHFD2	1.46918	0.000724	0.027008
IMPDH1	1.4684	0.004115	0.060737
METTL1	1.46824	0.000722	0.026961
POC1A	1.467989	0.005406	0.068506
MRPS17	1.467599	0.000301	0.018054
RHEB	1.466693	0.003027	0.052507
DNAH14	1.466038	0.004508	0.063084
C12orf11	1.46563	0.007628	0.082506
GPR19	1.464628	0.004538	0.063194
PIR	1.464427	4.26E-05	0.007169
CLCN1	1.462734	0.038843	0.1975
NPRL3	1.462374	0.010235	0.096195
KYNU	1.462324	0.00427	0.061416
RND1	1.460725	0.016289	0.123188
GJB3	1.460595	0.025918	0.158075
SNORA75	1.458158	0.000526	0.02339
C9orf21	1.456269	0.000245	0.016594
WDR3	1.454545	0.013391	0.110741
THBD	1.453833	0.036443	0.191027
DACH1	1.453805	0.007148	0.079722
LMCD1	1.453346	0.00068	0.02615
C20orf27	1.453217	0.000219	0.015622
ARID3A	1.451977	0.043686	0.210788
PDRG1	1.451237	0.002318	0.046459
FEN1	1.450149	0.00056	0.024133
WDR43	1.449222	0.003879	0.059152
NACC2	1.44801	0.006624	0.076758
WDR90	1.447667	0.009688	0.093613
RECQL	1.44713	0.000664	0.026024
ANTXR1	1.446734	0.01553	0.119923
ASB6	1.44532	0.000193	0.015016
C15orf42	1.445048	0.00125	0.034438
KIF23	1.444733	0.003608	0.057177
PDCD5	1.44347	0.012888	0.108854
AASDHPPT	1.442728	0.04965	0.225454
MND1	1.442684	0.014555	0.115796
SPNS2	1.442303	0.005793	0.071488
ZP3	1.441739	0.002502	0.047996
TPD52L2	1.440843	0.000595	0.024991
KIF20A	1.440622	0.001141	0.033062
DYSF	1.440496	0.000996	0.030777
NEK2	1.439597	0.001375	0.036027
MRPL50	1.438758	9.37E-05	0.010731
QTRTD1	1.438082	0.021528	0.143114
TCEB1	1.437954	0.001298	0.035227
IGFBP7	1.437517	2.87E-05	0.005614
SDCCAG3	1.436809	0.001977	0.042806

E2F1	1.436629	0.005916	0.072171
KIAA1958	1.435932	0.004772	0.064811
RRS1	1.435898	0.010253	0.096254
BRIX1	1.435764	0.000479	0.02226
P39193	1.435209	0.006605	0.076708
HIST1H2AL	1.434247	0.001632	0.039405
ENST0000C	1.433657	0.004347	0.06196
SLC2A3	1.433147	0.001163	0.033377
VMA21	1.432464	0.00356	0.056863
PARVB	1.431042	0.015473	0.119763
IFI44	1.430742	0.009131	0.090645
ZNF48	1.430208	0.003417	0.055793
NID1	1.429764	0.016319	0.123301
EPHB4	1.428966	0.005597	0.070144
TERF1	1.428332	0.000575	0.0245
IARS	1.427116	0.000203	0.015344
TUBB6	1.426733	0.001567	0.038615
HYAL2	1.42625	0.006402	0.075459
RASIP1	1.425204	0.002281	0.046266
PHF16	1.424628	0.002433	0.047326
PUSL1	1.42335	0.001817	0.041279
CXCR7	1.419929	0.001378	0.036068
MED30	1.417205	0.011884	0.104658
E2F8	1.415826	0.036285	0.190635
CALD1	1.415719	0.000201	0.015322
MCFD2	1.414443	0.004019	0.060088
EPHB2	1.413815	0.034564	0.185275
NSDHL	1.413416	0.002491	0.047855
POLR1C	1.413133	0.003752	0.058486
SLC25A22	1.412891	0.025737	0.157621
CD34	1.411755	0.000985	0.03071
FLVCR1	1.411723	0.000617	0.025359
EIF2S2	1.411541	0.000891	0.029559
SRXN1	1.41135	0.006237	0.074196
TMEM9	1.411221	7.80E-05	0.00977
HENMT1	1.410522	0.000405	0.02056
IL24	1.410392	0.005063	0.066451
VEGFA	1.409917	0.000383	0.019921
ADAMTS1	1.408509	0.002508	0.047996
NDUFA4L2	1.407912	0.000459	0.021838
MFSD2A	1.406762	0.019937	0.137111
KIAA0020	1.406332	0.000111	0.011475
TRMT12	1.40549	0.029582	0.169651
HIST2H3A	1.403761	0.000164	0.0142
FTSJ1	1.403333	0.003193	0.053779
MCM3	1.403195	0.001728	0.040391
DCAF13	1.402675	0.001073	0.031994

HIST1H3I	1.402386	0.000669	0.026049
BACE2	1.399292	0.00661	0.076723
NUP35	1.398631	0.006438	0.075585
CXCL5	1.397571	0.020876	0.140445
ZNF696	1.397523	0.001649	0.039553
BORA	1.397238	0.001485	0.037775
LIN9	1.395695	0.010888	0.099351
ODC1	1.395281	0.001771	0.040765
GPATCH4	1.394834	0.006152	0.073689
MKI67	1.394016	0.000172	0.014387
na	1.392391	0.01833	0.131357
RRP9	1.389719	0.000197	0.01519
KDR	1.389004	0.000806	0.028548
TMEM237	1.388568	0.000831	0.029018
SLC5A6	1.387114	0.000798	0.028425
CCL3L3	1.386755	0.000708	0.026656
KIAA1826	1.386468	0.011107	0.100874
RPA3	1.385315	0.007933	0.084343
GGCT	1.385019	0.001433	0.036969
MCM6	1.38496	0.004786	0.06486
NUF2	1.38457	0.000188	0.015016
PSMA7	1.383035	0.003124	0.053222
MME	1.382995	0.036056	0.189966
PTTG2	1.38141	0.000842	0.029247
C11orf95	1.381109	0.00724	0.080058
LAGE3	1.381057	0.000994	0.030777
P39193	1.377661	0.002625	0.049127
ARHGEF10	1.37592	0.02335	0.149527
ACOT9	1.374588	0.014866	0.116971
LACTB2	1.373439	0.000922	0.029915
CCDC34	1.373194	0.003874	0.059152
CDK5RAP1	1.371537	0.002713	0.049532
MSH2	1.370892	0.003712	0.058094
SLBP	1.369533	0.005156	0.067034
LMNB2	1.369104	0.002411	0.047216
MEST	1.369025	0.016544	0.124419
FAM57A	1.36896	0.019474	0.135641
FBXO5	1.367596	0.002988	0.052281
ANXA1	1.366336	0.004988	0.06594
NCF2	1.365817	0.001202	0.034037
BMP7	1.364752	0.025607	0.157319
PXDN	1.363554	0.02524	0.156139
RGS1	1.36151	0.000267	0.017271
PSPH	1.361013	0.035244	0.187291
ZNF280A	1.360696	0.001162	0.033375
C17orf53	1.360309	0.000317	0.018331
NUDT1	1.360015	0.00138	0.036097

ARL6IP1	1.359778	0.048666	0.223236
NLE1	1.359654	0.00616	0.073759
EEF1E1	1.359399	0.001014	0.031066
EXOSC4	1.356734	0.000289	0.017929
JUN	1.356563	0.005276	0.067876
DDX28	1.355617	0.002488	0.047855
SLC16A4	1.355584	0.004262	0.061391
GEMIN6	1.35552	0.005801	0.071488
DNMT3B	1.35495	0.010411	0.097167
ENST00000(	1.35395	0.004195	0.061078
VMA21	1.352567	0.000156	0.013802
ATAD3B	1.352499	0.000106	0.011394
DDX27	1.351981	0.007211	0.07993
NID2	1.351976	0.008216	0.085898
SPIN4	1.351183	0.009692	0.09362
MLF1	1.350893	0.000139	0.013131
HIP1	1.350252	0.000557	0.024133
ZNF503	1.348337	0.003573	0.056863
ATAD3C	1.347103	0.001508	0.037934
MLF1IP	1.346052	0.004519	0.063098
CSTA	1.345642	0.018857	0.133693
ASPN	1.344619	0.002684	0.049328
NSMCE1	1.344398	0.013411	0.110771
C20orf111	1.343774	0.002402	0.047136
TWISTNB	1.343342	0.006148	0.073668
ENST00000(	1.342984	0.000794	0.028331
CTNNAL1	1.34221	0.002403	0.047136
PPA1	1.342135	0.001286	0.034997
FERMT2	1.341358	0.00127	0.034818
ART3	1.340707	0.030969	0.173948
GDF15	1.340302	0.011095	0.100785
STAU2	1.338727	0.039488	0.199297
MX1	1.338458	0.007987	0.084621
KPNA2	1.338428	0.000999	0.030825
TTC12	1.338197	0.003474	0.056299
FPGS	1.336981	0.025341	0.156414
SOCS3	1.335567	0.003834	0.058887
na	1.335243	0.02191	0.14456
PPA1	1.334351	0.000966	0.030507
IL1RAP	1.334047	0.012911	0.108883
TUBG1	1.334043	0.005046	0.066389
DRAM1	1.333731	0.002349	0.046631
KIF3C	1.333157	0.001229	0.034276
C20orf20	1.332144	0.003349	0.055318
TSPAN5	1.331949	0.000287	0.017929
PBK	1.331511	0.005656	0.070489
DDIT4	1.331328	0.024911	0.155018

THOC2	1.330169	0.002323	0.046489
ENST0000C	1.330165	0.013163	0.109746
CTU2	1.32992	0.00208	0.044147
TFAP4	1.329818	0.043071	0.20896
PTPDC1	1.329712	0.000373	0.019782
ENC1	1.329502	0.000348	0.01925
RNASEH2A	1.329395	0.000451	0.021714
NXT1	1.328484	0.005681	0.070659
HIST1H3H	1.3283	0.005459	0.068888
GSTM3	1.327731	0.000152	0.013681
POLD3	1.327514	0.002454	0.047507
EIF1AX	1.32729	0.000612	0.025233
C13orf15	1.326713	0.005178	0.067205
TAP2	1.326384	0.027263	0.162767
EEF1E1	1.326103	0.001734	0.040395
PMCH	1.325771	0.000405	0.020554
EBNA1BP2	1.325142	0.001857	0.041598
GNA15	1.324163	0.003834	0.058887
TBC1D30	1.324045	0.004231	0.061254
LIF	1.324022	0.029215	0.168249
WDR34	1.323441	0.000978	0.030643
LRRC20	1.322967	0.003083	0.053011
SLC19A2	1.320711	0.013045	0.109332
POMP	1.320554	0.004627	0.063995
HIST3H2A	1.320278	0.000991	0.030776
LBH	1.319556	0.00035	0.019259
TMEM139	1.319047	0.011857	0.104447
PPA1	1.319003	0.004315	0.061771
SPARC	1.318973	0.004896	0.065366
SLC12A2	1.318658	0.007223	0.079984
PRSS23	1.317811	0.020751	0.140181
MAPRE1	1.317277	0.007274	0.080211
ANKS6	1.315133	0.005551	0.069767
CKS1B	1.314997	0.000942	0.030197
RCC1	1.313652	0.000939	0.030182
TACC3	1.312563	0.002037	0.043698
TREM1	1.311372	0.022204	0.145573
GTF3A	1.311291	0.006106	0.07332
SOX9	1.31041	0.018836	0.133692
AHCY	1.310323	0.00063	0.02557
TTC39B	1.309322	0.00222	0.045511
NEK2	1.30887	0.002647	0.049127
BATF3	1.30851	0.001335	0.035529
ACTR3B	1.308416	0.022133	0.145438
ILF3	1.308095	0.019201	0.1348
DPM1	1.307591	0.003156	0.053367
MKI67IP	1.307023	0.011542	0.102686

PPIH	1.306815	0.002637	0.049127
AGMAT	1.305872	0.002024	0.043573
ITGB1BP1	1.305604	0.000742	0.027304
RNF43	1.305234	0.004909	0.065486
ERF	1.304644	0.020966	0.140876
CKAP2	1.304421	0.001669	0.03972
EIF4EBP1	1.303924	0.007594	0.082383
SAV1	1.303658	0.003783	0.058639
PPA1	1.302241	0.00152	0.038036
WDHD1	1.300969	0.008745	0.088535
TONSL	1.3005	0.002031	0.043623
IFITM2	1.30027	0.000748	0.027344
DCLRE1A	1.300189	0.035558	0.188262
RHEB	1.299742	0.003495	0.056475
TAGLN	1.294175	0.001266	0.034751
DCAF13	1.291995	0.000455	0.021751
TROAP	1.291512	0.000453	0.021751
VRK1	1.291473	0.007009	0.079069
GLO1	1.291472	0.001822	0.041294
NDE1	1.291061	0.000616	0.025359
VWA2	1.288989	0.002921	0.051896
na	1.28846	0.02325	0.149198
TCOF1	1.28828	0.010759	0.098751
C9orf117	1.288138	0.009726	0.093762
CCT4	1.287809	0.000229	0.016031
NAA38	1.287	0.001752	0.040505
C6orf154	1.286406	0.012352	0.106549
PTS	1.285621	0.025124	0.155565
STIP1	1.285616	0.00315	0.053347
TEAD2	1.28549	0.001032	0.031318
CSPG4	1.282365	0.005633	0.070408
ADRM1	1.281208	0.00132	0.035376
C17orf53	1.280564	9.63E-05	0.010955
C20orf46	1.28037	0.008368	0.086432
ADCY3	1.279877	0.017776	0.12944
FAM91A1	1.279853	5.15E-05	0.008209
NOP2	1.279701	0.000535	0.023636
HOXA11	1.279676	0.02028	0.138477
TMPO	1.279157	0.002278	0.046266
HMGB3	1.279017	0.003509	0.056475
CHEK2	1.27901	0.004157	0.060975
AMIGO2	1.278916	0.040263	0.201222
DUSP4	1.278731	0.029696	0.169923
CALU	1.277455	0.000597	0.024991
CCT2	1.277246	0.006703	0.077154
CDC25B	1.277153	0.000872	0.029538
FGFRL1	1.276965	0.009706	0.093676

HELLS	1.27631	0.01817	0.130774
PRPS1L1	1.27593	0.00803	0.084842
CENPQ	1.274851	0.001639	0.03949
NT5C3L	1.274647	0.003272	0.054573
HMGA1	1.272858	0.012293	0.106284
CTSH	1.27197	0.001537	0.0383
POLD2	1.271132	0.00046	0.021838
NME1	1.270837	0.001194	0.033978
KLHL5	1.270452	0.000662	0.025981
MAGOH	1.270265	0.014302	0.114755
EPHA2	1.270223	0.001033	0.031318
C17orf79	1.269946	0.010968	0.099881
SPAG5	1.268876	0.000863	0.029439
C10orf125	1.267222	0.002645	0.049127
RAI14	1.267093	0.000955	0.030348
DNMT1	1.267082	0.012176	0.106022
EPSTI1	1.266432	0.022767	0.147757
IFFO2	1.2661	0.021159	0.141663
ZDHHC9	1.264422	0.002054	0.043896
OAS3	1.264422	0.000125	0.012313
NAA11	1.26424	0.002646	0.049127
C12orf45	1.262938	0.005332	0.068007
GNG11	1.262243	0.006527	0.076129
MTERFD1	1.262228	0.002657	0.049196
PLOD1	1.26184	0.001685	0.039823
PGM2	1.26184	0.002457	0.047507
RHOB	1.261799	0.021537	0.143147
DNAJA1	1.261432	0.000345	0.019206
RAP2A	1.260328	0.031532	0.176094
FAM207A	1.259822	0.000374	0.019784
G6PC3	1.25937	0.001286	0.034997
SNRPF	1.259164	0.015719	0.120648
ATP6V1E2	1.259139	0.004382	0.06224
AGTRAP	1.258145	0.000537	0.023659
DPY19L1	1.257174	0.002585	0.048803
DENND5A	1.257075	0.024855	0.154856
NOLC1	1.257014	0.00396	0.059613
GPR157	1.256003	0.000192	0.015016
EXOSC3	1.255335	0.003541	0.056716
SSB	1.254758	0.000653	0.025813
ZNF259	1.253453	0.003394	0.055607
REXO2	1.2522	0.008629	0.087995
EXO1	1.252075	0.000421	0.021076
PTPN12	1.251519	0.00939	0.092131
TMEM110	1.250908	0.000636	0.025626
MAPRE1	1.250322	0.009126	0.090625
LGALS1	1.250246	7.05E-05	0.009523

CDC25C	1.249548	0.015292	0.118771
FAM49B	1.248634	0.002791	0.050538
NPEPL1	1.247876	0.017782	0.12944
C9orf116	1.246232	0.002776	0.050357
PAM16	1.245408	0.007179	0.079855
WBP4	1.245261	0.02208	0.145235
PTHLH	1.244962	0.003127	0.053222
STK3	1.244761	0.023782	0.151329
LSAMP	1.244004	0.002947	0.052024
TOP1MT	1.243114	0.001626	0.03935
PFDN2	1.242806	0.001319	0.035376
CCDC99	1.242459	0.004556	0.063343
UCHL5	1.240248	0.005282	0.067876
WFDC1	1.24014	0.010884	0.099351
SRD5A1	1.238249	0.000279	0.017692
NSMCE1	1.237164	0.003131	0.053222
PHF13	1.236249	0.029302	0.16863
PODXL	1.236149	0.002916	0.051867
FAM24B	1.235866	0.026465	0.15986
GTF2F2	1.235615	0.00435	0.061989
CEP78	1.235323	0.003004	0.052334
HMGB3	1.235214	0.033128	0.180709
CDADC1	1.234033	0.012782	0.108545
WDR3	1.233811	0.001299	0.035227
BYSL	1.233022	0.004174	0.061048
PLEKHA4	1.232901	0.026766	0.160837
HSPD1	1.232832	0.000349	0.019259
ZNRF3	1.232728	0.004619	0.063972
MCM7	1.232575	0.006005	0.072898
PRDX1	1.231934	0.001065	0.031814
VIP	1.231177	0.016092	0.122328
TTYH3	1.231036	0.015926	0.12151
GTSE1	1.230078	0.006668	0.076942
RFC2	1.22971	0.006074	0.073016
MSH2	1.227687	0.002226	0.045511
C8orf38	1.227094	0.01114	0.101008
TIMM8A	1.226544	0.019329	0.135133
PTPLAD1	1.226252	0.001449	0.037201
CNN2	1.226117	0.006503	0.075974
ODF2	1.225614	0.002615	0.048991
ELMO2	1.225195	0.022874	0.148087
MYO1B	1.224468	0.006436	0.075585
PDP1	1.223491	0.038513	0.196705
KDELC2	1.223254	0.011673	0.103363
GDF11	1.222874	0.004853	0.065103
SKP2	1.222824	0.005648	0.070468
E2F3	1.22256	0.013514	0.111316

VWA1	1.221928	0.018568	0.132589
ANTXR1	1.221302	0.000881	0.029547
COL4A2	1.221091	0.005575	0.06997
RSRC1	1.220544	0.01563	0.120341
CDK5RAP1	1.219771	0.011785	0.104026
AP1S3	1.219653	0.030738	0.173359
ZNF485	1.219496	0.004087	0.060463
CAMTA1	1.218568	0.014651	0.116177
C20orf24	1.217155	0.019394	0.135393
FANCD2	1.216352	0.001753	0.040509
PAK1IP1	1.216178	0.00231	0.046395
HABP4	1.21581	0.000424	0.021099
NUP107	1.214822	0.005591	0.070089
NR4A2	1.214365	0.022483	0.146685
NOP16	1.212931	0.003023	0.052484
DDX20	1.211634	0.015344	0.119072
CDC7	1.211625	0.008678	0.088253
HSPE1	1.209977	0.001099	0.032464
FOXM1	1.209351	0.009223	0.091266
VCAN	1.207833	0.001229	0.034276
ACACA	1.207784	0.001744	0.040395
CD300E	1.20568	0.044179	0.212254
APITD1	1.205665	0.004071	0.060389
ELOVL5	1.205455	0.002402	0.047136
PCID2	1.204983	0.006136	0.073604
na	1.204615	0.001813	0.041279
SLC3A2	1.20456	0.001138	0.033006
RBL1	1.203709	0.011199	0.101137
SET	1.203682	0.001194	0.033978
ENO1	1.203565	0.000246	0.016594
NAA10	1.202612	0.000889	0.029559
STIL	1.201613	0.047119	0.219371
CCL4	1.200793	0.000272	0.017383
USP27X	1.200408	0.009488	0.092749
EXOSC5	1.200359	0.001245	0.034391
SIGMAR1	1.198596	0.001043	0.031472
UBE2S	1.197759	0.000287	0.017929
CH25H	1.196657	0.01403	0.113634
METTL5	1.196531	0.00881	0.088859
TGS1	1.196104	0.026764	0.160837
PJA1	1.195089	0.017125	0.126833
UTP14A	1.194339	0.00194	0.042526
LRRC46	1.194299	0.031894	0.176977
CEP164	1.193355	0.005623	0.070312
SSBP1	1.193119	0.003165	0.053472
CXorf38	1.19272	0.006413	0.075471
SNRPG	1.192279	0.005973	0.072656

HSD3B7	1.192239	0.025097	0.155565
na	1.190688	0.001317	0.035376
HYAL3	1.189815	0.001049	0.031516
na	1.189043	0.017571	0.128685
PKP4	1.189003	0.004877	0.065236
STIL	1.188491	0.000375	0.019784
ZNF521	1.187337	0.021879	0.144518
RAB11FIP3	1.185833	0.004124	0.060762
SMC4	1.185426	0.011921	0.10484
GLT25D1	1.183723	0.001319	0.035376
ESPL1	1.182302	0.008707	0.088385
TMPO	1.182259	0.003594	0.05708
PDCD5	1.181964	0.003048	0.052677
ITGA9	1.181426	0.001783	0.040911
CHEK2	1.181011	0.002634	0.049127
MYRIP	1.18074	0.030626	0.173051
PBX4	1.180095	0.002855	0.051217
C19orf12	1.179763	0.01523	0.118563
CCDC86	1.179679	0.00329	0.054788
TNFRSF11B	1.179021	0.014824	0.116856
EIF3B	1.178585	0.001715	0.040279
RRP12	1.178147	0.005979	0.072705
SDCCAG3	1.1779	0.004182	0.061053
CBFB	1.176613	0.006644	0.076777
NOL6	1.176565	0.003746	0.058424
CMTM8	1.176299	0.010605	0.098097
PTPN3	1.175243	0.039894	0.200564
PERP	1.175124	0.021602	0.143242
GARS	1.174955	0.001363	0.035884
GTPBP5	1.174766	0.01984	0.13672
NDUFAF2	1.174477	0.004362	0.062046
PCID2	1.174011	0.003951	0.05954
CSGALNAC	1.173494	0.019777	0.136503
DPCD	1.17278	0.000658	0.025905
C6orf228	1.172682	0.041348	0.20437
TMEM48	1.171902	0.017099	0.126669
ASNS	1.171788	0.000638	0.025626
C9orf3	1.171256	0.00096	0.030452
CCT6A	1.170832	0.004774	0.064811
CCL3L3	1.17083	0.00061	0.025233
TRAP1	1.170738	0.002861	0.051261
PAIP1	1.169099	0.000744	0.027304
DNAJB6	1.168884	0.011189	0.101122
ZDHHC20	1.1685	0.005936	0.072284
SHMT2	1.168021	0.000231	0.016139
WDR75	1.166245	0.001553	0.038472
AHNAK2	1.165132	0.03883	0.1975

ZNF259P1	1.165014	0.007455	0.081298
PODXL2	1.164545	0.022078	0.145235
E2F6	1.164003	0.00457	0.063475
SNRPE	1.163873	0.001886	0.041804
FILIP1L	1.162493	0.003828	0.058887
NOL6	1.160037	0.022101	0.145317
RRP1B	1.159574	0.001065	0.031814
NELF	1.157675	0.005925	0.072182
CDR2	1.157578	0.027184	0.162393
AHR	1.157047	0.016608	0.124623
XPO5	1.156922	0.001869	0.04172
NUAK1	1.156495	0.000194	0.015072
FAM60A	1.155513	0.019729	0.136405
CHEK2	1.154789	0.002487	0.047855
RSL24D1	1.154465	0.047096	0.219328
SNAPC4	1.154277	0.010986	0.099961
HIST1H1B	1.153625	0.010866	0.099248
KIAA1958	1.153301	0.008027	0.084842
DEF8	1.153273	0.002377	0.046978
GRB10	1.15319	0.003698	0.057995
CSTF1	1.15317	0.046655	0.218422
PRR3	1.153092	0.00602	0.072898
POLR3E	1.151841	0.038939	0.197714
POP1	1.151243	0.001538	0.0383
CDK5	1.150362	0.000825	0.028953
ZNF511	1.15008	0.008823	0.088928
VARS	1.149799	0.025123	0.155565
TRAF3IP1	1.149765	0.020301	0.138568
H2AFX	1.149556	0.009244	0.091342
MTFR1	1.149553	0.006819	0.077932
RELT	1.149046	0.019095	0.134445
SMARCC1	1.14886	0.001791	0.041073
PFKFB3	1.148552	0.002485	0.047855
TMEM189	1.148544	0.026859	0.161126
METTL21A	1.147897	0.014742	0.116569
GLIS2	1.147357	0.032572	0.17904
DTL	1.147029	0.007379	0.080887
POLR1D	1.146652	0.007126	0.079617
HIST1H3B	1.14595	0.004039	0.06025
C20orf134	1.145551	0.046944	0.219044
CEACAM6	1.143813	0.0158	0.121009
MCM4	1.143643	7.87E-05	0.009817
TUBG1	1.143562	0.002331	0.04652
TEAD4	1.143052	0.003289	0.054788
N6AMT2	1.142588	0.000438	0.021436
PIEZO1	1.142512	0.01061	0.098097
C20orf27	1.142149	0.003385	0.055537

PDLIM7	1.141954	0.00038	0.019853
FAM64A	1.14172	0.003268	0.054542
KLF11	1.141666	0.002227	0.045511
HIST1H2AM	1.141356	0.00123	0.034276
NOP56	1.141271	0.003379	0.055466
CHRNA3	1.141223	0.011481	0.102603
MCM7	1.140529	0.004414	0.062448
H2AFZ	1.139873	0.009661	0.093565
CMTM7	1.139438	0.002236	0.045674
PPP1R14B	1.139418	0.006345	0.075014
RAN	1.139094	0.005575	0.06997
ARMC10	1.139043	0.00161	0.039156
C1orf159	1.138186	0.007959	0.084484
CENPJ	1.137858	0.012167	0.105997
MTDH	1.137706	0.019885	0.136949
IARS	1.137563	0.001425	0.036796
CYB5R2	1.136659	0.003567	0.056863
C19orf28	1.136612	0.000814	0.028716
FXN	1.136402	0.002441	0.047424
KPNA3	1.136107	0.035335	0.187524
PABPC1L	1.135094	0.000559	0.024133
MRPL3	1.134931	0.032291	0.178067
TCOF1	1.134656	0.000847	0.029272
PANX1	1.134299	0.005127	0.066816
C9orf100	1.134109	0.007961	0.084484
PRKCDBP	1.133516	0.000642	0.02564
C16orf13	1.133347	0.001029	0.031318
ZRANB3	1.133192	0.005071	0.066451
LBH	1.133119	0.003604	0.057177
SLC4A2	1.13291	0.004749	0.064706
WBP5	1.132857	0.004213	0.061169
GART	1.132536	0.010007	0.095041
TARS	1.13206	0.006992	0.078976
CACNA1C	1.13186	0.03403	0.183464
INHBA	1.131388	0.001909	0.042102
PROX1	1.131325	0.008254	0.085952
TTC25	1.131106	0.002525	0.04814
SSB	1.131019	0.004071	0.060389
PIF1	1.130679	0.022939	0.148336
LBH	1.130082	0.002573	0.04873
PSMA3	1.130074	0.00635	0.075052
AURKB	1.12993	0.004267	0.061416
CPSF3	1.129804	0.001952	0.042583
MARS	1.129392	0.008585	0.087891
SAP30	1.12902	0.015898	0.121429
ZMYM2	1.128846	0.008172	0.085655
KCNN4	1.128737	0.003668	0.057762

FBXO15	1.128642	0.009499	0.0928
SF3B3	1.128346	0.019226	0.134891
EIF1AY	1.127992	0.002741	0.049909
PCDH12	1.12778	0.006957	0.078724
TGIF1	1.127696	0.022532	0.146889
TRAF5	1.127396	0.002293	0.04628
XPOT	1.127334	0.000269	0.017382
PSMG3	1.127315	0.0026	0.048979
PRPF40A	1.126933	0.009143	0.090693
NDUFAF2	1.126702	0.003069	0.052818
MKI67	1.126471	0.01785	0.129664
TRMT6	1.125415	0.012425	0.10701
CCT3	1.124604	0.000486	0.022369
POLR2K	1.1233	0.004252	0.061313
COL9A3	1.12184	0.010068	0.095292
PAAF1	1.121427	0.005206	0.067452
ARPC5L	1.120417	0.005081	0.066522
NINL	1.120317	0.039477	0.199273
PFKM	1.1202	0.012541	0.107493
C12orf29	1.118823	0.02495	0.1552
MTAP	1.118406	0.003612	0.057177
POLA2	1.118297	0.000392	0.020183
ZBTB12	1.118056	0.022703	0.147489
HIST1H3G	1.117659	0.005199	0.067378
PAICS	1.117235	0.005372	0.068258
DHFR	1.117076	0.003845	0.058948
WDYHV1	1.116824	0.001449	0.037201
MRPL36	1.11648	0.000207	0.015348
CD55	1.115269	0.002362	0.046806
UGGT2	1.115224	0.004125	0.060762
PA2G4	1.114774	0.002478	0.04784
E2F5	1.114081	0.002224	0.045511
STRAP	1.114057	0.005396	0.068487
C20orf196	1.11387	0.001325	0.035448
NEAT1	1.11245	0.009074	0.090361
USP28	1.111609	0.017414	0.128034
RCN1	1.111435	0.003561	0.056863
P39193	1.110975	0.018905	0.133798
PIBF1	1.110838	0.010612	0.098097
HIST1H4L	1.11003	0.007242	0.080058
TCF20	1.109995	0.001076	0.032041
TPM4	1.109848	0.000915	0.029777
NUP155	1.10941	0.000361	0.01941
FOXK1	1.108977	0.033353	0.181494
PYCRL	1.107863	0.005259	0.067795
PRPS1	1.107701	0.006644	0.076777
SNRPG	1.107629	0.018821	0.133661

UBA2	1.107499	0.00831	0.086215
OLA1	1.107497	0.001108	0.032541
MKLN1	1.106873	0.004178	0.061053
PLCB4	1.106834	0.018359	0.13149
YAP1	1.106526	0.00434	0.06192
GDPD5	1.105825	0.002659	0.049196
PNO1	1.105366	0.01751	0.128527
RFC4	1.104745	0.000584	0.024719
POT1	1.104618	0.006664	0.076931
FAM100A	1.1044	0.02792	0.164648
FUT1	1.104281	0.024705	0.15436
C7orf50	1.104166	0.00174	0.040395
PALB2	1.104099	0.00252	0.048131
na	1.104027	0.007244	0.080058
RAB31	1.103628	0.007669	0.08266
POLR3H	1.103208	0.005165	0.067104
na	1.102904	0.003632	0.057335
NME4	1.102337	0.00113	0.032847
PODXL2	1.101138	0.01009	0.095394
SMS	1.100702	0.000969	0.030507
CCNI2	1.099594	0.003771	0.058594
C9orf100	1.099442	0.000163	0.0142
UTP11L	1.09941	0.001161	0.033375
TSTA3	1.09896	0.002047	0.043794
MAK16	1.09862	0.021276	0.142279
C7orf70	1.098442	0.006341	0.075
DHFR	1.098031	0.010982	0.09996
RBBP8	1.09796	0.014483	0.115485
UMPS	1.097855	0.011245	0.101331
TGM2	1.097801	0.006275	0.074532
RAN	1.097157	0.005116	0.066788
PDXK	1.096474	0.003169	0.053481
NANOS3	1.096415	0.002071	0.044137
NUP37	1.096273	0.00274	0.049909
SSX2IP	1.095982	0.03185	0.176858
C8orf59	1.095871	0.009969	0.094881
HM13	1.095647	0.002002	0.043223
COX4NB	1.095338	0.013797	0.112541
FAM189B	1.095204	0.008912	0.089341
RPF2	1.094772	0.001175	0.033654
BZW2	1.094578	0.018723	0.133123
POLE3	1.093955	0.000816	0.028724
GLA	1.093885	0.000392	0.020183
SNCAIP	1.093692	0.016958	0.126119
NTHL1	1.093262	0.006729	0.077367
KAT2A	1.093158	0.000755	0.027566
WBSCR22	1.092592	0.005352	0.068135

GNL3	1.092393	0.005745	0.071171
SLC2A3	1.091868	0.003446	0.055978
C11orf73	1.091827	0.00412	0.060755
ENST0000C	1.091797	0.006847	0.078076
CD44	1.090789	0.00872	0.088386
PLCB4	1.090696	0.003736	0.058281
HELLS	1.089349	0.003549	0.056757
TRMT112	1.089181	0.002379	0.046978
C6orf125	1.0891	0.001802	0.041206
MRPL32	1.088533	0.027918	0.164648
F3	1.088134	0.044436	0.213004
CHCHD2	1.087675	0.014133	0.113939
CHPF	1.087272	0.012406	0.106874
NAA10	1.086455	0.003956	0.059593
GRINA	1.086196	0.00353	0.056616
UBIAD1	1.085928	0.01593	0.12151
LRRC32	1.084943	0.000218	0.015622
RAMP2	1.08471	0.011687	0.103436
RPAP3	1.084459	0.001947	0.042534
OTUD6B	1.084181	0.001678	0.039785
CDCA8	1.084057	0.006767	0.077606
NKRF	1.083278	0.001192	0.033978
BCDIN3D	1.082596	0.034314	0.184401
SENP1	1.082557	0.030517	0.172697
FANCE	1.082458	0.040136	0.201072
RANBP2	1.081413	0.041816	0.205207
OTX1	1.081283	0.015693	0.120523
IPO5	1.081077	0.036593	0.191487
GPSM2	1.081069	0.00494	0.065611
ZNF438	1.080818	0.000211	0.015409
FSTL1	1.07963	0.001159	0.033343
RNF217	1.078593	0.038772	0.19737
MGMT	1.077812	0.001369	0.035952
GOLT1B	1.077121	0.023982	0.152013
LSM5	1.076721	0.010136	0.095639
HM13	1.076117	0.007107	0.079561
PRDX1	1.076062	0.00188	0.041804
MIF	1.075827	0.020151	0.137875
SLC19A1	1.075652	0.005461	0.068888
ALDH1B1	1.07514	0.002193	0.045346
IL1RN	1.074562	0.013872	0.112808
DDX31	1.074453	0.009607	0.093396
RAD1	1.07396	0.000959	0.030431
EPT1	1.071971	0.025526	0.156957
EHD3	1.071866	0.010166	0.095749
ST3GAL2	1.071768	0.017353	0.12784
PNPT1	1.071616	0.002385	0.047032

VWF	1.071608	0.011686	0.103436
HS2ST1	1.071499	0.01494	0.117242
MCTS1	1.070786	0.005807	0.071488
UBL4A	1.070755	0.001881	0.041804
CAD	1.070212	0.003668	0.057762
TAGLN	1.069951	0.00325	0.054455
SOX4	1.069897	0.006511	0.076041
MAGOHB	1.068851	0.007367	0.080832
PSMA5	1.068679	0.002629	0.049127
SYAP1	1.068653	0.008316	0.086215
B4GALT2	1.068502	0.017019	0.126408
ENST0000C	1.068305	0.012589	0.107629
C1orf56	1.068303	0.012113	0.105808
HIST1H3H	1.06789	0.002776	0.050357
NAP1L1	1.067057	0.003134	0.053222
UCHL3	1.066092	0.004698	0.064531
NLGN4Y	1.065581	0.001012	0.031051
CCNB2	1.064477	0.032258	0.178036
SMS	1.064459	0.017929	0.130041
TSPO2	1.06368	0.017079	0.126669
WARS	1.063526	0.023322	0.149527
DPY19L4	1.063328	0.007273	0.080211
CTNNBL1	1.063265	0.002663	0.049196
PRKAR1B	1.062725	0.00515	0.066973
BST2	1.062645	0.013099	0.109571
TOMM40	1.061678	0.003756	0.058515
RIOK1	1.061483	0.004645	0.064096
COPZ2	1.060612	0.004799	0.064885
NOL10	1.06038	0.005001	0.066001
CCT4	1.060111	0.002413	0.047229
HIST1H2BE	1.060072	0.022389	0.146351
DLGAP4	1.059976	0.048047	0.221758
SRD5A1	1.058159	0.008966	0.089693
DPCD	1.05776	0.003576	0.05687
AEN	1.057586	0.01573	0.120648
ANAPC1	1.056917	0.004469	0.062858
NQO1	1.0568	0.008467	0.087083
TPRKB	1.056486	0.00323	0.05424
FOXP3	1.056397	0.025297	0.15637
CIRH1A	1.056376	0.000889	0.029559
PFDN6	1.056355	0.005803	0.071488
THOC4	1.056303	0.008962	0.08968
NPHP4	1.056176	0.002715	0.049532
HSP90AB1	1.05573	0.000838	0.029214
HN1L	1.055525	0.006017	0.072898
FAM173B	1.054864	0.004322	0.061771
RCL1	1.054685	0.004742	0.064703

IKBIP	1.053547	0.018045	0.130462
TUBG2	1.053022	0.0031	0.053118
HM13	1.052832	0.006317	0.074872
NR0B2	1.052121	0.04675	0.218652
ACSL1	1.052007	0.049991	0.22642
SKA2	1.051955	0.002643	0.049127
SNRPA1	1.050792	0.017812	0.129604
ZYX	1.050699	0.012999	0.109192
SSB	1.049992	0.001588	0.038901
MYBBP1A	1.04938	0.011907	0.10477
EIF2C2	1.049327	0.001811	0.041279
ZCCHC4	1.04921	0.028618	0.166604
MME	1.049153	0.047164	0.219533
PTPN11	1.048662	0.001976	0.042806
RLIM	1.048519	0.00304	0.052587
ATR	1.048488	0.00443	0.062578
C1QTNF5	1.047927	0.002058	0.043915
LYPLA1	1.04727	0.026064	0.158357
GLT25D1	1.047247	0.001303	0.035258
C4orf48	1.047102	0.018152	0.13073
EIF3B	1.046893	0.00177	0.040765
TXNL4B	1.046751	0.008429	0.086852
SNRPG	1.046038	0.012329	0.106454
SYDE1	1.045523	0.001467	0.037484
BRAT1	1.043627	0.007047	0.079201
CYB5R2	1.043018	0.006602	0.076708
PAFAH1B2	1.042592	0.012396	0.106814
PLOD1	1.042552	0.001675	0.039785
MOCOS	1.04209	0.007087	0.07944
FAM207A	1.042033	0.000951	0.030348
CENPM	1.041446	0.010004	0.095041
HSP90AB1	1.041381	0.00086	0.0294
VMP1	1.041197	0.010466	0.097415
MCM2	1.04109	0.001367	0.035932
COL5A1	1.040579	0.028921	0.167356
C3orf39	1.038955	0.007847	0.083942
GABPB1	1.038891	0.02256	0.146985
LEPREL4	1.038563	0.035236	0.187291
ARMC10	1.038414	0.014111	0.113869
PKP4	1.038054	0.00125	0.034438
SNRPE	1.038009	0.004446	0.062667
MAGED2	1.037478	0.002525	0.04814
LATS2	1.036934	0.003816	0.058802
RILPL2	1.036796	0.005646	0.070468
SOD2	1.03622	0.010766	0.098751
RHOBTB1	1.035659	0.008243	0.085952
NOP58	1.035099	0.003902	0.059333

ATP6V1G1	1.034815	0.018168	0.130774
NAT10	1.034121	0.0091	0.090445
NUP205	1.032696	0.029797	0.17029
CCT7	1.032654	0.001996	0.043156
na	1.032449	0.000169	0.014304
TXLNG	1.031977	0.005525	0.069518
FAM122B	1.031767	0.008688	0.088336
ALG8	1.031114	0.029377	0.168855
ALG1	1.030905	0.010298	0.096494
PSMA2	1.030449	0.003539	0.056712
MNDA	1.030187	0.040156	0.201072
LPGAT1	1.02944	0.004952	0.065719
MTAP	1.02888	0.01237	0.10665
PYCR2	1.02869	0.001332	0.035505
VGLL3	1.028605	0.020846	0.140341
RBM41	1.027984	0.018007	0.130328
TOMM5	1.027567	0.004484	0.062979
ZSCAN20	1.027519	0.007661	0.082625
C1orf31	1.027503	0.021112	0.141461
NUP98	1.026856	0.046098	0.217215
RG9MTD1	1.026842	0.00373	0.058222
SELM	1.026731	0.003833	0.058887
METTL1	1.025432	0.040941	0.203146
ENOPH1	1.025048	0.021456	0.142887
ZW10	1.024377	0.031717	0.176546
HIST1H3A	1.02396	0.003163	0.053461
HGF	1.023449	0.012608	0.107733
C12orf11	1.023329	0.002104	0.044438
APTX	1.023021	0.001609	0.039156
TLCD1	1.022844	0.015606	0.120237
FANCG	1.022801	0.00086	0.0294
PSME4	1.021833	0.01928	0.135043
IER2	1.021808	0.008052	0.084896
FTSJ2	1.021263	0.008043	0.084853
TOMM40	1.020144	0.003424	0.055831
POLR1B	1.020039	0.00379	0.058639
LEPRE1	1.019489	0.002304	0.046325
ECM1	1.019474	0.022961	0.1484
CYHR1	1.019261	0.010383	0.097016
ENST00000	1.019198	0.004371	0.062146
HSPA4	1.01903	0.003879	0.059152
HES4	1.018667	0.006424	0.0755
ADO	1.018263	0.017664	0.128993
ORAI1	1.01722	0.014369	0.114978
NXN	1.016936	0.013087	0.109523
PMPCA	1.016493	0.007644	0.082579
RNF208	1.016372	0.035593	0.188391

DNAJB6	1.016321	0.027788	0.164269
C11orf58	1.016029	0.041291	0.204174
C2	1.015718	0.006225	0.074171
TUBA1C	1.015515	0.015126	0.118128
G6PD	1.015152	0.033025	0.180446
BARD1	1.014522	0.00105	0.031516
DCN	1.01433	0.000628	0.02557
PTRF	1.013837	0.004237	0.061254
NOP56	1.013296	0.02549	0.156933
ZFAND1	1.012328	0.002421	0.047247
TFDP1	1.011961	0.018526	0.132379
RPUSD4	1.011877	0.001054	0.031591
NIT2	1.011613	0.002212	0.045441
FAIM	1.011393	0.026577	0.160311
KIF26B	1.011162	0.008429	0.086852
GLRX3	1.01092	0.002792	0.050538
TIMM8A	1.010829	0.01219	0.106022
SLC19A1	1.010074	0.010909	0.099506
POLA1	1.009819	0.004095	0.060558
ARHGEF19	1.009612	0.007036	0.079167
PTPLAD1	1.00877	0.006164	0.073787
MSL3	1.008452	0.015585	0.120102
SSRP1	1.008207	0.029362	0.168808
ENST0000C	1.008024	0.005273	0.067876
CCNA2	1.007767	0.024662	0.154199
KIF3A	1.007762	0.002552	0.048447
IRAK1BP1	1.007689	0.003217	0.054038
PIK3R3	1.007582	0.003304	0.054932
EBPL	1.007351	0.023004	0.148548
CBWD5	1.007287	0.00048	0.02226
PRKRIR	1.007242	0.018589	0.132662
MIS18A	1.007083	0.006688	0.077118
METTL8	1.006792	0.009626	0.093491
FAM101B	1.006652	0.038687	0.197117
METTL5	1.006482	0.015542	0.119937
SPIN4	1.006411	0.016028	0.12201
PADI3	1.006169	0.034391	0.184636
VMO1	1.00594	0.004995	0.065986
WDR60	1.005318	0.02532	0.15637
PLIN2	1.005303	0.010308	0.096532
C16orf91	1.005083	0.046634	0.218422
C2orf18	1.004297	0.02322	0.149177
DDX11	1.003879	0.039425	0.199157
CENPM	1.003459	0.011671	0.103363
SCRN1	1.00344	0.04158	0.204858
PRR11	1.003019	0.002013	0.043372
TFDP3	1.003012	0.014215	0.114299

RPL28	1.003002	0.002285	0.04628
na	1.002722	0.012858	0.108821
PSME4	1.002509	0.015506	0.11985
PCNA	1.001374	0.012229	0.106095
GRAMD1A	1.001354	0.001947	0.042534
WDR12	1.000983	0.002955	0.052121
PRMT1	0.999758	0.009082	0.090361
RNF24	0.998747	0.009684	0.093601
PDLIM7	0.998172	0.002209	0.045441
LAP3	0.997373	0.027356	0.163082
ITGA5	0.997262	0.007081	0.079405
POLR1C	0.997081	0.006409	0.075471
CCDC85B	0.996859	0.001214	0.03419
FKTN	0.996292	0.021104	0.141448
KIF12	0.996011	0.04177	0.205207
ENY2	0.995604	0.007123	0.079606
SRPK1	0.995563	0.029345	0.168767
RGS3	0.995406	0.019741	0.136436
FAM83H	0.995375	0.03954	0.19944
ITGAV	0.995012	0.028059	0.165062
PAAF1	0.994695	0.015585	0.120102
na	0.994543	0.044486	0.213151
PRIM1	0.994328	0.012915	0.108883
TTI1	0.994126	0.007009	0.079069
STMN1	0.994014	0.010058	0.095247
DOLK	0.993711	0.029611	0.169684
HMSD	0.99298	0.00158	0.038774
UXS1	0.992536	0.028048	0.165062
MAP1A	0.99246	0.024624	0.154144
FAM108B1	0.992434	0.021118	0.141478
PHTF1	0.991936	0.03743	0.193638
PA2G4	0.991478	0.001111	0.032541
LRP11	0.991128	0.000681	0.026164
TBCD	0.990861	0.013187	0.109864
WDR36	0.99082	0.022603	0.147178
FKBP14	0.990167	0.025675	0.157446
KATNB1	0.99016	0.036158	0.190241
NSMCE2	0.989431	0.001947	0.042534
GABRE	0.989191	0.007297	0.080377
CHAC1	0.989108	0.015017	0.117523
SNRPE	0.989106	0.006713	0.077227
EIF2B3	0.988744	0.005868	0.07185
SLC31A1	0.988698	0.004526	0.063098
EFCAB11	0.988674	0.029795	0.17029
HDGF	0.987316	0.009295	0.091706
FLT1	0.986613	0.001924	0.04228
HSD17B7	0.986334	0.005261	0.067795

YTHDF1	0.986278	0.019852	0.136779
IQCB1	0.986087	0.0268	0.160973
OAS2	0.985418	0.015359	0.119093
UGGT2	0.985251	0.00451	0.063084
ORC3	0.984966	0.012694	0.108219
PTPN11	0.984847	0.001481	0.037754
RASSF8	0.984287	0.007876	0.084152
NUPL2	0.983982	0.01193	0.104893
PHF20	0.983582	0.007399	0.080929
ELK1	0.983378	0.009634	0.093495
PSMB1	0.982817	0.02423	0.152737
KRT18	0.982745	0.005327	0.068007
SRGAP2	0.981965	0.022922	0.148295
B9D1	0.981305	0.004499	0.063041
ST7	0.981128	0.029921	0.170728
ZNF154	0.980705	0.001353	0.03575
CBWD7	0.980502	0.015236	0.118577
LHFP	0.980483	0.033708	0.182369
ENST0000C	0.980327	0.005562	0.06986
CBX4	0.980298	0.001047	0.031499
NUTF2	0.98005	0.008853	0.0891
BHLHE40	0.97986	0.012577	0.107604
MXRA7	0.979151	0.019136	0.134534
DNTTIP1	0.978806	0.013742	0.112355
NMI	0.97864	0.009179	0.090935
SET	0.97766	0.003257	0.054508
TK1	0.977525	0.001871	0.04172
LSM2	0.976863	0.008895	0.089272
EFEMP2	0.97677	0.00232	0.046459
HAT1	0.976592	0.012507	0.107363
HSP90AB6I	0.976323	0.038113	0.195758
CACYBP	0.976012	0.001025	0.031262
GCFC1	0.974934	0.016557	0.124433
PGM3	0.974603	0.03882	0.1975
ZNRD1	0.974489	0.018565	0.132589
ZNF729	0.973855	0.001595	0.038953
ZNF587	0.973176	0.006022	0.072898
PTPN14	0.972586	0.007264	0.080191
SMYD3	0.971946	0.004258	0.061366
OAS2	0.971894	0.030668	0.173152
CD44	0.9716	0.001664	0.039681
ITPRIP	0.971341	0.009356	0.091987
FNDC3B	0.970842	0.032088	0.177538
S100A4	0.970499	0.007228	0.080013
BUB3	0.970151	0.001746	0.040395
NOC2L	0.969568	0.019456	0.13563
LRRC8B	0.96912	0.004704	0.064563

MNAT1	0.968319	0.028126	0.165283
C1orf198	0.968268	0.008604	0.087914
ZDHHC4	0.968267	0.005371	0.068258
HIST1H3F	0.967833	0.008643	0.088065
YEATS4	0.967382	0.009095	0.090427
RPN2	0.966967	0.020374	0.138594
MIIP	0.966933	0.000733	0.02718
DPM2	0.966818	0.007742	0.083179
na	0.966735	0.020327	0.138594
HIST1H3J	0.966724	0.006791	0.077778
C9orf64	0.966526	0.027533	0.163597
HACL1	0.966121	0.024481	0.15366
TTC27	0.96573	0.012722	0.108297
na	0.964964	0.012261	0.106142
ACTR5	0.964144	0.002475	0.047819
TM2D2	0.964119	0.030691	0.173222
MYO19	0.963911	0.011121	0.100944
SFXN3	0.963005	0.000953	0.030348
RAD51	0.962988	0.004269	0.061416
NCAPD2	0.962911	0.008565	0.087825
NCL	0.962707	0.015281	0.118744
ZCCHC6	0.962269	0.025168	0.15578
NOC3L	0.962012	0.018955	0.133941
AXIN2	0.961763	0.014894	0.117151
DCN	0.961615	0.000423	0.021093
FLT1	0.961221	0.005128	0.066816
SET	0.960677	0.001628	0.03935
EXOSC2	0.960041	0.005015	0.066086
ITGB3BP	0.959775	0.002562	0.048607
ZCCHC7	0.958673	0.006023	0.072898
MRPS12	0.956929	0.004851	0.065103
PUF60	0.956466	0.009598	0.093383
UTP20	0.955737	0.007176	0.079854
POLR1A	0.955678	0.029022	0.167594
ENST00000	0.955435	0.003992	0.059925
IPO7	0.955062	0.000873	0.029538
DDX39A	0.954921	0.001628	0.03935
MYL9	0.954886	0.01134	0.10183
CETN2	0.954407	0.006869	0.078201
ZNF668	0.953615	0.011516	0.102643
C3orf78	0.952615	0.005363	0.068213
ZNF449	0.952348	0.025836	0.157816
VAPB	0.952067	0.045407	0.215596
MYNN	0.951953	0.007199	0.079902
FARP1	0.95132	0.000994	0.030777
DDX18	0.951103	0.028114	0.165271
PPFIBP1	0.950703	0.023792	0.151329

PTPN11	0.950558	0.000929	0.030067
PPP1R14B	0.950476	0.019453	0.13563
SEC61G	0.950267	0.02084	0.140341
SRP72	0.950057	0.045647	0.216285
NES	0.949388	0.005788	0.071473
PPT1	0.949267	0.009941	0.0947
TIMM9	0.948631	0.016149	0.122572
DSE	0.948271	0.017335	0.127801
HNRNPR	0.948144	0.037027	0.192412
RFWD3	0.947646	0.007889	0.084161
FOXN2	0.947328	0.010649	0.098303
RABGGTB	0.947119	0.016623	0.124653
na	0.946937	0.036748	0.191971
SAE1	0.94654	0.024988	0.155325
PTDSS1	0.945977	0.007615	0.082497
PHC2	0.944881	0.016092	0.122328
TPM4	0.944593	0.001687	0.039823
RBP1	0.944444	0.020726	0.14016
GUCY1B3	0.944387	0.006521	0.076113
SPSB2	0.944339	0.002521	0.048131
AVEN	0.943819	0.015358	0.119093
UTP18	0.943605	0.002398	0.047123
TMEM147	0.943581	0.005309	0.068001
RAB11FIP5	0.943553	0.025973	0.158185
SNRPC	0.942991	0.022139	0.145449
RPL7	0.942827	0.0031	0.053118
PDCD11	0.94277	0.037467	0.19377
PPFIBP1	0.94272	0.003789	0.058639
FAM58BP	0.942421	0.012252	0.106095
HIST1H4L	0.942368	0.007148	0.079722
ARHGAP11	0.942335	0.007655	0.082625
SHB	0.942243	0.019555	0.1359
SHMT1	0.941737	0.01698	0.126205
ICT1	0.941265	0.007123	0.079606
SMS	0.94108	0.012215	0.106095
E2F3	0.940822	0.015841	0.121213
TBL3	0.940506	0.011558	0.102749
PFKP	0.940295	0.019068	0.134326
C17orf51	0.940281	0.011424	0.102262
WDR18	0.940057	0.009566	0.093211
NCAPD2	0.939853	0.005826	0.071645
CEBPZ	0.939624	0.019469	0.135636
TGIF1	0.939504	0.029985	0.170927
SNORA66	0.939371	0.031182	0.174712
LDHB	0.939282	0.009865	0.094517
YEATS2	0.938921	0.013845	0.112768
ARID5A	0.938066	0.006952	0.078724

ARL5B	0.937556	0.008493	0.087294
GPR172A	0.937316	0.004418	0.062459
IARS	0.937199	0.008168	0.085655
CCDC77	0.936882	0.002164	0.045072
FTSJD2	0.936688	0.019687	0.1362
GATC	0.936477	0.014256	0.114525
PTPMT1	0.936314	0.004983	0.06592
CDK4	0.935899	0.00924	0.091342
FAM58A	0.935836	0.009826	0.094354
AP1S3	0.93514	0.0172	0.127274
IFIT3	0.934701	0.047917	0.221341
MRPL17	0.934422	0.003014	0.0524
EMD	0.934271	0.010552	0.09792
HDGF	0.934193	0.002081	0.044147
CCDC43	0.933751	0.022661	0.147356
DDX31	0.933496	0.00605	0.072987
KLF2	0.933414	0.010027	0.095141
NPM1	0.933073	0.001592	0.038953
ZDHHC24	0.932771	0.042567	0.207531
ENST0000C	0.932176	0.002273	0.046244
UFM1	0.932114	0.02018	0.137908
DDX19A	0.93198	0.005227	0.0676
CTNNBIP1	0.9317	0.004931	0.065597
ARMC10	0.931129	0.003696	0.057995
MTO1	0.929982	0.019647	0.136116
ZUFSP	0.929624	0.02562	0.157361
CAMSAP2	0.928814	0.000899	0.029634
NAA16	0.928495	0.040115	0.201072
RUNX1	0.92799	0.019937	0.137111
CDKN2D	0.927971	0.045798	0.216601
LIMS1	0.926972	0.001267	0.034763
TH1L	0.926826	0.003584	0.056967
EFTUD2	0.926723	0.011993	0.105252
IFT46	0.926375	0.03728	0.19324
TOMM40	0.926311	0.000992	0.030776
SNRPD1	0.925903	0.028952	0.167451
LDHA	0.925857	0.026468	0.15986
LTV1	0.925745	0.001932	0.042423
NUDT5	0.925744	0.003641	0.057441
C16orf59	0.925288	0.015296	0.118771
B3GALNT2	0.925024	0.003546	0.056745
ENOX2	0.924776	0.010045	0.095204
NOP56	0.924082	0.011774	0.104026
MRC2	0.923943	0.02505	0.155464
TAGLN2	0.923252	0.003144	0.053307
C9orf16	0.922957	0.002157	0.045072
PRMT3	0.922944	0.000707	0.026647

CSRP2	0.922784	0.011534	0.102643
PODN	0.922594	0.049927	0.226162
C12orf75	0.922517	0.019755	0.136498
TERF1	0.921904	0.010345	0.096741
ARHGAP23	0.9218	0.01736	0.127841
GPATCH4	0.921627	0.004698	0.064531
SH3PXD2B	0.921532	0.009926	0.094699
ZNHIT2	0.921304	0.025776	0.157627
RPUSD1	0.921189	0.003682	0.057881
LAMC2	0.920882	0.013692	0.112118
GRPEL1	0.920392	0.006967	0.078779
STX2	0.920074	0.00124	0.034391
STAU1	0.920016	0.028087	0.165169
AFAP1L2	0.919835	0.046365	0.21788
WDR5	0.919766	0.039386	0.199079
ZNF670	0.919151	0.040188	0.201072
PHTF2	0.918807	0.011808	0.104151
KIF5B	0.918618	0.01619	0.122766
BPGM	0.918242	0.005005	0.066011
SNRPC	0.918238	0.043579	0.210425
SYT7	0.917671	0.012882	0.108854
LSM3	0.917494	0.003691	0.057988
NASP	0.917242	0.007623	0.082497
TRIM39-RP	0.917135	0.007247	0.080059
STIP1	0.917019	0.013089	0.109523
ZNF189	0.916972	0.017333	0.127801
MANF	0.916285	0.030441	0.172619
POLR3K	0.915768	0.023949	0.151944
MRPL13	0.915644	0.027763	0.164217
TBRG4	0.915632	0.005059	0.06643
RGS5	0.915471	0.044772	0.213916
XPO1	0.91502	0.006232	0.074171
MECOM	0.914874	0.023246	0.149198
LIG1	0.914694	0.017097	0.126669
HIST2H3D	0.914495	0.005146	0.066956
DUSP6	0.914299	0.047226	0.219628
SAC3D1	0.913949	0.007344	0.080738
na	0.9138	0.010136	0.095639
AKIP1	0.913623	0.004419	0.062459
MORF4L2	0.913186	0.015012	0.117512
RPN2	0.913157	0.007989	0.084621
GPX1	0.913152	0.002546	0.048373
LSM2	0.912545	0.016928	0.125954
YTHDF1	0.912425	0.011665	0.103343
GEN1	0.912416	0.025301	0.15637
PSMD11	0.912372	0.008212	0.085898
AP4M1	0.911578	0.001068	0.031874

ERG	0.910845	0.006806	0.077873
H2AFV	0.909984	0.031684	0.176464
NUAK1	0.909304	0.007822	0.083748
DTD1	0.909108	0.018336	0.131358
CRIP2	0.908651	0.006306	0.074781
MPG	0.90834	0.00602	0.072898
POLK	0.907867	0.006022	0.072898
ENST00000C	0.907804	0.038033	0.195479
YARS	0.907132	0.001948	0.042534
MYL6B	0.906403	0.004321	0.061771
TATDN1	0.906127	0.005841	0.071731
LRFN4	0.90555	0.016107	0.122412
NAP1L1	0.904819	0.000918	0.029813
STK32C	0.904672	0.003186	0.053691
TTL	0.904377	0.01042	0.097174
MMP2	0.903438	0.039972	0.20084
ACTN1	0.902933	0.006011	0.072898
KSR1	0.902572	0.011845	0.104419
GAS2L3	0.901787	0.008013	0.084766
SRPRB	0.900998	0.017081	0.126669
NDE1	0.900973	0.0074	0.080929
RRM1	0.900941	0.026954	0.161574
EXOSC8	0.900616	0.012333	0.106455
TTF1	0.899868	0.008246	0.085952
CHERP	0.899166	0.014128	0.113932
CTNNBL1	0.898484	0.019232	0.134903
BUB1B	0.898337	0.011233	0.101253
SERPINB6	0.897734	0.007712	0.082936
APTX	0.897142	0.007278	0.080221
KLHL5	0.897082	0.011569	0.102766
HIST1H3C	0.896961	0.009709	0.093676
ZNF598	0.896675	0.042154	0.206261
TRIM27	0.896617	0.003574	0.056863
ATP6V1C1	0.89622	0.020371	0.138594
ZNF281	0.896015	0.00854	0.087648
C9orf30	0.895756	0.001242	0.034391
CSTB	0.895427	0.004337	0.061902
PIN4	0.895425	0.017562	0.128685
KIAA1430	0.895255	0.007437	0.081198
IPO7	0.894569	0.004826	0.064951
SLC7A1	0.894308	0.006829	0.077932
G3BP1	0.893947	0.028875	0.167293
TNPO3	0.893378	0.028074	0.165121
GPX1	0.893188	0.001276	0.034854
MST4	0.893172	0.041486	0.204693
MASTL	0.893162	0.010279	0.09641
NME7	0.89292	0.018948	0.133941

ZBTB33	0.892876	0.007885	0.084154
na	0.892862	0.001219	0.034193
GPR31	0.892816	0.032496	0.178883
E2F7	0.892653	0.041479	0.204693
NOL10	0.892572	0.003428	0.055832
MOSPD1	0.892208	0.046808	0.218802
PRMT1	0.892024	0.006421	0.0755
MAP3K8	0.89201	0.013869	0.112808
MX2	0.891229	0.012137	0.105891
LYRM1	0.890776	0.007618	0.082497
OCIAD2	0.890762	0.014112	0.113869
ENST0000C	0.890216	0.002393	0.047116
POU5F1	0.889407	0.026615	0.160382
LRRCC1	0.88903	0.030919	0.173725
CLNS1A	0.888631	0.048565	0.223015
C8orf59	0.888301	0.004973	0.065872
NBEAL2	0.888	0.02016	0.137896
NPM1	0.887284	0.025724	0.157571
LMBR1	0.887249	0.027145	0.162226
SLC11A2	0.887034	0.004086	0.060463
LRRC49	0.886378	0.025393	0.15654
IPO4	0.886099	0.033576	0.182038
MPDZ	0.8859	0.003924	0.059452
CHCHD4	0.885673	0.011094	0.100785
KIF18B	0.884957	0.002082	0.044147
C1orf112	0.884833	0.024858	0.154856
TIMP3	0.884673	0.001327	0.035448
MRPL55	0.884567	0.004856	0.065103
SEC61B	0.8845	0.009701	0.093676
GUK1	0.882328	0.00405	0.060318
WDR54	0.882032	0.011227	0.101252
NOL7	0.882005	0.034951	0.186429
ICAM1	0.881724	0.007641	0.082577
OBFC2A	0.881508	0.019448	0.13563
ACD	0.881064	0.006973	0.078814
PSMD12	0.88101	0.025893	0.158035
SPRY4	0.880577	0.00998	0.09491
PTCD1	0.880182	0.037696	0.194534
ISLR	0.880116	0.004263	0.061391
BID	0.880032	0.025952	0.158163
HIST1H2AD	0.879959	0.001831	0.041455
ZNF697	0.879654	0.045155	0.215074
BZW2	0.879245	0.014681	0.116248
FXN	0.878729	0.019954	0.137111
PEA15	0.878267	0.002001	0.043223
HTATSF1	0.878053	0.04601	0.217118
TRMT2B	0.878048	0.014494	0.115523

HIST2H2AA	0.877841	0.003837	0.058887
EIF2B3	0.877774	0.012635	0.107852
PPAT	0.877232	0.011301	0.1017
BCKDK	0.877126	0.041912	0.205435
VANGL1	0.8771	0.018676	0.132972
HIST1H2AB	0.876998	0.009085	0.090361
MRPL14	0.87689	0.024203	0.15261
RPS2	0.876855	0.005402	0.068506
MRPL32	0.876844	0.015345	0.119072
DUS3L	0.87628	0.020578	0.139589
HSBP1	0.876092	0.019777	0.136503
RBM12	0.876062	0.007193	0.079902
NQO2	0.8759	0.012899	0.108883
NR2C2AP	0.875507	0.00194	0.042526
DHODH	0.875464	0.048969	0.223866
BUB3	0.874931	0.008956	0.089654
PGK1	0.874825	0.022101	0.145317
ALG2	0.874816	0.01407	0.11372
GYG1	0.874781	0.007176	0.079854
TRIM39	0.874778	0.006962	0.078743
PMPCA	0.87441	0.033977	0.183293
PRKDC	0.874249	0.004485	0.062979
ZC3H15	0.873909	0.013854	0.112778
CHAF1A	0.873808	0.006247	0.07428
UBIAD1	0.873701	0.008534	0.087611
TAB3	0.873503	0.017936	0.130067
GRWD1	0.873377	0.012973	0.109053
HN1L	0.873306	0.004818	0.064885
SCARB1	0.873176	0.024436	0.153451
HIST1H2AG	0.873098	0.012485	0.107306
DIO2	0.872617	0.01089	0.099351
IMPDH2	0.872286	0.018106	0.130631
CASP2	0.87209	0.019966	0.137111
CCDC51	0.871864	0.008628	0.087995
SPNS1	0.871606	0.044714	0.213766
PPT1	0.870939	0.003776	0.05861
C11orf48	0.870853	0.006229	0.074171
FAM165B	0.870588	0.004198	0.061078
FAM116A	0.870476	0.031391	0.175615
SS18L2	0.870468	0.006956	0.078724
RNF126	0.870401	0.028417	0.166239
TOMM6	0.870005	0.008581	0.087891
PARP9	0.869764	0.041621	0.204894
CKLF	0.86976	0.028379	0.166131
C17orf90	0.869684	0.005197	0.067377
NUP35	0.869137	0.017241	0.127415
C20orf43	0.869136	0.031645	0.176333

DARS	0.868953	0.004065	0.060389
GNA12	0.868785	0.045689	0.216307
ZNF295	0.868715	0.007893	0.084161
RSL1D1	0.868375	0.003341	0.055282
BCAP31	0.868179	0.005435	0.068717
CHCHD8	0.868144	0.028733	0.166919
ZNF621	0.867514	0.023338	0.149527
FARSA	0.867313	0.012248	0.106095
C20orf3	0.86725	0.012663	0.108065
FOXRED2	0.865929	0.006934	0.078652
TRUB2	0.865621	0.007223	0.079984
HIST1H4F	0.864849	0.007777	0.08343
CDCA7L	0.864504	0.036658	0.191706
POLE2	0.864207	0.020166	0.137896
CDC5L	0.863916	0.011715	0.10363
EIF4A3	0.863908	0.046879	0.218889
PSMB2	0.863744	0.00403	0.06023
PDAP1	0.863258	0.007965	0.084497
TBX1	0.863145	0.013963	0.113217
AZIN1	0.86275	0.012439	0.107082
MUTED	0.862367	0.006655	0.076854
COMP	0.861722	0.022447	0.146564
CCZ1	0.861161	0.011497	0.102643
CLEC2D	0.860981	0.007189	0.079902
CACYBP	0.860637	0.002579	0.048754
UBE2H	0.860198	0.02796	0.164764
LSM14B	0.859904	0.043086	0.209003
TARS2	0.859775	0.009529	0.09298
TMEM185E	0.859629	0.029398	0.168925
B9D1	0.859335	0.022334	0.146163
MRPL4	0.859083	0.023793	0.151329
C1GALT1C1	0.858983	0.043229	0.209245
TMEM55A	0.858362	0.019951	0.137111
CCDC167	0.85823	0.016483	0.124071
ATIC	0.857741	0.041804	0.205207
POLR2I	0.857725	0.005847	0.071753
GINS2	0.857536	0.010036	0.09517
CBWD5	0.857505	0.001045	0.031496
TCP1	0.857463	0.025751	0.157627
UTP6	0.857373	0.003794	0.058648
COPG2	0.857329	0.008985	0.089822
FAM206A	0.857297	0.00603	0.0729
HAUS8	0.856801	0.005141	0.066919
FERMT1	0.856325	0.004151	0.060975
JUNB	0.85579	0.01556	0.120003
LYRM4	0.855761	0.004639	0.064072
SYNCRIP	0.855498	0.009314	0.091808

POLB	0.854773	0.021577	0.143235
DROSHA	0.854614	0.038654	0.197069
MRPS2	0.854538	0.025947	0.158163
NEXN	0.853932	0.009025	0.090022
RPA2	0.853823	0.008498	0.087298
ERVMER34	0.853119	0.041219	0.203941
RNF216	0.853056	0.029381	0.168855
ESCO2	0.852802	0.028544	0.166548
RAD51D	0.852417	0.014858	0.116971
PCNA	0.852225	0.010246	0.096215
EBPL	0.852073	0.00591	0.072152
MSMO1	0.851813	0.022171	0.145566
RUVBL2	0.851694	0.004152	0.060975
MRPL47	0.850379	0.014679	0.116248
UBQLN1	0.850024	0.011402	0.10217
TCEAL4	0.849717	0.014408	0.115086
PLCB1	0.848262	0.04392	0.211553
AEBP1	0.84791	0.011719	0.10363
DPY30	0.847786	0.005488	0.069204
HIST1H2AE	0.847172	0.010774	0.098751
HRSP12	0.84696	0.009929	0.094699
SUV39H1	0.846641	0.010617	0.098115
NHP2	0.846554	0.014956	0.117242
na	0.846244	0.004003	0.060037
VCP	0.846167	0.009706	0.093676
SMYD2	0.845558	0.013219	0.109983
SLC25A19	0.84528	0.009681	0.093601
RMND1	0.845277	0.009838	0.094388
DUT	0.845134	0.008067	0.085025
ENST00000	0.844853	0.019742	0.136436
C17orf51	0.844628	0.011516	0.102643
NOC2L	0.844471	0.023133	0.148861
MRE11A	0.843705	0.019836	0.13672
SP100	0.843303	0.013284	0.110253
STOML2	0.842564	0.001352	0.03575
CDH2	0.84215	0.005924	0.072182
LTBP2	0.841618	0.030887	0.173687
SLC25A33	0.841548	0.016554	0.124433
PSMB4	0.841414	0.027975	0.164797
MYL5	0.841269	0.011054	0.10052
MRPL33	0.840668	0.043218	0.209245
CCDC109B	0.840634	0.039095	0.198203
ZMAT5	0.840624	0.025699	0.157503
CXorf26	0.840237	0.02659	0.160311
ENST00000	0.83944	0.008024	0.084842
MRPL19	0.838983	0.015643	0.12041
PLEKHG2	0.838781	0.003055	0.052731

STXBP6	0.838609	0.030478	0.172629
LRRC10B	0.838256	0.046443	0.218119
KLHL35	0.838043	0.021563	0.143225
NOP14	0.837714	0.004795	0.064885
GADD45B	0.837271	0.023327	0.149527
C7orf30	0.837043	0.006144	0.073645
RAE1	0.836832	0.019782	0.136503
CNTNAP3	0.836785	0.007263	0.080191
OBFC2B	0.836405	0.004574	0.063512
CCDC137	0.836245	0.005697	0.070789
TSR1	0.836195	0.027635	0.163883
RARS	0.836034	0.007169	0.07984
SLC2A4RG	0.835857	0.043384	0.209722
NEK4	0.834828	0.016294	0.123194
GSTP1	0.834773	0.003264	0.054535
FZD4	0.834606	0.025631	0.157361
KIAA1731	0.834518	0.020139	0.13782
CKLF	0.834247	0.033962	0.183239
CNPY3	0.83381	0.019493	0.135705
BID	0.833584	0.023102	0.148786
CLCN5	0.833419	0.01406	0.11372
FAM86B2	0.833221	0.023088	0.148755
ACPL2	0.833198	0.035844	0.18933
SMC4	0.833188	0.011447	0.102443
TRIM65	0.832734	0.026602	0.160352
ST7	0.831903	0.019082	0.134384
UBQLN4	0.831489	0.011129	0.100985
HEATR3	0.831304	0.04514	0.215053
EEFSEC	0.830793	0.014531	0.115716
DNTTIP2	0.830459	0.044465	0.213083
RBMX2	0.829484	0.010582	0.097969
na	0.829465	0.004017	0.060081
LDHA	0.829443	0.01042	0.097174
CEP250	0.828827	0.009302	0.091751
NOP14	0.828708	0.046168	0.217409
PKMYT1	0.828224	0.016463	0.124
CDK5	0.828157	0.007317	0.08052
AGRN	0.82708	0.045778	0.216601
LAMP2	0.826724	0.040016	0.200957
FHL2	0.826628	0.046877	0.218889
RGL2	0.825938	0.012251	0.106095
PTMA	0.825603	0.006825	0.077932
TCF7	0.824686	0.01428	0.114619
FAM207A	0.8242	0.008345	0.086331
TAP1	0.82401	0.018065	0.130534
SELRC1	0.823704	0.012633	0.107852
USP6NL	0.823538	0.048225	0.222248

ATAD3B	0.823465	0.0023	0.046306
TMEM147	0.823305	0.005326	0.068007
CHID1	0.823037	0.000855	0.0294
MAGEL2	0.822906	0.048248	0.222294
PEA15	0.822771	0.002193	0.045346
MLKL	0.822502	0.008462	0.08706
SHFM1	0.82239	0.018713	0.13312
TARBP1	0.821826	0.008665	0.088205
DZIP3	0.821357	0.042855	0.208359
KIF11	0.820664	0.014503	0.115567
NPM3	0.820648	0.003789	0.058639
ATP11C	0.820634	0.017416	0.128034
HPS3	0.820339	0.007653	0.082625
PSMD6	0.820307	0.002355	0.046681
PCNA	0.819945	0.034444	0.184745
TRIT1	0.81927	0.025627	0.157361
SAMD10	0.819083	0.012276	0.106245
C2orf3	0.818812	0.01829	0.131239
PWP1	0.818762	0.040049	0.200979
PSMB8	0.818643	0.012379	0.106693
MAD2L2	0.818472	0.002059	0.043915
SNRPD3	0.818302	0.035265	0.187358
PLAA	0.818239	0.026234	0.159191
VBP1	0.818085	0.018084	0.130608
MCM8	0.817985	0.04042	0.201794
CDC20	0.817789	0.007217	0.079962
STT3A	0.816929	0.021509	0.143012
ARPC1B	0.816869	0.003772	0.058594
PSMB9	0.816804	0.020087	0.137516
ANGPTL2	0.816761	0.012512	0.107363
HS3ST1	0.816399	0.039782	0.200239
C14orf80	0.815771	0.025117	0.155565
PDIA4	0.815655	0.019024	0.134228
TERF1	0.81565	0.018182	0.130835
MITD1	0.815049	0.035912	0.189571
TMEM201	0.814162	0.013657	0.111949
DLGAP5	0.814096	0.023323	0.149527
C15orf63	0.813995	0.028984	0.167542
PIGX	0.813372	0.022059	0.145231
FKTN	0.81279	0.010202	0.095963
ORC5	0.812125	0.049174	0.224386
TTC12	0.811959	0.01396	0.113217
C1orf109	0.811922	0.033202	0.180969
LIPT2	0.811792	0.013089	0.109523
AIMP1	0.811675	0.012909	0.108883
UNKL	0.810581	0.034742	0.185753
HIST1H2AM	0.809943	0.009872	0.094517

CCDC124	0.808686	0.017401	0.127993
FOPNL	0.808018	0.028197	0.165552
NASP	0.807928	0.013715	0.112188
COL15A1	0.807737	0.045956	0.217016
CCT8	0.80763	0.017836	0.129621
NAA20	0.807515	0.014751	0.116573
YWHAG	0.806982	0.039173	0.198417
PARK7	0.806378	0.004431	0.062578
SRSF3	0.806177	0.007	0.079018
JMJD6	0.806074	0.003815	0.058802
CLIC3	0.805883	0.012548	0.107493
ARHGAP18	0.805308	0.021273	0.142279
SLC15A4	0.805009	0.013267	0.110155
MAPKAPK3	0.804738	0.025848	0.157816
SLC25A14	0.804292	0.014605	0.115997
GRHL2	0.804095	0.022199	0.145573
C4orf21	0.803789	0.001421	0.036787
C5orf13	0.80358	0.029115	0.167989
POLR2G	0.803572	0.023088	0.148755
HKDC1	0.803316	0.021369	0.142666
COX7A1	0.803147	0.00387	0.05913
PSMB2	0.803104	0.001772	0.040765
USP31	0.803017	0.009142	0.090693
FERMT2	0.802937	0.00149	0.037812
WDR67	0.802734	0.0421	0.20612
IQCK	0.802638	0.033035	0.180446
FAM60A	0.802273	0.014396	0.11507
RUNX1	0.802178	0.025176	0.155799
POLR3E	0.800871	0.047395	0.220069
PPME1	0.800832	0.02959	0.169651
XXYLT1	0.800702	0.00463	0.063995
MSH5	0.800546	0.013149	0.109738
YDJC	0.800501	0.019619	0.136108
RAB23	0.80049	0.026563	0.160287
TRIM24	0.800301	0.013046	0.109332
ENST0000C	0.800282	0.04752	0.220325
HMGB1	0.800275	0.006259	0.074372
LDHB	0.800226	0.018842	0.133692
THOC3	0.800211	0.042436	0.207069
SHISA5	0.800028	0.002995	0.052324
ATAT1	0.79975	0.03679	0.192025
TYRO3	0.79962	0.021381	0.142666
TFDP2	0.79855	0.027832	0.164299
EIF6	0.798502	0.028279	0.165832
RANBP1	0.797964	0.028408	0.166239
ACTL6A	0.797905	0.033323	0.181393
NEDD1	0.79787	0.028907	0.167356

CCDC149	0.797327	0.014072	0.11372
CD82	0.797038	0.020517	0.13928
PSMB1	0.796857	0.015721	0.120648
CALHM2	0.796641	0.008621	0.087995
LYPLA1	0.796501	0.007961	0.084484
ANAPC1	0.796434	0.018516	0.132338
ABHD1	0.796422	0.025781	0.157627
UBE2I	0.796292	0.012027	0.105402
MMP14	0.795936	0.029061	0.167788
RABEPK	0.795916	0.019045	0.134291
FOXK2	0.79587	0.004191	0.061078
CCDC59	0.795825	0.01466	0.116221
NOL8	0.795319	0.011534	0.102643
HSD17B12	0.79522	0.032383	0.178436
HIST1H2BF	0.795039	0.00481	0.064885
AGAP3	0.794785	0.034156	0.183849
SEC22C	0.794525	0.022691	0.147461
TCF7	0.794354	0.026657	0.160545
SRP19	0.794261	0.010008	0.095041
C9orf142	0.793989	0.00424	0.061255
BCAP31	0.793828	0.020377	0.138594
FPGS	0.793725	0.006257	0.074367
SUB1	0.79356	0.012886	0.108854
na	0.793501	0.005982	0.072714
EIF2B5	0.793464	0.031836	0.176811
DNAJC2	0.793398	0.011206	0.101148
HSPA14	0.792953	0.025963	0.158185
CUL2	0.792463	0.011167	0.101088
HCCS	0.792462	0.044668	0.213754
RNASEH2B	0.79244	0.025439	0.156739
NINJ1	0.791652	0.014582	0.115893
PPIAL4A	0.791197	0.002331	0.04652
THOC2	0.791136	0.009994	0.09499
ZFP30	0.790441	0.039626	0.199754
RBBP7	0.790222	0.010962	0.099845
MED21	0.789935	0.036667	0.191706
HSD17B12	0.788953	0.011966	0.105052
RPA4	0.78849	0.019949	0.137111
UBE2W	0.788024	0.03383	0.182824
TRMT1	0.787498	0.004871	0.06522
MTX1	0.787481	0.0174	0.127993
APTX	0.787154	0.01326	0.11012
TNFRSF10A	0.786798	0.030675	0.173163
CCDC41	0.786245	0.004493	0.063013
LEPRE1	0.785956	0.024387	0.153254
PYCR2	0.785841	0.046006	0.217118
NAT14	0.785556	0.028558	0.166551

HIST1H3D	0.785073	0.015887	0.121429
KLC2	0.78504	0.039807	0.200306
na	0.784988	0.008883	0.089255
ALG5	0.784297	0.046223	0.217575
TNFRSF4	0.784245	0.025087	0.155535
TFDP1	0.784241	0.00449	0.062984
GEMIN5	0.784152	0.014456	0.11539
KIAA1704	0.783887	0.0326	0.179133
ACOT7	0.783472	0.009405	0.092204
SNRNP40	0.783393	0.014667	0.116246
GEMIN8	0.782875	0.017993	0.130284
UHRF1BP1	0.782015	0.042671	0.207888
TMEM191E	0.780962	0.012283	0.106274
HSPB11	0.780773	0.030167	0.171557
RAB6C	0.780237	0.038058	0.195535
KIF5B	0.779544	0.009018	0.089976
PPIA	0.779228	0.003846	0.058948
ENST0000C	0.779136	0.027681	0.163982
TUBB	0.778614	0.028948	0.167451
MRPS34	0.777943	0.011588	0.102905
NR2C1	0.77761	0.03831	0.196164
CDYL2	0.77761	0.036557	0.191331
ACAT2	0.777605	0.007955	0.084484
TAF1B	0.777261	0.027342	0.163082
CD34	0.776568	0.005711	0.070886
ISCA1	0.776454	0.003504	0.056475
RPIA	0.776211	0.019068	0.134326
SNRPD1	0.775982	0.019897	0.136974
PLEKHA1	0.775928	0.026884	0.161219
FAM125A	0.775743	0.042256	0.206674
ICA1	0.775491	0.036525	0.191323
ATAD3A	0.775302	0.021072	0.141306
TAF1D	0.774984	0.011213	0.101148
EPB41L5	0.774865	0.038237	0.196041
ERCC2	0.774571	0.015967	0.121739
POMGNT1	0.774484	0.019143	0.134534
NUDC	0.773956	0.004744	0.064703
POP7	0.773858	0.016851	0.125772
NOL9	0.773711	0.012124	0.105851
PPIA	0.773574	0.002418	0.047237
HSF1	0.77353	0.01911	0.134467
PPPDE2	0.773405	0.012008	0.105296
C9orf41	0.773315	0.038431	0.196432
SH3BP4	0.773261	0.012031	0.105402
RNF113A	0.77324	0.031921	0.176995
SMC1A	0.772995	0.012568	0.107557
FAM86B2	0.772409	0.013597	0.111657

ACTA2	0.772375	0.01009	0.095394
FDPS	0.772304	0.015458	0.119673
FAM86A	0.772053	0.020935	0.1407
MTHFD1	0.772019	0.026335	0.159543
PAEP	0.771642	0.019105	0.134467
LRRC14	0.771632	0.02272	0.147536
CCDC23	0.770839	0.022924	0.148295
WDR45L	0.770573	0.049388	0.224993
NUPL2	0.770292	0.024857	0.154856
CDC26	0.770033	0.021668	0.14359
FAM86A	0.769886	0.026393	0.15973
FERMT1	0.769769	0.015673	0.120517
PPM1G	0.769345	0.013614	0.111685
C7orf74	0.769052	0.040881	0.202914
ABCE1	0.768827	0.013229	0.109983
METTL13	0.768748	0.030576	0.172833
AP1S1	0.768667	0.019703	0.136279
DNAJB6	0.768548	0.026834	0.161032
HAUS5	0.767865	0.003392	0.055607
PTBP3	0.767859	0.010491	0.097562
CDC123	0.767809	0.003341	0.055282
CXorf56	0.767319	0.022106	0.145318
PDZK1IP1	0.767043	0.035195	0.187164
HSPA8	0.766362	0.014863	0.116971
M6PR	0.766062	0.008328	0.086292
KIAA0947	0.765992	0.036675	0.191707
SRSF7	0.765845	0.015353	0.119093
C16orf48	0.76565	0.014804	0.116812
ASCC1	0.765635	0.008373	0.086432
CCR1	0.765193	0.031567	0.176094
GTF2H5	0.765004	0.017695	0.129121
CLEC2D	0.76492	0.003465	0.056196
NFS1	0.764375	0.038316	0.196164
ATP2B3	0.764254	0.00263	0.049127
SIKE1	0.763983	0.022468	0.146642
CD248	0.763361	0.020379	0.138594
DNAH11	0.763104	0.04015	0.201072
GPCPD1	0.762805	0.021367	0.142666
RAB35	0.762582	0.006521	0.076113
CBX8	0.762013	0.005536	0.069627
NMRAL1	0.762005	0.005615	0.070237
ANP32E	0.76177	0.045741	0.216499
RPP40	0.761582	0.031433	0.175735
TAF12	0.760732	0.039719	0.200013
CERCAM	0.760665	0.018259	0.13118
ZZZ3	0.760618	0.004974	0.065872
MXRA8	0.760404	0.017366	0.127841

ATR	0.75917	0.007762	0.083347
ANAPC7	0.759151	0.020623	0.139802
EBP	0.758417	0.014227	0.114349
C11orf31	0.758398	0.027825	0.164289
LSM12	0.758349	0.012328	0.106454
ANAPC1	0.758194	0.03063	0.173051
GYG1	0.758005	0.018391	0.131634
HIST1H2AK	0.757603	0.004679	0.064387
RLIM	0.756771	0.006896	0.078399
RUNX1	0.756715	0.02932	0.168679
CDCA7	0.75666	0.010683	0.098476
CLEC11A	0.75638	0.006606	0.076708
DDX56	0.756327	0.028263	0.165799
ELK1	0.7563	0.016302	0.123202
CSTF2	0.755955	0.010035	0.09517
MLST8	0.755349	0.006307	0.074781
SKIL	0.755188	0.015923	0.12151
ANAPC10	0.754731	0.016924	0.125954
GRB7	0.754705	0.024465	0.153603
PIGU	0.754203	0.042997	0.208779
ANAPC1	0.754022	0.006938	0.078661
SF3A3	0.75365	0.020168	0.137896
IL32	0.753259	0.049197	0.224456
SLC39A4	0.753153	0.010275	0.096404
HNRNPCL1	0.75286	0.007455	0.081298
PITRM1	0.75281	0.015285	0.118744
ZSWIM1	0.752766	0.037944	0.195191
SERPINB6	0.752658	0.011355	0.10188
PSMB3	0.752465	0.016914	0.125954
RHOD	0.752236	0.009264	0.091452
C18orf10	0.751725	0.036764	0.191992
ACP1	0.751579	0.01997	0.137111
FAM71E1	0.751435	0.010744	0.098715
C20orf196	0.751123	0.014652	0.116177
BST2	0.750914	0.030549	0.172782
B3GALNT2	0.750216	0.003779	0.058632
PGD	0.749728	0.00439	0.06224
GTPBP10	0.74963	0.046605	0.21841
NUP188	0.749465	0.020389	0.138603
ANKLE2	0.749387	0.009805	0.094201
SUV39H2	0.748687	0.002205	0.045424
POLR2F	0.748404	0.047798	0.221175
C9orf114	0.748202	0.011373	0.101994
ZCCHC17	0.748141	0.00687	0.078201
DUSP7	0.748139	0.025349	0.156414
na	0.747924	0.00933	0.091842
INTS7	0.747622	0.011416	0.102218

CDR1	0.746772	0.008418	0.086823
CLPB	0.746741	0.016323	0.123304
LLPH	0.746726	0.021581	0.143235
PMS1	0.746607	0.005257	0.067795
MSTO1	0.74618	0.022382	0.146333
COL4A1	0.746128	0.002185	0.045346
DUSP23	0.746097	0.048577	0.223015
TRAF2	0.745792	0.017086	0.126669
MRPS15	0.745398	0.008008	0.084743
STAT5A	0.745324	0.033575	0.182038
NUP43	0.745207	0.00454	0.063199
ANKRD13B	0.744949	0.005454	0.068864
PDCD2	0.744501	0.021792	0.144196
HEATR2	0.744208	0.015693	0.120523
TIMM8B	0.744188	0.049372	0.224993
MRPL48	0.74406	0.019974	0.137111
C2orf43	0.742962	0.034831	0.186082
TUBB	0.74273	0.014108	0.113869
HSPA4	0.742587	0.017779	0.12944
NUDT4	0.742551	0.04374	0.210988
ANAPC1	0.741778	0.007501	0.081719
NUP85	0.741757	0.025324	0.15637
FUS	0.741027	0.026159	0.158851
CCDC59	0.741018	0.014896	0.117151
XRCC5	0.740868	0.014921	0.117211
ZNF417	0.740723	0.0275	0.163455
TAMM41	0.740578	0.043985	0.211627
SS18L1	0.740283	0.019237	0.134909
CARD14	0.739951	0.018674	0.132972
RDBP	0.739601	0.021875	0.144518
GYG1	0.739275	0.045689	0.216307
TUBA1A	0.739084	0.006219	0.074171
ANAPC13	0.738672	0.002209	0.045441
RPL27	0.738635	0.013891	0.112909
HIST1H4I	0.73855	0.009566	0.093211
PLXNB1	0.738469	0.013185	0.109864
SPRY4	0.738225	0.008337	0.086331
PLAGL2	0.737942	0.043479	0.210062
RINT1	0.737922	0.03529	0.187385
SLC25A15	0.737524	0.039397	0.199106
SUMO1	0.737227	0.019644	0.136116
ABCE1	0.736609	0.008345	0.086331
PUS1	0.736605	0.008418	0.086823
CCDC167	0.736593	0.029641	0.169748
HIST1H4H	0.736572	0.005238	0.067654
LSM14B	0.736136	0.026589	0.160311
PSMD2	0.735869	0.027031	0.161787

MRPL20	0.735783	0.014169	0.114069
IPO7	0.735634	0.014763	0.116618
CPXM1	0.735559	0.046097	0.217215
C2orf56	0.735535	0.025519	0.156943
SLC11A2	0.734704	0.040512	0.202049
SKIV2L	0.734622	0.026715	0.160721
NPM1	0.734314	0.019189	0.134743
BAZ1B	0.73425	0.037191	0.193023
CAMSAP1	0.733962	0.018639	0.132812
FXYD5	0.733382	0.037529	0.193897
CLUAP1	0.733061	0.014338	0.114883
B3GNTL1	0.732076	0.009607	0.093396
FANCL	0.731827	0.028296	0.165878
METTL8	0.731437	0.02488	0.154937
SRF	0.731408	0.030459	0.172629
C2CD2	0.731378	0.018289	0.131239
TIMM17B	0.731148	0.031009	0.174112
DAP3	0.7306	0.014485	0.115485
CNOT10	0.730195	0.013661	0.111949
MTPAP	0.730096	0.008271	0.086065
GNL3L	0.729868	0.013359	0.110628
LSM10	0.729592	0.003673	0.057791
ANP32E	0.729485	0.03534	0.187524
WDR74	0.729383	0.005033	0.066276
SMYD5	0.729294	0.006333	0.074953
RPS21	0.729235	0.02942	0.169
NOB1	0.728924	0.010703	0.098567
R3HDM1	0.728625	0.039826	0.200375
TRAF2	0.72836	0.033443	0.181702
HAUS2	0.728204	0.031565	0.176094
ZNF551	0.727075	0.024852	0.154856
RASSF1	0.726986	0.025255	0.156202
FBXL4	0.726915	0.038226	0.19604
PSMA1	0.726589	0.01885	0.133693
MRPL9	0.726257	0.013367	0.110657
FCHO1	0.725972	0.026891	0.161232
na	0.725806	0.025786	0.157627
GUK1	0.725592	0.005299	0.067974
RBMXL1	0.725355	0.048514	0.222938
PSMD10	0.725144	0.012839	0.108767
E4F1	0.725072	0.049601	0.225352
USP39	0.724997	0.037063	0.192482
C11orf1	0.724993	0.041485	0.204693
PROSER1	0.724055	0.042803	0.20822
ZEB1	0.724043	0.034977	0.186448
MRPL52	0.724001	0.025755	0.157627
CAV1	0.723931	0.003949	0.05954

ZNF32	0.723754	0.025076	0.155511
NDUFA4	0.723617	0.03029	0.172113
FHL2	0.723577	0.048967	0.223866
BCL7C	0.722893	0.013437	0.110908
HBS1L	0.722763	0.03738	0.193522
SRPX2	0.722015	0.020317	0.138593
ZNF121	0.721624	0.007017	0.079123
KIAA1797	0.721605	0.016087	0.122328
RP9	0.721449	0.013694	0.112118
EIF3I	0.721441	0.012252	0.106095
KRT8	0.720374	0.01385	0.112768
B7H6	0.72014	0.036059	0.189966
HIST2H4B	0.719218	0.023807	0.151363
ANAPC11	0.719143	0.016423	0.123787
CCNE2	0.719136	0.039652	0.199854
KCTD13	0.718889	0.01963	0.136108
UBAP2	0.718278	0.023007	0.148548
TALDO1	0.718206	0.01469	0.116294
PPIA	0.718204	0.01584	0.121213
TEAD1	0.718126	0.014005	0.113483
SOD1	0.717492	0.0318	0.176758
AMD1	0.716436	0.031723	0.176546
PARP14	0.716211	0.022348	0.146223
MTCP1NB	0.716153	0.020314	0.138593
CD46	0.716141	0.010794	0.098853
C16orf61	0.716119	0.027349	0.163082
FGFR1OP	0.716084	0.026466	0.15986
TMEM185E	0.715833	0.01323	0.109983
NPM1	0.715807	0.012838	0.108767
NBN	0.715678	0.016459	0.123999
GTF2E2	0.71498	0.039062	0.198127
EIF2AK2	0.714968	0.012032	0.105402
ISY1	0.714944	0.018783	0.133477
ARMC6	0.71278	0.012211	0.106095
AATF	0.711674	0.009713	0.093688
CANX	0.710908	0.049072	0.224078
PRPS1	0.710858	0.024131	0.152386
PHF20	0.710559	0.030375	0.17239
RPL7	0.708991	0.014067	0.11372
WDR70	0.70867	0.009938	0.094699
MUTED	0.707973	0.026026	0.158242
MLST8	0.707757	0.017585	0.128699
HIST1H2AA	0.707514	0.047219	0.219626
PPP2R1B	0.707447	0.03425	0.184204
NOL10	0.706863	0.007789	0.083473
BRI3BP	0.706745	0.015628	0.120341
CD3EAP	0.705605	0.008624	0.087995

RAD50	0.70519	0.034499	0.185012
DNAJA3	0.70491	0.045656	0.216297
ENST0000C	0.704872	0.015736	0.120648
PRRG1	0.704778	0.002627	0.049127
MAP4	0.704434	0.01925	0.134974
PML	0.704268	0.014947	0.117242
C7orf44	0.704087	0.012739	0.108327
CNPY3	0.703731	0.03284	0.179826
TUBB4B	0.70365	0.026997	0.16175
THOC7	0.702895	0.036098	0.190075
METTL23	0.702874	0.026728	0.160743
TMEM120E	0.702666	0.014371	0.114978
MEGF6	0.701347	0.041538	0.204814
BOLA2B	0.701149	0.016408	0.123724
BSCL2	0.701073	0.00982	0.09432
na	0.700263	0.028308	0.165908
CHAF1A	0.700204	0.025052	0.155464
CWC15	0.699658	0.022119	0.145377
FLJ38773	0.699455	0.020816	0.140341
DZIP1	0.699283	0.027883	0.164515
CLIC1	0.698414	0.009147	0.090703
KARS	0.698306	0.016657	0.124764
LPCAT2	0.698304	0.033819	0.18279
TMEM14B	0.698051	0.032619	0.179183
INTS8	0.69794	0.026017	0.158242
KCTD20	0.697782	0.022612	0.147183
TMEM42	0.697623	0.042425	0.207069
CENPN	0.697575	0.049747	0.225646
KLHL18	0.697116	0.04803	0.221711
SMG9	0.696841	0.010712	0.098567
PPRC1	0.696568	0.042151	0.206261
CCDC53	0.695818	0.031479	0.175934
NUCKS1	0.695799	0.02767	0.163974
SH3RF3	0.695322	0.024267	0.152874
PRR16	0.69506	0.02045	0.13898
UTP23	0.694739	0.038641	0.197062
PAFAH1B3	0.694257	0.03196	0.17712
ZNF850	0.693995	0.019622	0.136108
PAPD7	0.693962	0.036	0.189797
FAM86B2	0.693672	0.030264	0.171992
ANXA6	0.693265	0.003419	0.055793
IL15RA	0.69319	0.048321	0.222537
ZCRB1	0.692661	0.003918	0.059422
CCDC104	0.692555	0.001893	0.041878
BCL2A1	0.692405	0.031036	0.174237
PLXNA4	0.692352	0.035873	0.189454
RFC5	0.692258	0.030903	0.173691

DDX55	0.691918	0.008146	0.085501
DNAJB1	0.691719	0.048906	0.22384
PHF19	0.691138	0.015793	0.120988
DRAP1	0.690701	0.013795	0.112541
MFGE8	0.690393	0.011545	0.102687
COASY	0.689924	0.008859	0.0891
NAA50	0.689671	0.011327	0.101796
FRY	0.689429	0.01497	0.117243
KIF20B	0.689376	0.025317	0.15637
HIST1H4D	0.689116	0.012732	0.108297
PSMD2	0.689053	0.00904	0.090088
LEMD3	0.688631	0.048225	0.222248
FUNDC1	0.687198	0.027846	0.164351
AXIN1	0.686737	0.044767	0.213916
MED10	0.686562	0.015648	0.120422
LAMB2	0.686486	0.032178	0.177834
PABPC1L	0.686315	0.029857	0.170518
YIF1A	0.686033	0.032552	0.17904
C7orf25	0.685887	0.019226	0.134891
BOLA2B	0.685647	0.017819	0.129604
C7orf36	0.685635	0.036307	0.190669
ZNF597	0.685252	0.029856	0.170518
PNKP	0.684564	0.031592	0.176094
RPA2	0.684478	0.046269	0.217703
TONSL	0.68418	0.007441	0.081223
IMP4	0.683123	0.01599	0.121808
ZFP64	0.682937	0.041581	0.204858
POLR1E	0.682929	0.005695	0.070786
PDCD2	0.682858	0.006394	0.07539
SRSF3	0.68251	0.038299	0.196164
COPS6	0.682392	0.02861	0.166604
FAM91A1	0.682344	0.009919	0.094699
MRPL24	0.682205	0.041434	0.20468
FAM50A	0.682149	0.028791	0.166926
C1R	0.681921	0.042079	0.206048
ENST0000C	0.681921	0.008951	0.089626
TRIM28	0.681723	0.04061	0.202215
DDX54	0.681438	0.048895	0.223835
NUDT21	0.680327	0.032303	0.178083
WWC3	0.679907	0.020367	0.138594
na	0.67908	0.024431	0.153449
PSME2	0.678654	0.041049	0.203391
KRT18	0.678445	0.016264	0.123108
ATAD3B	0.678324	0.023792	0.151329
YTHDF1	0.677982	0.01937	0.135312
C8orf76	0.67794	0.027192	0.162393
CCDC28B	0.677646	0.007708	0.082936

PITRM1	0.677547	0.015174	0.118288
DCLRE1C	0.677084	0.042308	0.206818
RPS7	0.67708	0.016138	0.122549
FIBP	0.676762	0.021383	0.142666
MRPL15	0.676391	0.045816	0.216601
THBS1	0.676149	0.033072	0.180518
ZNF343	0.675561	0.014679	0.116248
MRPL37	0.674631	0.026634	0.16043
PTGES3	0.674442	0.036948	0.192301
HIST1H4A	0.67401	0.006067	0.07299
PHC1	0.673937	0.017828	0.129604
RPUSD3	0.673707	0.012785	0.108545
SLC1A5	0.673232	0.030422	0.172571
DTWD1	0.672624	0.013779	0.112541
C11orf31	0.672442	0.037393	0.193539
SMC1A	0.672252	0.013286	0.110253
CCDC23	0.672247	0.039434	0.199172
FAM98B	0.671555	0.041657	0.20494
THOC3	0.670995	0.043611	0.210518
TOMM20	0.670086	0.034075	0.183647
TSN	0.669842	0.03934	0.198984
ALKBH2	0.66984	0.006827	0.077932
CENPO	0.669076	0.021905	0.14456
HSD17B7	0.668907	0.013154	0.109746
ZNF367	0.668118	0.015131	0.118128
HRAS	0.667742	0.016699	0.124995
INO80B	0.667572	0.016413	0.123734
FAM122B	0.667458	0.040141	0.201072
ENST00000	0.667378	0.048066	0.221758
C2CD4D	0.667283	0.011597	0.10293
EDARADD	0.666948	0.036192	0.190332
GPI	0.666901	0.027192	0.162393
RFT1	0.666855	0.017309	0.127716
CSDA	0.666786	0.024617	0.154128
TMEM9	0.666605	0.017367	0.127841
C1orf124	0.66613	0.011562	0.102757
SSR1	0.666061	0.048503	0.222938
WDR19	0.66603	0.038936	0.197714
GIT1	0.665767	0.016391	0.123624
GNL2	0.665341	0.037213	0.193023
ATP6V1F	0.66475	0.018434	0.131912
SSR1	0.664295	0.041862	0.205309
MTAP	0.664119	0.031369	0.175527
MGAT5	0.663132	0.025487	0.156933
BUD31	0.662828	0.019659	0.136144
TUBB2A	0.662758	0.037721	0.194547
CDH5	0.662481	0.008905	0.089327

HDAC2	0.66236	0.01237	0.10665
CBX1	0.66226	0.007837	0.083856
RPS15A	0.662182	0.030492	0.172651
TXNDC9	0.662136	0.018126	0.130685
NUP43	0.662115	0.015811	0.121068
SPNS3	0.661756	0.012568	0.107557
TUBB	0.661533	0.016644	0.124725
PPAT	0.661032	0.018725	0.133123
EIF1AX	0.660541	0.035297	0.187385
MPHOSPH1	0.6603	0.023731	0.151107
PRPF6	0.660175	0.042754	0.20814
SPIN3	0.660148	0.004995	0.065986
U2AF1	0.65997	0.015182	0.118293
APOA1BP	0.659436	0.012871	0.108823
GRASP	0.65937	0.022622	0.147207
SNX8	0.658846	0.013528	0.111356
NDUFAF4	0.658764	0.012291	0.106284
HNRNPA3	0.65851	0.023416	0.149807
DNAJC14	0.658392	0.017551	0.128685
CBX5	0.658182	0.012801	0.108641
MITD1	0.657985	0.021796	0.144196
PSMC3	0.657923	0.01109	0.100785
PPIAL4A	0.657917	0.009634	0.093495
SNRPB2	0.657773	0.023998	0.152031
PEMT	0.657442	0.049464	0.225065
MEAF6	0.657224	0.02017	0.137896
BRD3	0.657133	0.036736	0.191939
MCFD2	0.657108	0.036044	0.189966
CWF19L1	0.6568	0.017274	0.127515
DDX55	0.656744	0.032826	0.179826
SNRNP48	0.655737	0.031897	0.176977
CNPY2	0.655057	0.036289	0.190635
FBL	0.654718	0.024045	0.152178
ADAM15	0.654707	0.03364	0.182209
TSNARE1	0.654348	0.037207	0.193023
XRN2	0.654149	0.033857	0.18288
C7orf70	0.653719	0.024296	0.152969
TBCCD1	0.653421	0.027514	0.163512
TAF4	0.653399	0.015819	0.121104
SAR1A	0.652836	0.006362	0.075166
HSPBP1	0.652801	0.017784	0.12944
GTF2H4	0.652702	0.045314	0.215373
ELMO2	0.652429	0.021585	0.143235
RGS12	0.652418	0.024724	0.15442
ADK	0.652208	0.03255	0.17904
VPS35	0.652093	0.032666	0.179277
DNASE2	0.651802	0.005609	0.070237

STYX	0.651367	0.016289	0.123188
PQBP1	0.651241	0.045925	0.216911
MYEOV2	0.650187	0.031624	0.176244
DHX34	0.650033	0.030892	0.173687
CBWD5	0.649691	0.009479	0.092738
FUNDC2	0.649647	0.023205	0.149109
RAD23A	0.649443	0.021501	0.14299
C5orf13	0.649375	0.023911	0.151851
STUB1	0.649271	0.021542	0.143147
NCKAP1	0.64904	0.012871	0.108823
PKM2	0.648971	0.033734	0.182448
MACROD1	0.64877	0.009507	0.09285
OGFOD1	0.648356	0.044391	0.212906
MIPEP	0.647508	0.047681	0.220859
SEPHS1	0.647224	0.014187	0.11416
ADAM15	0.646938	0.021881	0.144518
SUMO2	0.646843	0.017889	0.129812
EYA3	0.646613	0.035222	0.187248
GTF3C4	0.645996	0.020771	0.140269
RCN2	0.645818	0.031575	0.176094
ABHD11	0.645615	0.048503	0.222938
DEM1	0.645584	0.033072	0.180518
SURF2	0.645548	0.030553	0.172782
EIF4A1	0.645456	0.048835	0.223621
LOH12CR1	0.644521	0.029962	0.170855
NUBP1	0.64441	0.012493	0.107306
IFI27L2	0.644179	0.00977	0.09406
C11orf74	0.643881	0.043222	0.209245
TSSC1	0.643645	0.011848	0.104419
C21orf59	0.643399	0.017743	0.129371
MRPL30	0.64306	0.016857	0.125792
REEP6	0.641461	0.015112	0.118089
DFFA	0.641289	0.037655	0.194374
CTPS2	0.640552	0.03268	0.179313
NONO	0.640188	0.012068	0.105585
VASH2	0.640035	0.012093	0.105721
RTKN	0.639991	0.022995	0.148548
RPL21	0.639832	0.047908	0.221331
TMEM204	0.639263	0.032342	0.178269
BCCIP	0.639255	0.028746	0.166926
PPME1	0.63917	0.019066	0.134326
PSMD7	0.638682	0.034623	0.185413
SNRPB	0.638451	0.032208	0.177892
PSMD1	0.63825	0.027811	0.164271
WRAP73	0.637905	0.047264	0.219715
PHC2	0.637417	0.026364	0.159635
PHF14	0.637157	0.024601	0.154056

GLMN	0.636427	0.029191	0.168238
OSBPL8	0.635893	0.016912	0.125954
PIGA	0.635428	0.024501	0.153712
DNAJC30	0.634581	0.035007	0.186489
C16orf42	0.634552	0.01921	0.134834
PTGES2	0.634539	0.020177	0.137908
DUT	0.634315	0.03279	0.17974
ADCK5	0.634202	0.006797	0.077816
ING1	0.634202	0.025328	0.15637
CD47	0.634129	0.032169	0.177834
TRIM27	0.633949	0.008707	0.088385
MRPL37	0.633444	0.033341	0.181461
ENST00000	0.633243	0.029197	0.168238
NKAP	0.632742	0.033671	0.182284
C6orf26	0.63266	0.009396	0.092163
SNX8	0.631948	0.01426	0.114531
PHKA1	0.63191	0.025068	0.155511
CNN3	0.631101	0.029673	0.16984
GPN1	0.630993	0.013517	0.111316
ZZZ3	0.630685	0.016729	0.125139
TBCE	0.629565	0.025121	0.155565
MRPL18	0.62938	0.043358	0.209629
RPS2	0.629283	0.010582	0.097969
HIST1H2AJ	0.62893	0.01954	0.13582
RFXANK	0.628883	0.01271	0.108297
EAF1	0.628669	0.049571	0.225276
CACNA1D	0.628657	0.039682	0.199947
ATP5J2	0.628593	0.027351	0.163082
ZNF643	0.627859	0.014643	0.116177
BBX	0.627829	0.007159	0.079772
HSF1	0.627581	0.023655	0.150766
PHF19	0.626931	0.005614	0.070237
LAS1L	0.626613	0.032914	0.180129
EIF3M	0.626499	0.015204	0.118411
KLHL15	0.625242	0.008995	0.089856
CCDC104	0.624778	0.005439	0.068743
SLC41A3	0.624312	0.031093	0.174424
NIP7	0.624045	0.010664	0.098359
RNPS1	0.622977	0.035	0.186485
NRGN	0.622489	0.020849	0.140341
GSTCD	0.622464	0.038716	0.197204
AP4M1	0.621878	0.019355	0.135263
HIST1H4D	0.621203	0.041631	0.204895
ASAP1	0.621068	0.024051	0.152178
UBE2W	0.620682	0.039178	0.198417
AMZ2	0.620515	0.028526	0.166548
PLP2	0.619512	0.02414	0.152403

TXN	0.619355	0.032295	0.178067
ZNF260	0.61899	0.01358	0.111595
LLPH	0.618634	0.045255	0.215283
CEP250	0.618608	0.041031	0.203368
ODF2	0.618391	0.030639	0.173076
THOC6	0.618265	0.040827	0.202865
DCBLD1	0.61819	0.031095	0.174424
SNRPA	0.618134	0.019615	0.136108
HIST1H4K	0.617952	0.006704	0.077154
POLR2D	0.617743	0.011284	0.101627
INTS6	0.617616	0.036923	0.192289
ALOX5AP	0.617596	0.030855	0.17363
EIF2AK2	0.617169	0.009404	0.092204
TNNT3	0.616999	0.014197	0.114212
MRPS24	0.616767	0.031412	0.175674
ENST00000	0.61676	0.028478	0.166366
HIST1H1E	0.616631	0.008768	0.088638
C11orf41	0.615398	0.026446	0.15986
ZNF623	0.614496	0.01967	0.136189
RNF38	0.613881	0.013354	0.110628
BRD3	0.612919	0.04343	0.209889
TRAPPC2L	0.612688	0.023344	0.149527
UBE3C	0.612623	0.04313	0.209097
MEA1	0.612306	0.041625	0.204894
IPO11	0.611919	0.036802	0.192045
HSP90AA1	0.610269	0.031924	0.176995
EML3	0.610155	0.016904	0.125954
HEATR1	0.609927	0.015505	0.11985
BCL2L12	0.609789	0.020807	0.140339
na	0.609653	0.043999	0.211665
ESAM	0.609178	0.046559	0.218365
TNRC18	0.609157	0.037792	0.194763
RPL39	0.607787	0.023179	0.149032
HM13	0.607489	0.025472	0.156882
STARD3NL	0.607482	0.017036	0.126484
CDK5RAP2	0.607206	0.031721	0.176546
ABCF2	0.606979	0.045255	0.215283
SPTLC1	0.606834	0.038595	0.196975
COPS8	0.606641	0.008855	0.0891
ASF1B	0.606249	0.031537	0.176094
HSD17B10	0.606094	0.043598	0.210486
SLC35A2	0.605687	0.046055	0.217212
TTC4	0.605685	0.033159	0.180791
JMJD4	0.605685	0.045411	0.215596
ANAPC1	0.605037	0.021465	0.142898
CLN6	0.604677	0.037141	0.192825
PARN	0.604383	0.020014	0.137302

UBE3C	0.603632	0.032563	0.17904
BOK	0.603429	0.023036	0.148584
FKBP4	0.60317	0.014479	0.115485
TFB1M	0.602121	0.023015	0.148572
ATP11C	0.602076	0.041372	0.204428
PSMD4	0.601871	0.01191	0.10477
CTAGE5	-0.60053	0.02913	0.167999
CALY	-0.60053	0.04258	0.207534
CACNA1I	-0.60054	0.039472	0.199273
FBLN1	-0.60093	0.019961	0.137111
ARRB1	-0.60097	0.028466	0.166323
RABGAP1L	-0.60122	0.048354	0.222598
TUBAL3	-0.6013	0.048113	0.221913
PDE9A	-0.60161	0.022611	0.147183
BCAS3	-0.60175	0.017347	0.127828
TBC1D3G	-0.60206	0.036312	0.190669
na	-0.60294	0.023371	0.14961
DHDDS	-0.60315	0.028775	0.166926
RIPK3	-0.60379	0.019526	0.13582
USP3	-0.60519	0.024013	0.152099
STARD10	-0.60602	0.029627	0.169718
HERC2	-0.60732	0.031652	0.176343
CHIT1	-0.60863	0.040179	0.201072
na	-0.60886	0.04002	0.200957
PRKCD	-0.60955	0.032569	0.17904
C1orf229	-0.60996	0.034114	0.183713
CFLAR	-0.61103	0.046717	0.218557
ITGA3	-0.61214	0.032201	0.177892
PBXIP1	-0.6126	0.008717	0.088386
DEF6	-0.61312	0.016877	0.125856
KRAS	-0.61386	0.045428	0.215643
ANKRD13A	-0.61421	0.021453	0.142887
CYP21A2	-0.6148	0.036558	0.191331
GPX3	-0.61559	0.044365	0.212891
PIGS	-0.61635	0.007337	0.080684
SYTL4	-0.61684	0.014969	0.117243
BTBD7	-0.61708	0.02467	0.154199
ARHGAP32	-0.61783	0.046482	0.218214
ECH1	-0.61784	0.013556	0.111556
ENST0000C	-0.61786	0.02585	0.157816
TLE4	-0.61806	0.026676	0.1606
ELL3	-0.62074	0.046279	0.217719
na	-0.62124	0.034029	0.183464
UBA7	-0.62134	0.035525	0.188184
TBC1D20	-0.62232	0.016607	0.124623
SMPDL3B	-0.62244	0.020474	0.139098
CYTH1	-0.62263	0.026215	0.159104

CHRNA2	-0.62266	0.016145	0.122566
ZNF664-FA	-0.62275	0.008202	0.085863
ARSA	-0.62313	0.010521	0.097792
RPS6KA1	-0.6233	0.018111	0.130631
MYO5A	-0.62364	0.046954	0.219058
SUCLG2	-0.62417	0.04393	0.211573
C1orf63	-0.62499	0.028792	0.166926
PXMP2	-0.62616	0.028459	0.166316
HECTD2	-0.62662	0.033378	0.181548
P2RY8	-0.62669	0.027364	0.163082
ENHO	-0.62673	0.049695	0.225492
CIB2	-0.62675	0.007624	0.082497
SH3BP5	-0.62774	0.034093	0.183687
COQ9	-0.62804	0.030761	0.173359
CD33	-0.62819	0.034701	0.185571
PPARD	-0.63037	0.037384	0.193522
GPR155	-0.6304	0.039343	0.198984
MCOLN1	-0.63205	0.010531	0.0978
PPIP5K1	-0.63254	0.038771	0.19737
MPND	-0.63476	0.005284	0.067876
GPR18	-0.63604	0.026018	0.158242
ZNF609	-0.63632	0.015691	0.120523
C5orf63	-0.63639	0.048955	0.223866
SZT2	-0.63683	0.008737	0.088513
NUDT7	-0.63702	0.036962	0.192343
SHPRH	-0.6373	0.036271	0.190629
TNFSF12	-0.63836	0.022731	0.147558
C9orf7	-0.63853	0.019308	0.135103
SIM1	-0.63877	0.027735	0.164188
ZDHHC14	-0.6392	0.015504	0.11985
LGI4	-0.63928	0.025121	0.155565
RAB24	-0.64026	0.027766	0.164217
DHDDS	-0.64086	0.027474	0.163377
ADAP2	-0.64148	0.014945	0.117242
KCTD1	-0.64196	0.033527	0.181973
EVL	-0.64217	0.034986	0.186461
ATG16L2	-0.64287	0.02798	0.164797
BMF	-0.64301	0.025763	0.157627
PER1	-0.64404	0.027811	0.164271
MINK1	-0.64568	0.012488	0.107306
GTF2IRD2	-0.64721	0.049175	0.224386
NCKAP5	-0.64774	0.019811	0.136658
NUAK2	-0.64799	0.013915	0.113046
na	-0.64835	0.008115	0.085282
ZNF91	-0.64893	0.033802	0.18273
ZADH2	-0.649	0.024827	0.15478
QSOX1	-0.64955	0.03868	0.197117

CSF1R	-0.65	0.028544	0.166548
MPDU1	-0.65062	0.024581	0.154018
LAIR1	-0.65141	0.040506	0.202049
OR10G8	-0.65199	0.009485	0.092749
SPINT1	-0.65219	0.049926	0.226162
SIN3A	-0.65223	0.027397	0.163144
NUDT16	-0.6525	0.03785	0.194916
SMCHD1	-0.65252	0.020845	0.140341
MAP2K3	-0.65257	0.004715	0.064638
ARHGEF9	-0.65279	0.020042	0.137355
DOCK1	-0.65309	0.041455	0.20468
RAB4B	-0.65331	0.045843	0.216698
GPR162	-0.65546	0.023743	0.151157
CEACAM19	-0.65696	0.042444	0.207079
HADHB	-0.65703	0.017641	0.128952
HMBOX1	-0.65783	0.020817	0.140341
SNPH	-0.65856	0.014404	0.115086
CYP3A5	-0.65861	0.035939	0.189617
GSTM1	-0.66074	0.038035	0.195479
C14orf45	-0.66164	0.020373	0.138594
DNM2	-0.66205	0.024383	0.153254
PTGDR2	-0.66237	0.038556	0.196834
DIP2A	-0.66318	0.010067	0.095292
ASTE1	-0.66318	0.044716	0.213766
NBPF11	-0.66355	0.033077	0.180518
PPP1R13B	-0.66535	0.007158	0.079772
TAPT1	-0.66596	0.020837	0.140341
PGAP3	-0.66596	0.022202	0.145573
PHLDB1	-0.66686	0.02004	0.137355
SLC25A45	-0.66707	0.043201	0.20923
DNAJC4	-0.6682	0.011184	0.101122
WDFY4	-0.66832	0.023547	0.150361
RPS23	-0.66897	0.038626	0.197014
CLEC4M	-0.66916	0.049671	0.225473
AFF1	-0.67024	0.016739	0.125186
GOT1	-0.67046	0.04598	0.21704
PIAS2	-0.67073	0.041579	0.204858
TMEM127	-0.6709	0.003516	0.056511
na	-0.67146	0.027148	0.162226
na	-0.67231	0.012158	0.105957
ENST0000C	-0.67388	0.033595	0.182077
DENND1B	-0.67407	0.02646	0.15986
ZNF678	-0.67419	0.022937	0.148336
KAT7	-0.67528	0.04673	0.218587
TOMM40L	-0.67543	0.046145	0.21739
ENST0000C	-0.67555	0.020076	0.137478
ENST0000C	-0.67625	0.016343	0.123377

LMF1	-0.67647	0.009372	0.092046
RNPC3	-0.67671	0.006415	0.075471
MLXIP	-0.67676	0.008635	0.088006
PDGFRA	-0.67692	0.036094	0.190075
IL3RA	-0.67697	0.02009	0.137516
CRYAB	-0.67866	0.028978	0.167539
RAD52	-0.67943	0.010057	0.095247
CCDC144A	-0.67943	0.028788	0.166926
KALRN	-0.67946	0.044713	0.213766
CACNB1	-0.67998	0.006771	0.077635
KIR2DS4	-0.68073	0.033587	0.182064
ARSG	-0.68082	0.010871	0.099264
USP6	-0.68128	0.013404	0.110771
PPM1B	-0.68208	0.012859	0.108821
ENST00000	-0.68218	0.008294	0.086151
KIAA1683	-0.68249	0.020486	0.139126
TPP1	-0.68275	0.016702	0.124995
FCGRT	-0.68291	0.040345	0.201511
AGPAT4	-0.68304	0.017765	0.129413
FGD4	-0.68314	0.018887	0.13379
LEAP2	-0.68381	0.007396	0.080929
RASD2	-0.68403	0.019172	0.134685
PDE4DIP	-0.68455	0.008193	0.085812
ENST00000	-0.68612	0.021603	0.143242
FLJ40606	-0.68614	0.044903	0.214378
PLEKHB2	-0.68614	0.018002	0.130318
LY86	-0.68651	0.028541	0.166548
MED11	-0.68687	0.006927	0.078644
MICALCL	-0.68742	0.02067	0.139952
na	-0.6875	0.046934	0.219044
FBLIM1	-0.68766	0.004817	0.064885
HIGD1A	-0.68892	0.024236	0.152737
FEV	-0.68923	0.019645	0.136116
MKNK2	-0.68928	0.041685	0.205043
na	-0.68964	0.026768	0.160837
A2M	-0.68978	0.013562	0.111556
NDUFS2	-0.68993	0.01347	0.111027
HMBOX1	-0.68996	0.015887	0.121429
ZNF350	-0.69003	0.030367	0.17239
FBXO25	-0.69082	0.041844	0.205254
C16orf89	-0.69099	0.033154	0.180791
PARK2	-0.69104	0.008243	0.085952
PBXIP1	-0.69106	0.0488	0.223615
ABCA6	-0.69147	0.016499	0.124131
DUSP22	-0.69148	0.032812	0.179802
na	-0.69161	0.036284	0.190635
SLC9A9	-0.69204	0.01887	0.133699

PRKG1	-0.69228	0.034845	0.186124
MEF2D	-0.69406	0.025271	0.156272
MAST3	-0.69468	0.005236	0.067654
LGMN	-0.69471	0.013914	0.113046
ATP8B2	-0.69528	0.006633	0.076777
ACVR1C	-0.69683	0.004675	0.064351
WASL	-0.69749	0.047254	0.219698
CTNS	-0.69762	0.029845	0.170507
ZEB2	-0.69792	0.027482	0.163377
KIR3DL1	-0.69794	0.03005	0.171156
RGS3	-0.6987	0.017574	0.128685
ACVR2A	-0.69881	0.034782	0.185939
DEPDC5	-0.70001	0.004641	0.064075
RASSF5	-0.7002	0.035727	0.188924
GRIA2	-0.70067	0.046494	0.218241
CRIP1	-0.70072	0.044622	0.213593
EIF4EBP2	-0.70125	0.004892	0.065355
MPI	-0.70181	0.045814	0.216601
FIBCD1	-0.70192	0.019462	0.135636
PPP1CB	-0.70196	0.017665	0.128993
BHLHA15	-0.70273	0.017324	0.127801
PPM1A	-0.70278	0.02027	0.138437
RAB19	-0.7029	0.016037	0.122043
ENST00000	-0.70291	0.009988	0.09496
ADCY9	-0.70344	0.016571	0.124481
CNKSR3	-0.70379	0.032635	0.179212
LMOD1	-0.70437	0.040551	0.202112
FAM193B	-0.70461	0.033043	0.180446
PANK3	-0.70492	0.041724	0.205114
MBNL3	-0.70516	0.03799	0.195365
CD14	-0.70534	0.025836	0.157816
SIDT2	-0.70625	0.012855	0.108821
TTC39A	-0.7066	0.0448	0.213956
FBLN1	-0.70684	0.048483	0.222938
MYH6	-0.70718	0.005348	0.068133
RNPC3	-0.70719	0.018294	0.131239
HECTD3	-0.70825	0.013849	0.112768
SLC22A17	-0.7084	0.021681	0.14362
RBKS	-0.70847	0.048546	0.222995
SGSM2	-0.70898	0.003916	0.059422
SLC26A10	-0.71025	0.038845	0.1975
KCTD9	-0.71028	0.049073	0.224078
KIAA1217	-0.71134	0.014079	0.11372
TOM1L2	-0.71166	0.017569	0.128685
SYNJ2BP	-0.71386	0.048364	0.222615
na	-0.71397	0.034514	0.18505
PTPN6	-0.71445	0.031359	0.175527

CDH17	-0.71447	0.027608	0.163778
TPD52	-0.7148	0.038547	0.19682
FLJ26086	-0.71486	0.029925	0.170728
FOXN3	-0.71524	0.042762	0.20814
SDHA	-0.71597	0.015684	0.120523
ZNF678	-0.71683	0.008083	0.085134
BCL2L11	-0.71918	0.033508	0.181901
SLA2	-0.72032	0.038402	0.196319
PRICKLE1	-0.72069	0.048773	0.223582
CCDC159	-0.72164	0.018982	0.134018
USP6	-0.72168	0.031932	0.176995
PINK1	-0.72202	0.009725	0.093762
PLAGL1	-0.72258	0.012772	0.10853
ZNF175	-0.72314	0.0181	0.130631
KRTAP2-1	-0.72338	0.018319	0.131348
RIPK3	-0.72339	0.029968	0.170863
SH3BGRL	-0.72355	0.046866	0.218889
FEZ2	-0.72386	0.003615	0.057184
PXMP2	-0.72411	0.004977	0.065872
CAMK2N1	-0.72504	0.016793	0.125477
SOX5	-0.72553	0.046087	0.217215
SDHA	-0.72714	0.017647	0.128973
WDR73	-0.72756	0.010212	0.096034
TMEM87B	-0.7277	0.019428	0.135603
SDHA	-0.73241	0.013056	0.109347
na	-0.73242	0.025604	0.157319
SMAD3	-0.73289	0.043229	0.209245
PTPN2	-0.7331	0.0114	0.10217
RBMS3	-0.73319	0.037735	0.194562
ACACB	-0.73378	0.009384	0.092106
SLCO2B1	-0.73458	0.014153	0.113994
HNF4G	-0.73522	0.037213	0.193023
CYP2R1	-0.73551	0.022811	0.147931
TMEM98	-0.73573	0.034276	0.184256
PDLIM1	-0.73613	0.032283	0.178067
LTK	-0.73635	0.003527	0.056616
EPG5	-0.73697	0.0237	0.150964
BPTF	-0.73729	0.010094	0.095402
IGSF3	-0.73764	0.031176	0.174712
DAAM2	-0.73872	0.030046	0.171156
na	-0.73886	0.005049	0.06639
SLC25A11	-0.73916	0.006647	0.076777
FYB	-0.74015	0.045123	0.215053
TP53I3	-0.74017	0.025078	0.155511
ENST0000C	-0.74026	0.026039	0.158264
GOLPH3L	-0.74046	0.022032	0.145118
ATG4D	-0.74167	0.027565	0.163676

PPP1R13B	-0.74183	0.007348	0.08075
SGSH	-0.74297	0.009902	0.094652
SGPP2	-0.74312	0.018951	0.133941
MGLL	-0.74391	0.037913	0.195091
USP30	-0.74429	0.006479	0.07594
EYS	-0.74477	0.022874	0.148087
FARP2	-0.74622	0.010401	0.097101
CST3	-0.74681	0.005098	0.066643
FBLN1	-0.74748	0.039384	0.199079
METTL21D	-0.74758	0.012684	0.108185
ANKAR	-0.74847	0.009491	0.092749
TET2	-0.74871	0.041267	0.204086
IDH3A	-0.74907	0.020934	0.1407
MTMR10	-0.74992	0.0098	0.094201
PKD1L3	-0.75078	0.003623	0.057273
SCARA3	-0.7514	0.020694	0.140055
ACAT1	-0.75237	0.027784	0.164269
KRT74	-0.75244	0.007106	0.079561
AAK1	-0.75257	0.034036	0.183464
TLR7	-0.75275	0.020748	0.140181
GSR	-0.75326	0.005737	0.071109
SCN8A	-0.75345	0.046655	0.218422
SLC44A2	-0.75376	0.009004	0.089865
TBC1D9	-0.75406	0.042342	0.206852
ZNF678	-0.75554	0.007361	0.08082
C5orf25	-0.75603	0.039865	0.200512
WNK4	-0.75635	0.025902	0.158056
RALGAPA1	-0.75653	0.009915	0.094699
NBPF14	-0.75656	0.015991	0.121808
ACAA2	-0.75695	0.048121	0.221918
BRPF3	-0.75741	0.016236	0.122949
PSEN1	-0.75869	0.0397	0.200006
BGLAP	-0.75903	0.01907	0.134326
SORBS3	-0.75996	0.004389	0.06224
NVL	-0.76034	0.013649	0.111916
FLJ12334	-0.76136	0.041594	0.204891
CDC42SE2	-0.76183	0.006572	0.076466
FRYL	-0.76213	0.033431	0.181702
ATP6V0D1	-0.76215	0.036395	0.190927
BRPF3	-0.76215	0.003122	0.05322
CKMT1A	-0.76314	0.026585	0.160311
DST	-0.76319	0.016651	0.124752
FAM21C	-0.76359	0.034922	0.18633
KLHL28	-0.76382	0.023753	0.151189
GSDMB	-0.76387	0.041125	0.203686
P39194	-0.76408	0.023715	0.151035
ENST0000C	-0.76425	0.006554	0.076308

ECHDC2	-0.7644	0.009895	0.094644
UGT2B11	-0.76448	0.038021	0.195479
GNA13	-0.76491	0.020035	0.137355
EIF3L	-0.76508	0.00923	0.091283
CPT2	-0.76519	0.012131	0.105891
CCDC17	-0.76557	0.016601	0.124623
TMEM59	-0.76588	0.002683	0.049328
TRAPPC8	-0.76608	0.016549	0.124428
KIF18B	-0.76629	0.006321	0.074888
FAM160A1	-0.76645	0.012597	0.107667
PLEKHG6	-0.76679	0.009803	0.094201
ADORA3	-0.7671	0.014613	0.116011
na	-0.76739	0.027459	0.163355
LGALS9	-0.76746	0.021634	0.143418
NBPF15	-0.76817	0.007194	0.079902
na	-0.76853	0.006823	0.077932
CACNA2D4	-0.769	0.01214	0.105891
RHBDL2	-0.76925	0.045677	0.216307
ENST00000	-0.76986	0.004662	0.064233
CTSD	-0.77022	0.009962	0.094874
na	-0.77026	0.01048	0.097516
EXOC6B	-0.77047	0.006955	0.078724
FAM162A	-0.77051	0.049481	0.225065
MOB3A	-0.77063	0.007945	0.08445
CATSPERG	-0.77133	0.011888	0.104666
RMND5A	-0.77139	0.002984	0.052281
SCNN1A	-0.77232	0.032843	0.179826
PIP5K1B	-0.77367	0.027759	0.164217
ENST00000	-0.7746	0.005641	0.070425
RABGAP1L	-0.77492	0.020453	0.13898
C14orf182	-0.77512	0.036713	0.191845
LILRA3	-0.7759	0.02857	0.166551
HCFC2	-0.77609	0.029885	0.17059
MTMR8	-0.77626	0.011118	0.100944
LTK	-0.77746	0.011401	0.10217
FAM160A2	-0.77765	0.022282	0.145853
NBPF10	-0.77813	0.022359	0.146242
TRAF3IP2	-0.77848	0.019627	0.136108
CCBP2	-0.77976	0.021446	0.142887
EVI2A	-0.78037	0.04667	0.218459
ACO2	-0.78053	0.031691	0.176474
KIAA0317	-0.78134	0.003572	0.056863
CYTH4	-0.78189	0.022182	0.145573
ZER1	-0.78275	0.007453	0.081298
NCF1	-0.7839	0.037443	0.193676
ZSWIM6	-0.78419	0.00542	0.06861
MYO5B	-0.78451	0.008247	0.085952

UTRN	-0.78465	0.020636	0.139827
OPTN	-0.78494	0.00622	0.074171
FEM1B	-0.78507	0.024143	0.152403
DMXL2	-0.78519	0.023693	0.150951
SERINC2	-0.78521	0.028693	0.166859
VAV1	-0.7867	0.016272	0.123139
TSPAN3	-0.7868	0.015628	0.120341
GDF9	-0.78686	0.024652	0.154199
ARFGEF1	-0.78741	0.031558	0.176094
ANKRD36	-0.78783	0.019446	0.13563
na	-0.78792	0.012468	0.107244
LGALS9	-0.78854	0.015487	0.119809
SNX22	-0.78886	0.019173	0.134685
HPS1	-0.78914	0.008427	0.086852
NRXN1	-0.78925	0.023231	0.149198
SCP2	-0.78941	0.03047	0.172629
LIMK2	-0.78967	0.01687	0.125833
NCOR1	-0.79098	0.010528	0.0978
SNX29	-0.79118	0.005139	0.066914
IL10RA	-0.79147	0.016224	0.122901
MUSTN1	-0.79163	0.018162	0.130774
SLC6A8	-0.79191	0.024412	0.153353
RAB30	-0.79192	0.004438	0.062593
FAM46A	-0.79206	0.005805	0.071488
FLJ39653	-0.79294	0.039191	0.198455
SOS2	-0.79367	0.044413	0.212954
GNA11	-0.79437	0.005859	0.071797
ME2	-0.79475	0.022187	0.145573
ENST0000C	-0.79479	0.016471	0.124011
ARSG	-0.79491	0.0103	0.096494
PDE4DIP	-0.79525	0.046049	0.217211
NOTCH2	-0.79545	0.002867	0.051329
STXBP5	-0.79547	0.007118	0.079597
PDGFA	-0.79571	0.018718	0.133123
MYH11	-0.79635	0.025307	0.15637
NAALADL2	-0.79636	0.025002	0.155353
PANK3	-0.79663	0.028571	0.166551
HSBP1L1	-0.79731	0.011529	0.102643
ENST0000C	-0.79762	0.020803	0.140339
ABR	-0.79765	0.013289	0.110253
P2RX1	-0.79819	0.024674	0.154199
ACADVL	-0.79822	0.007896	0.084163
PAQR3	-0.79858	0.005851	0.07177
ZFYVE26	-0.79866	0.044703	0.213766
KIAA0141	-0.79992	0.012314	0.106407
MKNK2	-0.8005	0.024411	0.153353
STIM1	-0.80058	0.012495	0.107306

LGALS9	-0.80082	0.028448	0.166316
PGCP	-0.80139	0.034934	0.186368
XRCC6BP1	-0.80185	0.010921	0.099583
PNPO	-0.80288	0.004934	0.065597
MDM2	-0.80328	0.00423	0.061254
NAIP	-0.80437	0.047182	0.219572
LRCH3	-0.80482	0.02743	0.163216
CIRBP	-0.80536	0.009031	0.090052
NPY	-0.80559	0.02304	0.148584
KIAA1671	-0.80563	0.004942	0.065619
ANTXR2	-0.80616	0.014849	0.116947
PEX26	-0.80777	0.007981	0.084595
CCDC81	-0.80803	0.013589	0.111628
NLRP1	-0.80832	0.023807	0.151363
ACADM	-0.80904	0.002645	0.049127
CGN	-0.81032	0.01309	0.109523
ACSS2	-0.81049	0.047167	0.219533
LIMK2	-0.81087	0.040557	0.202112
CDC42EP5	-0.81234	0.006763	0.077606
SEL1L	-0.81242	0.018037	0.130435
SPECC1L	-0.81311	0.002856	0.051217
OSBPL7	-0.81343	0.005495	0.069276
OPN3	-0.81389	0.028258	0.165797
BMP2K	-0.81419	0.013168	0.109759
ACR	-0.81487	0.007557	0.082111
APOL4	-0.81541	0.036533	0.191323
DOCK5	-0.81612	0.01336	0.110628
CNST	-0.81639	0.004358	0.062041
CCDC144A	-0.81661	0.024655	0.154199
TEX9	-0.81833	0.007293	0.080362
ADRB2	-0.81848	0.011233	0.101253
TLCD2	-0.81889	0.016996	0.126267
FGD4	-0.81918	0.015424	0.119516
na	-0.81969	0.023566	0.150434
CAMK4	-0.81982	0.044129	0.212046
STX19	-0.82011	0.021211	0.141954
NDRG2	-0.82051	0.024114	0.152335
C14orf183	-0.82142	0.009852	0.094461
MTMR10	-0.82233	0.020019	0.137307
WNK4	-0.82301	0.022511	0.146832
LTBP4	-0.82309	0.003399	0.055607
ZBTB20	-0.8233	0.004592	0.063673
LGALSL	-0.82401	0.036388	0.190916
AAK1	-0.82436	0.022867	0.148087
CRAT	-0.82468	0.03679	0.192025
EFCAB4B	-0.82578	0.045134	0.215053
na	-0.8259	0.013934	0.113122

MAMDC4	-0.82729	0.018485	0.13214
OSBPL1A	-0.82804	0.021936	0.144652
NCOA1	-0.82855	0.002201	0.045424
MAPK7	-0.82873	0.008838	0.089053
BCL2L14	-0.82967	0.044085	0.211924
PP12719	-0.83007	0.009261	0.091452
PRKXP1	-0.83015	0.004318	0.061771
CXCL14	-0.83133	0.038619	0.197014
RUNX2	-0.83149	0.027701	0.164045
C15orf38	-0.83156	0.005179	0.067205
ANTXR2	-0.83273	0.008091	0.085172
CCNG1	-0.83291	0.002177	0.045271
FAM104B	-0.83307	0.007636	0.08257
CBX7	-0.83393	0.019581	0.136024
ENST00000	-0.83402	0.018898	0.133798
RAC2	-0.83491	0.046162	0.217409
MGLL	-0.83523	0.02208	0.145235
PPP1R12B	-0.83533	0.033128	0.180709
PPP2CB	-0.83596	0.025495	0.156933
STARD5	-0.83894	0.010324	0.096626
ANO7	-0.83992	0.019837	0.13672
LRRFIP2	-0.84111	0.012965	0.109053
BCL2	-0.84127	0.008624	0.087995
CCDC25	-0.84249	0.009742	0.093869
na	-0.84293	0.004052	0.060318
LRRFIP2	-0.845	0.016281	0.12318
PGM5	-0.84539	0.00804	0.084853
OR10H2	-0.84676	0.047293	0.219728
ACHE	-0.84725	0.019968	0.137111
FOXP1	-0.84837	0.00831	0.086215
C1orf21	-0.84901	0.008662	0.088202
ACOT11	-0.8492	0.00605	0.072987
ZSWIM6	-0.85192	0.0018	0.041206
CXCL12	-0.85206	0.022567	0.147003
MYO1D	-0.85209	0.013116	0.109654
GAL3ST4	-0.85373	0.01452	0.115655
TNFSF10	-0.85439	0.022421	0.146474
MAP4K1	-0.85465	0.045389	0.215579
LGALS9C	-0.85508	0.024277	0.152876
PLXNC1	-0.85561	0.017946	0.130071
SPECC1L	-0.85723	0.010419	0.097174
HMBOX1	-0.85741	0.00294	0.051992
ENST00000	-0.85779	0.022991	0.148548
VPS54	-0.8579	0.002395	0.047116
UAP1	-0.85916	0.004764	0.064806
GNPTAB	-0.85956	0.020804	0.140339
RABGAP1L	-0.86114	0.024819	0.15478

KAT6B	-0.86127	0.007321	0.080535
GPER	-0.8613	0.01874	0.133199
TOLLIP	-0.86133	0.027477	0.163377
ZNF704	-0.86151	0.01258	0.107604
MYOT	-0.86196	0.003463	0.056196
CAB39	-0.86253	0.012116	0.105808
na	-0.86273	0.006501	0.075974
FAM83E	-0.86292	0.022174	0.145566
ACACB	-0.86353	0.009242	0.091342
FAM151A	-0.86395	0.012234	0.106095
ENST00000C	-0.8641	0.024809	0.154751
SEL1L	-0.86471	0.003405	0.055676
CTNND1	-0.86481	0.00588	0.071957
MEF2C	-0.86483	0.031795	0.176758
NCF4	-0.86743	0.046608	0.21841
OXER1	-0.86782	0.04316	0.209181
PDE8A	-0.87012	0.017162	0.127082
NEIL1	-0.87036	0.025752	0.157627
ZNF346	-0.87082	0.019499	0.135705
C1orf226	-0.87153	0.012484	0.107306
PLSCR4	-0.87196	0.030482	0.172629
TTC39A	-0.87312	0.04032	0.201414
LPAR5	-0.87517	0.005075	0.066462
RERE	-0.87542	0.002849	0.051217
na	-0.87579	0.038851	0.197505
TCF7L2	-0.87586	0.011945	0.104976
KCTD9	-0.87622	0.005326	0.068007
WIPF1	-0.87651	0.020361	0.138594
SLC25A34	-0.87683	0.003591	0.057058
RASL11A	-0.87714	0.044381	0.212891
na	-0.87748	0.023348	0.149527
NANOG	-0.87877	0.014547	0.115795
MAVS	-0.87881	0.000937	0.030182
GGT6	-0.87973	0.008081	0.085134
WDR78	-0.87998	0.016133	0.122549
NBEAL1	-0.8806	0.007885	0.084154
WDR20	-0.88106	0.011852	0.104425
C20orf173	-0.88114	0.014025	0.113617
SNX1	-0.88137	0.007931	0.084343
CYTH4	-0.88164	0.010631	0.098185
ABR	-0.88238	0.002571	0.04873
na	-0.88246	0.042321	0.206841
APBB1IP	-0.88295	0.020839	0.140341
GPT	-0.88332	0.048823	0.223615
TRAF3IP3	-0.88511	0.020864	0.140414
PTP4A1	-0.88672	0.000987	0.03075
MRC1	-0.88752	0.049228	0.224566

TRAK1	-0.88777	0.000879	0.029547
PAPSS2	-0.88833	0.022679	0.147422
C5orf52	-0.8884	0.029211	0.168249
C21orf63	-0.88957	0.044338	0.21284
DNAJC13	-0.89064	0.033658	0.182245
C17orf87	-0.89107	0.035293	0.187385
SWT1	-0.89261	0.004467	0.062855
KIAA1671	-0.89411	0.006824	0.077932
SHROOM3	-0.89428	0.015709	0.120618
FXYD3	-0.89465	0.001849	0.041537
PBX1	-0.89503	0.017238	0.127415
SEMA4A	-0.89508	0.000878	0.029547
GLIPR2	-0.89533	0.003132	0.053222
ENST00000	-0.89558	0.042006	0.205787
EPB41L4B	-0.89587	0.012969	0.109053
MGAT4A	-0.89622	0.020839	0.140341
ADAMTSL1	-0.89649	0.001621	0.039322
ENST00000	-0.89661	0.015681	0.120523
DUSP26	-0.89727	0.001395	0.036361
PLD1	-0.89895	0.022444	0.146564
RAPGEFL1	-0.90137	0.019784	0.136503
FAM160A1	-0.90166	0.003365	0.055387
GRIA3	-0.90185	0.008169	0.085655
TMPRSS2	-0.902	0.039528	0.199417
RMND5A	-0.90284	0.001736	0.040395
NCF1	-0.90375	0.043979	0.211625
ENST00000	-0.90438	0.012725	0.108297
AMN	-0.9056	0.009561	0.093211
CSF1	-0.90631	0.00436	0.062046
RAP1GAP2	-0.90796	0.002672	0.049281
SH3BGRL2	-0.90829	0.012228	0.106095
MAML3	-0.90948	0.007383	0.080905
MLL5	-0.91004	0.009368	0.092046
USP37	-0.91122	0.030123	0.171469
CPT1A	-0.91156	0.00874	0.088517
CACNA1A	-0.91159	0.006487	0.075956
MCTP2	-0.9126	0.016919	0.125954
ZEB2	-0.9131	0.01523	0.118563
IQGAP2	-0.91532	0.006632	0.076777
ST6GALNA(	-0.91555	0.040219	0.201118
SNX24	-0.91591	0.019291	0.13505
na	-0.91608	0.018092	0.13062
ANKRD36B	-0.91641	0.005282	0.067876
SLC9A1	-0.917	0.026703	0.160677
CAT	-0.91713	0.028208	0.165591
MYO7B	-0.91793	0.005825	0.071645
IRF6	-0.91813	0.005724	0.071015

CTC1	-0.91897	0.006646	0.076777
FAS	-0.92168	0.049625	0.225378
TM6SF2	-0.92194	0.00727	0.080211
ULK3	-0.92306	0.017269	0.127509
PPM1L	-0.9238	0.039557	0.199497
LEAP2	-0.92446	0.036128	0.190202
AMY1C	-0.92472	0.041758	0.205207
NRIP1	-0.92572	0.010199	0.095963
CACNB2	-0.9258	0.037035	0.192412
HNF1B	-0.92629	0.00992	0.094699
FOXO1	-0.92655	0.019319	0.135103
TLN2	-0.92685	0.000941	0.030182
CYP4V2	-0.92753	0.016673	0.124831
RNASE4	-0.92791	0.039453	0.199241
HLA-DOA	-0.92804	0.024345	0.15319
SEC31B	-0.92806	0.041173	0.203829
DDX26B	-0.92809	0.007497	0.081701
MFAP4	-0.92831	0.010084	0.095381
MUC3A	-0.92908	0.008037	0.084853
STRADB	-0.93014	0.001775	0.040782
MEIS1	-0.93116	0.019264	0.135018
RELL1	-0.93166	0.012098	0.105735
NBPF10	-0.93197	0.005195	0.067377
EXOC3L4	-0.93217	0.038221	0.19604
RETN	-0.93258	0.01647	0.124011
DHRS1	-0.93357	0.000781	0.028107
ECI2	-0.93396	0.03216	0.177819
ADHFE1	-0.93483	0.047902	0.221331
TNXB	-0.93519	0.003608	0.057177
ZNF483	-0.93534	0.016605	0.124623
HRH3	-0.93566	0.010715	0.098567
KLRC4	-0.93652	0.009781	0.094103
RHOU	-0.93653	0.007756	0.083304
C10orf129	-0.93693	0.001516	0.03802
na	-0.93738	0.004038	0.06025
HLA-DRB1	-0.93747	0.038705	0.197176
MUC4	-0.93767	0.03499	0.186461
PACSIN2	-0.93852	0.005166	0.067104
SLAMF1	-0.93855	0.023567	0.150434
CHAD	-0.93909	0.030196	0.171669
ZNF575	-0.93954	0.002993	0.052322
FBXL17	-0.93978	0.034034	0.183464
APOBEC3C	-0.9416	0.01477	0.11665
ALDH6A1	-0.94172	0.026246	0.159233
INSR	-0.94212	0.005641	0.070425
ANKRD12	-0.94246	0.048633	0.223182
VLDLR	-0.94329	0.04096	0.203188

NBEAL1	-0.94342	0.002298	0.0463
PDE4DIP	-0.9439	0.003428	0.055832
CCNJL	-0.94576	0.004635	0.064041
ACER2	-0.94597	0.029642	0.169748
CPNE2	-0.94673	0.002941	0.051992
RBM47	-0.94696	0.002809	0.05071
na	-0.94708	0.004222	0.061246
C4orf19	-0.94774	0.03369	0.182345
CD200	-0.94799	0.01396	0.113217
HIGD1A	-0.94805	0.048062	0.221758
AAK1	-0.94849	0.002686	0.049328
FNBP1	-0.94907	0.022428	0.146494
HSD17B11	-0.94972	0.005839	0.071731
BDH1	-0.95139	0.000715	0.026743
VDR	-0.95269	0.00356	0.056863
ABI3	-0.95406	0.003105	0.053118
PLCG2	-0.95619	0.004006	0.060046
MUC4	-0.95761	0.023634	0.150659
C6orf136	-0.95799	0.000915	0.029777
na	-0.96153	0.001965	0.042748
COL28A1	-0.96171	0.032202	0.177892
SLC44A4	-0.96217	0.021436	0.142887
NOTO	-0.96235	0.01352	0.111316
MORN1	-0.96293	0.006935	0.078652
NOTCH2NL	-0.96367	0.009438	0.092468
na	-0.9659	0.00923	0.091283
TMEM106/	-0.96742	0.016566	0.124474
CDC14A	-0.96743	0.033568	0.182038
KIAA1737	-0.96744	0.006588	0.076623
ARHGAP24	-0.96745	0.023078	0.148747
B4GALNT3	-0.96901	0.003698	0.057995
CYP3A7	-0.96903	0.034094	0.183687
ABCC3	-0.96987	0.002481	0.047855
C6orf174	-0.97051	0.045604	0.216159
FCRL1	-0.97064	0.020748	0.140181
IKZF3	-0.97067	0.010529	0.0978
GAL3ST2	-0.97102	0.008104	0.085255
PDE4DIP	-0.9732	0.004129	0.060786
AMACR	-0.97478	0.009682	0.093601
CPNE5	-0.97533	0.004203	0.061095
SQRDL	-0.9765	0.038622	0.197014
N4BP2L1	-0.97734	0.017681	0.129078
MOCS1	-0.97746	0.019618	0.136108
ZNF844	-0.97796	0.017877	0.129781
TMEM131	-0.97889	0.005261	0.067795
na	-0.98001	0.005058	0.06643
AAK1	-0.98009	0.002249	0.045906

MXD1	-0.98077	0.022723	0.147536
STYK1	-0.98102	0.048486	0.222938
C1QA	-0.98138	0.049883	0.226085
BMP6	-0.98212	0.002335	0.04652
C14orf43	-0.98257	0.017332	0.127801
C15orf17	-0.98409	0.004739	0.064703
SYNE2	-0.98507	0.018216	0.130969
LSP1	-0.98537	0.004976	0.065872
PJA2	-0.98636	0.042777	0.208158
FAM18B2-(	-0.98644	0.030556	0.172782
ETHE1	-0.98674	0.010109	0.095512
PAPLN	-0.98704	0.002759	0.05013
TNFSF10	-0.98705	0.045775	0.216601
VAMP2	-0.98732	0.000806	0.028548
CKMT1A	-0.98756	0.012439	0.107082
MTMR3	-0.98766	0.002004	0.043223
FOXN3	-0.98776	0.003167	0.05348
ATP11B	-0.98795	0.01552	0.119876
CYP3A7	-0.98903	0.046236	0.217575
CDKL1	-0.98968	0.000746	0.027304
CHCHD10	-0.99014	0.036497	0.191224
ZNF839	-0.99112	0.019389	0.135387
TRPV1	-0.99198	0.008459	0.087059
ENST00000	-0.99337	0.005727	0.071026
MOB3B	-0.9938	0.002425	0.047251
AFF1	-0.99405	0.000355	0.01927
OBSCN	-0.99428	0.004775	0.064811
MFSD6L	-0.99495	0.005338	0.06805
PPP1R14D	-0.99529	0.036981	0.19238
LTBP4	-0.99597	0.019596	0.136069
na	-0.99623	0.00621	0.074134
SRGAP3	-0.99701	0.020072	0.137476
na	-0.99758	0.001042	0.031472
TRIM69	-0.99845	0.048235	0.222264
MUC4	-0.99921	0.029742	0.170117
MCTP2	-0.99971	0.005261	0.067795
RNPC3	-1.00161	0.012335	0.106455
DGKA	-1.00169	0.014514	0.115635
TP53INP1	-1.00241	0.021466	0.142898
PON3	-1.00316	0.038305	0.196164
NHSL1	-1.00331	0.005675	0.070659
TMC4	-1.00461	0.002176	0.045269
MAVS	-1.00469	0.000752	0.027478
FARP2	-1.00634	0.019777	0.136503
STRADB	-1.00849	0.000907	0.029719
LINS	-1.00877	0.018925	0.133864
MUC3	-1.00898	0.004817	0.064885

CCNG1	-1.00968	0.022028	0.145118
PRKAR2B	-1.01068	0.034441	0.184745
MOB3B	-1.01105	0.004732	0.06467
MS4A14	-1.01139	0.001961	0.042728
CRIP1	-1.01329	0.027566	0.163676
LRRC19	-1.01341	0.003946	0.05954
STBD1	-1.01351	0.035373	0.187609
CCL14	-1.01431	0.026419	0.159821
SPATA24	-1.01479	0.005097	0.066643
NOTCH2NL	-1.01487	0.008985	0.089822
ITGAL	-1.01525	0.020785	0.140335
CLYBL	-1.01525	0.01182	0.104224
ABCC3	-1.01559	0.002507	0.047996
TRAF3IP2	-1.01575	0.001663	0.039681
FAM63A	-1.01868	0.022205	0.145573
CD27	-1.01894	0.015537	0.119923
PDE4D	-1.02041	0.012997	0.109192
DOCK8	-1.02234	0.00521	0.067475
SLC22A5	-1.0226	0.030829	0.173623
AMY1C	-1.02309	0.017587	0.128699
MYZAP	-1.02316	0.010573	0.097969
MAVS	-1.0233	0.001726	0.04038
TLR9	-1.02367	0.007487	0.081621
MEI1	-1.02369	0.024907	0.155018
PDK4	-1.02376	0.012865	0.108823
CSF1	-1.02439	0.00311	0.053118
ZNF169	-1.02508	0.004925	0.065588
PRDX6	-1.0254	0.000183	0.014841
PRSS12	-1.026	0.0063	0.074743
AVIL	-1.02617	0.02392	0.151877
HVCN1	-1.02625	0.019973	0.137111
ZSCAN30	-1.02769	0.002291	0.04628
MAML3	-1.02779	0.003921	0.059434
OCR1	-1.02891	0.018961	0.13395
IL10RB	-1.02991	0.029654	0.169786
ARHGAP15	-1.03042	0.034888	0.186298
na	-1.03053	0.004931	0.065597
PDE4DIP	-1.03173	0.009581	0.093297
CYTIP	-1.03224	0.019453	0.13563
PAG1	-1.03289	0.006999	0.079018
NBPF1	-1.0332	0.003521	0.056566
MAPK10	-1.03503	0.03829	0.196164
TARP	-1.03578	0.021762	0.144071
RASGRP3	-1.03696	0.0346	0.185367
CCDC152	-1.03754	0.04954	0.225165
AGFG2	-1.03782	0.007353	0.080784
ENHO	-1.03957	0.004497	0.063037

APBB1IP	-1.04102	0.024054	0.152178
ATP8A1	-1.04131	0.02272	0.147536
LY9	-1.04188	0.020708	0.140092
NOTCH2NL	-1.04477	0.000676	0.026073
IGSF3	-1.04555	0.007114	0.079587
PIK3R6	-1.04561	0.004188	0.061077
CCNG2	-1.0464	0.047849	0.221215
OAF	-1.04658	0.000178	0.014698
FAM84A	-1.04678	0.016771	0.125368
CITED2	-1.04705	0.006909	0.078463
EDNRB	-1.04753	0.016883	0.125868
AKAP9	-1.04766	0.024995	0.155341
IMPA1	-1.0484	0.006193	0.074028
GRAMD4	-1.04851	0.012963	0.109053
CNNM2	-1.04869	0.000643	0.025654
ANK2	-1.04909	0.013682	0.112074
STON2	-1.05228	0.003152	0.053349
ILDR1	-1.0536	0.001415	0.036692
GLIPR2	-1.05408	0.000183	0.014841
EFCAB4B	-1.05422	0.01589	0.121429
LILRB1	-1.05616	0.016797	0.125477
TNFRSF13C	-1.05633	0.040879	0.202914
C14orf159	-1.05678	0.001365	0.03591
SESN2	-1.05791	0.004808	0.064885
FKBP1B	-1.05834	0.027412	0.163144
C5orf41	-1.05881	0.033508	0.181901
CDH19	-1.05887	0.003377	0.055466
GNRH1	-1.06377	0.000628	0.02557
PIK3CG	-1.06423	0.01863	0.132812
na	-1.06455	0.002092	0.044217
CCDC144A	-1.06495	0.029834	0.170474
CD8A	-1.06497	0.046184	0.217445
CCR10	-1.06618	0.023485	0.15022
NEURL	-1.06713	0.00451	0.063084
LGALS4	-1.06734	0.006016	0.072898
na	-1.0675	0.03424	0.184178
UQCR10	-1.06844	0.003444	0.055978
UGP2	-1.07077	0.017235	0.127415
GPD1L	-1.07179	0.008236	0.085952
MASP2	-1.0722	0.004327	0.061782
TXNIP	-1.07363	0.003911	0.0594
ENST00000	-1.07367	0.003369	0.055415
TFCP2L1	-1.07393	0.037005	0.192388
PDE3A	-1.07489	0.021317	0.142494
SLC18A2	-1.07505	0.004451	0.062685
GHR	-1.07812	0.037799	0.194772
C5orf63	-1.07884	0.002661	0.049196

na	-1.07886	0.027126	0.162226
C1QB	-1.07925	0.024244	0.152756
FBLIM1	-1.08076	0.007932	0.084343
ANKRD36	-1.08147	0.013798	0.112541
RGS9	-1.08267	0.00208	0.044147
C1orf210	-1.08275	0.002142	0.045012
IKZF1	-1.08393	0.046093	0.217215
na	-1.08561	0.007037	0.079167
ASAP3	-1.08587	0.002092	0.044217
TRANK1	-1.08731	0.033947	0.183214
APOBEC3F	-1.08805	0.022225	0.145674
SYCP3	-1.08829	0.000522	0.023351
P2RX4	-1.08875	0.000257	0.017125
RHBDL2	-1.09001	0.015905	0.121429
SLC39A5	-1.09065	0.005317	0.068007
CLIC5	-1.09075	0.013119	0.109654
PPARGC1B	-1.09288	0.016996	0.126267
TCP11L1	-1.09318	0.001484	0.037775
SLC41A2	-1.09349	0.004687	0.06444
MIER3	-1.09423	0.014407	0.115086
RBM47	-1.09511	0.008802	0.088859
GNPTAB	-1.09553	0.000574	0.024489
na	-1.09588	0.046307	0.217757
ATP10B	-1.09681	0.012716	0.108297
TMEM14E	-1.09719	0.018296	0.131239
FAIM3	-1.09994	0.040049	0.200979
C21orf62	-1.10081	0.007891	0.084161
ADAMTSL1	-1.10083	0.000756	0.027577
APPL2	-1.10181	0.001033	0.031318
GSN	-1.10196	0.011565	0.102762
KIAA1370	-1.10242	0.006428	0.075522
GCNT2	-1.10362	0.008772	0.088656
AHNAK	-1.10364	0.004158	0.060975
CIDEB	-1.10375	0.004489	0.062984
LINC00483	-1.10407	0.016223	0.122901
C14orf159	-1.10507	0.000372	0.019782
NIPAL1	-1.10557	0.008301	0.086192
TCL1A	-1.10681	0.010579	0.097969
KIF16B	-1.10827	0.007094	0.07948
NIPAL2	-1.10853	0.001628	0.03935
MUSK	-1.10925	0.003938	0.059515
COX6B2	-1.10942	0.012612	0.107742
HOXD8	-1.11074	0.009575	0.093273
C14orf182	-1.1108	0.000641	0.02564
TBC1D10C	-1.11097	0.013477	0.111048
NPTN	-1.11161	0.008249	0.085952
na	-1.11219	0.042417	0.207067

ANTXR2	-1.11278	0.00219	0.045346
ENST0000C	-1.11363	0.005856	0.071793
RAB37	-1.11365	0.009517	0.092888
MS4A6A	-1.11588	0.01784	0.129624
GUSBP1	-1.11684	0.012079	0.105657
TRIM36	-1.11818	0.045249	0.215283
KCNN3	-1.11873	0.011211	0.101148
BMP2K	-1.11888	0.000731	0.027163
WIPF1	-1.11936	0.045542	0.21597
ANK3	-1.11976	0.011318	0.101796
FAS	-1.1203	0.040256	0.201214
HGD	-1.12033	0.029918	0.170728
DEFB1	-1.12732	0.010051	0.095239
PROM2	-1.12826	0.037735	0.194562
DOCK2	-1.12869	0.00496	0.065799
CAMK2D	-1.13103	0.01321	0.109975
GSTM2	-1.13338	0.005905	0.072149
CHST6	-1.1348	0.003881	0.059154
PTPRR	-1.13579	0.040204	0.201072
na	-1.13583	0.006069	0.07299
SNX29	-1.13643	0.001741	0.040395
MIER3	-1.13838	0.015284	0.118744
na	-1.13849	0.037714	0.194544
TPSG1	-1.13915	0.005374	0.068263
ALPI	-1.14079	0.003363	0.055387
na	-1.14162	0.013844	0.112768
AHNAK	-1.14218	0.007424	0.08111
na	-1.1425	0.034665	0.185491
LPCAT4	-1.14255	0.01072	0.098573
MS4A6A	-1.14345	0.024962	0.155246
CD209	-1.14396	0.002971	0.052177
MICAL3	-1.14435	0.012169	0.105997
CD200	-1.1444	0.014585	0.115893
KLHL6	-1.14466	0.006068	0.07299
TTLL7	-1.1448	0.027321	0.163053
PDE4DIP	-1.14528	0.001341	0.035613
SCML4	-1.1462	0.002922	0.051896
HLA-DPA1	-1.14739	0.023385	0.149668
VAMP2	-1.14829	0.004875	0.065236
C5orf30	-1.1483	0.007147	0.079722
STAP2	-1.14838	0.005738	0.071109
SHD	-1.1489	0.021549	0.143165
VSIG7	-1.1508	0.041027	0.203368
TJP3	-1.15148	0.002217	0.045511
JAM2	-1.15194	0.023001	0.148548
NCF1	-1.15339	0.012965	0.109053
FRRS1	-1.15681	0.018954	0.133941

FNIP2	-1.15715	0.000448	0.021666
na	-1.15912	0.003333	0.055273
PARVG	-1.15964	0.008602	0.087914
CCDC69	-1.16002	0.013204	0.109958
FNBP1	-1.16142	0.003461	0.056187
NEURL1B	-1.16165	0.027812	0.164271
OR2A7	-1.16442	0.002262	0.046146
C17orf110	-1.16619	0.019538	0.13582
GSN	-1.16642	0.006749	0.07754
C1QC	-1.16709	0.015675	0.120517
ENST0000C	-1.16987	0.00532	0.068007
ENST0000C	-1.17087	0.031669	0.176411
FBXO25	-1.17092	0.003885	0.059189
ANKRD43	-1.17132	0.01848	0.132135
PPP1R36	-1.17156	0.018908	0.133798
TMEM82	-1.17186	0.002803	0.050657
FSIP1	-1.17294	0.000346	0.019206
MARVELD3	-1.17301	0.008314	0.086215
PTPN22	-1.17447	0.031816	0.176811
VWA5A	-1.17527	0.003931	0.059487
TRAF3IP3	-1.17659	0.011137	0.101008
ZNF135	-1.17721	0.000448	0.021666
GLTP	-1.17937	0.001303	0.035258
na	-1.18036	0.00528	0.067876
NLGN4X	-1.18083	0.001946	0.042534
CACNB2	-1.18107	0.009039	0.090088
CES3	-1.18234	0.000421	0.021076
na	-1.183	0.004843	0.06504
GSR	-1.18349	0.004682	0.06439
MXI1	-1.1848	0.01742	0.128036
SULT1A4	-1.18604	0.008206	0.085863
ACADS	-1.18669	0.000527	0.023421
RCSD1	-1.187	0.007112	0.079586
TET1	-1.1873	0.002424	0.047247
HLA-DPB1	-1.18796	0.02592	0.158075
PPARGC1B	-1.18969	0.001187	0.03389
SYNE1	-1.19348	0.013802	0.112541
KIT	-1.19459	0.049863	0.226026
MAP2K6	-1.19465	0.022264	0.145787
CYP3A5	-1.19645	0.041229	0.203957
C4orf34	-1.19667	0.014324	0.114795
COL17A1	-1.19676	0.039925	0.200692
CALCOCO2	-1.19689	0.002278	0.046266
RILP	-1.19702	0.007025	0.079164
QSOX1	-1.19824	0.002147	0.045036
GFRA3	-1.19934	0.005547	0.069742
MXI1	-1.20111	0.004297	0.061646

EIF5	-1.20287	0.017631	0.128911
FHL1	-1.20391	0.002365	0.046816
TNXB	-1.20499	0.001018	0.031126
MICAL3	-1.20525	0.02148	0.142923
P2RY4	-1.20891	0.017665	0.128993
TTC22	-1.21295	0.005289	0.067917
FXYD3	-1.21351	0.016842	0.125729
KIAA1683	-1.21357	0.0017	0.040051
GPNMB	-1.21432	0.011503	0.102643
GNA14	-1.21466	0.027906	0.164623
FAM178A	-1.21493	0.017521	0.128553
na	-1.21499	0.008	0.084691
GPX3	-1.215	0.004117	0.060737
KLRK1	-1.21502	0.023134	0.148861
MYZAP	-1.21517	0.006238	0.074196
FCRL5	-1.21548	0.013015	0.109298
UGT2B10	-1.21779	0.010133	0.095639
ATP8B1	-1.22305	0.008761	0.088638
SLC25A20	-1.22505	0.010996	0.100026
PAQR5	-1.22538	0.027013	0.161761
CLEC3B	-1.22559	0.030889	0.173687
na	-1.22703	0.004858	0.065103
na	-1.22811	0.030575	0.172833
na	-1.23106	0.003356	0.055387
BIN2	-1.23299	0.007711	0.082936
KIF13B	-1.23543	0.000886	0.029559
ENST00000	-1.2363	0.006475	0.07594
LPAR1	-1.23658	0.002423	0.047247
na	-1.23813	0.005054	0.066424
na	-1.23828	0.016538	0.124398
CCDC89	-1.23929	0.000342	0.019163
CD48	-1.23971	0.030524	0.172697
CTSS	-1.24075	0.035481	0.188036
LIPH	-1.24196	0.004524	0.063098
SH2D6	-1.24233	0.037894	0.195053
NOV	-1.24278	0.037425	0.193638
GSTM2	-1.2433	0.002272	0.046244
na	-1.24364	0.001718	0.040279
EPHA7	-1.24416	0.007921	0.084297
RAB37	-1.24518	0.005967	0.072607
ANKRD18B	-1.24715	0.038602	0.196982
PRDX6	-1.248	0.000878	0.029547
na	-1.24887	0.01204	0.105449
HSD3B1	-1.24959	0.007564	0.082167
SFMBT2	-1.25135	0.004926	0.065588
TMEM45B	-1.2526	0.013116	0.109654
CPA3	-1.25378	0.026039	0.158264

SLC22A23	-1.2538	0.002379	0.046978
LAX1	-1.25476	0.041141	0.203735
HLA-DPA1	-1.25547	0.01636	0.123478
KBTBD11	-1.25638	0.040181	0.201072
GCET2	-1.25745	0.001184	0.033824
APOE	-1.2575	0.008999	0.089856
HLA-DOB	-1.25863	0.023627	0.150647
MS4A4A	-1.25867	0.003655	0.057613
FXYD3	-1.25924	0.000804	0.028548
SLC25A23	-1.2593	0.000211	0.015409
IGSF9	-1.25967	0.000788	0.028217
DOCK2	-1.26143	0.012559	0.107531
ABHD3	-1.26227	0.00809	0.085172
AKAP9	-1.26231	0.002794	0.050538
NRG4	-1.26371	0.011777	0.104026
PLEKHA6	-1.26385	0.00034	0.019132
CD8A	-1.26678	0.035603	0.188414
CERK	-1.26853	0.013371	0.110661
KCTD12	-1.26872	0.029607	0.169684
TDP2	-1.26872	0.005317	0.068007
COQ6	-1.26879	0.002679	0.049328
CCL15	-1.26899	0.001205	0.034046
RCAN2	-1.26946	0.003611	0.057177
MOB3B	-1.26948	0.007066	0.079314
ENST00000	-1.26952	0.006637	0.076777
TMCC3	-1.26972	0.002092	0.044217
na	-1.27123	0.004237	0.061254
SLC30A4	-1.27174	0.001569	0.038615
ACAP1	-1.27262	0.024324	0.153089
HLA-DPB1	-1.27587	0.035332	0.187524
ASB2	-1.27604	0.015498	0.11985
CDHR2	-1.27655	0.005409	0.068516
MIXL1	-1.27856	0.001343	0.035616
PEX26	-1.27995	0.001307	0.035293
CHRNA1	-1.28058	0.020036	0.137355
na	-1.28293	0.04597	0.217024
MPEG1	-1.28422	0.008674	0.088239
BHMT2	-1.28734	0.001575	0.038684
CD38	-1.28883	0.01752	0.128553
PTPRH	-1.28962	0.000341	0.019135
CEACAM7	-1.28979	0.035824	0.189258
AGXT2L2	-1.29064	0.0013	0.035228
ACVRL1	-1.29089	0.017873	0.129774
NDRG2	-1.29193	0.011802	0.104148
ITGB7	-1.29246	0.006035	0.07291
NR3C1	-1.29481	0.001143	0.03307
PHLPP2	-1.29489	0.001469	0.037522

PAG1	-1.2954	0.013329	0.110534
PRKACB	-1.29717	0.009438	0.092468
ACOX1	-1.29753	0.006328	0.074953
CD37	-1.298	0.029673	0.16984
AGFG2	-1.29929	0.006767	0.077606
GOLM1	-1.30026	0.002366	0.046816
HHIP	-1.30109	0.033146	0.180778
ENST0000C	-1.30309	0.028632	0.166657
MPEG1	-1.30541	0.022062	0.145231
KCTD12	-1.30607	0.017099	0.126669
HLA-DPB1	-1.30894	0.019685	0.1362
CLMN	-1.30904	0.000693	0.026359
PTN	-1.31296	0.000899	0.029634
SLC15A1	-1.31431	0.038386	0.196295
ZSCAN1	-1.31761	0.000791	0.028269
MXD1	-1.31775	0.007392	0.080929
ESYT3	-1.31904	0.00269	0.049328
na	-1.32126	0.016027	0.12201
EVI2B	-1.32452	0.025978	0.158185
KIAA0513	-1.32484	0.00606	0.07299
ENTPD8	-1.3283	0.010925	0.099595
OGN	-1.32951	0.010552	0.09792
MYZAP	-1.33217	0.00855	0.087694
NCKAP5	-1.33694	0.008809	0.088859
C1orf81	-1.33769	0.000312	0.018271
PTPRCAP	-1.33881	0.02358	0.150486
ANKRD44	-1.34261	0.024574	0.154018
RNASE1	-1.34346	0.003146	0.053307
FRZB	-1.34584	0.015548	0.119953
NAT1	-1.34594	0.033855	0.18288
CCDC144A	-1.34614	0.005776	0.07142
KIF13B	-1.34991	0.004887	0.065319
PLCL2	-1.35287	0.010759	0.098751
MYO5B	-1.35298	0.002189	0.045346
CAMK1D	-1.35401	0.004383	0.06224
na	-1.35518	0.004757	0.064761
RAVER2	-1.35527	0.000769	0.02784
ZZEF1	-1.3557	0.008137	0.085442
GSTA2	-1.3586	0.018868	0.133699
CECR1	-1.36362	0.005126	0.066816
SEMA4G	-1.3639	0.010283	0.09641
ENST0000C	-1.36563	0.001506	0.037931
PBLD	-1.36633	0.012812	0.108651
SLC30A10	-1.36723	0.006637	0.076777
TMEM61	-1.37017	0.016669	0.124829
SLC15A2	-1.37175	0.001917	0.042167
XPNPEP3	-1.37205	0.002941	0.051992

ZMAT1	-1.37287	0.026157	0.158851
RORB	-1.3731	0.00713	0.07963
GAB1	-1.37355	0.006785	0.077736
SETBP1	-1.37475	0.003508	0.056475
KIAA1324	-1.37878	0.040153	0.201072
CXCL12	-1.3803	0.002319	0.046459
IL16	-1.38196	0.011613	0.102992
CASD1	-1.38568	0.002076	0.044137
SLC25A23	-1.38588	0.001848	0.041537
SLC22A23	-1.38792	0.001849	0.041537
PTPRR	-1.38845	0.005884	0.071972
VWA5A	-1.39024	0.009616	0.093422
ROR1	-1.39039	0.01611	0.122412
RAB27A	-1.39193	0.007039	0.079167
NR3C1	-1.39332	0.00146	0.03741
MTMR11	-1.39334	0.001866	0.04172
TCN2	-1.39368	0.00221	0.045441
ARL14	-1.39454	0.004485	0.062979
CAPN5	-1.39608	0.002448	0.047424
PTPRC	-1.39824	0.040452	0.201896
CXCL12	-1.39829	9.20E-05	0.010723
BCL2	-1.399	0.006749	0.07754
C17orf76	-1.40085	0.003143	0.053307
C6orf105	-1.40212	0.002421	0.047247
na	-1.4023	0.040093	0.201072
CDKL1	-1.40331	1.89E-05	0.00433
PPY	-1.41065	0.002878	0.051428
PTK2B	-1.4107	0.000727	0.027095
OR5A2	-1.41189	0.003104	0.053118
SYT17	-1.41267	0.025048	0.155464
UGDH	-1.41351	0.000467	0.021958
SGK2	-1.41854	0.009491	0.092749
SLAMF7	-1.41974	0.033939	0.183214
CREB3L3	-1.42142	0.002756	0.05013
PCSK5	-1.42173	0.00017	0.014309
BBIP1	-1.42268	0.008323	0.086258
ENST00000	-1.42391	0.000483	0.022369
TCEA3	-1.42422	0.01219	0.106022
HLA-DPB1	-1.42556	0.018332	0.131357
ABP1	-1.42579	0.000428	0.021218
HRK	-1.42799	0.003528	0.056616
MIER3	-1.42818	0.009086	0.090361
FAM134B	-1.42859	0.019041	0.134291
UGT1A6	-1.42978	0.039228	0.198523
MT2A	-1.4302	0.00305	0.052677
SYTL2	-1.43248	0.002044	0.043794
GPER	-1.43736	0.004726	0.06467

CSRNP3	-1.43835	0.004435	0.062588
C15orf48	-1.43847	0.033769	0.182581
FAM82A1	-1.4385	0.001675	0.039785
GOLM1	-1.44243	0.003145	0.053307
C7	-1.44369	0.000293	0.017972
SIDT1	-1.44405	0.020639	0.139827
CPNE8	-1.44426	0.007609	0.082497
GPRIN2	-1.44502	0.000433	0.021336
FECH	-1.44718	0.021678	0.14362
SDK2	-1.44799	0.000516	0.023158
NASP	-1.44996	0.000445	0.0216
HHIP	-1.45524	0.000852	0.029357
TPSAB1	-1.45564	0.01178	0.104026
PIGZ	-1.45792	0.003716	0.058116
na	-1.45816	0.011413	0.102218
na	-1.45942	0.011158	0.101059
ASPG	-1.46008	0.009867	0.094517
na	-1.46228	0.045229	0.215275
HTR4	-1.46452	0.011717	0.10363
SLC37A2	-1.46606	0.008712	0.088385
SCIN	-1.46784	0.026323	0.159498
CAMK2D	-1.47173	0.004324	0.061771
TPSD1	-1.47283	0.009043	0.090096
TST	-1.47454	0.009798	0.094201
FXYD1	-1.47532	9.33E-05	0.010731
FUCA1	-1.47591	0.015516	0.119876
BEND4	-1.47719	0.001626	0.03935
GFI1	-1.47737	0.004552	0.063328
TCF21	-1.48076	0.033421	0.181689
CCL5	-1.4874	0.035565	0.188273
FGFR3	-1.48802	0.022481	0.146685
ENST0000C	-1.48902	0.001092	0.032307
EAF2	-1.49108	0.002192	0.045346
BEST4	-1.49146	0.00019	0.015016
na	-1.49243	0.000657	0.025879
PBLD	-1.49286	0.009932	0.094699
ACVR1C	-1.49324	0.015536	0.119923
PLCD1	-1.4942	0.00042	0.021069
TECPR2	-1.49724	0.001425	0.036796
ITPKA	-1.49748	0.000976	0.030643
SULT1A2	-1.49802	0.002666	0.049207
SLCO2A1	-1.49842	0.003443	0.055978
ENST0000C	-1.49913	0.032044	0.177352
GPER	-1.50068	0.005749	0.071191
CD79A	-1.50415	0.002688	0.049328
na	-1.50634	0.043828	0.211262
TTLL7	-1.50635	0.008605	0.087914

SEMA4G	-1.50902	0.011151	0.101053
na	-1.51006	0.030047	0.171156
SMPD1	-1.5115	0.000319	0.018382
SETBP1	-1.51403	4.71E-05	0.007743
IGF1	-1.51454	0.011956	0.105017
AKNA	-1.51507	0.001503	0.037931
FCRL2	-1.5154	0.00084	0.029214
MMP28	-1.51584	0.002457	0.047507
ITM2A	-1.5177	0.004304	0.061722
APOBR	-1.51903	0.012451	0.107124
SOSTDC1	-1.5237	0.015119	0.118104
FRMD1	-1.52494	0.003005	0.052334
CCDC144A	-1.52548	0.004656	0.064173
A1CF	-1.52847	0.004202	0.061095
CHN2	-1.53066	0.044361	0.212891
ENDOD1	-1.5308	0.028693	0.166859
UGT1A8	-1.53144	0.028437	0.1663
PLAC8	-1.53532	0.04162	0.204894
ABAT	-1.53809	0.021571	0.143225
KLK15	-1.53871	0.009684	0.093601
NEDD4L	-1.53902	0.000466	0.021957
NRAP	-1.54041	0.023521	0.150269
CD36	-1.54206	0.021859	0.1445
CCR2	-1.54313	0.009938	0.094699
FAM71E2	-1.54401	0.000685	0.026283
STAB1	-1.54669	0.003098	0.053118
PDCD4	-1.55041	0.000364	0.019533
GDPD2	-1.55165	0.010074	0.095316
FCRL5	-1.55557	0.037121	0.192753
SLC36A1	-1.55738	0.000105	0.011394
ABCA5	-1.55847	0.006371	0.075215
ANK3	-1.5588	0.003512	0.056475
SSBP2	-1.56082	0.004158	0.060975
TARP	-1.56141	0.011332	0.101811
IZUMO2	-1.5639	0.003757	0.058515
SYNPO2	-1.56539	0.001742	0.040395
O3FAR1	-1.56857	0.046581	0.21841
FABP1	-1.56942	0.035938	0.189617
DHRS11	-1.56974	0.000186	0.014893
ESR2	-1.57243	0.003611	0.057177
TRANK1	-1.57504	0.004733	0.06467
NLGN1	-1.57991	0.001111	0.032541
SCGN	-1.58163	0.000159	0.014014
GLP2R	-1.58504	0.00011	0.011458
DDX26B	-1.59376	0.008286	0.086144
FXYD3	-1.59379	0.002849	0.051217
na	-1.59544	0.002497	0.047944

PLCD3	-1.59581	0.000301	0.018054
EPB41L4A	-1.59669	0.004244	0.061255
SLC37A2	-1.5997	0.004935	0.065597
TRAF3IP3	-1.60711	0.015666	0.120509
TEX11	-1.60775	0.00678	0.077711
MT1B	-1.61047	0.001016	0.031098
PPARGC1A	-1.61175	0.005259	0.067795
CD177	-1.61241	0.001966	0.042748
CAPN3	-1.61269	0.008378	0.086456
HRCT1	-1.61968	0.014911	0.117211
KLK1	-1.62142	0.011956	0.105017
TNXB	-1.62334	0.00136	0.03585
na	-1.62614	0.003794	0.058648
TMEM236	-1.63505	0.000457	0.021797
ANK3	-1.63599	0.00264	0.049127
SGSM1	-1.64598	0.002893	0.051643
FAM107A	-1.64791	0.005169	0.067117
GPR174	-1.65185	0.037701	0.194534
ADAMTSL1	-1.65323	0.000102	0.01119
TRANK1	-1.65509	0.001547	0.038414
ADORA3	-1.65738	0.000138	0.013094
FAM129C	-1.65801	0.027036	0.161787
CES2	-1.66082	0.006331	0.074953
PKNOX2	-1.66138	0.001407	0.036562
FRMD3	-1.6615	0.000885	0.029559
SLC16A9	-1.66975	0.008853	0.0891
LRRC31	-1.67291	0.01147	0.102577
PLA2G10	-1.67414	0.002868	0.051329
ACSF2	-1.67461	0.002331	0.04652
PARM1	-1.67597	0.006085	0.073123
ENST00000	-1.67964	0.000151	0.013645
C1orf186	-1.68117	0.00038	0.019853
PPYR1	-1.68302	0.001773	0.040765
MT1A	-1.68647	0.001121	0.032677
FOLR2	-1.6894	0.000539	0.023708
ADAM28	-1.69409	0.001682	0.039823
na	-1.69509	0.004324	0.061771
POU2AF1	-1.69646	0.000291	0.017929
MALL	-1.7086	0.014483	0.115485
SIAE	-1.7097	0.000386	0.020042
MAOA	-1.70996	0.000303	0.018061
na	-1.71011	0.047367	0.219978
TTLL6	-1.71068	0.000398	0.020434
FABP2	-1.71136	0.011188	0.101122
na	-1.71315	0.026841	0.161045
CFD	-1.71323	0.000152	0.013681
NHEJ1	-1.71753	0.000205	0.015344

ANKRD20A	-1.71837	0.002907	0.051781
CD3E	-1.72587	0.026971	0.161622
CAPN13	-1.72707	2.15E-05	0.004575
TMEM236	-1.72952	0.000118	0.012082
na	-1.73523	0.006009	0.072898
BTNL8	-1.73815	0.000109	0.011456
SLC6A7	-1.74327	0.00812	0.085318
LRRC31	-1.74431	0.019492	0.135705
FOLR2	-1.74975	0.000122	0.012224
METTL7A	-1.75039	0.000651	0.025813
CD200R1	-1.75086	0.0065	0.075974
SRI	-1.75603	0.000917	0.029803
SLC28A2	-1.75636	0.043977	0.211625
KRTAP13-2	-1.75715	0.028974	0.167539
FMO4	-1.75833	0.000647	0.025807
NAAA	-1.7584	6.73E-05	0.009289
FAM150B	-1.76307	0.008811	0.088859
NR5A2	-1.76373	0.006875	0.078236
C11orf86	-1.76391	0.012828	0.10873
SPTLC3	-1.76474	0.007968	0.08451
CLDN23	-1.76708	0.0022	0.045424
FAM46C	-1.76769	0.000503	0.022769
ATP2A3	-1.77037	0.001767	0.04073
KCNMA1	-1.77911	0.004872	0.06522
LPHN2	-1.78066	0.033483	0.181852
NAT2	-1.78203	0.037889	0.195053
ABCB1	-1.7866	0.004279	0.061514
TRANK1	-1.78711	0.006494	0.075956
GCNT3	-1.78823	0.00955	0.093134
KLF4	-1.78955	0.004761	0.064795
PRKACB	-1.79015	0.006038	0.072922
DENND2A	-1.79317	0.011515	0.102643
RDH5	-1.79338	0.000517	0.023207
LAMA1	-1.79386	0.004724	0.06467
LPHN3	-1.79389	0.000317	0.018331
DISP2	-1.79552	0.002224	0.045511
RGS9	-1.79719	0.000102	0.01119
CLIC5	-1.80219	0.01899	0.134046
TMEM63C	-1.80272	0.022235	0.145713
DPF3	-1.80307	3.41E-05	0.006304
na	-1.81105	0.041046	0.203391
MATN2	-1.81752	0.003174	0.053514
IRF4	-1.8226	0.000211	0.015409
ADH6	-1.8234	0.005591	0.070089
ENST0000C	-1.82348	0.02999	0.170928
ZMAT1	-1.82481	0.001407	0.036562
NEO1	-1.82511	0.010388	0.097034

HOXD10	-1.82757	0.011996	0.105252
SMPD3	-1.82833	0.00302	0.052463
SLITRK6	-1.82862	0.043024	0.208812
na	-1.82863	0.019107	0.134467
P2RY1	-1.83283	0.010158	0.095735
na	-1.83633	0.018135	0.130691
FCER1A	-1.83999	0.048083	0.221807
CLMN	-1.84544	0.001102	0.032479
na	-1.8459	0.0263	0.159419
CD160	-1.84804	0.000691	0.026359
P2RY1	-1.85307	0.004459	0.062769
ENST00000	-1.85428	0.004389	0.06224
FAM3D	-1.85499	0.002418	0.047237
GLP2R	-1.85639	0.000475	0.022182
ENST00000	-1.85794	0.007669	0.08266
IL6R	-1.86155	0.000464	0.021881
ANGPTL1	-1.86369	6.95E-05	0.009512
na	-1.86546	0.049627	0.225378
na	-1.86654	0.0419	0.205435
CTSW	-1.86768	0.007624	0.082497
DHRS11	-1.86846	0.000101	0.01119
SEMA6D	-1.86995	0.004052	0.060318
PDE9A	-1.87	0.0003	0.018054
PHGR1	-1.87206	0.001636	0.039483
na	-1.87307	0.000297	0.018054
GPA33	-1.88132	0.011486	0.102623
CD209	-1.8816	0.000223	0.015789
B3GNT7	-1.88307	0.00441	0.06241
na	-1.88585	0.000982	0.030689
MUC2	-1.88884	0.030355	0.172365
AKAP5	-1.89258	0.002974	0.052177
EPB41L3	-1.89273	0.005066	0.066451
ARHGAP44	-1.89294	0.00781	0.083642
IGLL1	-1.89317	0.036151	0.190236
PHLPP2	-1.89603	3.01E-06	0.001741
PIGR	-1.89941	0.015561	0.120003
IL6R	-1.90392	0.000124	0.012266
CDHR5	-1.90462	0.000328	0.018687
PLA2G10	-1.91109	0.000368	0.019687
MT1X	-1.91224	0.001661	0.039681
ARHGAP44	-1.91896	0.000809	0.028585
na	-1.91904	0.011182	0.101122
TP53INP2	-1.92305	0.000846	0.029272
BMP5	-1.92353	0.004812	0.064885
na	-1.9305	0.042501	0.207327
CDHR1	-1.93131	0.042782	0.208158
na	-1.93193	0.006612	0.076724

NEDD4L	-1.93214	0.000438	0.021436
MT1X	-1.93539	0.001151	0.033208
NEDD4L	-1.94019	0.001894	0.041878
PCSK6	-1.94045	0.002758	0.05013
ADH6	-1.94147	0.006491	0.075956
RIMKLA	-1.94334	0.004345	0.06196
ATP2A3	-1.94945	0.001286	0.034997
SAMD13	-1.95016	0.001856	0.041598
na	-1.95258	0.030323	0.172243
TSPAN1	-1.95385	0.016778	0.125395
na	-1.95634	0.024276	0.152876
na	-1.9584	0.006932	0.078652
na	-1.95872	0.049154	0.224349
AKAP5	-1.96097	0.001389	0.036273
SEPP1	-1.96502	0.012181	0.106022
SPINK2	-1.96996	0.001794	0.041108
CDKN2B	-1.97138	0.000737	0.027264
SLC10A5	-1.97147	0.000694	0.026359
ABI3BP	-1.97192	0.000436	0.021436
OTOP2	-1.97829	5.09E-06	0.002315
UNC5C	-1.97903	0.000912	0.029777
na	-1.9812	0.003498	0.056475
REP15	-1.98337	0.048057	0.221758
PNLIPRP2	-1.98377	0.027377	0.163085
FOXP2	-1.98479	0.00228	0.046266
SLC9A2	-1.98633	8.63E-05	0.010377
ITM2C	-1.992	0.001639	0.03949
na	-1.99339	0.026567	0.160287
MYH15	-1.99376	3.29E-05	0.006241
na	-1.99518	0.012842	0.108771
na	-2.00125	0.027931	0.164653
HPGDS	-2.00151	0.00074	0.0273
C2orf72	-2.00976	0.00416	0.060975
CCL28	-2.01414	0.004073	0.060389
na	-2.01432	0.014941	0.117242
MT1H	-2.01636	0.001957	0.042669
CHL1	-2.02065	0.001913	0.042136
KCNMA1	-2.0267	0.004751	0.064706
OSTBETA	-2.02736	0.00721	0.07993
MT1E	-2.02992	0.005834	0.071707
TBX10	-2.03164	0.004523	0.063098
na	-2.03261	0.032007	0.177236
PAPPA2	-2.03364	6.02E-05	0.008825
SLC17A4	-2.03422	0.002508	0.047996
PDZD3	-2.03444	0.005609	0.070237
ATP13A4	-2.04128	0.004732	0.06467
MZB1	-2.04207	0.002407	0.047202

SELENBP1	-2.04645	0.000758	0.027615
C11orf86	-2.05106	0.00715	0.079722
GPR15	-2.05479	0.013342	0.110607
VIPR1	-2.05567	7.66E-05	0.009704
EPHX2	-2.06571	3.33E-05	0.006241
NAAA	-2.06574	2.00E-05	0.00449
na	-2.06687	0.02268	0.147422
KLHL6	-2.06973	0.001562	0.038595
F13A1	-2.07149	0.008696	0.088385
FOXP2	-2.07461	0.001492	0.037812
MT1E	-2.077	0.001895	0.041878
na	-2.07792	0.014796	0.116811
MUC4	-2.08434	0.005844	0.071734
na	-2.086	0.049011	0.223911
ATP6V0D2	-2.0906	0.001571	0.038615
C17orf109	-2.09726	0.001371	0.035961
DNASE1L3	-2.0984	0.016387	0.12362
CHGB	-2.10229	0.001027	0.031297
BCAS1	-2.10843	0.008584	0.087891
na	-2.10935	0.042682	0.207896
MYO1A	-2.11066	0.00112	0.03266
PLXNA2	-2.1119	0.000206	0.015344
FGFR2	-2.11195	0.036304	0.190669
ANPEP	-2.1123	0.003207	0.053926
na	-2.11305	0.007683	0.082758
na	-2.12132	0.032049	0.177352
HPGD	-2.1331	0.001835	0.041465
CA12	-2.1403	0.013148	0.109738
ENST0000C	-2.14442	0.028386	0.166142
XDH	-2.14823	0.029515	0.169422
ATOH1	-2.1487	0.039013	0.197968
PCDH11Y	-2.1499	0.000826	0.028953
FLJ46446	-2.14992	0.002932	0.05196
HHLA2	-2.1538	0.000493	0.022555
TMEM171	-2.15553	0.001576	0.038684
MYLK	-2.15575	6.32E-06	0.002391
ATP13A4	-2.15969	0.005099	0.066643
CDHR1	-2.16152	0.002711	0.049525
SPTLC3	-2.16419	0.001226	0.03426
FMO5	-2.16718	0.000137	0.013094
na	-2.16852	0.005781	0.071433
na	-2.16965	0.010609	0.098097
SMPDL3A	-2.17169	0.002609	0.048991
C1orf81	-2.17234	1.68E-05	0.004132
ITM2C	-2.17291	0.001329	0.035462
na	-2.17309	0.043178	0.209206
KCNA3	-2.17396	0.001033	0.031318

EPB41L4A	-2.17709	0.000888	0.029559
SCN7A	-2.17715	0.000195	0.015124
HSD11B2	-2.18004	9.13E-05	0.010683
PCSK6	-2.18665	0.001203	0.034037
na	-2.18838	0.030796	0.173466
LRRN2	-2.19265	6.07E-05	0.008833
HOXD11	-2.19339	0.046912	0.218981
na	-2.1958	0.032236	0.178004
na	-2.19766	0.00758	0.082287
MUC2	-2.20071	0.030913	0.17372
na	-2.20608	0.028247	0.165775
LPHN2	-2.20883	0.020503	0.139213
FMO5	-2.20897	0.000314	0.018271
EDN3	-2.21285	0.002411	0.047216
CLIC5	-2.21481	0.023594	0.150528
GABRA2	-2.21755	0.00987	0.094517
RPS6KA6	-2.22718	0.000307	0.018106
AFF3	-2.22818	6.08E-07	0.00082
PLCE1	-2.22995	0.000927	0.030023
SULT1B1	-2.23011	0.005364	0.068213
F13A1	-2.23557	0.001042	0.031472
na	-2.23567	0.021453	0.142887
MOGAT2	-2.23858	0.014375	0.114985
MZB1	-2.24326	0.048973	0.223866
TMEM72	-2.24452	7.80E-05	0.00977
GNG7	-2.24487	0.000698	0.026421
na	-2.24858	0.024982	0.155312
HHLA2	-2.24904	0.001553	0.038472
SECTM1	-2.25428	9.35E-05	0.010731
AHCYL2	-2.25483	0.001346	0.035661
HSD3B2	-2.25918	0.028496	0.166438
CLDN8	-2.25938	0.004205	0.061095
KIF5C	-2.26066	0.003788	0.058639
PLCE1	-2.26266	0.000858	0.0294
PDGFD	-2.26608	0.000484	0.022369
SCG2	-2.27436	0.000145	0.013419
TNFRSF13B	-2.27691	0.000245	0.016594
FAM55D	-2.28165	0.00266	0.049196
C14orf176	-2.28381	0.002649	0.049141
ENST0000C	-2.28474	0.007513	0.081818
C1orf115	-2.29129	0.003127	0.053222
MFSD4	-2.29855	0.00011	0.011458
UGT1A6	-2.29862	0.012313	0.106407
KRT20	-2.31328	0.007135	0.079659
CD163L1	-2.31524	0.000417	0.021049
WFDC2	-2.31725	0.03683	0.192126
SDCBP2	-2.32032	0.002776	0.050357

FRMD3	-2.34208	0.000227	0.015885
VSTM2A	-2.34627	0.001235	0.034335
GLDN	-2.3562	0.042274	0.206729
na	-2.36403	0.020908	0.140625
CAPN9	-2.3664	0.008282	0.086129
BEST2	-2.3806	0.00042	0.021069
na	-2.38995	0.010673	0.098419
DHRS9	-2.39076	0.045744	0.216499
TMEM236	-2.39082	0.000261	0.017247
LPHN2	-2.40258	0.019943	0.137111
SEMA6A	-2.40978	0.000206	0.015344
RNF152	-2.41115	4.97E-05	0.008079
TTR	-2.41282	0.000315	0.018322
SEPP1	-2.41322	0.019271	0.135023
ENTPD5	-2.41764	0.000129	0.012531
TNFRSF17	-2.4347	0.001425	0.036796
na	-2.4348	0.036631	0.19164
HSD17B2	-2.43504	0.003533	0.056638
ABCB1	-2.45645	0.010764	0.098751
na	-2.45716	0.007374	0.080867
MUC12	-2.48911	0.03304	0.180446
CDHR5	-2.49339	0.001214	0.03419
ZBTB7C	-2.49449	0.008255	0.085952
na	-2.49552	0.010715	0.098567
CADM2	-2.49723	0.000151	0.013645
SPTLC3	-2.49986	0.003445	0.055978
STMN2	-2.50722	0.003497	0.056475
CKB	-2.52141	0.000514	0.023138
CPA6	-2.52246	4.61E-05	0.00762
NR3C2	-2.52336	0.000296	0.018054
CRHBP	-2.53149	2.90E-05	0.005644
USP2	-2.53258	0.000205	0.015344
na	-2.53339	0.011472	0.102577
FOXP2	-2.53762	0.000713	0.026727
ST6GALNA(	-2.54136	0.000953	0.030348
LDHD	-2.54168	0.000317	0.018331
SLC6A19	-2.54274	1.68E-05	0.004132
BMP3	-2.55436	0.001594	0.038953
MUC2	-2.55624	0.0206	0.1397
MT1G	-2.60074	0.000246	0.016594
PCSK5	-2.60164	9.05E-05	0.010661
ABI3BP	-2.61127	0.000305	0.018061
MT1F	-2.6258	5.39E-06	0.002331
CHST5	-2.62644	0.001538	0.0383
FCGBP	-2.6402	0.007095	0.07948
TTLL6	-2.64709	0.000478	0.022256
BCAS1	-2.65425	0.00432	0.061771

LGALS2	-2.671	0.000891	0.029559
EDN3	-2.67136	0.000858	0.0294
ISX	-2.67854	0.006195	0.074028
IL1R2	-2.68299	2.08E-05	0.004494
na	-2.68318	0.01057	0.097969
na	-2.69851	0.01224	0.106095
USP2	-2.70606	0.000788	0.028217
na	-2.75294	0.01076	0.098751
MCOLN3	-2.76427	3.09E-06	0.001741
PADI2	-2.76435	0.000452	0.021714
NR5A2	-2.76809	0.001615	0.039195
SCGB2A1	-2.77924	0.002932	0.05196
MAMDC2	-2.78407	2.26E-05	0.004719
na	-2.80274	0.013469	0.111027
MT1H	-2.80669	5.82E-05	0.00872
MT1M	-2.81806	0.000669	0.026049
ADHFE1	-2.83101	7.58E-05	0.009702
AMPD1	-2.8468	0.000435	0.021436
C2orf88	-2.84808	0.000218	0.015622
CAPN13	-2.89251	0.000219	0.015622
na	-2.89931	0.001057	0.031635
VSTM2A	-2.90572	2.89E-06	0.001741
na	-2.91097	0.016613	0.124623
GUCA2B	-2.92504	1.73E-06	0.001353
BTNL3	-2.93121	0.001259	0.034642
C10orf99	-2.95635	0.033027	0.180446
MEP1A	-2.9676	0.003937	0.059515
SPON1	-2.98048	0.000827	0.028953
na	-3.00892	0.02225	0.145744
CHP2	-3.01933	0.000617	0.025359
DCHS2	-3.02174	0.000104	0.011301
B3GALT5	-3.06151	0.002335	0.04652
SLC17A4	-3.07197	0.000563	0.024213
THRB	-3.07265	1.40E-05	0.003794
SPINK5	-3.07319	0.00252	0.048131
FAM55A	-3.09307	0.002752	0.050072
PADI2	-3.09323	0.000266	0.017271
TMIGD1	-3.10477	4.18E-05	0.007123
TSPAN7	-3.10893	0.000898	0.029634
HMGCS2	-3.10919	0.000772	0.02784
C6orf105	-3.12721	0.003134	0.053222
na	-3.14569	0.001079	0.032073
HEPACAM2	-3.16572	0.008372	0.086432
PTGDR	-3.1797	5.17E-05	0.008209
RETNLB	-3.18634	0.011523	0.102643
SCARA5	-3.20364	0.001686	0.039823
SLC26A2	-3.23342	0.001872	0.04172

ADAMDEC1	-3.25797	0.001332	0.035505
TRPM6	-3.31191	0.000189	0.015016
BTNL8	-3.33927	0.001851	0.041537
CXCL12	-3.38964	3.28E-06	0.001782
SPINK5	-3.42093	0.006463	0.075822
TRPM6	-3.44776	5.54E-06	0.002331
GCG	-3.47825	1.49E-06	0.001291
CWH43	-3.48618	1.77E-06	0.001353
na	-3.51862	0.003252	0.054455
CA2	-3.56027	0.00014	0.013205
SCNN1B	-3.56191	6.66E-05	0.009239
SPIB	-3.57608	9.75E-09	8.21E-05
PKIB	-3.6024	0.000147	0.013458
AQP8	-3.61691	4.76E-06	0.002226
VSIG2	-3.66995	0.005228	0.0676
B3GNT7	-3.68822	0.001491	0.037812
ITLN1	-3.77041	0.016226	0.122901
PCK1	-3.80747	9.58E-05	0.010938
CEACAM7	-3.83303	0.010704	0.098567
PCSK5	-3.83844	0.000298	0.018054
CPB1	-3.84576	0.005783	0.071433
CA7	-3.85135	2.03E-08	0.000114
ABCA8	-3.86569	7.40E-06	0.002653
ADH1A	-3.94183	0.001043	0.031472
SLC4A4	-4.21904	7.46E-05	0.009701
CLCA4	-4.2256	0.009938	0.094699
TRPM6	-4.23911	6.23E-05	0.008951
TMIGD1	-4.24458	2.16E-05	0.004575
TRPM6	-4.24854	0.00022	0.015659
ADH1C	-4.26935	0.002612	0.048991
MS4A12	-4.29888	0.000889	0.029559
CA4	-4.37633	0.000147	0.013458
SI	-4.51506	0.00122	0.034193
MS4A12	-4.60493	0.001802	0.041206
ZG16	-4.75937	0.004924	0.065588
TPH1	-4.80841	6.14E-06	0.002376
SLC26A3	-4.86917	0.001645	0.039533
GUCA2A	-4.88349	5.65E-05	0.00863
CD177	-5.49936	3.65E-05	0.006501
CLCA1	-5.50886	0.000955	0.030348
INSL5	-5.51358	0.001018	0.031126
CHGA	-5.56178	1.75E-05	0.0042
CD177	-5.57313	0.00013	0.012561
SST	-5.74713	2.93E-07	0.000659
GCG	-6.34476	1.13E-05	0.003345
CA1	-6.90487	0.000302	0.018054
