# Supplementary material for: Identification of Novel lncRNAs Related to Colorectal Cancer Through Bioinformatics Analysis
Source: Biomed Res Int. 2025 Jan 29;2025:5538575. doi: 10.1155/bmri/5538575 (PMC11824705; doi:10.1155/bmri/5538575)
Supplement: Supporting Information 2 — GSE134834 was analyzed in R software using the limma package, and DElncs were identified according to the defined criteria (p < 0.05 and |log (FC)| ≥ 0.5). [file 5538575.f2.pdf]

Identification of novel lncRNAs related to colorectal cancer through bioinformatic analysis

GSE134834 was analyzed in R software using the Limma package

GeneSymbol	logFC	P.Value	adj.P.Val
lncRNA p3467	4.480166	2.85E-06	0.001741
lncRNA p15938	4.028627	5.46E-07	0.00082
lncRNA p30155	3.962785	9.27E-07	0.001041
lncRNA p15421	3.776749	4.71E-06	0.002226
lncRNA p39026_v4	3.665108	1.30E-05	0.003626
lncRNA p15430	3.471953	1.41E-05	0.003794
lncRNA p34144_v4	3.388798	2.04E-05	0.004494
LOC340340	3.239423	8.20E-05	0.010084
lncRNA p29027	3.230258	1.37E-05	0.003769
lncRNA p34360_v4	3.207409	4.19E-05	0.007123
lncRNA p17009	3.193662	7.45E-07	0.000929
lncRNA p30261	3.089308	1.29E-06	0.001291
lncRNA p24045	3.084878	1.22E-06	0.001291
lncRNA p23831	3.08157	0.000903	0.029689
lncRNA p21811	3.058199	3.71E-07	0.00072
lncRNA p9376	3.050053	0.001343	0.035616
lncRNA p16534	3.036413	0.004104	0.060659
lncRNA p16370	2.990761	2.45E-05	0.004912
lncRNA p21975	2.926155	0.000445	0.0216
lncRNA p2786	2.904332	2.08E-05	0.004494
lncRNA p35380_v4	2.900774	0.000235	0.01617
lncRNA p956	2.865494	7.09E-07	0.000918
lncRNA p2787	2.793576	3.93E-05	0.00683
lncRNA p10603	2.788621	0.000184	0.014888
lncRNA p44527_v4	2.786527	8.51E-05	0.010317
lncRNA p28920	2.708014	4.85E-06	0.002238
LOC100653147	2.648485	0.000607	0.025159
lncRNA p2227	2.636894	0.000628	0.02557
lncRNA p18281	2.584973	0.000672	0.026049
lncRNA p10499	2.571046	0.000515	0.023158
lncRNA p14903	2.553404	2.34E-07	0.000601
lncRNA p21076	2.549822	1.08E-05	0.003269
lncRNA p21710	2.532405	1.47E-06	0.001291
lncRNA p30340	2.505407	9.64E-06	0.003215
lncRNA p29508	2.471494	2.72E-05	0.005388
lncRNA p33749	2.468114	2.29E-05	0.004727
lncRNA p26438	2.45529	0.000871	0.029538
lncRNA p15445	2.452066	0.031776	0.176704
lncRNA p25714	2.449723	7.60E-05	0.009702
lncRNA p11400	2.449627	0.002968	0.052177
lncRNA p15138	2.444671	0.000428	0.021218

lncRNA p26042	2.444671	0.001194	0.033978
LOC100292922	2.435443	0.005678	0.070659
lncRNA p9289	2.430086	5.97E-07	0.00082
lncRNA p16373	2.419692	1.58E-05	0.004132
lncRNA p29295	2.417101	0.008276	0.086092
lncRNA p33760	2.412764	5.66E-05	0.00863
lncRNA p15429	2.392588	0.00184	0.041501
lncRNA p36905_v4	2.383051	0.002201	0.045424
lncRNA p21160	2.378396	0.011939	0.10495
lncRNA p25763	2.360483	0.001558	0.038542
lncRNA p33596	2.342811	0.003249	0.054455
lncRNA p25432	2.34276	0.001101	0.032479
lncRNA p11322	2.326336	0.000564	0.024213
lncRNA p39031_v4	2.300936	0.000558	0.024133
lncRNA p3758	2.300324	1.02E-05	0.003269
lncRNA p10556	2.299778	0.00285	0.051217
lncRNA p36744_v4	2.281558	0.000314	0.018271
lncRNA p9879	2.275669	1.24E-05	0.003513
lncRNA p26665	2.272841	0.000292	0.017929
lncRNA p36264_v4	2.243034	1.76E-05	0.004202
lncRNA RNA146965|p0(	2.234645	3.61E-05	0.006501
lncRNA p29061	2.227512	0.000119	0.012082
lncRNA p41666_v4	2.22439	0.019178	0.134694
lncRNA p955	2.217952	1.45E-06	0.001291
lncRNA p23797	2.205057	0.000559	0.024133
lncRNA p957	2.200313	5.70E-07	0.00082
lncRNA p41651_v4	2.20011	0.000798	0.028425
lncRNA p44525_v4	2.188332	8.95E-05	0.010648
lncRNA p25167	2.179387	4.23E-05	0.007154
lncRNA p27912	2.165972	0.00414	0.06089
lncRNA p14904	2.16078	0.003862	0.05913
lncRNA p12897	2.153589	0.004244	0.061255
lncRNA p38136_v4	2.151433	0.001976	0.042806
lncRNA p8590	2.121697	0.04694	0.219044
lncRNA p37247_v4	2.111441	8.71E-05	0.010435
lncRNA p15328	2.109289	7.80E-06	0.002751
lncRNA p35793_v4	2.106777	0.000554	0.024089
lncRNA p28389	2.096193	0.000192	0.015016
lncRNA p15678	2.093527	0.000462	0.021881
lncRNA p10102	2.069611	0.00049	0.022487
lncRNA p20395	2.056577	0.008238	0.085952
lncRNA p20662	2.053843	0.041167	0.203829
lncRNA p9296	2.04488	0.000304	0.018061
lncRNA p15677	2.040768	0.032648	0.179255
lncRNA p7857	2.040637	0.014608	0.115997
lncRNA p11777	2.009459	0.009256	0.091432
lncRNA p37804_v4	1.99063	0.000198	0.015223

lncRNA p26361	1.981979	0.000194	0.015072
lncRNA p654	1.979007	0.006069	0.07299
lncRNA p10778	1.97496	0.017245	0.127415
lncRNA p16378	1.965643	0.000531	0.023498
lncRNA p14932	1.958192	0.001655	0.039612
lncRNA p7856	1.948414	0.00212	0.044668
lncRNA p39029_v4	1.941757	0.001142	0.033062
lncRNA p15360	1.922414	0.000285	0.017879
lncRNA p38411_v4	1.921921	6.08E-05	0.008833
lncRNA p8048	1.920955	0.004975	0.065872
lncRNA p29999	1.920851	0.000687	0.026333
lncRNA p33713	1.920376	0.000925	0.029982
lncRNA p29255	1.909655	0.012752	0.108416
lncRNA p33576	1.898144	0.002903	0.051746
lncRNA p28318	1.89327	1.92E-05	0.004381
lncRNA p29207	1.885693	0.001009	0.03101
lncRNA RNA33677|snoF	1.872574	0.000578	0.024627
lncRNA p1220	1.8712	0.00293	0.05196
lncRNA p26302	1.863358	0.002578	0.048754
lncRNA p5208	1.861054	0.002815	0.050767
lncRNA p23798	1.857363	0.001692	0.039894
LOC254057	1.85329	0.039837	0.200399
lncRNA p25411	1.83848	0.000156	0.013802
lncRNA p15361	1.836835	0.000877	0.029547
lncRNA p13558	1.833853	0.004011	0.06005
LOC729680	1.824979	0.001535	0.0383
lncRNA p27661	1.813651	0.016961	0.126119
lncRNA p38033_v4	1.813295	0.00026	0.017247
lncRNA p6956	1.80871	0.000325	0.018553
lncRNA p1001	1.807116	0.00087	0.029538
lncRNA p33841	1.804173	0.002297	0.0463
lncRNA p27159	1.801301	0.003347	0.055318
lncRNA p23381	1.800366	0.000652	0.025813
lncRNA p7231	1.792486	0.000161	0.014104
lncRNA RNA33526|snoF	1.790849	0.000764	0.027769
lncRNA p15662	1.786059	0.00191	0.042112
lncRNA p34806_v4	1.783945	2.33E-05	0.004755
lncRNA p11398	1.779317	0.022356	0.146242
lncRNA p13666	1.777429	3.40E-05	0.006304
lncRNA p1630	1.776027	0.006801	0.077843
lncRNA p35551_v4	1.764453	0.001213	0.03419
lncRNA p9881	1.751688	8.81E-06	0.002968
lncRNA p25536	1.750243	0.00097	0.030507
lncRNA p43703_v4	1.746959	0.000882	0.029547
lncRNA p16364	1.741905	0.000673	0.026049
lncRNA p28333	1.737918	0.000341	0.019135
lncRNA p28829	1.734877	0.003348	0.055318

lncRNA p25512	1.728591	0.00144	0.037075
lncRNA p13491	1.724884	0.002942	0.051992
lncRNA p36651_v4	1.71977	0.002203	0.045424
lncRNA RNA33446|snoF	1.713243	0.00249	0.047855
lncRNA p822	1.712793	0.002333	0.04652
lncRNA p28868	1.710544	0.000357	0.019313
lncRNA p28507	1.70892	0.003027	0.052507
lncRNA p24555	1.702926	7.76E-05	0.00977
lncRNA p29998	1.701597	0.00173	0.040395
lncRNA p25087	1.696747	0.004064	0.060389
lncRNA p29324	1.696695	0.002693	0.049328
lncRNA RNA33592|snoF	1.693808	0.000672	0.026049
lncRNA p28169	1.688686	0.002509	0.047996
lncRNA p29858	1.686868	0.032572	0.17904
lncRNA p26490	1.685732	0.0236	0.150528
lncRNA p9866	1.683898	0.028701	0.166859
LOC100653149	1.676555	0.013227	0.109983
lncRNA p23296	1.67639	0.002968	0.052177
lncRNA p36267_v4	1.674042	0.004125	0.060762
lncRNA p16210	1.669851	5.11E-05	0.008194
lncRNA p16377	1.668635	0.003038	0.052587
lncRNA p37121_v4	1.665401	0.000256	0.017109
lncRNA p39820_v4	1.662505	0.000633	0.02561
lncRNA p28907	1.656338	0.001224	0.034252
lncRNA p38141_v4	1.651077	0.002325	0.046514
lncRNA p9292	1.650593	1.13E-05	0.003345
lncRNA p6779	1.648739	0.000493	0.022555
lncRNA p7561	1.648562	0.00056	0.024133
lncRNA p11959	1.646335	0.004836	0.064972
lncRNA p10440	1.644467	0.000865	0.029443
LOC100128338	1.643755	7.97E-05	0.009904
lncRNA p24141	1.64256	0.019371	0.135312
lncRNA p41536_v4	1.641256	0.001165	0.033397
lncRNA p33425	1.641212	0.007231	0.080017
lncRNA p9290	1.640491	2.96E-05	0.005739
lncRNA RNA33448|snoF	1.639744	0.00094	0.030182
lncRNA p34331_v4	1.639087	0.031743	0.176585
lncRNA p18315	1.63689	0.001322	0.035401
lncRNA p10441	1.63585	0.003338	0.055282
lncRNA p3347	1.633277	0.002151	0.045072
lncRNA p42753_v4	1.633196	0.015252	0.118646
lncRNA p43702_v4	1.630369	0.002696	0.049361
lncRNA p41207_v4	1.622337	0.005442	0.068758
lncRNA p21043	1.621683	0.005866	0.07185
lncRNA p33561	1.61943	0.001305	0.035286
LOC157860	1.615401	0.001265	0.034751
lncRNA p16375	1.614786	0.000422	0.021083

lncRNA p37244_v4	1.614753	1.73E-05	0.0042
lncRNA p25286	1.613989	0.000705	0.026594
lncRNA p24237	1.610188	0.001923	0.042279
lncRNA p5979	1.610006	0.004526	0.063098
lncRNA p9294	1.608422	2.32E-05	0.004755
lncRNA p8357	1.606742	0.00063	0.02557
lncRNA p8185	1.605118	9.69E-05	0.010965
lncRNA p20665	1.598812	0.041023	0.203368
lncRNA p4290	1.598629	0.004162	0.060975
lncRNA p28001	1.597199	0.017356	0.12784
lncRNA p1176	1.595496	5.66E-05	0.00863
lncRNA p11317	1.595351	0.000263	0.017247
lncRNA p38955_v4	1.594877	0.000998	0.030802
lncRNA p2974	1.594325	0.000403	0.020525
lncRNA p14934	1.593596	0.001821	0.041294
lncRNA p37203_v4	1.592259	0.000881	0.029547
lncRNA p28827	1.591955	0.002853	0.051217
lncRNA p7544	1.590256	0.001147	0.033153
lncRNA p38412_v4	1.586859	0.001151	0.033208
lncRNA p37973_v4	1.585949	0.019496	0.135705
lncRNA p35019_v4	1.585009	0.01418	0.114129
lncRNA p9293	1.581757	1.78E-05	0.004221
lncRNA p15661	1.578949	0.00174	0.040395
lncRNA p34737_v4	1.578434	0.000423	0.021093
lncRNA p9291	1.57281	2.02E-05	0.004494
lncRNA p16419	1.56783	0.000199	0.015244
lncRNA p26153	1.56684	0.000234	0.01617
lncRNA RNA33715|snoF	1.564379	0.005227	0.0676
lncRNA p10439	1.562859	0.002932	0.05196
lncRNA p15162	1.558994	0.001203	0.034037
lncRNA p29482	1.558584	0.020579	0.139589
lncRNA p10442	1.558506	0.002524	0.04814
lncRNA p35428_v4	1.556474	0.002699	0.049383
lncRNA p43193_v4	1.555724	0.000694	0.026359
lncRNA RNA33688|snoF	1.555489	0.000307	0.018106
lncRNA p35123_v4	1.547141	0.002825	0.050911
lncRNA p15112	1.546886	0.001557	0.038524
lncRNA p16376	1.545776	0.001227	0.034276
lncRNA p41005_v4	1.544795	0.002396	0.047116
lncRNA p10088	1.543235	0.003945	0.05954
lncRNA p11597	1.541963	0.000936	0.030182
LOC100506190	1.537788	0.001972	0.042771
lncRNA p15753	1.537272	0.001735	0.040395
lncRNA p1124	1.536285	0.00165	0.039553
lncRNA p41731_v4	1.532451	0.004742	0.064703
lncRNA p8817	1.532157	0.004808	0.064885
lncRNA p34803_v4	1.531275	0.00031	0.018183

LOC100131831	1.527315	0.00052	0.023287
lncRNA p33859	1.52501	0.002142	0.045012
lncRNA p40666_v4	1.524011	0.004048	0.060318
lncRNA p36366_v4	1.510583	0.001372	0.035967
lncRNA RNA33407|snoF	1.507469	0.001159	0.033343
lncRNA p5025	1.503295	0.003631	0.057335
lncRNA p37933_v4	1.503295	0.004768	0.064811
lncRNA p25585	1.499988	0.010722	0.098573
lncRNA p38225_v4	1.496946	0.001485	0.037775
lncRNA RNA33615|snoF	1.496147	0.000903	0.029689
lncRNA p36268_v4	1.495051	0.001315	0.035374
lncRNA p35309_v4	1.486542	0.000905	0.029689
lncRNA p35458_v4	1.485275	9.89E-05	0.011104
lncRNA p38410_v4	1.484128	0.003085	0.053011
lncRNA p20795	1.481616	0.000633	0.025616
LOC100505730	1.479568	0.002088	0.044215
lncRNA p34881_v4	1.478282	0.006753	0.077558
lncRNA p17015	1.472739	0.000218	0.015622
lncRNA p19102	1.4682	0.007243	0.080058
lncRNA p15137	1.45879	0.003273	0.054573
lncRNA p8553	1.444303	0.005777	0.07142
lncRNA p10845	1.441867	0.011806	0.104151
lncRNA p12307	1.440142	1.88E-05	0.00433
lncRNA p24554	1.439082	0.00077	0.02784
lncRNA p16995	1.436467	0.001763	0.040705
lncRNA RNA33773|snoF	1.435075	0.003012	0.0524
lncRNA p35433_v4	1.431738	0.00916	0.0908
lncRNA p10930	1.431629	0.001078	0.032073
lncRNA p14176	1.427238	0.002212	0.045441
lncRNA p33802	1.427181	0.00373	0.058222
lncRNA p41652_v4	1.423453	0.009373	0.092046
lncRNA p911	1.423395	0.00037	0.019751
lncRNA p17156	1.422381	0.00788	0.084152
lncRNA p7445	1.421789	0.005113	0.066785
lncRNA p8751	1.419408	0.013607	0.111683
lncRNA p14175	1.408822	0.003107	0.053118
lncRNA p13543	1.402797	0.004707	0.064576
lncRNA RNA33664|snoF	1.402665	0.001084	0.032208
lncRNA p37712_v4	1.4001	0.000816	0.028724
lncRNA p11750	1.399846	0.000629	0.02557
lncRNA p33665	1.398756	0.001149	0.033207
lncRNA p33719	1.395379	0.030141	0.171469
lncRNA p25821	1.391093	0.000361	0.019411
lncRNA p16939	1.385565	0.006462	0.075822
lncRNA p39756_v4	1.383817	0.002273	0.046244
LOC100506190	1.380145	0.000894	0.029588
lncRNA p13258	1.377062	0.000845	0.029272

lncRNA p26405	1.371936	0.002693	0.049328
lncRNA p24449	1.371247	0.002878	0.051428
lncRNA p28866	1.369265	0.011412	0.102218
lncRNA p17206	1.369043	0.00391	0.0594
lncRNA p36822_v4	1.36895	0.002303	0.046325
lncRNA p28565	1.360408	3.53E-05	0.006435
lncRNA p33803	1.357493	0.009917	0.094699
lncRNA p9318	1.355932	0.009464	0.092615
lncRNA p42291_v4	1.355364	0.001834	0.041465
LOC100506190	1.35257	0.004796	0.064885
lncRNA p8355	1.352029	0.040122	0.201072
lncRNA p33936	1.346941	0.004181	0.061053
lncRNA RNA147125|p0:	1.346661	0.029747	0.170117
lncRNA p1123	1.339839	0.009677	0.093601
lncRNA p37239_v4	1.335972	0.001744	0.040395
lncRNA p5975	1.335783	0.017889	0.129812
lncRNA p33682	1.335147	0.024054	0.152178
lncRNA p21044	1.331289	0.005614	0.070237
lncRNA p43192_v4	1.330494	0.002581	0.048754
lncRNA p41255_v4	1.326744	0.003281	0.054688
lncRNA p29617	1.32303	0.001647	0.039533
lncRNA p13406	1.322158	0.000829	0.028953
lncRNA p24065	1.320809	0.003121	0.05322
lncRNA RNA33500|snoF	1.317121	0.00769	0.082784
lncRNA p33866	1.309387	0.000463	0.021881
lncRNA p34807_v4	1.3067	0.002327	0.046514
lncRNA p36266_v4	1.297724	0.024761	0.154514
lncRNA p25703	1.291716	0.0015	0.03793
lncRNA RNA96102|RNS_	1.291417	0.001885	0.041804
lncRNA p35430_v4	1.291202	0.001667	0.039707
lncRNA p6606	1.284712	0.003253	0.054455
LOC100506190	1.283104	0.002153	0.045072
lncRNA p7545	1.282352	0.027404	0.163144
lncRNA p30220	1.281933	0.002936	0.051989
lncRNA p36409_v4	1.28107	0.006492	0.075956
lncRNA p8175	1.280535	0.001523	0.038079
lncRNA p5848	1.279972	0.008735	0.088513
lncRNA p6756	1.278643	0.015265	0.118726
lncRNA RNA144458|snF	1.278206	0.027686	0.163982
lncRNA p16573	1.277619	0.001812	0.041279
lncRNA p24814	1.272723	0.010239	0.096201
lncRNA p28922	1.271402	0.009978	0.09491
lncRNA p44565_v4	1.264717	0.017533	0.12861
lncRNA p21127	1.26174	0.011617	0.103006
lncRNA p6051	1.260296	0.002476	0.047819
lncRNA p16372	1.254111	0.024872	0.154914
lncRNA p2271	1.251035	0.001313	0.035342

LOC100506411	1.250758	0.003546	0.056745
LOC100506190	1.240017	0.002348	0.046631
lncRNA p13664	1.23733	0.032664	0.179277
lncRNA p18863	1.235771	0.036869	0.192187
lncRNA p9954	1.233443	0.000601	0.025049
lncRNA p37875_v4	1.232725	0.009658	0.093559
lncRNA p39340_v4	1.232439	0.004537	0.063194
lncRNA RNA33496|snoF	1.227427	0.000206	0.015344
lncRNA p6605	1.226024	0.003318	0.055076
lncRNA p36265_v4	1.225863	0.005072	0.066451
lncRNA p26244	1.224994	0.002279	0.046266
lncRNA p1121	1.224906	0.008575	0.087891
lncRNA p2721	1.220689	0.006621	0.076742
lncRNA p26406	1.220033	0.011601	0.102939
lncRNA p870	1.219719	0.017948	0.130071
lncRNA p7427	1.217179	0.003377	0.055466
lncRNA p9781	1.213165	0.005835	0.071707
lncRNA p33759	1.212183	0.000552	0.024034
lncRNA RNA33786|snoF	1.2077	0.010163	0.095749
lncRNA p34095_v4	1.205355	0.003716	0.058116
lncRNA p17006	1.204043	0.005279	0.067876
lncRNA p38963_v4	1.199261	0.010736	0.098668
lncRNA p35848_v4	1.197011	0.013762	0.112458
lncRNA p35431_v4	1.192188	0.011154	0.101055
lncRNA RNA33540|snoF	1.190575	0.00197	0.042764
lncRNA p34736_v4	1.18749	0.003154	0.053361
lncRNA p33548	1.187438	0.018977	0.134018
lncRNA p30240	1.183357	0.020311	0.138593
lncRNA RNA33604|snoF	1.18067	0.004197	0.061078
lncRNA p2670	1.179257	0.00513	0.066816
lncRNA p26407	1.178965	0.015003	0.117472
lncRNA p30156	1.176848	0.006551	0.076306
lncRNA p16639	1.172321	0.003938	0.059515
lncRNA p7850	1.170966	0.04721	0.219616
lncRNA p28277	1.170827	0.0177	0.12913
lncRNA p38406_v4	1.170006	0.010023	0.095141
lncRNA p8160	1.169923	0.004044	0.060299
lncRNA p24867	1.169567	0.02407	0.152204
lncRNA p9295	1.16431	0.00324	0.054378
lncRNA p38467_v4	1.16355	0.005894	0.072055
lncRNA p3565	1.162644	0.018309	0.1313
lncRNA p35432_v4	1.159809	0.003507	0.056475
lncRNA p534	1.158143	0.004233	0.061254
lncRNA p38723_v4	1.157196	0.003781	0.058635
lncRNA p25165	1.157007	0.00094	0.030182
lncRNA RNA147586|p0(	1.156695	0.00261	0.048991
lncRNA p11574	1.154571	0.002528	0.048165

lncRNA p24241	1.153578	0.018094	0.13062
lncRNA p29038	1.153266	0.023663	0.150789
lncRNA p739	1.151449	0.001181	0.033792
LOC100130009	1.150734	0.000637	0.025626
lncRNA p2344	1.149753	0.001532	0.038292
lncRNA RNA96027|RNS	1.14859	0.020916	0.140656
lncRNA p15670	1.147361	0.041343	0.20437
lncRNA RNA146935|p0(	1.14602	0.000235	0.01617
lncRNA RNA33465|snoF	1.145473	0.007299	0.080377
lncRNA p15104	1.140172	0.00998	0.09491
lncRNA p39782_v4	1.135253	0.000543	0.023765
LOC286109	1.134572	0.00025	0.016776
lncRNA p28666	1.128269	0.000612	0.025233
lncRNA p20060	1.127866	0.000787	0.028217
lncRNA p17192	1.126256	0.022694	0.147461
lncRNA p33501	1.123761	0.006424	0.0755
lncRNA p33663	1.122472	0.0016	0.039016
lncRNA p24965	1.117214	0.013715	0.112188
lncRNA p22206	1.1169	0.000272	0.017383
lncRNA p27897	1.115593	0.045263	0.215283
lncRNA p8161	1.115373	0.002911	0.051814
lncRNA p14980	1.115119	0.028028	0.164995
lncRNA p30077	1.11408	0.018329	0.131357
lncRNA p14849	1.111912	0.025664	0.157446
lncRNA p34268_v4	1.111851	0.01201	0.105296
lncRNA RNA33733|snoF	1.109513	0.017603	0.128757
lncRNA p4049	1.107221	0.002591	0.048857
lncRNA p28495	1.105921	0.004725	0.06467
lncRNA p2337	1.105667	0.001235	0.034335
lncRNA p28615	1.105125	0.002431	0.047326
lncRNA p29186	1.104854	0.013958	0.113217
lncRNA p24236	1.098479	0.014348	0.114934
lncRNA p26498	1.096203	0.01153	0.102643
lncRNA p15919	1.09574	0.046861	0.218889
lncRNA p33696	1.094897	0.003814	0.058802
lncRNA p12895	1.094272	0.012157	0.105957
lncRNA p2451	1.092662	0.003398	0.055607
lncRNA p21029	1.092232	0.004883	0.065295
lncRNA p2329	1.092225	0.006415	0.075471
lncRNA p5555	1.092136	0.01071	0.098567
lncRNA RNA33759|snoF	1.091655	0.004402	0.062354
lncRNA p40091_v4	1.091488	0.002351	0.046631
lncRNA p26367	1.090832	0.029585	0.169651
lncRNA p15824	1.087823	0.027954	0.164762
lncRNA p25883	1.087748	0.005218	0.067553
lncRNA p29580	1.087123	0.008053	0.084896
lncRNA p12872	1.086904	0.004806	0.064885

lncRNA p178	1.085884	0.00188	0.041804
lncRNA p26536	1.084943	0.011325	0.101796
lncRNA RNA146087|vau	1.084706	0.021456	0.142887
lncRNA p33868	1.083887	0.002265	0.046182
lncRNA p7854	1.076705	0.016199	0.122811
lncRNA p39157_v4	1.073909	0.009861	0.094517
lncRNA RNA33740|snoF	1.073169	0.004699	0.064531
lncRNA RNA33785|snoF	1.072624	0.007034	0.079167
lncRNA p40143_v4	1.072097	0.006958	0.078724
LOC100652727	1.071133	0.000869	0.029538
lncRNA p35846_v4	1.069437	0.028776	0.166926
LOC100506190	1.068926	0.004555	0.063343
lncRNA p34068_v4	1.068671	0.000142	0.013274
lncRNA p29298	1.068189	0.018261	0.13118
lncRNA p29116	1.067615	0.003693	0.057988
lncRNA p37999_v4	1.066766	0.010824	0.099047
lncRNA p811	1.065415	0.012206	0.106095
lncRNA p36705_v4	1.06505	0.000288	0.017929
lncRNA p15416	1.062338	0.000271	0.017383
lncRNA p2241	1.060507	0.000649	0.025813
lncRNA p2332	1.05943	0.001426	0.036796
lncRNA p15116	1.058275	0.003918	0.059422
LOC128322	1.056978	0.005444	0.068758
lncRNA p8176	1.051322	0.002651	0.049143
lncRNA p2474	1.050981	0.007807	0.083642
lncRNA p33666	1.047704	0.009144	0.090693
lncRNA p6239	1.046253	0.010716	0.098567
lncRNA p6083	1.045055	0.022644	0.147306
lncRNA p24235	1.043169	0.007717	0.082968
lncRNA p1912	1.043119	0.018982	0.134018
lncRNA p37737_v4	1.040641	0.013243	0.11001
lncRNA RNA33494|snoF	1.040587	0.002433	0.047326
lncRNA p4269	1.039821	0.010824	0.099047
LOC729887	1.038777	0.006762	0.077606
lncRNA RNA33487|snoF	1.038038	0.015148	0.118137
lncRNA p14877	1.036491	0.008114	0.085282
lncRNA p44239_v4	1.034368	0.000566	0.024251
lncRNA p25743	1.031352	0.031884	0.176977
lncRNA p4296	1.030345	0.033623	0.182172
lncRNA p2335	1.030332	0.001677	0.039785
lncRNA p3210	1.030052	0.001337	0.035549
lncRNA p25780	1.021456	0.00071	0.026683
lncRNA p35899_v4	1.019111	0.029248	0.16838
lncRNA p36559_v4	1.017292	0.001006	0.030963
lncRNA p25452	1.016093	0.042354	0.206881
lncRNA p11163	1.015412	0.027111	0.162203
lncRNA p10599	1.01483	0.022253	0.145744

lncRNA p15814	1.010116	0.018232	0.131054
lncRNA p35502_v4	1.009948	0.001216	0.034193
lncRNA p39295_v4	1.008505	0.017565	0.128685
lncRNA p14724	1.007144	0.036894	0.192204
lncRNA p2334	1.006825	0.002506	0.047996
lncRNA p2338	1.006647	0.004834	0.064972
lncRNA p3161	1.006589	0.001604	0.039106
lncRNA RNA147194|p02	1.00654	0.029125	0.167999
lncRNA p26022	1.006194	0.00717	0.07984
lncRNA p26491	1.006108	0.031109	0.17445
lncRNA p15839	1.006009	0.026665	0.160562
lncRNA RNA33430|snoR	1.003671	0.035098	0.186946
lncRNA p6241	1.003558	0.004857	0.065103
lncRNA RNA33716|snoR	1.002878	0.022837	0.148077
lncRNA p11095	1.002835	0.037413	0.193613
lncRNA p2331	1.002748	0.003767	0.058588
lncRNA p33677	1.002065	0.006832	0.077932
lncRNA p6479	1.000319	0.002604	0.048983
lncRNA p9099	0.997379	0.028834	0.167111
lncRNA p25139	0.997249	0.001007	0.030963
lncRNA p37838_v4	0.997232	0.00705	0.079208
lncRNA p2328	0.996558	0.008205	0.085863
lncRNA p8588	0.996547	0.012203	0.106095
lncRNA p25266	0.994937	0.014956	0.117242
lncRNA p33693	0.994603	0.013226	0.109983
lncRNA p16969	0.99342	0.002609	0.048991
lncRNA p25231	0.991885	0.023938	0.151942
LOC100130581	0.991377	0.004629	0.063995
lncRNA p2070	0.991319	0.004195	0.061078
lncRNA p28714	0.989319	0.015057	0.117729
lncRNA p36296_v4	0.987421	0.00592	0.072182
lncRNA RNA33611|snoR	0.987352	0.003511	0.056475
lncRNA p39122_v4	0.984839	0.008857	0.0891
lncRNA p6478	0.983602	0.025657	0.157446
lncRNA p15102	0.982204	0.013051	0.109332
lncRNA p25370	0.98134	0.008776	0.088662
lncRNA p34064_v4	0.980859	0.038307	0.196164
lncRNA RNA147041|p01	0.980805	0.034964	0.186448
lncRNA p37645_v4	0.979731	0.000913	0.029777
lncRNA RNA95892|RNS	0.979549	0.001003	0.030916
lncRNA p9417	0.978511	0.016245	0.122989
lncRNA p17779	0.977537	0.004998	0.065995
lncRNA p35501_v4	0.9765	0.002367	0.046816
lncRNA p17588	0.973612	0.026696	0.160661
lncRNA p38409_v4	0.971677	0.001102	0.032479
lncRNA p34890_v4	0.968943	0.00891	0.089341
lncRNA p43732_v4	0.966843	0.017451	0.128175

lncRNA p4208	0.966673	0.000915	0.029777
lncRNA p2543	0.964895	0.03085	0.17363
lncRNA p34805_v4	0.964637	0.01471	0.116395
lncRNA p35398_v4	0.963843	0.012061	0.105554
lncRNA p33867	0.959157	0.01527	0.118731
lncRNA p38826_v4	0.959074	0.018057	0.130525
lncRNA RNA33432|snoF	0.957695	0.015964	0.121739
lncRNA p9419	0.955903	0.013578	0.111595
lncRNA p41216_v4	0.955696	0.002971	0.052177
LOC388796	0.953471	0.017466	0.128234
lncRNA p15115	0.952175	0.00552	0.06948
lncRNA p26500	0.950607	0.002604	0.048983
lncRNA p30172	0.949376	0.013707	0.112177
lncRNA p35504_v4	0.947198	0.001846	0.041537
lncRNA p34266_v4	0.946833	0.009643	0.09354
lncRNA p33937	0.945874	0.016168	0.12266
lncRNA p9287	0.945616	0.027605	0.163778
lncRNA p25560	0.945304	0.004295	0.061646
lncRNA p28919	0.945122	0.003508	0.056475
lncRNA p5006	0.943912	0.002923	0.051896
lncRNA p3266	0.94359	0.002438	0.047385
lncRNA p26499	0.942797	0.006692	0.077118
lncRNA p329	0.941518	0.01077	0.098751
lncRNA p30043	0.941034	0.026484	0.159929
lncRNA p36734_v4	0.939621	0.014312	0.114795
lncRNA p36654_v4	0.938247	0.002877	0.051428
lncRNA p33938	0.937361	0.007593	0.082383
lncRNA p44294_v4	0.937202	0.013161	0.109746
lncRNA p30148	0.936898	0.012217	0.106095
lncRNA p17084	0.936749	0.012861	0.108821
lncRNA RNA33711|snoF	0.93635	0.005812	0.071524
lncRNA p41419_v4	0.934241	0.019317	0.135103
lncRNA p4435	0.934201	0.00204	0.043739
lncRNA p5878	0.931753	0.005425	0.068644
lncRNA p29335	0.931126	0.025982	0.158185
lncRNA p4268	0.931003	0.02868	0.166859
lncRNA p16998	0.927646	0.003006	0.052334
lncRNA p5286	0.927109	0.004582	0.063592
lncRNA p37310_v4	0.926814	0.036366	0.190893
lncRNA p6328	0.925269	0.008215	0.085898
lncRNA p29035	0.924835	0.009651	0.093554
lncRNA p27422	0.924704	0.000598	0.024991
lncRNA p14340	0.923574	0.0255	0.156933
lncRNA p39151_v4	0.921038	0.004057	0.060354
lncRNA p466	0.920954	0.008757	0.088631
lncRNA p25369	0.92054	0.021326	0.142531
lncRNA RNA33768|snoF	0.919615	0.022244	0.145741

lncRNA RNA33613|snoF	0.918859	0.012719	0.108297
lncRNA p35456_v4	0.914995	0.001606	0.03911
lncRNA p15817	0.914416	0.00453	0.063131
lncRNA p15114	0.913997	0.024171	0.152523
lncRNA p16940	0.913238	0.008599	0.087914
lncRNA p11055	0.912788	0.046537	0.21832
lncRNA p15679	0.90976	0.023495	0.150234
lncRNA p29838	0.907553	0.024826	0.15478
lncRNA p4046	0.905589	0.011595	0.10293
lncRNA p2343	0.904738	0.001274	0.034854
lncRNA p10598	0.904708	0.038668	0.19711
lncRNA p2330	0.904655	0.006479	0.07594
lncRNA p41669_v4	0.902516	0.001292	0.035103
lncRNA p10600	0.902169	0.023043	0.148584
lncRNA p33925	0.901498	0.036146	0.190236
lncRNA RNA33776|snoF	0.898449	0.005857	0.071793
lncRNA p464	0.895431	0.009111	0.090528
lncRNA p23314	0.894995	0.004506	0.063084
lncRNA p34616_v4	0.893477	0.008841	0.089053
lncRNA p33635	0.892331	0.014146	0.113981
lncRNA p2333	0.892198	0.008719	0.088386
LOC100505473	0.891779	0.012824	0.108721
lncRNA p35927_v4	0.890939	0.008894	0.089272
lncRNA p35878_v4	0.888923	0.009079	0.090361
lncRNA p25461	0.887947	0.014069	0.11372
lncRNA p8609	0.887784	0.024578	0.154018
lncRNA p4368	0.887187	0.020377	0.138594
lncRNA p19520	0.884289	0.026694	0.160661
lncRNA p26993	0.883773	0.017897	0.129839
lncRNA p8114	0.883133	0.018845	0.133692
lncRNA RNA33696|snoF	0.878632	0.014046	0.11368
lncRNA p13247	0.876022	0.037806	0.194778
lncRNA p14466	0.875499	0.01495	0.117242
lncRNA p23619	0.875475	0.011298	0.1017
lncRNA p1363	0.87454	0.004176	0.061048
lncRNA p17278	0.874473	0.040994	0.203299
lncRNA p11168	0.873752	0.021865	0.1445
lncRNA p33709	0.872618	0.004625	0.063995
lncRNA p15990	0.871095	0.012926	0.108911
lncRNA p43003_v4	0.870255	0.030101	0.171384
lncRNA p17217	0.870235	0.000865	0.029443
lncRNA p2862	0.870223	0.003507	0.056475
lncRNA p33520	0.869693	0.026958	0.161574
lncRNA p15649	0.869628	0.003729	0.058222
lncRNA p25629	0.868577	0.039271	0.198709
lncRNA p25774	0.865872	0.013029	0.109332
lncRNA p29619	0.863086	0.046706	0.218535

lncRNA p867	0.86267	0.010284	0.09641
lncRNA p27462	0.859486	0.002782	0.050428
lncRNA p21362	0.857751	0.011057	0.10052
lncRNA p25614	0.857358	0.017222	0.127387
lncRNA p13516	0.857196	0.03785	0.194916
lncRNA p14058	0.854269	0.023044	0.148584
lncRNA p27983	0.852435	0.04158	0.204858
lncRNA p10649	0.850202	0.029336	0.168745
lncRNA p10493	0.850172	0.010227	0.096147
lncRNA p14825	0.847984	0.00871	0.088385
lncRNA p29316	0.847159	0.014475	0.115485
lncRNA p29032	0.844006	0.007366	0.080832
lncRNA p35217_v4	0.841863	0.036934	0.192301
lncRNA p33500	0.840496	0.007019	0.079123
lncRNA RNA33435|snoF	0.840391	0.008473	0.087123
lncRNA RNA33480|snoF	0.839367	0.007777	0.08343
lncRNA RNA146995|p0(	0.836935	0.002945	0.052011
lncRNA p6325	0.833029	0.019997	0.13724
lncRNA RNA96035|RNS_	0.832249	0.009736	0.09383
lncRNA RNA33597|snoF	0.831479	0.008763	0.088638
lncRNA p27469	0.831209	0.03345	0.181702
lncRNA p28298	0.8308	0.037327	0.193373
lncRNA p34414_v4	0.829916	0.00373	0.058222
lncRNA p34143_v4	0.828241	0.009211	0.0912
lncRNA p33735	0.82756	0.012484	0.107306
lncRNA p37053_v4	0.826269	0.007657	0.082625
LOC100128077	0.825411	0.014568	0.115872
lncRNA p37050_v4	0.823694	0.013048	0.109332
lncRNA RNA33518|snoF	0.822146	0.028615	0.166604
lncRNA p26507	0.820733	0.003571	0.056863
lncRNA p13451	0.820102	0.006125	0.073504
lncRNA p5159	0.819796	0.013234	0.109983
lncRNA p10433	0.81967	0.008236	0.085952
lncRNA p34405_v4	0.819639	0.044699	0.213766
lncRNA RNA33721|snoF	0.818328	0.008032	0.084842
lncRNA p28890	0.814516	0.019139	0.134534
lncRNA p26790	0.814099	0.003673	0.057791
lncRNA p43767_v4	0.813242	0.003766	0.058587
LOC100289388	0.81183	0.004354	0.062013
lncRNA p33532	0.811401	0.012222	0.106095
lncRNA p2793	0.810175	0.026823	0.161032
lncRNA p38954_v4	0.808061	0.010931	0.099619
lncRNA p28622	0.8065	0.023991	0.152017
lncRNA p34289_v4	0.806228	0.009085	0.090361
lncRNA p20622	0.804604	0.00483	0.064971
lncRNA RNA147445|p0!	0.802652	0.006191	0.074028
lncRNA p36733_v4	0.802181	0.014165	0.114065

lncRNA p191	0.801779	0.013354	0.110628
lncRNA p33706	0.800327	0.044936	0.214488
lncRNA p29461	0.798251	0.018792	0.133487
lncRNA p37501_v4	0.797319	0.004007	0.060046
LOC100506190	0.796773	0.013039	0.109332
lncRNA p21763	0.796584	0.00439	0.06224
lncRNA p5912	0.796531	0.002676	0.049316
lncRNA p539	0.795791	0.006387	0.075379
lncRNA RNA95114|RNS	0.795377	0.023174	0.149032
lncRNA p30046	0.794792	0.019526	0.13582
lncRNA p126	0.793418	0.03433	0.184461
lncRNA p33638	0.790751	0.020608	0.139725
lncRNA p16731	0.790484	0.004789	0.06486
lncRNA p2099	0.790327	0.014861	0.116971
lncRNA p29034	0.789867	0.046046	0.217211
lncRNA p4844	0.788822	0.002447	0.047424
lncRNA RNA95930|RNS	0.788706	0.021847	0.144461
lncRNA p3265	0.788688	0.006591	0.076639
lncRNA p4526	0.788347	0.03829	0.196164
lncRNA p30034	0.787754	0.001873	0.04172
lncRNA p18504	0.784269	0.041542	0.204814
lncRNA p14745	0.783903	0.046857	0.218889
lncRNA p13385	0.783301	0.045222	0.215273
lncRNA p28453	0.782286	0.034231	0.18416
lncRNA p2462	0.781588	0.02521	0.155983
lncRNA p3222	0.781388	0.036133	0.190202
lncRNA p23898	0.781349	0.04154	0.204814
lncRNA p16926	0.781202	0.038651	0.197069
lncRNA p38952_v4	0.78098	0.004924	0.065588
lncRNA p10125	0.780453	0.034701	0.185571
lncRNA p4430	0.780272	0.036842	0.192126
lncRNA p11284	0.777817	0.015892	0.121429
lncRNA p4302	0.777635	0.034622	0.185413
lncRNA p28933	0.777135	0.027138	0.162226
lncRNA p33734	0.775976	0.012915	0.108883
lncRNA RNA33732|snoF	0.775403	0.013927	0.113097
lncRNA p27244	0.774518	0.029137	0.167999
lncRNA p33637	0.772441	0.014148	0.113981
lncRNA p33439	0.772173	0.032873	0.17996
lncRNA RNA33508|snoF	0.770609	0.005068	0.066451
lncRNA p9935	0.769835	0.045905	0.216911
lncRNA p34873_v4	0.769675	0.035907	0.189571
lncRNA p25624	0.768509	0.025503	0.156933
lncRNA p37052_v4	0.767755	0.013559	0.111556
lncRNA p34870_v4	0.766828	0.041998	0.205787
lncRNA RNA33691|snoF	0.763561	0.017058	0.126617
lncRNA RNA33658|snoF	0.763464	0.013019	0.109298

lncRNA p28682	0.762952	0.026282	0.159398
lncRNA p33899	0.762237	0.042224	0.206546
lncRNA p21378	0.761258	0.023953	0.151944
lncRNA p9743	0.76042	0.022411	0.146441
lncRNA p28482	0.75854	0.032529	0.179005
lncRNA p18500	0.758514	0.040885	0.202914
lncRNA RNA33724|snoF	0.757389	0.022962	0.1484
lncRNA p813	0.757079	0.008368	0.086432
lncRNA RNA147423|p0!	0.757012	0.015136	0.118128
lncRNA p39250_v4	0.756845	0.010134	0.095639
lncRNA RNA147242|p0:	0.756643	0.040195	0.201072
lncRNA p37717_v4	0.755729	0.024745	0.154491
lncRNA p36785_v4	0.755641	0.010844	0.099069
lncRNA RNA95378|RNS_	0.752435	0.005007	0.066016
lncRNA RNA144469|snF	0.751872	0.006671	0.076957
lncRNA p19101	0.751869	0.028795	0.166926
lncRNA p33585	0.751766	0.024657	0.154199
lncRNA p10555	0.750353	0.03935	0.198991
LOC100131831	0.750078	0.04849	0.222938
lncRNA p3530	0.748829	0.010551	0.09792
lncRNA RNA145882	0.748217	0.041693	0.205048
lncRNA p30061	0.747568	0.018035	0.130435
LOC100506190	0.746691	0.028522	0.166548
lncRNA p7102	0.746191	0.021901	0.14456
lncRNA p28719	0.745104	0.008367	0.086432
lncRNA p28701	0.744282	0.042661	0.207868
lncRNA p39144_v4	0.743228	0.009774	0.09406
lncRNA RNA33468|snoF	0.743196	0.012981	0.109093
lncRNA p8776	0.742738	0.008449	0.087007
lncRNA RNA147360|p0	0.7421	0.03951	0.199378
lncRNA p41377_v4	0.740809	0.044907	0.214378
lncRNA p2356	0.740571	0.012006	0.105296
lncRNA p27350	0.740091	0.013807	0.112541
lncRNA RNA33539|snoF	0.740063	0.014604	0.115997
lncRNA p25773	0.738614	0.027644	0.16391
lncRNA p27841	0.737946	0.023512	0.150252
lncRNA p27590	0.737563	0.045298	0.215333
lncRNA p3208	0.735403	0.021309	0.142471
lncRNA p38818_v4	0.733461	0.022012	0.145068
lncRNA p34875_v4	0.732194	0.044051	0.211851
lncRNA RNA33514|snoF	0.731585	0.013136	0.109738
lncRNA p2340	0.728606	0.009848	0.094451
lncRNA p34342_v4	0.728021	0.043338	0.209562
lncRNA p467	0.726824	0.003004	0.052334
lncRNA RNA33695|snoF	0.72671	0.032754	0.179632
lncRNA p15799	0.726644	0.02103	0.14109
lncRNA p35869_v4	0.725659	0.012917	0.108883

lncRNA p546	0.725643	0.013358	0.110628
lncRNA p23152	0.72509	0.01117	0.101088
lncRNA p27339	0.723522	0.029421	0.169
lncRNA p4156	0.723438	0.007275	0.080211
LOC650293	0.722246	0.003505	0.056475
lncRNA p18871	0.721588	0.040842	0.20288
lncRNA RNA147664|p0	0.719101	0.013242	0.11001
lncRNA p41610_v4	0.718347	0.030413	0.172552
lncRNA p37018_v4	0.717483	0.036245	0.190552
lncRNA p36841_v4	0.716931	0.025076	0.155511
lncRNA p33679	0.716091	0.022538	0.146896
lncRNA p6109	0.716028	0.014281	0.114619
lncRNA p5818	0.715806	0.023097	0.148785
lncRNA p39190_v4	0.715313	0.013884	0.11288
lncRNA p14495	0.712843	0.049484	0.225065
lncRNA p26729	0.711148	0.048284	0.222397
lncRNA p7914	0.710638	0.040249	0.201207
lncRNA p11798	0.710615	0.01634	0.123377
lncRNA RNA95068|RNS	0.71004	0.020531	0.139346
lncRNA p33636	0.709731	0.03633	0.190734
lncRNA p16425	0.709267	0.025462	0.15685
lncRNA p40076_v4	0.706151	0.023241	0.149198
lncRNA p33595	0.704439	0.048622	0.22316
lncRNA p30237	0.703908	0.031576	0.176094
lncRNA p42164_v4	0.701847	0.017036	0.126484
lncRNA p2400	0.700895	0.014358	0.114957
lncRNA p11283	0.698137	0.03128	0.175197
lncRNA RNA147673|p0	0.698105	0.008705	0.088385
lncRNA p33448	0.697091	0.015983	0.121802
lncRNA p7192	0.696939	0.028436	0.1663
LOC100288602	0.695573	0.004784	0.06486
lncRNA p17869	0.695236	0.043792	0.211149
lncRNA p13542	0.693602	0.009514	0.092888
lncRNA p28911	0.693184	0.010132	0.095639
lncRNA p6590	0.692128	0.013423	0.110845
lncRNA p24490	0.691994	0.047002	0.219095
lncRNA p35147_v4	0.691345	0.027721	0.164131
lncRNA p2492	0.691142	0.042707	0.20797
lncRNA p38816_v4	0.690337	0.021933	0.144652
lncRNA p25889	0.68949	0.042532	0.207429
lncRNA p27840	0.688444	0.041094	0.203559
lncRNA p6789	0.688001	0.006012	0.072898
lncRNA p26651	0.687841	0.025508	0.156935
lncRNA p29031	0.686936	0.018151	0.13073
lncRNA p33609	0.683689	0.012546	0.107493
lncRNA p1116	0.68348	0.022907	0.148244
lncRNA RNA33722|snoR	0.682092	0.021346	0.14262

lncRNA p7524	0.681446	0.008042	0.084853
lncRNA p8143	0.680678	0.049074	0.224078
lncRNA p11948	0.679995	0.020286	0.138491
lncRNA p9986	0.679924	0.016717	0.125074
lncRNA RNA33781|snoF	0.678709	0.014828	0.116865
lncRNA p17151	0.678148	0.045069	0.214932
lncRNA p10567	0.677265	0.024025	0.152142
lncRNA p25581	0.677065	0.014314	0.114795
lncRNA p33441	0.675811	0.021601	0.143242
lncRNA p5920	0.675106	0.012686	0.108185
lncRNA p36234_v4	0.674683	0.022852	0.148087
lncRNA p44354_v4	0.673287	0.017563	0.128685
lncRNA p23073	0.673275	0.033915	0.183134
lncRNA p6904	0.672155	0.003447	0.055978
lncRNA p38815_v4	0.669775	0.043694	0.210795
lncRNA p29455	0.668381	0.010842	0.099069
lncRNA p30103	0.667757	0.039991	0.200873
lncRNA p26086	0.66595	0.047601	0.220548
LOC100506293	0.663937	0.024379	0.153254
lncRNA p13540	0.663627	0.029733	0.170098
lncRNA p37048_v4	0.662939	0.020353	0.138594
lncRNA p14432	0.662057	0.016017	0.121978
lncRNA p34635_v4	0.658131	0.044397	0.212906
lncRNA p38899_v4	0.657352	0.011208	0.101148
LOC157860	0.657107	0.037927	0.195131
lncRNA p42398_v4	0.656232	0.046295	0.217732
lncRNA p5853	0.654455	0.007951	0.084484
lncRNA p42182_v4	0.653628	0.011631	0.103098
lncRNA p7855	0.653362	0.040713	0.202567
lncRNA p17125	0.65243	0.033018	0.180446
lncRNA p26013	0.651036	0.01604	0.122043
lncRNA p44037_v4	0.649108	0.023878	0.151726
LOC81691	0.648789	0.021076	0.141306
lncRNA p22595	0.64792	0.017736	0.129369
lncRNA p2857	0.645629	0.016802	0.125493
lncRNA p33466	0.645051	0.047869	0.221242
lncRNA p41740_v4	0.644598	0.026624	0.160401
lncRNA p37779_v4	0.644007	0.01968	0.1362
lncRNA p28873	0.643567	0.010045	0.095204
lncRNA p8888	0.642003	0.033394	0.181593
lncRNA p1396	0.641605	0.02178	0.144163
lncRNA p11518	0.640393	0.026777	0.160864
lncRNA p43246_v4	0.639568	0.004859	0.065103
lncRNA p2327	0.637902	0.031399	0.175633
LOC100287852	0.636005	0.039663	0.199882
lncRNA p6892	0.635561	0.018574	0.132589
lncRNA p36561_v4	0.635143	0.00963	0.093495

lncRNA p3338	0.628217	0.010561	0.097969
lncRNA p26721	0.62655	0.041601	0.204894
lncRNA p15557	0.626216	0.041657	0.20494
lncRNA p19104	0.624472	0.049249	0.22463
lncRNA p5288	0.624073	0.010379	0.097007
lncRNA p1207	0.622731	0.01769	0.129116
lncRNA p2342	0.62225	0.014918	0.117211
lncRNA RNA147383|p04	0.622086	0.040434	0.201832
lncRNA p8767	0.621644	0.014321	0.114795
lncRNA p38062_v4	0.6206	0.029796	0.17029
lncRNA p16760	0.620096	0.009836	0.094388
lncRNA RNA33555|snoF	0.619197	0.043006	0.208794
lncRNA p44280_v4	0.618753	0.035202	0.187176
lncRNA p4206	0.61731	0.020546	0.139419
lncRNA p35914_v4	0.617282	0.039794	0.200273
lncRNA p33583	0.616954	0.01725	0.12742
LOC100652965	0.614438	0.030524	0.172697
lncRNA RNA95937|RNS	0.614357	0.03254	0.179037
lncRNA RNA33784|snoF	0.612741	0.018109	0.130631
lncRNA p6463	0.610958	0.047517	0.220325
lncRNA p13420	0.609705	0.044738	0.213841
lncRNA p30027	0.608722	0.009885	0.09459
lncRNA RNA147108|p02	0.60872	0.045908	0.216911
lncRNA p2467	0.608347	0.035743	0.188977
lncRNA p24922	0.605438	0.034428	0.184719
lncRNA p18757	0.604764	0.041179	0.203831
lncRNA p20607	0.601	0.01994	0.137111
lncRNA p9506	-0.60005	0.03329	0.18127
lncRNA p25063	-0.6002	0.037521	0.193897
lncRNA RNA95546|RNS	-0.60091	0.020689	0.14005
lncRNA p37529_v4	-0.60095	0.038054	0.195535
lncRNA p36224_v4	-0.60116	0.007071	0.079342
lncRNA p279	-0.6012	0.019818	0.136676
lncRNA p4707	-0.60327	0.022855	0.148087
LOC100506422	-0.60401	0.033706	0.182369
lncRNA p36870_v4	-0.60449	0.04853	0.222954
lncRNA p26436	-0.60514	0.038839	0.1975
lncRNA p15	-0.60523	0.048677	0.223236
lncRNA p36998_v4	-0.60561	0.042008	0.205787
lncRNA p23336	-0.60569	0.047844	0.221215
lncRNA p28564	-0.60619	0.02285	0.148087
lncRNA p23743	-0.607	0.033646	0.182209
lncRNA p11054	-0.60741	0.048711	0.223356
lncRNA p27119	-0.60768	0.034416	0.184715
lncRNA p5829	-0.60793	0.036229	0.190498
lncRNA p37927_v4	-0.60913	0.038964	0.197811
lncRNA p20473	-0.60913	0.027379	0.163085

lncRNA p12615	-0.60921	0.041804	0.205207
LOC100130890	-0.60953	0.030779	0.173398
lncRNA p15499	-0.6096	0.041437	0.20468
lncRNA p25776	-0.60989	0.030656	0.173144
lncRNA p10658	-0.61028	0.043148	0.20915
lncRNA p15142	-0.61038	0.035138	0.187032
lncRNA p772	-0.61039	0.012242	0.106095
lncRNA p40691_v4	-0.61076	0.027973	0.164797
lncRNA p35412_v4	-0.61191	0.039961	0.20084
lncRNA p15140	-0.61194	0.029239	0.168355
lncRNA p11675	-0.6121	0.038331	0.196164
lncRNA p33731	-0.61272	0.026136	0.158766
LOC100134663	-0.61314	0.028055	0.165062
lncRNA p9889	-0.61341	0.027577	0.16368
lncRNA p33476	-0.61365	0.045805	0.216601
lncRNA p39120_v4	-0.614	0.049423	0.225039
lncRNA p25394	-0.6141	0.035293	0.187385
lncRNA p36871_v4	-0.61448	0.023628	0.150647
lncRNA p9388	-0.61476	0.042867	0.208359
lncRNA p38479_v4	-0.61488	0.030759	0.173359
lncRNA p40770_v4	-0.61517	0.010301	0.096494
lncRNA p6623	-0.61542	0.025608	0.157319
lncRNA p6917	-0.61726	0.039422	0.199157
lncRNA p6422	-0.61801	0.028993	0.167542
lncRNA p11239	-0.6182	0.024578	0.154018
lncRNA p34998_v4	-0.62101	0.035246	0.187291
lncRNA p44496_v4	-0.62119	0.023511	0.150252
lncRNA p11249	-0.62164	0.024052	0.152178
lncRNA p41772_v4	-0.62603	0.046643	0.218422
lncRNA p14356	-0.62661	0.025853	0.157816
lncRNA p42056_v4	-0.62686	0.012244	0.106095
lncRNA p20535	-0.62691	0.018855	0.133693
lncRNA p27293	-0.62752	0.013145	0.109738
lncRNA p23238	-0.62807	0.049708	0.225502
lncRNA p22694	-0.62812	0.021031	0.14109
LOC100128703	-0.62923	0.041899	0.205435
lncRNA p2848	-0.63007	0.013797	0.112541
lncRNA p43049_v4	-0.63013	0.010453	0.097323
lncRNA p25293	-0.63156	0.04876	0.22355
lncRNA p24088	-0.63178	0.040999	0.203299
lncRNA p25451	-0.63419	0.013591	0.111628
lncRNA p10796	-0.6346	0.019042	0.134291
lncRNA p6175	-0.6347	0.031456	0.175831
lncRNA p25647	-0.63514	0.015345	0.119072
lncRNA p28672	-0.63554	0.006211	0.074134
lncRNA RNA143468|tRN	-0.63773	0.047497	0.220325
lncRNA p16494	-0.6378	0.023982	0.152013

lncRNA p36458_v4	-0.63783	0.036841	0.192126
lncRNA p36012_v4	-0.63819	0.029938	0.170749
lncRNA p15601	-0.63856	0.034187	0.183981
lncRNA p7873	-0.63874	0.048525	0.222954
lncRNA p13100	-0.63881	0.011967	0.105052
lncRNA p37700_v4	-0.63913	0.043321	0.209507
lncRNA p37026_v4	-0.63928	0.034967	0.186448
lncRNA p4648	-0.64071	0.018827	0.133677
lncRNA p36738_v4	-0.64097	0.021655	0.143534
lncRNA p33729	-0.64208	0.016867	0.125833
lncRNA p7997	-0.64259	0.027138	0.162226
lncRNA p34969_v4	-0.64312	0.044821	0.214027
lncRNA RNA147109|p0:	-0.64388	0.038738	0.197286
lncRNA p22437	-0.6443	0.005656	0.070489
lncRNA p35360_v4	-0.64462	0.032414	0.17855
lncRNA p7074	-0.64572	0.046686	0.218496
lncRNA p33796	-0.64618	0.025517	0.156943
lncRNA p18033	-0.64687	0.028951	0.167451
lncRNA p35063_v4	-0.64695	0.024232	0.152737
lncRNA p17963	-0.64755	0.027008	0.161761
lncRNA p4955	-0.64781	0.044427	0.212993
lncRNA p2847	-0.64834	0.034351	0.184511
lncRNA p11490	-0.64925	0.029624	0.169718
lncRNA p13771	-0.6496	0.038077	0.195604
lncRNA p10228	-0.65032	0.037294	0.19324
lncRNA p24126	-0.65035	0.013297	0.110288
lncRNA p27949	-0.65046	0.02886	0.167231
lncRNA p39410_v4	-0.65108	0.02493	0.155103
lncRNA p2732	-0.65182	0.047207	0.219616
lncRNA p23344	-0.65229	0.024737	0.154478
lncRNA p3406	-0.65231	0.01803	0.130435
lncRNA p36194_v4	-0.65237	0.021971	0.144831
lncRNA p4459	-0.65246	0.043853	0.21134
lncRNA p23875	-0.6527	0.014385	0.115042
lncRNA p287	-0.65281	0.025026	0.155464
lncRNA RNA143504|tRN	-0.65311	0.040819	0.202865
lncRNA p11131	-0.65314	0.036481	0.191195
lncRNA p8136	-0.65594	0.01291	0.108883
lncRNA p7136	-0.65614	0.027755	0.164217
lncRNA p28070	-0.65634	0.020639	0.139827
lncRNA p11509	-0.65734	0.024486	0.15366
lncRNA p36392_v4	-0.65745	0.036433	0.191003
lncRNA p4686	-0.65755	0.015483	0.119809
lncRNA p38768_v4	-0.65811	0.020706	0.140092
lncRNA p6620	-0.65921	0.023028	0.148584
lncRNA p28936	-0.6593	0.01566	0.12049
lncRNA p38004_v4	-0.6593	0.035893	0.189533

lncRNA p25052	-0.65967	0.015433	0.119535
lncRNA p33615	-0.66028	0.019568	0.135962
lncRNA p23761	-0.66044	0.038202	0.196013
lncRNA p12059	-0.66064	0.047313	0.219788
lncRNA p8374	-0.66119	0.036828	0.192126
lncRNA p6626	-0.66179	0.011496	0.102643
lncRNA p28388	-0.664	0.035449	0.187894
lncRNA p1883	-0.66436	0.013968	0.113217
lncRNA RNA147491|p0!	-0.66597	0.042807	0.20822
lncRNA p4256	-0.66601	0.041489	0.204693
lncRNA p14881	-0.66641	0.033437	0.181702
lncRNA p18034	-0.66691	0.021798	0.144196
lncRNA p16616	-0.66704	0.006529	0.076129
lncRNA p20421	-0.66725	0.012229	0.106095
lncRNA p20265	-0.66741	0.014581	0.115893
lncRNA RNA33609|snoF	-0.66772	0.028312	0.165908
lncRNA p36994_v4	-0.66793	0.039165	0.198417
lncRNA p39207_v4	-0.66903	0.017403	0.127993
lncRNA p3412	-0.66916	0.011529	0.102643
lncRNA p33950	-0.66936	0.034427	0.184719
lncRNA p22967	-0.66989	0.025852	0.157816
lncRNA p15803	-0.67016	0.012286	0.106279
lncRNA p15302	-0.67157	0.030714	0.173282
lncRNA p12231	-0.6724	0.030613	0.173013
lncRNA p5516	-0.6727	0.03228	0.178067
lncRNA p23629	-0.67273	0.012805	0.108641
lncRNA p33940	-0.67364	0.044066	0.211893
lncRNA p36915_v4	-0.67398	0.017748	0.129371
lncRNA p40171_v4	-0.67501	0.014926	0.117224
lncRNA p29568	-0.67751	0.036793	0.192025
lncRNA p9578	-0.67826	0.024651	0.154199
lncRNA p34785_v4	-0.67902	0.013809	0.112541
lncRNA p12194	-0.67981	0.026745	0.160816
lncRNA p18275	-0.68121	0.023939	0.151942
lncRNA p33681	-0.68203	0.026024	0.158242
lncRNA p36165_v4	-0.68257	0.02712	0.162226
lncRNA p9234	-0.68262	0.047856	0.221215
lncRNA p36948_v4	-0.6833	0.007774	0.08343
lncRNA p25768	-0.68465	0.010243	0.096215
lncRNA p41456_v4	-0.68508	0.00654	0.076231
lncRNA p1956	-0.68508	0.024907	0.155018
lncRNA p766	-0.68525	0.032843	0.179826
lncRNA p33687	-0.68552	0.029019	0.167594
lncRNA p14882	-0.68663	0.013142	0.109738
lncRNA p16635	-0.68795	0.024584	0.154018
lncRNA p10758	-0.68819	0.039725	0.200013
LOC100287497	-0.68844	0.018437	0.131912

lncRNA p27237	-0.68875	0.014555	0.115796
LOC100131829	-0.68933	0.005803	0.071488
lncRNA p30124	-0.68935	0.037658	0.194374
lncRNA RNA143545|tRN	-0.68947	0.027364	0.163082
lncRNA p38731_v4	-0.6903	0.037862	0.194945
lncRNA p23670	-0.69067	0.014799	0.116811
lncRNA p7516	-0.6908	0.042335	0.206849
lncRNA p6786	-0.69198	0.036869	0.192187
lncRNA p5692	-0.69204	0.023247	0.149198
lncRNA p21177	-0.69214	0.03201	0.177236
lncRNA p28242	-0.69246	0.031513	0.176062
lncRNA p28533	-0.69341	0.033618	0.182172
lncRNA p27250	-0.69498	0.007911	0.084219
lncRNA p16634	-0.69536	0.046388	0.217955
lncRNA p2000	-0.69806	0.013751	0.112395
lncRNA RNA143543|tRN	-0.69913	0.023248	0.149198
lncRNA p26303	-0.70018	0.030434	0.172613
lncRNA p612	-0.70058	0.018686	0.133016
lncRNA p26600	-0.70088	0.014583	0.115893
lncRNA p411	-0.70261	0.01252	0.10739
lncRNA p15167	-0.70291	0.014389	0.115044
lncRNA p29709	-0.70327	0.033271	0.181194
lncRNA p41988_v4	-0.7052	0.028316	0.165908
lncRNA p6079	-0.70598	0.045486	0.215813
lncRNA p18827	-0.70608	0.033027	0.180446
lncRNA RNA143456|tRN	-0.7061	0.048789	0.223615
lncRNA p27769	-0.70727	0.02565	0.157431
lncRNA p15141	-0.70736	0.010431	0.097191
lncRNA p43084_v4	-0.70747	0.043753	0.21102
lncRNA p283	-0.70979	0.018456	0.13199
lncRNA RNA143477|tRN	-0.7107	0.043961	0.211609
lncRNA p771	-0.7117	0.047519	0.220325
lncRNA p34574_v4	-0.71182	0.02007	0.137476
lncRNA RNA143479|tRN	-0.71397	0.028602	0.166604
lncRNA p26774	-0.71455	0.016822	0.125613
lncRNA p11110	-0.7149	0.005002	0.066001
lncRNA p10661	-0.71564	0.031528	0.176094
lncRNA p8519	-0.71598	0.043452	0.20996
lncRNA p22596	-0.71647	0.025923	0.158075
lncRNA p40215_v4	-0.71672	0.013039	0.109332
lncRNA RNA95839|RNS	-0.71747	0.034914	0.18633
lncRNA p29453	-0.71859	0.014748	0.116573
lncRNA p6965	-0.71885	0.043168	0.209188
lncRNA p16685	-0.71958	0.042766	0.20814
lncRNA p22325	-0.71959	0.038327	0.196164
lncRNA p24799	-0.7196	0.013396	0.110759
lncRNA p11012	-0.72002	0.006365	0.075178

lncRNA p6105	-0.7207	0.040083	0.201072
lncRNA p21046	-0.72206	0.01728	0.127531
lncRNA RNA143486|tRN	-0.72226	0.021135	0.14153
lncRNA p34620_v4	-0.72301	0.006068	0.07299
lncRNA p35408_v4	-0.72304	0.031763	0.176667
lncRNA p1323	-0.72445	0.013385	0.110721
lncRNA p30253	-0.72449	0.024114	0.152335
lncRNA p1393	-0.7246	0.009169	0.090863
lncRNA p20738	-0.72472	0.040734	0.202583
lncRNA p19617	-0.72529	0.045005	0.214754
lncRNA p3411	-0.72535	0.014726	0.116497
lncRNA p6405	-0.72638	0.044512	0.213219
lncRNA p23150	-0.72663	0.02437	0.153254
lncRNA p38754_v4	-0.72691	0.026057	0.158347
lncRNA p16096	-0.72694	0.004787	0.06486
lncRNA p33438	-0.72699	0.037059	0.192482
lncRNA p5276	-0.72801	0.028716	0.166859
lncRNA p36459_v4	-0.72862	0.018284	0.131239
lncRNA p7205	-0.72879	0.01744	0.128124
lncRNA p34257_v4	-0.72902	0.019892	0.136968
lncRNA p280	-0.72925	0.011645	0.103196
lncRNA p29067	-0.73007	0.047047	0.219216
lncRNA p25960	-0.73022	0.0176	0.128757
lncRNA p42057_v4	-0.73189	0.010442	0.097274
lncRNA p24093	-0.73191	0.016065	0.122209
lncRNA p29249	-0.73208	0.026832	0.161032
lncRNA RNA147585|p06	-0.73232	0.04431	0.212758
lncRNA p33602	-0.73258	0.034604	0.185367
lncRNA p3740	-0.73267	0.035165	0.187095
lncRNA p4820	-0.73293	0.012346	0.106519
lncRNA p29452	-0.7336	0.021106	0.141448
lncRNA p26584	-0.73491	0.011551	0.102715
LOC100506613	-0.73511	0.023856	0.151648
lncRNA p11353	-0.73531	0.014046	0.11368
lncRNA p4108	-0.73584	0.015142	0.118129
lncRNA p21788	-0.73727	0.027021	0.161779
lncRNA RNA143539|tRN	-0.73739	0.036882	0.192204
lncRNA p17456	-0.73757	0.030248	0.171934
lncRNA p14357	-0.73883	0.036412	0.190936
lncRNA p12978	-0.74013	0.033561	0.182038
lncRNA p23066	-0.74146	0.040612	0.202215
lncRNA p43526_v4	-0.74236	0.011357	0.10188
lncRNA p1746	-0.74257	0.016297	0.123194
lncRNA p21787	-0.74258	0.020754	0.140181
lncRNA p1119	-0.74373	0.027413	0.163144
lncRNA p24911	-0.74438	0.013408	0.110771
lncRNA p5465	-0.74497	0.017979	0.130209

lncRNA p729	-0.7458	0.023943	0.151942
lncRNA p44265_v4	-0.74669	0.009319	0.091808
lncRNA p11925	-0.74671	0.018025	0.130432
lncRNA RNA143541|tRN	-0.74743	0.037545	0.193905
lncRNA p20985	-0.74795	0.012971	0.109053
lncRNA RNA147048|p0:	-0.74823	0.049261	0.224654
lncRNA p10962	-0.74921	0.022625	0.147207
lncRNA p12100	-0.75001	0.005313	0.068007
lncRNA p25103	-0.75006	0.024185	0.152554
lncRNA p24747	-0.75124	0.016629	0.124665
lncRNA p18707	-0.75206	0.03514	0.187032
lncRNA p6733	-0.75262	0.009598	0.093383
lncRNA p28294	-0.75376	0.0479	0.221331
lncRNA p33559	-0.75454	0.030135	0.171469
lncRNA p1669	-0.75508	0.028249	0.165775
lncRNA p33418	-0.75529	0.04816	0.22207
lncRNA p14451	-0.75531	0.01373	0.112283
lncRNA p17326	-0.75601	0.024533	0.153886
lncRNA p2401	-0.75604	0.011289	0.101642
lncRNA p26861	-0.75694	0.024154	0.152448
lncRNA p28086	-0.75822	0.022463	0.146636
lncRNA p87	-0.75825	0.006855	0.078137
lncRNA p42510_v4	-0.75844	0.039529	0.199417
lncRNA p12099	-0.75912	0.044315	0.212758
lncRNA p5684	-0.75952	0.043283	0.209409
lncRNA p24432	-0.76036	0.01718	0.127182
lncRNA p1076	-0.76083	0.003266	0.054535
lncRNA p18705	-0.76144	0.043102	0.209016
LOC100134138	-0.76171	0.03178	0.176704
lncRNA p13752	-0.76283	0.024591	0.154018
lncRNA p36014_v4	-0.76395	0.008703	0.088385
lncRNA RNA147809|p09	-0.76471	0.047757	0.22105
lncRNA p10091	-0.76527	0.008577	0.087891
lncRNA p23879	-0.76582	0.047598	0.220548
lncRNA p6598	-0.7661	0.02733	0.163056
lncRNA p13775	-0.7672	0.027012	0.161761
lncRNA p25947	-0.76752	0.040861	0.202889
lncRNA p23513	-0.768	0.028593	0.166601
lncRNA p28562	-0.76814	0.038908	0.197714
lncRNA p25520	-0.7693	0.026541	0.160189
lncRNA p16937	-0.76958	0.008173	0.085655
lncRNA p25526	-0.77075	0.049277	0.224696
lncRNA RNA95983|RNS_	-0.77225	0.021865	0.1445
lncRNA p6262	-0.77252	0.020986	0.140928
lncRNA p11410	-0.77318	0.030859	0.17363
lncRNA p6233	-0.77374	0.034377	0.184595
lncRNA RNA147002|p0:	-0.77418	0.019769	0.136503

lncRNA p23178	-0.77443	0.041493	0.204693
lncRNA p26247	-0.77461	0.023893	0.151768
lncRNA p28718	-0.77464	0.047762	0.22105
lncRNA p11007	-0.77493	0.006121	0.073472
lncRNA p16759	-0.7751	0.029018	0.167594
lncRNA p13738	-0.77637	0.014941	0.117242
lncRNA p38031_v4	-0.77646	0.028022	0.164987
lncRNA p24103	-0.7769	0.024089	0.15227
lncRNA p7916	-0.77731	0.049502	0.225115
LOC100130560	-0.77815	0.013663	0.111949
lncRNA p36540_v4	-0.77818	0.039735	0.200036
lncRNA p40022_v4	-0.77884	0.009351	0.091962
lncRNA p29789	-0.78055	0.024749	0.154491
lncRNA p1409	-0.78084	0.028757	0.166926
lncRNA p24981	-0.78233	0.011723	0.10364
lncRNA RNA95037|RNS_	-0.78275	0.009463	0.092615
lncRNA p284	-0.78291	0.021379	0.142666
lncRNA RNA95442|RNS_	-0.78346	0.037532	0.193897
lncRNA p11216	-0.78394	0.022775	0.147757
lncRNA p16495	-0.78461	0.018199	0.130931
lncRNA RNA143548|tRN	-0.78496	0.030665	0.173152
lncRNA RNA95042|RNS_	-0.78685	0.016591	0.124605
lncRNA p29436	-0.78691	0.049082	0.224081
lncRNA p2353	-0.78703	0.012758	0.10844
lncRNA p2398	-0.78958	0.010654	0.098307
lncRNA p38733_v4	-0.79018	0.028915	0.167356
lncRNA p20090	-0.79038	0.008996	0.089856
lncRNA p15139	-0.7914	0.028894	0.167315
lncRNA p35062_v4	-0.79197	0.008189	0.085794
lncRNA p3080	-0.79238	0.046413	0.218042
lncRNA p9563	-0.79246	0.037292	0.19324
lncRNA p33607	-0.79293	0.02098	0.140912
lncRNA p12585	-0.79347	0.02289	0.148161
lncRNA p4855	-0.79425	0.034109	0.183713
lncRNA p33708	-0.79442	0.012084	0.105673
lncRNA p25499	-0.79496	0.032397	0.178483
lncRNA RNA143540|tRN	-0.79518	0.022559	0.146985
lncRNA p5196	-0.79534	0.001891	0.041871
lncRNA p39424_v4	-0.79584	0.010485	0.097537
lncRNA p33670	-0.79586	0.003293	0.054806
lncRNA p29643	-0.79599	0.002687	0.049328
lncRNA p2397	-0.79602	0.017827	0.129604
LOC100505894	-0.79615	0.032692	0.17935
lncRNA p6719	-0.79633	0.014079	0.11372
lncRNA p2203	-0.79635	0.005711	0.070886
lncRNA RNA33528|snoR	-0.79783	0.002444	0.047424
lncRNA p11378	-0.7979	0.019382	0.135363

lncRNA p1895	-0.79793	0.029873	0.17055
lncRNA p5823	-0.79793	0.007527	0.081894
lncRNA p36997_v4	-0.7981	0.031054	0.17428
lncRNA p43472_v4	-0.79833	0.039403	0.199106
lncRNA p23345	-0.79865	0.042865	0.208359
lncRNA p8828	-0.79915	0.008888	0.089264
lncRNA p8307	-0.80048	0.002991	0.052302
lncRNA p23014	-0.80107	0.022029	0.145118
lncRNA p8514	-0.80253	0.009657	0.093559
LOC100129408	-0.80267	0.008289	0.086144
lncRNA p7646	-0.80331	0.036018	0.189865
lncRNA p37721_v4	-0.80408	0.033644	0.182209
lncRNA p44160_v4	-0.80419	0.009328	0.091842
LOC100131860	-0.80537	0.008112	0.085282
lncRNA p37423_v4	-0.80539	0.030526	0.172697
lncRNA p36131_v4	-0.80595	0.023135	0.148861
lncRNA p10755	-0.80674	0.021601	0.143242
lncRNA p26676	-0.80675	0.015973	0.121757
lncRNA p30090	-0.80677	0.008453	0.08702
lncRNA p6096	-0.80747	0.007545	0.082036
lncRNA RNA33771|snoR	-0.80748	0.023125	0.148861
lncRNA p23350	-0.80753	0.028713	0.166859
lncRNA p17455	-0.80804	0.019293	0.13505
lncRNA p43279_v4	-0.80807	0.036896	0.192204
lncRNA p34794_v4	-0.80965	0.026616	0.160382
lncRNA p28794	-0.80965	0.005404	0.068506
lncRNA RNA143542|tRN	-0.81022	0.015854	0.121285
lncRNA p9270	-0.81026	0.03753	0.193897
lncRNA p37863_v4	-0.81473	0.014835	0.116887
lncRNA p6782	-0.81593	0.038162	0.195925
lncRNA p36092_v4	-0.81675	0.01343	0.110876
lncRNA p28100	-0.81745	0.033024	0.180446
lncRNA p13834	-0.81834	0.019651	0.136116
lncRNA p16302	-0.81905	0.012444	0.107096
lncRNA p33356	-0.81984	0.005305	0.067996
lncRNA p7697	-0.8202	0.026213	0.159104
lncRNA p34085_v4	-0.82188	0.023178	0.149032
lncRNA p23347	-0.82258	0.03998	0.200849
lncRNA p14563	-0.82372	0.005237	0.067654
lncRNA p2959	-0.82407	0.009615	0.093422
lncRNA p14656	-0.82474	0.038487	0.196601
lncRNA p1184	-0.8254	0.024191	0.152563
lncRNA p3550	-0.82556	0.010158	0.095735
lncRNA p35064_v4	-0.82616	0.047444	0.220186
lncRNA p35336_v4	-0.82649	0.003836	0.058887
lncRNA p3063	-0.82652	0.021252	0.142176
lncRNA p25799	-0.82662	0.032459	0.178709

lncRNA p24907	-0.827	0.012514	0.107363
lncRNA p18615	-0.82737	0.010564	0.097969
lncRNA p410	-0.82804	0.00505	0.06639
lncRNA p17737	-0.82813	0.028993	0.167542
lncRNA p37274_v4	-0.82881	0.004293	0.061646
lncRNA p43854_v4	-0.82906	0.020752	0.140181
lncRNA p34651_v4	-0.82908	0.01552	0.119876
lncRNA p38831_v4	-0.83016	0.007419	0.08111
lncRNA p7784	-0.83254	0.0102	0.095963
lncRNA p8376	-0.83367	0.008897	0.089272
lncRNA p44240_v4	-0.834	0.018861	0.133695
lncRNA p37173_v4	-0.83452	0.047003	0.219095
lncRNA p33945	-0.835	0.001596	0.038966
lncRNA p9695	-0.83518	0.024591	0.154018
lncRNA p43864_v4	-0.8354	0.029198	0.168238
lncRNA p19151	-0.83586	0.007304	0.080399
lncRNA p28781	-0.83599	0.018595	0.132672
lncRNA p11489	-0.83605	0.023197	0.149088
lncRNA p36293_v4	-0.83617	0.012945	0.109006
lncRNA p42183_v4	-0.83619	0.013871	0.112808
LOC100128402	-0.8371	0.018283	0.131239
lncRNA p8855	-0.83765	0.005351	0.068135
lncRNA p33827	-0.83911	0.025328	0.15637
lncRNA p26697	-0.83962	0.015429	0.11953
lncRNA p26380	-0.83968	0.013234	0.109983
LOC100509196	-0.83989	0.043633	0.210594
lncRNA p36627_v4	-0.84165	0.025995	0.158225
lncRNA p30191	-0.84192	0.005751	0.071191
lncRNA p33486	-0.84291	0.047203	0.219616
lncRNA p36519_v4	-0.84366	0.025407	0.156599
lncRNA p22197	-0.84439	0.023532	0.150291
lncRNA p26215	-0.84482	0.002117	0.044615
lncRNA p7871	-0.84691	0.014252	0.114522
lncRNA p29642	-0.84913	0.008254	0.085952
lncRNA p14160	-0.84917	0.009969	0.094881
lncRNA p11827	-0.84984	0.012049	0.105499
lncRNA p6591	-0.85125	0.03216	0.177819
lncRNA p12258	-0.85128	0.010826	0.099047
lncRNA p41150_v4	-0.85137	0.009292	0.091706
lncRNA p33773	-0.85425	0.008814	0.088866
lncRNA p9612	-0.85497	0.015024	0.117557
lncRNA p6621	-0.85644	0.003298	0.054867
lncRNA p26903	-0.85728	0.040634	0.202289
lncRNA RNA95688|RNS	-0.85754	0.019688	0.1362
lncRNA p30233	-0.85823	0.040806	0.20285
lncRNA p3173	-0.86047	0.010655	0.098307
lncRNA p758	-0.86143	0.01379	0.112541

lncRNA p5570	-0.8615	0.008257	0.085952
lncRNA p37474_v4	-0.86154	0.012523	0.107391
lncRNA p40314_v4	-0.86249	0.018615	0.132735
lncRNA p26488	-0.86266	0.021913	0.14456
lncRNA RNA147697|p08	-0.863	0.011165	0.101088
lncRNA p22255	-0.8653	0.046448	0.218119
lncRNA p30229	-0.86568	0.021746	0.143991
lncRNA p35965_v4	-0.8657	0.033199	0.180969
lncRNA p33557	-0.867	0.008096	0.085191
lncRNA p43648_v4	-0.86753	0.006178	0.073903
lncRNA p2809	-0.86844	0.012781	0.108545
lncRNA p27837	-0.86866	0.009341	0.091895
lncRNA p24760	-0.86873	0.032835	0.179826
lncRNA p30119	-0.87013	0.040639	0.202289
lncRNA p38953_v4	-0.87059	0.049418	0.225039
lncRNA p17419	-0.87151	0.004786	0.06486
lncRNA p26882	-0.87229	0.004893	0.065355
lncRNA p22084	-0.87239	0.038685	0.197117
lncRNA p6744	-0.87531	0.013641	0.111879
lncRNA p25317	-0.87556	0.024488	0.15366
lncRNA p6437	-0.87587	0.004966	0.065855
lncRNA p3030	-0.87676	0.004912	0.065496
lncRNA p37375_v4	-0.877	0.046099	0.217215
lncRNA p9119	-0.87817	0.010392	0.097047
lncRNA p21696	-0.87858	0.037038	0.192412
lncRNA p618	-0.87866	0.048964	0.223866
lncRNA p12192	-0.87877	0.007525	0.081893
lncRNA p944	-0.88075	0.03303	0.180446
lncRNA p40312_v4	-0.88083	0.002027	0.0436
lncRNA p39801_v4	-0.8811	0.026009	0.158242
lncRNA p36013_v4	-0.88115	0.039202	0.198464
lncRNA p33794	-0.88139	0.01524	0.118582
lncRNA p37600_v4	-0.88201	0.010428	0.097191
lncRNA p26898	-0.88205	0.021353	0.142623
lncRNA p35324_v4	-0.88281	0.000969	0.030507
lncRNA p23916	-0.88319	0.018389	0.131634
lncRNA p18900	-0.88345	0.041458	0.20468
lncRNA RNA33529|snoF	-0.8837	0.019258	0.135001
lncRNA p43498_v4	-0.88497	0.014801	0.116811
lncRNA p41190_v4	-0.887	0.007556	0.082111
lncRNA p43307_v4	-0.88735	0.015032	0.11759
lncRNA p26260	-0.88921	0.007076	0.079374
lncRNA p40624_v4	-0.88948	0.00304	0.052587
lncRNA p1944	-0.89262	0.034918	0.18633
lncRNA RNA147563|p0(	-0.89311	0.014355	0.114957
lncRNA p6613	-0.89553	0.007909	0.084219
lncRNA p29370	-0.89636	0.007828	0.083791

lncRNA p43056_v4	-0.89746	0.006616	0.076742
lncRNA p23268	-0.89795	0.041769	0.205207
lncRNA p38077_v4	-0.8987	0.005511	0.069443
lncRNA p20362	-0.89979	0.006415	0.075471
lncRNA p17358	-0.90157	0.002965	0.052177
lncRNA p1208	-0.90175	0.04653	0.21832
lncRNA p14886	-0.90271	0.026189	0.159001
lncRNA p1993	-0.90289	0.002814	0.050761
lncRNA p44161_v4	-0.90502	0.013956	0.113217
lncRNA p8586	-0.91026	0.01971	0.136298
LOC100131581	-0.91395	0.022204	0.145573
lncRNA p40181_v4	-0.91527	0.002985	0.052281
lncRNA p19553	-0.9153	0.043787	0.211149
lncRNA p42790_v4	-0.916	0.009931	0.094699
lncRNA p945	-0.91805	0.026347	0.159558
lncRNA p20140	-0.92028	0.003174	0.053514
lncRNA p24369	-0.92088	0.002633	0.049127
lncRNA p30211	-0.92099	0.038328	0.196164
lncRNA RNA147065|p0:	-0.92159	0.015051	0.117707
lncRNA p42175_v4	-0.92247	0.010168	0.095749
lncRNA p8133	-0.92466	0.018709	0.133119
lncRNA p1168	-0.92541	0.028756	0.166926
lncRNA p33444	-0.92664	0.041449	0.20468
lncRNA p38730_v4	-0.92667	0.007516	0.081826
lncRNA p11485	-0.92738	0.020802	0.140339
lncRNA p39687_v4	-0.92756	0.011196	0.101136
lncRNA p17099	-0.9287	0.015793	0.120988
lncRNA p13169	-0.93408	0.028288	0.165855
lncRNA p5877	-0.9341	0.020821	0.140341
lncRNA p13519	-0.93448	0.033564	0.182038
lncRNA p29895	-0.93548	0.016644	0.124725
lncRNA p11491	-0.93557	0.003901	0.059333
lncRNA p1193	-0.93574	0.020804	0.140339
lncRNA p43076_v4	-0.93604	0.047081	0.219318
lncRNA p29367	-0.93688	0.006692	0.077118
lncRNA p36015_v4	-0.9369	0.012557	0.107531
lncRNA p4761	-0.93957	0.00673	0.077367
lncRNA p24493	-0.9408	0.008356	0.086413
lncRNA p20888	-0.94134	0.02892	0.167356
lncRNA p8727	-0.94227	0.034207	0.184064
lncRNA p3852	-0.94286	0.003058	0.052731
lncRNA p16768	-0.94338	0.02855	0.166551
lncRNA p29352	-0.94523	0.003041	0.052587
lncRNA p29384	-0.94571	0.011739	0.103758
lncRNA p3633	-0.94572	0.020478	0.1391
lncRNA p10939	-0.94605	0.038839	0.1975
lncRNA p4555	-0.94642	0.03221	0.177892

lncRNA p24424	-0.94903	0.003863	0.05913
LOC100507653	-0.95002	0.042886	0.208392
LOC284757	-0.95077	0.005584	0.070055
lncRNA p5476	-0.95196	0.016376	0.123566
lncRNA p26332	-0.95243	0.003877	0.059152
lncRNA p20460	-0.95269	0.010335	0.09669
lncRNA p13220	-0.95299	0.002222	0.045511
lncRNA p24847	-0.95505	0.017072	0.126669
lncRNA p22419	-0.95511	0.025779	0.157627
lncRNA p6561	-0.95605	0.006229	0.074171
lncRNA p29429	-0.95662	0.029312	0.168663
lncRNA p11486	-0.95778	0.010451	0.097323
lncRNA p28928	-0.95805	0.018787	0.133479
lncRNA p26475	-0.9593	0.004036	0.06025
lncRNA p29096	-0.96043	0.004623	0.063995
lncRNA p28217	-0.96144	0.003418	0.055793
lncRNA p38377_v4	-0.96238	0.008885	0.089255
lncRNA p25364	-0.96527	0.014738	0.116562
lncRNA p28947	-0.96802	0.048951	0.223866
lncRNA p44476_v4	-0.96814	0.018084	0.130608
lncRNA p41061_v4	-0.96843	0.003825	0.05888
lncRNA p4518	-0.96862	0.008137	0.085442
lncRNA p21794	-0.96866	0.003985	0.059874
lncRNA p37078_v4	-0.96869	0.004984	0.06592
lncRNA p21772	-0.96971	0.011461	0.10254
lncRNA p40742_v4	-0.97184	0.026461	0.15986
lncRNA p30131	-0.97201	0.044591	0.213513
LOC100128402	-0.97231	0.004836	0.064972
lncRNA p7786	-0.9727	0.003819	0.058821
lncRNA p42545_v4	-0.97369	0.002506	0.047996
lncRNA p36988_v4	-0.97413	0.039472	0.199273
lncRNA p36986_v4	-0.97413	0.034115	0.183713
lncRNA p1479	-0.97413	0.028704	0.166859
lncRNA p27939	-0.97418	0.041909	0.205435
lncRNA p20480	-0.97481	0.001494	0.03785
lncRNA p29420	-0.97767	0.019132	0.134534
lncRNA p20266	-0.97787	0.00259	0.048857
lncRNA p17610	-0.97819	0.02846	0.166316
lncRNA p33968_v4	-0.9802	0.003968	0.059698
lncRNA p21278	-0.98041	0.015898	0.121429
lncRNA p34100_v4	-0.98172	0.0491	0.224136
lncRNA p330	-0.98214	0.015193	0.118353
lncRNA RNA95944|RNS	-0.98232	0.004649	0.064123
lncRNA p1194	-0.98266	0.035108	0.186968
lncRNA p22151	-0.98394	0.0215	0.14299
lncRNA p15145	-0.98486	0.022861	0.148087
lncRNA p11851	-0.98553	0.026378	0.15969

lncRNA p40179_v4	-0.98789	0.009668	0.093601
lncRNA p5828	-0.98904	0.004776	0.064811
lncRNA p23349	-0.98961	0.02505	0.155464
lncRNA p9792	-0.98973	0.002394	0.047116
lncRNA p11134	-0.99045	0.010772	0.098751
lncRNA p1852	-0.99379	0.027304	0.162982
lncRNA p26477	-0.99444	0.016493	0.124121
lncRNA p27533	-0.99512	0.003497	0.056475
lncRNA p36991_v4	-0.99675	0.00067	0.026049
lncRNA p26547	-0.99704	0.044593	0.213513
lncRNA p15595	-0.99877	0.030518	0.172697
lncRNA p42889_v4	-0.99942	0.008291	0.086144
lncRNA p5544	-1.00011	0.006286	0.074605
lncRNA p19254	-1.00054	0.030483	0.172629
lncRNA p25026	-1.00061	0.012143	0.105891
lncRNA p35257_v4	-1.00084	0.007543	0.082036
lncRNA p13841	-1.00175	0.038345	0.196173
lncRNA p6153	-1.00194	0.020748	0.140181
lncRNA p35189_v4	-1.00211	0.040651	0.202318
lncRNA p6068	-1.00218	0.036634	0.19164
lncRNA p40694_v4	-1.00255	0.03594	0.189617
lncRNA p33522	-1.00274	0.008316	0.086215
lncRNA p17334	-1.00299	0.011321	0.101796
lncRNA p16633	-1.00766	0.000612	0.025233
LOC100128402	-1.00843	0.006567	0.076439
lncRNA p9580	-1.00905	0.002968	0.052177
lncRNA p43654_v4	-1.00976	0.047489	0.220325
lncRNA p9482	-1.01112	0.037537	0.193897
lncRNA p2561	-1.01112	0.048584	0.223019
LOC100507904	-1.01183	0.004073	0.060389
lncRNA p23352	-1.01223	0.008949	0.089626
lncRNA p26217	-1.01275	0.010273	0.096404
lncRNA p23016	-1.01302	0.032993	0.180446
lncRNA p10041	-1.01453	0.0119	0.104742
lncRNA p23057	-1.01567	0.003366	0.055387
lncRNA p39412_v4	-1.01582	0.018905	0.133798
lncRNA p9714	-1.01635	0.028883	0.167308
lncRNA p43527_v4	-1.01824	0.025595	0.157319
lncRNA p11926	-1.01884	0.032253	0.178036
lncRNA p43179_v4	-1.02197	0.004033	0.060235
lncRNA p12385	-1.02241	0.013614	0.111685
lncRNA p40324_v4	-1.0242	0.001105	0.032497
lncRNA p11098	-1.02608	0.001817	0.041279
lncRNA p24892	-1.02683	0.006224	0.074171
lncRNA p40113_v4	-1.02814	0.004727	0.06467
lncRNA p35875_v4	-1.02928	0.003941	0.059525
lncRNA p1998	-1.02959	0.004937	0.065599

lncRNA p22923	-1.03047	0.025675	0.157446
lncRNA p26717	-1.0319	0.004075	0.060389
lncRNA p13709	-1.03302	0.024062	0.152178
lncRNA p12977	-1.03563	0.030984	0.174003
lncRNA p5296	-1.03628	0.007901	0.084171
lncRNA p42074_v4	-1.03724	0.006628	0.076777
lncRNA p43046_v4	-1.04068	0.026291	0.1594
lncRNA p13985	-1.04106	0.04751	0.220325
lncRNA p29548	-1.04168	0.017183	0.127182
lncRNA p22274	-1.04219	0.001326	0.035448
lncRNA p10040	-1.04237	0.00945	0.092534
lncRNA p18158	-1.04263	0.029698	0.169923
lncRNA p38121_v4	-1.04282	0.002489	0.047855
lncRNA p3461	-1.04445	0.04233	0.206849
lncRNA p11550	-1.04453	0.021905	0.14456
lncRNA p13419	-1.04466	0.045292	0.215333
lncRNA p473	-1.04641	0.033366	0.181536
lncRNA p22280	-1.04653	0.044075	0.211909
lncRNA RNA95477|RNS	-1.04739	0.002172	0.045213
lncRNA p20126	-1.04785	0.005106	0.066712
lncRNA p15266	-1.04794	0.001721	0.040321
lncRNA p24257	-1.04823	0.003888	0.059198
lncRNA p43846_v4	-1.04832	0.00016	0.014104
lncRNA p20627	-1.04978	0.032606	0.17914
lncRNA p23018	-1.04995	0.020979	0.140912
lncRNA RNA143454|tRN	-1.05046	0.003895	0.059287
LOC100131581	-1.05076	0.004221	0.061246
lncRNA p21938	-1.05117	0.00552	0.06948
lncRNA p9577	-1.05227	0.003398	0.055607
lncRNA p5275	-1.05337	0.001519	0.038036
lncRNA p30348	-1.05605	0.010374	0.096985
lncRNA p29485	-1.05745	0.039708	0.200013
lncRNA p13809	-1.05873	0.028566	0.166551
lncRNA p41349_v4	-1.05882	0.017745	0.129371
lncRNA p13399	-1.06019	0.009363	0.092032
lncRNA p17081	-1.06113	0.028154	0.165388
lncRNA p40322_v4	-1.06161	0.001464	0.03745
lncRNA p5581	-1.06212	0.001091	0.032307
lncRNA p34	-1.06372	0.002106	0.044438
lncRNA p36979_v4	-1.06429	0.008227	0.085952
LOC650293	-1.06542	0.004189	0.061077
lncRNA p13381	-1.06672	0.013041	0.109332
lncRNA p33792	-1.0686	0.003871	0.05913
lncRNA p16036	-1.06868	0.001822	0.041294
lncRNA p3009	-1.06873	0.003867	0.05913
lncRNA RNA147224|p0	-1.06902	0.004236	0.061254
lncRNA RNA147248|p0	-1.07106	0.015113	0.118089

lncRNA p26885	-1.07141	0.047966	0.221449
lncRNA p17776	-1.07192	0.002903	0.051746
lncRNA p18745	-1.07307	0.003562	0.056863
lncRNA p12238	-1.07331	0.023032	0.148584
lncRNA p5981	-1.07363	0.014429	0.115225
lncRNA p37542_v4	-1.07391	0.005418	0.06861
lncRNA p3407	-1.07532	0.014114	0.113869
lncRNA p12198	-1.07541	0.028165	0.165394
LOC100132495	-1.07553	0.00136	0.03585
lncRNA p30144	-1.07748	0.004899	0.065378
lncRNA p5966	-1.07813	0.040823	0.202865
lncRNA p27505	-1.08141	0.014649	0.116177
lncRNA p35647_v4	-1.0817	0.016753	0.125263
lncRNA p9197	-1.0817	0.041712	0.205086
lncRNA p22420	-1.08208	0.020374	0.138594
lncRNA p37619_v4	-1.0826	0.003705	0.05804
lncRNA p1335	-1.08313	0.017756	0.129375
lncRNA p37617_v4	-1.08577	0.030779	0.173398
lncRNA p1051	-1.08852	0.025345	0.156414
lncRNA p8762	-1.08969	0.025049	0.155464
lncRNA p39610_v4	-1.09226	0.031833	0.176811
lncRNA p27443	-1.09488	0.004183	0.061053
lncRNA p12196	-1.09642	0.045909	0.216911
lncRNA p24942	-1.09783	0.006049	0.072987
lncRNA p37764_v4	-1.09797	0.002305	0.046325
LOC100507206	-1.0981	0.01386	0.112799
lncRNA p24092	-1.10028	0.001704	0.040108
lncRNA p26805	-1.10029	0.001803	0.041212
lncRNA p15549	-1.1011	0.001941	0.042526
lncRNA p16734	-1.10213	0.017438	0.128124
lncRNA p24119	-1.10436	0.002048	0.043794
lncRNA p7317	-1.10626	0.010026	0.095141
lncRNA p8028	-1.10709	0.008522	0.087517
lncRNA p33953_v4	-1.10742	0.003416	0.055793
lncRNA p1308	-1.10875	0.010154	0.095735
lncRNA p17356	-1.10879	0.002705	0.049441
lncRNA p33914	-1.10967	0.008923	0.089425
lncRNA p14327	-1.11031	0.030137	0.171469
lncRNA p5907	-1.11097	0.000451	0.021714
lncRNA RNA95752|RNS	-1.11114	0.004608	0.063878
lncRNA p19686	-1.11232	0.014278	0.114619
lncRNA p34738_v4	-1.11233	0.008368	0.086432
lncRNA p768	-1.11295	0.003006	0.052334
lncRNA p41958_v4	-1.113	0.035964	0.189667
lncRNA RNA147196|p0	-1.11375	0.043108	0.209016
lncRNA p17601	-1.11411	0.007571	0.08221
lncRNA p9007	-1.11493	0.021567	0.143225

lncRNA p26187	-1.11788	0.017339	0.127802
lncRNA p6930	-1.1213	0.016139	0.122549
lncRNA p3842	-1.1215	0.011604	0.102939
lncRNA p3409	-1.12202	0.007731	0.083095
LOC100128402	-1.12297	0.002446	0.047424
lncRNA p34516_v4	-1.12459	0.008647	0.088079
lncRNA p35663_v4	-1.1248	0.046861	0.218889
lncRNA p25757	-1.12504	0.00349	0.056475
lncRNA p25999	-1.127	0.001896	0.041878
lncRNA p28988	-1.12719	0.023599	0.150528
lncRNA p28951	-1.1273	0.019821	0.136676
lncRNA p38694_v4	-1.12917	0.016433	0.123836
lncRNA p10540	-1.13124	0.013697	0.112118
lncRNA p3059	-1.1326	0.020264	0.138424
lncRNA p6579	-1.13322	0.007202	0.079903
LOC100509860	-1.1335	0.015905	0.121429
lncRNA p2166	-1.1355	0.005881	0.071957
lncRNA p33793	-1.1364	0.000485	0.022369
lncRNA p15617	-1.13648	0.000302	0.018054
lncRNA p3733	-1.13659	0.012787	0.108545
lncRNA RNA147794|p0[illegible]	-1.13718	0.002346	0.046631
lncRNA p3260	-1.13734	0.011345	0.10185
lncRNA p19982	-1.14483	0.018205	0.130942
LOC100129113	-1.14728	0.002115	0.044609
lncRNA p25519	-1.14772	0.007423	0.08111
LOC100653058	-1.15135	0.011477	0.102601
lncRNA p11906	-1.15198	0.034264	0.184251
lncRNA p42963_v4	-1.15263	0.002072	0.044137
lncRNA p37486_v4	-1.15291	0.015738	0.120648
lncRNA p4197	-1.15468	0.00035	0.019259
lncRNA p1649	-1.15788	0.005029	0.06625
lncRNA p26296	-1.15871	0.019858	0.136786
lncRNA p38074_v4	-1.15945	0.000369	0.019751
lncRNA p36244_v4	-1.15957	0.002204	0.045424
lncRNA p12558	-1.16133	0.017752	0.129373
lncRNA p5596	-1.16154	0.001685	0.039823
lncRNA p15144	-1.16197	0.00363	0.057335
LOC100509490	-1.16295	0.001128	0.032831
lncRNA RNA147830|p0[illegible]	-1.16407	0.003265	0.054535
lncRNA p9809	-1.16736	0.012941	0.109001
lncRNA p6008	-1.16787	0.0303	0.17214
lncRNA p42960_v4	-1.16824	0.001717	0.040279
lncRNA p18748	-1.17673	0.005655	0.070489
lncRNA p23015	-1.17719	0.012497	0.107306
lncRNA p30190	-1.17781	0.001541	0.038346
lncRNA p28539	-1.17805	0.004108	0.060697
lncRNA p15629	-1.18229	0.011149	0.101053

lncRNA p12535	-1.18305	0.022655	0.147347
lncRNA p20112	-1.18418	0.01484	0.116904
lncRNA p20620	-1.18489	0.000528	0.023421
lncRNA p3841	-1.1856	0.000205	0.015344
lncRNA p39114_v4	-1.18995	0.00054	0.023708
lncRNA p6044	-1.19025	0.007062	0.079294
lncRNA p40931_v4	-1.1917	0.019535	0.13582
lncRNA p13740	-1.19716	0.001886	0.041804
LOC729468	-1.19816	0.014037	0.11366
lncRNA p13755	-1.19842	0.002388	0.047067
lncRNA p25508	-1.19954	9.25E-05	0.010731
lncRNA p26264	-1.19969	0.028269	0.165807
lncRNA p6931	-1.20012	0.014753	0.116573
lncRNA p9689	-1.2004	0.011195	0.101136
lncRNA p21101	-1.20091	0.007198	0.079902
lncRNA p24460	-1.20364	0.015446	0.119609
lncRNA p23019	-1.204	0.007621	0.082497
lncRNA p35068_v4	-1.20589	0.01914	0.134534
lncRNA p35067_v4	-1.20659	0.030194	0.171669
lncRNA p13398	-1.20687	0.01496	0.117243
lncRNA p42972_v4	-1.20794	0.001409	0.036593
lncRNA p17602	-1.20838	0.002544	0.048373
lncRNA p601	-1.20848	0.013471	0.111027
lncRNA p6491	-1.20986	0.000148	0.013508
lncRNA p11351	-1.21383	0.000305	0.018061
lncRNA p33420	-1.21403	0.032796	0.179743
lncRNA p41492_v4	-1.21489	0.003864	0.05913
lncRNA p1501	-1.21872	0.008768	0.088638
lncRNA p4825	-1.22029	0.005517	0.06948
lncRNA p29904	-1.22172	0.003804	0.058751
lncRNA p20247	-1.22259	0.001393	0.036353
lncRNA p43301_v4	-1.22347	0.000437	0.021436
lncRNA p200	-1.22499	0.00018	0.014718
lncRNA p33429	-1.2266	0.017461	0.128225
lncRNA p5569	-1.22868	0.004064	0.060389
lncRNA p12195	-1.22893	0.008632	0.088001
LOC100130931	-1.2303	0.001058	0.031635
lncRNA p8730	-1.23031	0.015281	0.118744
lncRNA p43028_v4	-1.23087	0.009	0.089856
lncRNA p7386	-1.23108	0.000403	0.020525
lncRNA p19769	-1.23519	0.004774	0.064811
LOC100127885	-1.23538	0.000455	0.021751
lncRNA p2624	-1.23954	0.005638	0.070425
lncRNA p1238	-1.23991	0.005308	0.068001
lncRNA p29343	-1.24217	0.000432	0.021319
lncRNA p25471	-1.24452	0.039338	0.198984
lncRNA p20124	-1.24704	0.00218	0.045298

lncRNA p37080_v4	-1.24707	0.002598	0.048978
lncRNA p6340	-1.24921	0.008031	0.084842
lncRNA p16098	-1.25416	0.006863	0.078174
lncRNA p3100	-1.25544	0.012144	0.105891
lncRNA p34820_v4	-1.25578	0.002162	0.045072
lncRNA p12144	-1.25746	0.003366	0.055387
lncRNA p29733	-1.25798	0.014117	0.113869
lncRNA p39893_v4	-1.25975	0.000347	0.019206
lncRNA p387	-1.26151	0.013969	0.113217
lncRNA p43909_v4	-1.26198	0.006619	0.076742
lncRNA p29114	-1.26392	0.008257	0.085952
lncRNA p35760_v4	-1.26577	0.010774	0.098751
lncRNA p14264	-1.26724	0.044987	0.2147
lncRNA p184	-1.26924	0.015173	0.118288
lncRNA p41491_v4	-1.27103	0.001246	0.034391
lncRNA p13472	-1.27415	0.046595	0.21841
lncRNA p5547	-1.27488	0.000743	0.027304
lncRNA p43175_v4	-1.27698	0.006063	0.07299
lncRNA p6787	-1.2774	0.000352	0.01927
lncRNA p13754	-1.27947	0.022396	0.146372
lncRNA p28097	-1.28321	0.006529	0.076129
lncRNA p19771	-1.28325	0.000784	0.028165
lncRNA p33788	-1.28387	0.001032	0.031318
lncRNA p26010	-1.28682	0.03902	0.197974
lncRNA p38960_v4	-1.28932	0.003567	0.056863
lncRNA p39119_v4	-1.2906	0.000809	0.028585
lncRNA p401	-1.29127	0.016205	0.122825
lncRNA p4647	-1.29403	0.009831	0.094374
LOC100131023	-1.29886	0.001644	0.039533
lncRNA p88	-1.30033	0.00197	0.042764
lncRNA p37430_v4	-1.30295	0.004806	0.064885
LOC100130913	-1.30392	0.004138	0.06089
lncRNA p26189	-1.30709	0.002154	0.045072
lncRNA p43169_v4	-1.30816	0.000431	0.021319
lncRNA p26017	-1.30963	0.002574	0.04873
lncRNA p34712_v4	-1.31125	0.012916	0.108883
lncRNA p36153_v4	-1.31135	0.030968	0.173948
lncRNA p34460_v4	-1.31453	0.043288	0.209409
lncRNA p40411_v4	-1.31555	0.009874	0.094517
lncRNA p37432_v4	-1.31643	0.008342	0.086331
lncRNA p37431_v4	-1.31941	0.00064	0.025633
lncRNA p33484	-1.32288	0.006391	0.07539
lncRNA p12500	-1.32322	0.011173	0.101088
lncRNA p3472	-1.32576	0.003109	0.053118
lncRNA p30177	-1.32885	0.002192	0.045346
lncRNA p23708	-1.32962	0.003135	0.053222
lncRNA p20098	-1.33236	0.016924	0.125954

lncRNA p18739	-1.3324	0.015392	0.119295
lncRNA p5068	-1.33336	0.017081	0.126669
lncRNA p574	-1.33613	0.03618	0.190299
lncRNA p4861	-1.33694	0.002289	0.04628
lncRNA p27722	-1.33763	0.006394	0.07539
lncRNA p20246	-1.3382	0.000356	0.019285
lncRNA p34066_v4	-1.33904	0.045925	0.216911
lncRNA p23755	-1.33975	0.03137	0.175527
lncRNA p44394_v4	-1.34008	0.035547	0.188233
lncRNA RNA95497|RNS	-1.3404	0.002546	0.048373
LOC100132292	-1.34186	0.00534	0.068052
lncRNA p22649	-1.34245	0.004244	0.061255
lncRNA p5543	-1.34342	0.004168	0.061014
lncRNA p39115_v4	-1.34811	0.000219	0.015622
lncRNA p6707	-1.34968	0.001444	0.037149
lncRNA p20125	-1.35059	0.002625	0.049127
lncRNA p5079	-1.3512	0.001022	0.031203
lncRNA p43055_v4	-1.35247	0.008165	0.085655
lncRNA p1716	-1.35351	0.006877	0.078237
lncRNA p24815	-1.35418	0.008672	0.088239
lncRNA p5922	-1.35755	0.012685	0.108185
lncRNA p13844	-1.36179	0.015737	0.120648
lncRNA p21533	-1.3622	0.003925	0.059452
LOC100131043	-1.36513	0.007879	0.084152
lncRNA p34857_v4	-1.36649	0.005561	0.06986
lncRNA p5580	-1.36651	0.003064	0.052777
lncRNA p5675	-1.36739	0.005392	0.068461
lncRNA p41490_v4	-1.36897	0.000945	0.030238
lncRNA p22083	-1.36942	0.001411	0.036614
lncRNA p11964	-1.37201	0.00046	0.021838
lncRNA p25806	-1.37223	0.02196	0.144783
lncRNA p1522	-1.3738	0.007207	0.07993
lncRNA p34980_v4	-1.37429	0.000354	0.01927
lncRNA p28219	-1.37565	0.000272	0.017383
LOC100653245	-1.37801	0.027139	0.162226
lncRNA RNA95763|RNS	-1.37925	0.008926	0.089428
lncRNA p16791	-1.3817	0.01273	0.108297
lncRNA RNA147325|p0	-1.38472	0.001546	0.038414
lncRNA p183	-1.38508	0.000673	0.026049
lncRNA p28968	-1.38526	0.006979	0.078857
lncRNA p33941	-1.38755	0.042574	0.207534
lncRNA p39670_v4	-1.38969	0.013201	0.109952
lncRNA p26869	-1.39038	0.003975	0.05977
lncRNA p1239	-1.3909	0.009684	0.093601
lncRNA p12332	-1.39504	0.002267	0.046206
lncRNA p34012_v4	-1.40013	0.001549	0.038417
lncRNA p37345_v4	-1.40167	0.000322	0.018425

lncRNA p22648	-1.40405	0.004074	0.060389
lncRNA p40425_v4	-1.40522	0.00401	0.06005
lncRNA p15295	-1.40625	0.013021	0.109298
lncRNA p28791	-1.40673	0.001222	0.034209
lncRNA p35066_v4	-1.41038	0.007039	0.079167
lncRNA RNA95672|RNS	-1.41077	0.021204	0.141938
lncRNA p20121	-1.41633	0.000599	0.025022
lncRNA p34983_v4	-1.41796	5.84E-05	0.00872
lncRNA p40057_v4	-1.41892	0.000619	0.025381
lncRNA p35075_v4	-1.42409	0.019953	0.137111
lncRNA p29951	-1.4254	0.001497	0.037879
lncRNA p26194	-1.42712	0.000261	0.017247
lncRNA p13820	-1.42795	0.000766	0.027769
lncRNA p20248	-1.42808	0.000203	0.015344
lncRNA p15628	-1.42953	0.003338	0.055282
LOC653720	-1.43151	0.000415	0.020982
lncRNA p27205	-1.43422	4.59E-05	0.00762
lncRNA p16031	-1.4363	0.010784	0.0988
lncRNA p18546	-1.43665	0.048653	0.223236
lncRNA p11051	-1.43666	0.009307	0.091774
lncRNA p25322	-1.43762	0.027662	0.163974
lncRNA p15025	-1.44029	0.003475	0.056299
lncRNA p24812	-1.44183	0.001114	0.032577
lncRNA p8637	-1.44678	0.000401	0.020525
lncRNA p6933	-1.44714	0.009605	0.093396
lncRNA p24494	-1.45237	0.018615	0.132735
lncRNA p36451_v4	-1.45326	0.0076	0.082424
lncRNA p33855	-1.46001	0.005679	0.070659
lncRNA p7877	-1.46054	0.001092	0.032307
lncRNA p27931	-1.46777	0.002704	0.049441
lncRNA p15922	-1.46886	0.008544	0.087666
lncRNA p39041_v4	-1.472	0.003101	0.053118
lncRNA p5243	-1.47209	0.00077	0.02784
lncRNA p12169	-1.47497	0.038762	0.19737
lncRNA p27692	-1.47662	0.005041	0.066354
lncRNA RNA147249|p0	-1.47982	0.013465	0.111027
lncRNA p2727	-1.48032	0.001202	0.034037
lncRNA p24852	-1.48567	0.005291	0.067917
lncRNA p4442	-1.4861	0.000651	0.025813
lncRNA p8857	-1.48733	0.000143	0.013308
LOC100505908	-1.48762	0.014673	0.116248
lncRNA p9224	-1.48829	0.005915	0.072171
LOC100652777	-1.49032	0.000216	0.015622
lncRNA p23880	-1.49165	0.010633	0.098185
lncRNA p12767	-1.498	0.001686	0.039823
lncRNA p28075	-1.50421	0.020066	0.137476
lncRNA p29407	-1.50923	0.002292	0.04628

lncRNA p40395_v4	-1.51354	0.00683	0.077932
lncRNA p5143	-1.5192	0.005895	0.072055
lncRNA p29018	-1.52407	0.000676	0.026073
lncRNA p39488_v4	-1.53505	0.000661	0.025981
lncRNA p39331_v4	-1.53527	0.001851	0.041537
lncRNA p33508	-1.53627	0.028689	0.166859
lncRNA p24628	-1.53941	0.001196	0.034005
lncRNA p4862	-1.54294	0.001711	0.040255
lncRNA RNA95848|RNS	-1.54358	0.001382	0.036117
lncRNA p18895	-1.54388	0.000176	0.014575
lncRNA p33923	-1.54625	0.001502	0.037931
lncRNA p14372	-1.54748	0.001247	0.034391
lncRNA p7771	-1.55188	0.006891	0.078363
lncRNA p36972_v4	-1.55238	0.029872	0.17055
lncRNA p8760	-1.55247	0.016901	0.125954
lncRNA p22313	-1.55248	0.008339	0.086331
lncRNA p25618	-1.55736	0.000296	0.018054
lncRNA p5642	-1.55767	6.77E-05	0.009307
lncRNA p9225	-1.55987	0.018997	0.134066
lncRNA p14217	-1.56018	0.003653	0.057599
lncRNA p7482	-1.56116	0.001817	0.041279
lncRNA p11060	-1.56286	0.033839	0.18284
lncRNA p19411	-1.56328	0.008872	0.089178
lncRNA p15802	-1.5634	0.000209	0.015388
lncRNA RNA95997|RNS	-1.56649	0.002729	0.049777
lncRNA p36069_v4	-1.56703	0.005094	0.066643
lncRNA p12330	-1.56753	0.003711	0.058094
lncRNA p42936_v4	-1.58602	0.006285	0.074605
lncRNA p38726_v4	-1.58663	0.004485	0.062979
lncRNA p1346	-1.58804	0.002032	0.043623
lncRNA p20240	-1.59171	0.000894	0.029588
lncRNA p20137	-1.59234	0.011278	0.1016
lncRNA p17535	-1.59275	0.002654	0.049172
lncRNA p37546_v4	-1.59279	0.028415	0.166239
lncRNA p9227	-1.59397	0.000206	0.015344
lncRNA p1161	-1.59469	0.039144	0.198417
lncRNA p28804	-1.59567	0.002287	0.04628
lncRNA p39790_v4	-1.59761	0.027476	0.163377
lncRNA p20548	-1.60366	0.000379	0.019839
lncRNA p33919	-1.60403	0.01934	0.13518
lncRNA p19783	-1.6045	0.002604	0.048983
lncRNA p39332_v4	-1.61066	0.001449	0.037201
lncRNA p2982	-1.61074	0.000876	0.029547
lncRNA p35070_v4	-1.61545	0.005984	0.072714
LOC100131581	-1.61646	0.001916	0.042167
lncRNA p3796	-1.61778	1.68E-05	0.004132
lncRNA p41603_v4	-1.62368	7.23E-05	0.00955

lncRNA p19414	-1.62485	0.014964	0.117243
lncRNA p20136	-1.63146	0.006903	0.078443
lncRNA p15393	-1.63327	0.000584	0.024719
lncRNA p9008	-1.63605	0.000963	0.030495
lncRNA p22403	-1.64652	0.010828	0.099047
lncRNA p4864	-1.65698	0.001515	0.03802
lncRNA p26113	-1.65711	0.022167	0.145566
lncRNA p4012	-1.66046	0.00157	0.038615
lncRNA p8732	-1.66271	0.001464	0.03745
lncRNA p28462	-1.66454	0.005805	0.071488
lncRNA p35071_v4	-1.66529	0.006064	0.07299
LOC286161	-1.66903	0.014819	0.116852
lncRNA p1738	-1.6711	0.007375	0.080867
LOC440993	-1.67489	0.00216	0.045072
LOC100507025	-1.68147	0.001508	0.037934
lncRNA p26319	-1.68261	0.001899	0.04192
lncRNA p38755_v4	-1.68783	0.009771	0.09406
lncRNA p22406	-1.69154	0.004384	0.06224
lncRNA p29369	-1.69468	0.002055	0.043896
lncRNA p25901	-1.69741	0.001883	0.041804
lncRNA p33934	-1.69762	0.004229	0.061254
lncRNA p1052	-1.70169	0.000185	0.014888
lncRNA p33918	-1.70286	0.031262	0.175127
lncRNA RNA95034|RNS	-1.71028	0.006027	0.072898
lncRNA p43250_v4	-1.71397	0.000273	0.017383
lncRNA p29125	-1.7233	0.00067	0.026049
lncRNA p2205	-1.73043	0.001765	0.04073
lncRNA p9309	-1.73713	0.043514	0.210138
lncRNA p33891	-1.74002	0.010587	0.097969
lncRNA p29368	-1.74309	0.005303	0.067991
lncRNA p22402	-1.7483	0.001734	0.040395
lncRNA p33507	-1.74854	0.005908	0.072152
lncRNA p35816_v4	-1.76144	0.002613	0.048991
lncRNA p13846	-1.76154	0.015062	0.117741
lncRNA p23261	-1.76874	0.003117	0.05319
lncRNA p37400_v4	-1.77481	0.000201	0.015322
lncRNA p24927	-1.77513	3.32E-05	0.006241
lncRNA p26090	-1.77939	0.046559	0.218365
lncRNA p28338	-1.78331	0.00296	0.052174
lncRNA p35074_v4	-1.78897	0.008783	0.088707
lncRNA p22407	-1.79106	0.006704	0.077154
lncRNA p35815_v4	-1.79434	0.001841	0.041501
lncRNA p14318	-1.80336	0.005665	0.070564
lncRNA p41265_v4	-1.80857	0.002384	0.047032
lncRNA p44165_v4	-1.80965	0.003059	0.052731
lncRNA p37931_v4	-1.81144	0.003847	0.058948
lncRNA p35069_v4	-1.81174	0.004396	0.062301

lncRNA p36497_v4	-1.81416	0.004617	0.063972
LOC100129324	-1.81752	0.000477	0.022218
lncRNA p29139	-1.82625	0.038785	0.197405
lncRNA p33711	-1.82774	0.009645	0.09354
lncRNA p29480	-1.82946	0.019292	0.13505
lncRNA p35376_v4	-1.83896	0.000438	0.021436
lncRNA p33506	-1.84382	0.010178	0.095814
LOC100130811	-1.84651	0.028543	0.166548
lncRNA p28965	-1.84743	0.011785	0.104026
lncRNA p19689	-1.85032	0.008436	0.0869
lncRNA p28780	-1.85279	0.004439	0.062593
lncRNA p22523	-1.85508	0.009443	0.092492
lncRNA p14295	-1.85808	0.001746	0.040395
lncRNA p33457	-1.86652	0.000694	0.026359
lncRNA p44526_v4	-1.87743	0.037201	0.193023
lncRNA p26760	-1.89434	0.021073	0.141306
lncRNA p40555_v4	-1.89567	0.00881	0.088859
lncRNA p38756_v4	-1.90106	3.54E-05	0.006435
lncRNA p33456	-1.92955	0.000623	0.025464
lncRNA p1101	-1.93148	0.007878	0.084152
LOC401847	-1.93769	0.018893	0.133798
lncRNA p41682_v4	-1.93804	0.001266	0.034751
lncRNA p15240	-1.94929	0.002075	0.044137
lncRNA p5140	-1.9532	0.020933	0.1407
lncRNA p6555	-1.95665	0.004307	0.061729
lncRNA p36836_v4	-1.96231	0.000241	0.016366
lncRNA p25660	-1.96266	0.000765	0.027769
lncRNA p22405	-1.97362	0.001651	0.039553
lncRNA RNA95566|RNS	-1.98531	0.000944	0.03022
lncRNA p19695	-1.99384	0.001516	0.03802
lncRNA p2410	-1.99733	0.00181	0.041279
lncRNA p24627	-2.01361	0.000979	0.030662
LOC651536	-2.02922	0.006543	0.076234
lncRNA p12806	-2.02958	0.000224	0.015789
lncRNA p22608	-2.06757	0.004206	0.061095
lncRNA p2983	-2.09204	0.000126	0.012342
lncRNA p5921	-2.11282	0.002161	0.045072
lncRNA p12651	-2.12188	0.00167	0.03973
lncRNA p42366_v4	-2.13367	0.000197	0.015212
lncRNA p5782	-2.13567	0.002085	0.044171
lncRNA p22524	-2.13573	0.006068	0.07299
lncRNA p12621	-2.14862	0.0005	0.022712
lncRNA p42929_v4	-2.14904	0.026805	0.160976
lncRNA p42930_v4	-2.15062	0.017631	0.128911
lncRNA p36036_v4	-2.15254	0.007041	0.079167
lncRNA p14838	-2.17094	0.006494	0.075956
lncRNA p4443	-2.1749	0.000298	0.018054

lncRNA p22404	-2.17516	0.00623	0.074171
lncRNA p10534	-2.1823	0.002083	0.044147
lncRNA p19694	-2.18572	0.001586	0.038893
lncRNA p4428	-2.19531	2.47E-06	0.001684
lncRNA p28776	-2.21335	0.001436	0.037001
lncRNA p42344_v4	-2.22081	0.00024	0.016349
lncRNA p12039	-2.22507	0.006824	0.077932
lncRNA p40499_v4	-2.24325	5.03E-05	0.0081
lncRNA p23260	-2.27697	0.001011	0.031042
lncRNA p26713	-2.34012	0.000169	0.014304
lncRNA p28082	-2.34565	0.002955	0.052121
lncRNA p16629	-2.34684	0.002827	0.050924
lncRNA p11592	-2.35128	0.001548	0.038414
lncRNA p28676	-2.35527	0.00146	0.03741
lncRNA p42655_v4	-2.37891	0.000444	0.0216
lncRNA p36037_v4	-2.38181	0.005923	0.072182
lncRNA p16630	-2.38855	0.005689	0.070732
lncRNA p17436	-2.40828	0.000376	0.019795
lncRNA p9438	-2.41066	0.000538	0.023688
lncRNA p26684	-2.41935	0.027565	0.163676
lncRNA p14861	-2.4388	0.000806	0.028548
lncRNA p42656_v4	-2.43954	0.00049	0.022487
lncRNA p38426_v4	-2.44377	0.000782	0.028107
lncRNA p25903	-2.46301	0.00186	0.041631
lncRNA p7182	-2.46434	0.000319	0.018382
lncRNA p10204	-2.54651	0.000587	0.024775
lncRNA p13842	-2.57666	0.004749	0.064706
lncRNA p29258	-2.59859	0.002291	0.04628
lncRNA p34749_v4	-2.63286	0.002343	0.046631
LOC100653210	-2.64827	0.018922	0.133864
lncRNA p21571	-2.65782	0.000291	0.017929
lncRNA p21182	-2.6847	0.000887	0.029559
lncRNA p21503	-2.70226	0.000905	0.029689
lncRNA p10203	-2.73306	0.007197	0.079902
lncRNA p9437	-2.74284	0.002311	0.046395
lncRNA p9436	-2.77277	0.002416	0.047237
lncRNA p9435	-2.77886	0.001284	0.034997
lncRNA p6436	-2.78507	0.001239	0.034391
lncRNA p21183	-2.79433	0.00079	0.028235
lncRNA RNA143457|tRN	-2.8047	7.57E-05	0.009702
lncRNA p34748_v4	-2.81057	0.00332	0.055082
lncRNA p12499	-2.81665	0.000444	0.0216
lncRNA p25925	-2.89301	0.004081	0.06043
lncRNA p9434	-2.95954	0.001688	0.039823
lncRNA p11319	-2.98159	2.08E-05	0.004494
lncRNA p25346	-3.06741	8.12E-05	0.010024
lncRNA p20355	-3.08187	0.000289	0.017929

lncRNA p16220	-3.12744	1.03E-05	0.003269
lncRNA p42345_v4	-3.15791	0.001349	0.035736
lncRNA p25200	-3.17449	0.000467	0.021958
lncRNA p262	-3.19356	0.012808	0.108641
lncRNA p10205	-3.26443	1.23E-05	0.003513
lncRNA p27381	-3.29996	2.42E-05	0.004912
lncRNA p20353	-3.31869	0.00018	0.014718
lncRNA p33876	-3.37081	0.001039	0.031466
lncRNA p20354	-3.37675	0.000218	0.015622
lncRNA p34576_v4	-3.39792	0.001358	0.03585
lncRNA p22463	-3.45933	9.77E-05	0.011008
lncRNA p28599	-3.49506	1.67E-05	0.004132
lncRNA p21501	-3.50976	0.000693	0.026359
lncRNA p16716	-3.5144	0.000395	0.020321
lncRNA p8237	-3.52283	0.000656	0.025879
lncRNA p16717	-3.52951	0.000343	0.019171
lncRNA p12653	-3.58082	5.85E-05	0.00872
lncRNA p6343	-3.71976	0.001883	0.041804
lncRNA p4715	-3.8548	0.000469	0.021989
lncRNA p33840	-3.893	0.000264	0.017247
LOC646627	-4.79267	0.001453	0.037281
lncRNA p33911	-4.93989	7.23E-06	0.002648
